# Supplementary material for: Yoga and Cardiovascular Health Trial (YACHT): a UK-based randomised mechanistic study of a yoga intervention plus usual care versus usual care alone following an acute coronary event
Source: BMJ Open. 2019 Nov 3;9(11):e030119. doi: 10.1136/bmjopen-2019-030119 (PMC6858127; doi:10.1136/bmjopen-2019-030119)
Supplement: Supplementary data [file bmjopen-2019-030119supp001.pdf]

YACHT

Yoga **A**nd **C**ardiovascular **H**ealth **T**rial

Manual of Operations

Version 1.2

July 2012

Compiled by Barbara Sowa and Claire Tuson

Imperial College London

Table of Contents

STUDY SUMMARY **3**

OUTLINE STUDY DESIGN **4**

YOGA SUMMARY **5**

YOGA FULL DESCRIPTION **5**

PART 1: INITIAL RELAXATION AND WARM-UP – 10 MINUTES 5
Initial relaxation *6*
Warm-up *7*
PART II: ASANAS - 20 MINUTES 8
Standing poses (High positions) *8*
Kneeling & Sitting poses (Medium positions) *10*
Lying down poses (Low positions) *13*
PART III: COOL DOWN: BREATHING EXERCISES AND FINAL RELAXATION - 20 MINUTES 14
Deep Abdominal Breathing *14*
Full Yogic Breath *15*
Final Relaxation *15*
Alternative relaxation *16*
PART IV: SUPERVISION OF PARTICIPANTS POST-EXERCISE AND EDUCATION - 20 MINUTES 18
Proper Exercise *18*
Proper Breathing *18*
Proper Relaxation-Savasana *19*
Proper Diet-Vegetarian – Part I *19*
Proper Diet-Vegetarian – Part II *20*
Positive Thinking and Meditation – Part I *20*
Positive Thinking and Meditation – Part II *21*
Making Positive Changes in Your Life *22*

APPENDIX 1 ..... **23**

POLICY FOR CARDIAC REHABILITATION IN EALING 23

APPENDIX 2 ..... **32**

EALING CARDIAC REHABILITATION HEALTH EDUCATION TALKS 32
Drugs for heart disease and how they work *32*
Managing Stress *34*
Eating for a healthy heart *37*
Exercise and the benefits for your heart *38*
Risk factors and making lifestyle changes *41*
The heart and how it works *44*

REFERENCES **48**

DRAFT

Tillin T, *et al. BMJ Open* 2019; 9:e030119. doi: 10.1136/bmjopen-2019-030119

Imperial College London

Study Summary

TITLE Yoga And Cardiovascular Health Trial (YACHT)

DESIGN Epidemiological

AIMS To perform a mechanistic study to determine the acute and chronic effects of yoga on neuro-endocrine pathways, and downstream effects on CVD risk factors and subclinical outcomes. This will provide complimentary information to a larger clinical trial in India designed to determine effects of yoga on cardiovascular morbidity and mortality in acute coronary syndromes.

POPULATION Indian Asians and Europeans, 40 in each ethnic group, (self-defined, verified by country of birth of all 4 grandparents). Aged between 35 to 80 years, male or female, without co-morbid disease and mobility limitations that would preclude participation in cardiac rehabilitation and our investigations.

ELIGIBILITY Referred to cardiac rehabilitation programmes in West London post-angioplasty as treatment for an acute coronary syndrome. Able to understand English or Punjabi, Hindi or Gujarati, but in order to be able to follow the yoga class instructions the participant will need to have a basic command of the English language.

DURATION Recruitment is planned for 1 year.

DRAFT

Tillin T, *et al. BMJ Open* 2019; 9:e030119. doi: 10.1136/bmjopen-2019-030119

Imperial College London

Outline Study Design

Indian Asians and Europeans, 40 in each ethnic group, (self-defined, verified by country of birth of all 4 grandparents), referred to cardiac rehabilitation programmes in West London post-angioplasty as treatment for an acute coronary syndrome, will be invited to participate when being discharged from hospital.

Those who agree will be randomized to the yoga intervention plus their standard cardiac rehabilitation programme (usual care), or usual care alone.

In order to evaluate the chronic effects of yoga, baseline and 3 month measurements will be performed on all participants as described below:

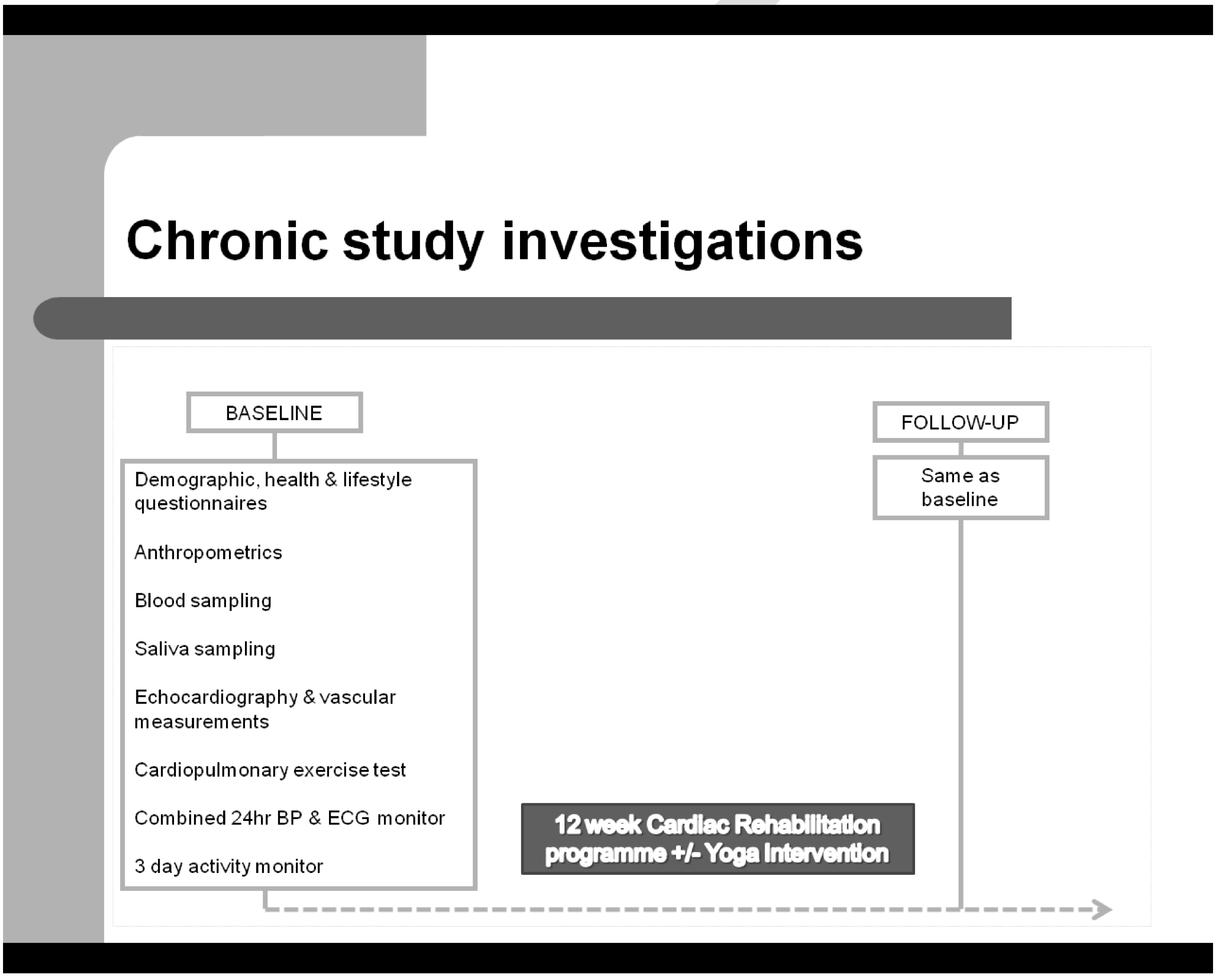

Imperial College London

Yoga Summary

The yoga intervention will be delivered on a bi-weekly group session basis for 12 weeks alongside the cardiac rehabilitation programme. There will be 24 yoga classes in total, of which, each participant will be required to attend a minimum of 18. The yoga session will be designed and conducted by a teacher certified in yoga and cardiac rehabilitation, and will encompass physical fitness (yoga poses), stress reduction (breath control and meditation) and positive lifestyle changes (diet, smoking and alcohol).

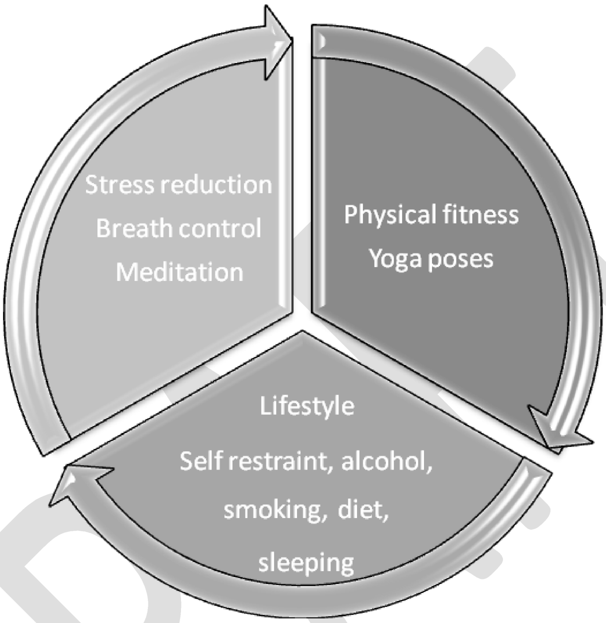

DRAFT

Each class will be approximately 1 hour and 15 minutes and in order to address the balance shown in the above diagram it will consist of the following parts:

- Yogic poses – approx 25 mins
- Breathing exercises and meditation – approx 25 mins
- Education and discussion – approx 25 mins

Yoga Full Description

Part 1: Initial relaxation and warm-up – 10 minutes

Rationale:

Tillin T, *et al. BMJ Open* 2019; 9:e030119. doi: 10.1136/bmjopen-2019-030119

Imperial College London

The initial relaxation and warm-up should be 10 - 15 minutes in duration. Gradual increase in intensity triggers three mechanisms which increase coronary blood flow to match the increased myocardial demand. As a result the ischaemic threshold is extended and the risk of angina and the risk of arrhythmias is reduced.
Due to older average age of this group (compared with mainstream) a gradual progression of range of motion exercises is prescribed.
The short preparatory stretches are included to prepare the muscles for the range of movement involved in Asanas to reduce risk of injury and encourage good balance and alignments. Since incorporating a static stretching will result in fall in heart rate, stretches will be intersperse with some dynamic movement (walking on the spot) designed to maintain the elevated heart rate.

Initial relaxation

1. Emphasise mood, breathing, and relaxation

2. Set the mood for the class. Explain what yoga is, how can help, how should be practiced. Concentration, breathing and relaxation in all yoga practice should be explained and repeated in each class. Put participants in a relaxed state emphasising body position, breathing and relaxation. Use voice to relax them.

3. Begin by finding a comfortable position standing position with your feet hip or shoulder-width apart. You can change positions any time during the relaxation exercises to make yourself more comfortable as needed. Start from breathing. Breathe in slowly and deeply through your nose. Continue to breathe slowly and gently. Allow your breathing to relax you.

The next relaxation exercise focuses on relaxing the muscles of your body.

1. Start with the large muscles of your legs. Tighten all the muscles of your legs. Hold it for a few moments and now relax. Let all the tension go.

2. Now focus on the muscles in your arms. Tighten your shoulders, upper arms, lower arms, and hands. Squeeze your hands into tight fists. Tense the muscles in your arms and hands as tightly as you can. Hold it for a few moments and release. Allow the muscles in your arms to relax completely.

3. Focus again on your breathing. Slow, even, regular breaths. Continue to breathe slowly and rhythmically.

4. Now focus on the muscles of your buttocks. Tighten these muscles as much as you can. Hold this tension and release. Relax your muscles.

5. Tighten the muscles of your back now. Feel your back tightening, pulling your shoulders back and tensing the muscles along your spine. Arch your back slightly as you tighten these muscles. Hold and relax. Let all the tension go. Feel your back comfortably relaxing into a good and healthy posture.

6. Turn your attention now to the muscles of your chest and stomach. Tighten and tense these muscles and release. Relax the muscles of your trunk.

7. Finally, tighten the muscles of your face. Scrunch your eyes shut tightly, wrinkle your nose, and tighten your cheeks and chin. Hold this tension in your face and relax. Release all the tension. Feel how relaxed your face is.

Notice all of the muscles in your body, notice how relaxed your muscles fee l. Allow any last bits of tension to drain away. Enjoy the relaxation you are experiencing. Notice your calm breathing and your relaxed muscles. Enjoy the relaxation for a few moments.

DRAFT

Tillin T, *et al. BMJ Open* 2019; 9:e030119. doi: 10.1136/bmjopen-2019-030119

Imperial College
London

When you are ready to return to your usual level of alertness and awareness, slowly begin to re-awaken your body. Wiggle your toes and fingers. Swing your arms gently. Shrug your shoulders.

Warm-up

1. Now take a few steps on the spot and stand with your feet shoulder-width apart (set position), inhale and bend your knees and do mini-squat, exhale and come back to your set position. Repeat 4 times.

2. Take another few steps on the spot and come back to your set position. Inhale, roll your shoulders up, exhale bring them back and down. Repeat 4 times. During the last repetition hold your shoulders up for 4 to 8 seconds.

3. Take another few steps on the spot and come back to your set position. Inhale, roll your shoulders up, exhale bring them forward and down. Repeat 4 times. During the last repetition hold your shoulders up for 4 to 8 seconds.

4. Take another few steps on the spot and come back to your set position. Inhale, raise your arms to the side (shoulder height), exhale bring them down. Repeat 4 times. During the last repetition hold your arms up for 4 to 8 seconds.

5. Take another few steps on the spot and come back to your set position. Inhale, raise your arms to the front (shoulder height), exhale bring them down. Repeat 2 times. As above add heel raises at the same time if comfortable. Hold the last repetition for 4 to 8 seconds.

6. Take another few steps on the spot and come back to your set position. Inhale, raise your arms to the side (shoulder height), exhale and twist your trunk to the right. Inhale and come back to the centre, exhale and twist your trunk to the left. Repeat 2 times on each side. Hold the last repetition for 4 to 8 seconds.

7. Relax your arms and take another few steps on the spot and come back to set position. Inhale, raise your arms to the side (shoulder height) and look up. Exhale, bring your right arm crossover the chest to the left arm. Inhale, bring your right arm to the side. Exhale, bring your left arm crossover the chest to the right arm. Inhale, bring your left arm to the side. Repeat 2 times on each side. Hold the last repetition for 4 to 8 seconds.

8. Take another few steps on the spot and come back to your set position. Inhale and laterally bend your trunk to the right, exhale and come back to the centre. . Inhale and laterally bend your trunk to the left, exhale and come back to the centre. Repeat 2 times on each side. Hold the last repetition for 4 to 8 seconds.

DRAFT

Tillin T, *et al. BMJ Open* 2019; 9:e030119. doi: 10.1136/bmjopen-2019-030119

Imperial College London

Part II: Asanas - 20 minutes

Rationale:
Gentle progression from high to low positions is prescribed to avoid rapid changes of body positions in order to decrease the risk of arrhythmias or orthostatic hypotension in some individuals. Various ‘chest opener’ Asanas are prescribed to correct the round shouldered posture adopted by the population in response to discomfort in the area of the sterna incision.

Standing poses (High positions)

Mountain pose –Tad asana

1. Come to stand with the big toes touching.

2. Lift up all your toes and let them fan out, then drop them down creating a wide solid base. You can separate your heels slightly if your ankles are knocking together uncomfortably.

3. Bring your weight evenly onto all four corners of both feet.

4. Let the feet and the calves root down into the floor.

5. Engage the quadriceps and draw them upward, causing your knee caps to rise.

6. Rotate both thighs inward, creating a widening of the sit bones, and tuck your tailbone in between the sit bones.

7. Tone the belly, drawing it in slightly.

8. Widen the collar bones and make sure the shoulders are parallel to the pelvis.

9. The neck is long, the crown of the head rises toward the ceiling, and the shoulder blades slide down the back.

10. Hold for 5-10 breaths.

Raised Hands Pose - Urdhva Hastasana

1. From Tadasana, bring your arms out to the side and up.

2. Press the palms together, keep the arms straight and take the gaze up toward your thumbs.

3. Slide the shoulder blades down the back.

4. Maintain your alignment.

5. Hold for 5-10 breaths.

Awkward Chair Pose - Utkatasana

1. From Tadasana bend the knees until the thighs are almost parallel to the floor.

2. Keep the butt low.

DRAFT

3. Bring the arms up towards the ceiling.

4. Bring a slight back bend into the upper back.

5. Hold for 5-10 breaths.

Beginners: Work on bring the thighs closer and closer to parallel to the floor.

Advanced: Try this variation: Bring the hands into a prayer position at the heart. Twist to the right side, bringing the left elbow outside the right knee. Stay low in the pose and keep the knees pressing together. Come back to centre and then do the left side.

Triangle Pose – Trikonasana

1. From Tadasana take a big step backwards with your left leg.

2. Pivot on the ball of the left foot and drop the left heel onto the floor with the toes turned out about 45 degrees from the heel.

3. Bring the arms out to the side.

4. Slide the shoulder blades down the back.

5. Begin to reach the right arm forward, drawing the right thigh upwards and tucking the hip as you come forward.

6. Drop the right hand down to your shin or ankle, or if you are able, onto the floor inside or outside the right foot. Do whichever one feels most comfortable,

7. The left shoulder stacks on top of the right one as you open the chest reaching the left fingertips upwards while keeping the left shoulder rooted in the socket.

8. Take your gaze up towards the left fingertips.

9. Draw the right thigh muscle upwards, deepening the right hip crease.

10. Slightly bend the right knee.

12. Hold for 5-10 breaths

13. Repeat on the left side.

Beginners: Bring the right hand higher up on your leg or use a block on the floor to rest your hand on. It is more important to keep the right leg straight than to bring the right hand to the floor. Do not rest the hand directly on the knee, though, as this creates too much pressure on the knee.

Advanced: Line up the right heel with the arch of the left foot. For a variation, try dropping the left arm over the left ear so it comes parallel to the floor, while keeping the shoulder rooting into the socket.

Tree Pose - Vrksasana

1. Come to stand in Tadasana.

2. Feel your weight equally on all four corners of both feet.

DRAFT

Imperial College London

3. Begin to shift the weight over to the right foot, lifting the left foot off the floor.

4. Bend the left knee, bringing the sole of the left foot high onto the inner right thigh.

5. Press the foot into the thigh and the thigh back into the foot.

6. Try not to let the right hip jut out. Keep both hips squared towards the front.

7. Focus on something that doesn't move to help you keep your balance.

8. Hold for 5-10 breaths.

9. Repeat the move while standing on the left foot.

Beginners: If you cannot bring the left foot high inside the right thigh, bring it lower on the right leg - but be careful to avoid placing the left foot directly on the right knee.

Use the wall for balance if necessary.

Advanced: Bring the arms up towards the ceiling with the palms touching. Open the arms out to side.

Try closing the eyes and see if you can stay balanced.

Kneeling & Sitting poses (Medium positions)

Cat - Cow Stretch - Chakravakasana

1. Start on all fours, bringing the wrists underneath the shoulders and the knees underneath the hips.

2. Think of the spine as a straight line connecting the shoulders to the hips. Try visualizing the line extending forward through the crown of the head and backwards through the tail bone.

3. Keep the neck the natural extension of the spine.

On an inhale:

1. Curl the toes under.

2. Drop the belly.

3. Take the gaze up toward the ceiling.

4. Let the movement in the spine start from the tailbone, so that that neck is the last part to move.

On the next exhale:

1. Release the tops of the feet to the floor.

2. Round the spine.

3. Drop the head.

DRAFT

Tillin T, *et al. BMJ Open* 2019; 9:e030119. doi: 10.1136/bmjopen-2019-030119

Imperial College London

4. Take the gaze to the navel.

5. Repeat the Cat - Cow Stretch on each inhale and exhale, matching the movement to your own breath.

6. Continue for 5-10 breaths, moving the whole spine. After your final exhale, come back to a neutral spine.

Hands and Knees Balance

1. Come on to all fours with the wrists underneath the shoulders and the knees underneath the hips.

2. Extend the right leg to the back of your mat and flex the foot.

3. Lift the right leg up to hip-level, keeping the hips squared towards the floor and the foot flexed.

4. Lift the left arm up to shoulder level.

5. Balance on the left knee and right hand, keeping the spine neutral and the neck long.

5. Stay 5-10 breaths before lowering the lifted hand and knee and doing the other side.

Beginners: Take care not to let the spine collapse while you are balancing.

Advanced: Bend the knee of the lifted leg. Reach around with the lifted arm and hold on to the inside of the lifted foot.

Staff Pose - Dandasana

1. Sit with the legs outstretched straight in front.

2. Engage the thigh muscles and flex the feet. The heels may come up off the floor.

3. Make your spine long.

4. Stack the shoulders directly on top of the hips.

5. Hold for 5-10 breaths.

Beginners: Put padding under your sit bones, if necessary.

Advanced: This pose looks easy, but if you are really working the thighs, you can break a sweat.

Seated Forward Bend - Paschimottanasana

1. From Dandasana bring the arms straight out to the sides and up over your head.

2. Inhale and draw the spine up long.

3. As you exhale, begin to come forward, hinging at the hips.

4. On each inhale, extend the spine, and on each exhale, come a bit farther into the forward bend.

5. Keep the neck at the natural extension of the spine.

6. Do not round the back.

DRAFT

Tillin T, *et al. BMJ Open* 2019; 9:e030119. doi: 10.1136/bmjopen-2019-030119

Imperial College London

7. Take hold of the ankles or shins, whichever you can reach.

8. Hold for 5-10 breaths.

Beginners: Put padding under the sit bones if necessary. Imagine the belly coming to rest on your thighs, rather than the nose coming to the knees - this will help you keep the spine long instead of curving over.

Advanced: If you can easily grab the soles of your feet, try taking a block in front of the feet and holding that instead.

Crab pose- Catuspadapitham

1. From Dandasana, bend the knees bringing the feet flat on the floor hip width apart. Keep the arms behind your hips with the fingers pointed away from your body.

2. Lean back into the arms and slowly inhale and lift the hips up towards the ceiling. Make sure the toes and knees are pointing straight ahead. Look straight ahead, up at the ceiling or carefully drop the head back.

3. Press into the feet, squeezing the thighs and buttocks and engaging Mula Bandha to lift the hips high. Press into the hands and draw the shoulder blades towards each other to lift up high through the sternum.

4. Breathe and hold for 2-6 breaths.

5. To release: slowly exhale the hips back down to the floor.

Beginners: If there is pain or discomfort in the wrists, point the fingers in the opposite direction or make fists with the hands.

Advanced: Inhale one leg up towards the ceiling at a time, pressing out through the heel.

Half Lord of the Fishes Pose (Half Spinal Twist) - Ardha Matsyendrasana

1. From Dandasana, bend your left knee and bring the sole of your left foot to the floor on the outside of the right thigh.

2. Bend the right knee, and tuck the right foot in near the left buttock.

3. Inhale and bring the right arm up near your right ear.

4. Exhale and twist the to the left, bringing the right elbow to the outside the of left knee and the left palm to the floor, just behind your sit bones.

5. Look out over the left shoulder, but don't overturn the neck -- the twist originates in the belly, not the neck.

6. On each inhale, draw the spine long, and on each exhale, twist a little deeper.

7. Be sure to keep the sole of your left foot flat on the floor.

8. Hold for 5-10 breaths.

9. When you release the pose, take a slight counter twist to the opposite direction.

10. Release the legs and switch their position as you prepare to twist to the other side.

DRAFT

Tillin T, *et al. BMJ Open* 2019; 9:e030119. doi: 10.1136/bmjopen-2019-030119

Imperial College London

Beginners: You may want to sit on some padding if you are uncomfortable. If you cannot bend it into the ideal position, you may also keep the right leg extended.

Advanced: Come into a bind with the arms. Thread the right arm back underneath the left knee. Reach the left arm behind your back, and clasp the left wrist with your right hand.

Easy Pose - Sukhasana

1. Arrange padding under your sit bones so that your hips come above your knees.

2. Come to sit in a comfortable, cross-legged position.

3. Bring one heel in towards your groin. The other foot may rest on the floor in front of you or you may bring it into your lap.

4. Root your seat down as your spine grows long. Stack the shoulders over the hips and slide the shoulder blades down your back. The crown of your head rises towards the ceiling.

Neck Exercises

1. In Sukhasana, with your back straight and your chest erect. Slowly bring your head forwards towards the chest to give the back of your neck a good stretch.

2. After a few breaths slowly lift your head and extend your neck back and bring your head to neutral position.

3. Lower your right ear close to your right shoulder, then repeat on the other side. Keep both shoulders level throughout. Repeat the exercise 5 times.

4. Turn your head to the right side. Contract the muscles on the right side of your neck and feel the stretch on the left side. Repeat on the opposite side. Repeat the exercise 5 times.

Lying down poses (Low positions)

Single Leg Lift

1. Lie flat on your back with your legs together, arm next to your body, and palms face down.

2. Inhale and raise your left leg, keeping your knee straight, toes towards your head.

3. Exhale and lower your leg to the starting position.

4. Repeat up to 5 times on the each side.

Head to Knee Raise

1. Start from Single Leg Left Step 2.

2. With an exhalation, bend your left leg and clasp your hands around your knee.

3. With an inhalation, lift your head and try to bring your forehead against your left knee.

4. With an exhalation, lower your head, arms, and leg.

5. Repeat on the opposite side.

DRAFT

Beginners: Keep your head on the floor.

Advanced: Progress to Deep Stretch Single Leg Lift.

1. Start from Single Leg Lift Step 2.

2. With an exhalation, take hold of your leg with both hands, lift your back off the mat and try to bring your chest and head close to the raised leg.

<u>Happy Baby Pose - Ananda Balasana</u>

1. Come to lie on the back.

2. Bend the knees into the chest.

3. Open the knees, bringing them towards the armpits.

4. Stack each ankle directly over the knee, so that the shins are perpendicular to the floor.

5. Flex the feet.

6. Hold the outer edges of the feet at you draw the knees towards the floor.

This pose is appropriate for both beginners and advanced students.

<u>Corpse Pose - Savasana</u>

1. Come to lie down on the back with your arms and feet apart and your eyes closed.

2. Let the feet fall out to either side.

3. Turn the palms to face upwards.

4. Relax the whole body, including the face. Let the body feel heavy.

Part III: Cool Down: Breathing exercises and final relaxation - 20 minutes

Rationale:
Twenty minutes Cool Down/Breathing/Relaxation period is prescribed to reduce risk of hypotension or arrhythmias and to allow the hart rate to return to pre-exercise rates.

Deep Abdominal Breathing and Full Yogic Breath Practice-5 minutes

Deep Abdominal Breathing

1. In Corpse Pose place both hands on your abdomen with your fingers apart.

2. As you inhale, feel your abdomen and hands rising.

3. As you exhale, feel your abdomen and hands sinking.

4. Try to breathe rhythmically, with an inhalation lasting 3-5 seconds and exhalation of the same length.

DRAFT

Imperial College London

Full Yogic Breath

1. In Corpse Pose place one hand on your chest and the other on your abdomen.

2. As you inhale, gradually expand the abdomen, then rise and open the rib cage, and finally lift the collar bones.

3. Begin the exhalation by relaxing the abdomen, then lower the rib cage, and finally slightly contract the abdomen to actively empty the lungs.

Alternate Nostril Breathing (Anuloma Viloma)- 5 minutes

Single Nostril Breathing

In Easy Pose, place your right hand in front of your face in Vishnu Mudra*. Close your right nostril with your thumb. Inhale for three seconds and exhale for six seconds through your left nostril. This is one round. Practice ten rounds. Repeat on the other nostril: close your left nostril with your ring finger, and inhale and exhale through your right nostril.

Beginners: Gradually increase the ratio of the inhalation to the exhalation lengthening to 4:8, 5:10 and 6:12.
Advanced: Progress to Simple Alternate Nostril Breathing

Simple Alternate Nostril Breathing

In Easy Pose close your right nostril with your thumb, inhale through the left nostril for three seconds, close your left nostril with your ring finger, open your right nostril and exhale through it for six seconds. Inhale through your right nostril for three seconds, then exhale through your left nostril for six seconds. Practice for ten rounds. Gradually increase the inhalation: exhalation ratio to 4:8, 5:10 and 6:12.

*Vishnu Mudra: Hold your right hand with the palm facing you and fold the first and second fingers into the palm. Try to keep your thumb and ring fingers straight.

Final Relaxation

1. Inhale and lift your right leg a few inches off the mat. Tense your leg, then exhale and allow your leg to drop. Repeat with the left leg.

2. Inhale and lift both arms a few inches off the mat. Clench your fists, tense your arms, then exhale and allow your arms to drop to the mat.

3. Inhale and lift your hips and buttocks off the mat. Tense your buttocks and then exhale and release.

4. Inhale and lift your chest off the mat. Tense your shoulder blades, then exhale and release.

5. Inhale and pull your shoulders towards your ears. Exhale and release your shoulders.

6. Inhale and squeeze the muscles of your face tightly together. Exhale and release.

7. Inhale, open your mouth, stick your tongue out and look to your forehead. Exhale and release.

8. With an inhalation, slowly roll your head to one side; with an exhalation, roll it to the other side. End by bringing your head back to centre.

DRAFT

Tillin T, *et al. BMJ Open* 2019; 9:e030119. doi: 10.1136/bmjopen-2019-030119

Imperial College
London

Take a few slow rhythmic breaths using your abdomen, then follow this exercise in auto suggestion.

I'm relaxing my feet.....My feet are relaxed.....

I'm relaxing my ankles.....My ankles are relaxed.....

I'm relaxing my calves.....My calves are relaxed.....

I'm relaxing my knees.....My knees are relaxed.....

I'm relaxing my thighs.....My thighs are relaxed.....

I'm relaxing my hips and buttocks.....My hips and buttocks are relaxed.....

I'm relaxing my abdomen and chest.....My abdomen and chest are relaxed.....

I'm relaxing my lower and middle back.....My lower and middle back are relaxed.....

I'm relaxing my shoulders and neck.....My shoulders and neck are relaxed.....

I'm relaxing my hands and fingers.....My hands and fingers are relaxed.....

I'm relaxing my arms.....My arms are relaxed.....

I'm relaxing my mouth and eyes.....My mouth and eyes are relaxed.....

I'm relaxing my facial muscles and scalp.....My facial muscles and scalp are relaxed.....

I'm relaxing my internal organs: my kidneys, my livers, my intestines, my bladder, my pancreas, my stomach, my heart, my lungs and my brain.....My internal organs: my kidneys, my livers, my intestines, my bladder, my pancreas, my stomach, my heart, my lungs and my brain are relaxed.....

Continue abdominal breathing and relaxation. Visualise a calm lake, unruffled by waves. Picture the still water resting on your inner self, which is timeless and unchanging. Continue for a few more minutes.

Then take a few deep breaths, slowly move your legs and arms, and give your whole body a good stretch. Finally bring yourself slowly to sitting cross-legged position. Deeply inhale and exhale. Inhale and bring your hands into a prayer position and as you exhale bow your head thanking everyone for the practice.

Alternative relaxation

In Savasana pose. For the next few moments, focus on calming your mind by focusing on your breathing. Allow you breathing to centre and relax you. Breathe in.... and out.

In..... out.....

In.... Out.....

Continue to breathe slowly and peacefully as you allow the tension to start to leave your body.

Release the areas of tension, feeling your muscles relax and become more comfortable with each breath.

Continue to let your breathing relax you....

DRAFT

Imperial College London

Breathe in....2...3...4.... hold....2.....3...... out...2...3....4...... 5

again....2.....3....4....hold....2....3.... out...2...3....4.... 5

Continue to breathe slowly, gently, comfortably.....

Let the rate of your breathing become gradually slower as your body relaxes.

Now begin to create a picture in your mind of a place where you can completely relax. Imagine what this place needs to be like in order for you to feel calm and relaxed.

Start with the physical layout of the place you are imagining..... where is this peaceful place? You might envision somewhere outdoors.... or indoors..... it may be a small place or large one..... create an image of this place.

(pause)

Now picture some more details about your peaceful place. Who is in this place? Are you alone? Or perhaps you are with someone else? Are there other people present? Animals? Birds? Imagine who is at your place, whether it is you only, or if you have company.

(pause)

Imagine even more detail about your surroundings. Focus now on the relaxing sounds around you in your peaceful place.

Now imagine any tastes and smells your place has to offer.

Imagine the sensations of touch... including the temperature, any breeze that may be present, the surface you are on.... imagine the details of this calming place in your mind.

Focus now on the sights of your place - colours, shapes.... objects.... plants..... water..... all of the beautiful things that make your place enjoyable.

To add further detail to this relaxing scene, imagine yourself there. What would you be doing in this calming place? Perhaps you are just sitting, enjoying this place, relaxing. Maybe you imagine walking around.... or doing any other variety of activities.

Picture yourself in this peaceful place. Imagine a feeling of calm..... of peace..... a place where you have no worries, cares, or concerns.... a place where you can simply rejuvenate, relax, and enjoy just being.

(pause)

Enjoy your peaceful place for a few moments more. Memorize the sights, sounds, and sensations around you. Know that you can return to this place in your mind whenever you need a break. You can take a mental vacation to allow yourself to relax and regroup before returning to your regular roles.

In these last few moments of relaxation, create a picture in your mind that you will return to the next time you need a quick relaxation break. Picture yourself in your peaceful place. This moment you are imagining now, you can picture again the next time you need to relax.

When you are ready to return to your day, file away the imaginary place in your mind, waiting for you the next time you need it.

Turn your attention back to the present. Notice your surroundings as your body and mind return to their usual level of alertness and wakefulness.

DRAFT

Keep with you the feeling of calm from your peaceful place as you return to your everyday life.

Part IV: Supervision of participants post-exercise and education - 20 minutes

Rationale:
Because of an increased risk of arrhythmia and hypotension following exercise, a period of 15-20 minutes supervision is adopted before participants go home. This time will be used for education sessions.

Proper Exercise

Yoga versus physical culture

Aim:

- For the group to understand the importance of taking up physical activity in cardiac rehabilitation
- For the participants to understand that yoga improves not only flexibility but strength, balance and cardiovascular function

Brainstorm question: What are the differences between yoga and physical culture?

Explore the answers and highlight the following:

- Yoga regards the body as a vehicle for the soul in its journey towards perfection
- Yoga promotes gentle movement whereas physical culture emphasises violent muscle movements
- Muscle development does not necessarily mean a healthy body
- Health is a state wherein all organs function perfectly under intelligent control of mind
- Asanas are designed to develop not only the body but also broaden the mental faculties and spiritual capacities
- The body is as young as it is flexible, so yoga postures primary focus on the health of the spine, its strength and flexibility
- Asanas work on the internal machinery of the body, the glands and organs as well as the muscles
- Hand in hand with the practice of yoga postures we practise deep breathing and concentration of the mind

DRAFT

Proper Breathing

Yogic breathing

Observe your breathing for a while. How would you describe your breath?

Ask participants to share their observation and then highlight the following:

- Yoga philosophy claims we are allotted a certain number of breaths per lifetime. How we choose to illustrate that then becomes our practice of longevity. Breathing is the first thing we do when we are born and the last thing we do when we die. Practice observing your breath as often as possible.
- Most people use only a fraction of their potential lung capacity when breathing

Imperial College London

- There are three types of breathing: clavicular, intercostal and deep abdominal.
- A full yogic breath combines all three types of breathing
- Yogic breathing exercises are called pranayama which means to control the prana-subtle energy. Pranayama begins by controlling the motion of the lungs, by which the prana is control.
- Yogic breathing exercises might be very useful in process of quitting smoking

Proper Relaxation-Savasana

Brainstorm question: How do we relax?

Explore the answers and highlight the following:

- When the body and mind and the mind are constantly overworked, their natural efficiency diminishes
- Modern social life and entertainment make it difficult for people to relax by over stimulating the nervous system
- By learning to relax we learn to economise the energy produced by our body as well as regulate and balance the work of the body and mind
- In ordered to achieve perfect relaxation, three methods are used for yogis: physical, mental and spiritual relaxation
- The relaxation position is known as Savasana, the 'Corpse pose'

Proper Diet-Vegetarian – Part I

'You are what you eat'

Discuss and highlight the following:

- Proper yogic diet is lactovegetarian one based on simple, natural and wholesome food
- According to yogic philosophy all of Nature, including our diet, is categorised into three qualities (Gunas): sattvic (pure), rajasic (overstimulating) and tamasic (putrified)
- Sattvic food increases vitality, energy, vigor, health and joy
- Food should be as fresh and natural as possible, preferably organically grown
- Sattvic food include:

  Grains: corn, barley, wheat, unpolished rice, oat, millet and quinoa.
  Grains supply necessary carbohydrates, the main source of energy for the body, and they also contain about half the amino acids that are needed to form protein.
  Protein foods: legumes, nuts and seeds

  Fruits: both fresh and dried, as well as pure fruit juices

DRAFT

Tillin T, *et al. BMJ Open* 2019; 9:e030119. doi: 10.1136/bmjopen-2019-030119

Imperial College London

Vegetables: they contain minerals, vitamins and fibre. There are best eaten raw or cooked as lightly as possible

Herbs: for seasoning and herbal tea

Natural sweeteners: honey, molasses, maple syrup, and apple juice concentrate. White sugar is best avoided in a healthy diet.

Dairy products: milk, butter, cheese and yogurt

Proper Diet-Vegetarian – Part II

Guidelines for healthy eating

Recap from the previous 'Proper diet' session and then highlight the following:

- Always respect your food and maintain a peaceful attitude during meals
- Do not eat when you are angry
- Do not eat food that is too hot or too cold, as this will upset your stomach
- Do not force yourself to eat anything you do not like, but also do not only eat foods that you like the most
- Abandon too many mixtures or combination of foods as they are difficult to digest
- Try to refrain from drinking during meals as this will dilute the gastric juice
- Eat slowly and savour your food
- Eat moderately, do not overload your stomach
- Try to eat at fixed times and try to refrain from eating between meals
- Try not to eat large meals at night
- Take some lemon and honey in the morning for health and energy and to purify the blood
- Do not practise asanas immediately after eating, nor when you are hungry
- Try sitting in Vajra Asana (sitting on the heels with knees and feet together) for 10 minutes after a meal to assist digestion

DRAFT

Positive Thinking and Meditation – Part I

Practical approach to meditation

Discuss and highlight the following:

- Before we can learn to meditate we have to be able to concentrate
- Concentration means attending fully to one thought or object for a substantial length of time

Imperial College London

- Concentration exercises energise the mind, boosting efficiency at work and in the other tasks, while building will-power and the ability to influence other people positively

Exercise: Listen to a sound
Now listen carefully to the ticking of a watch. When your mind wanders, bring it back to the sound. How long can you concentrate on that sound?
Exercises to practice at home-leaflet to be given

Lose yourself in a book
Read two or three pages of a book, giving them your full attention. Then test your concentration by stopping at the end of a page. How much do you remember of the story? Can you classify, group or compare the facts you have been reading about?

Contemplate nature
During the day, concentrate on the sky. Feel your mind expand as you reflect on its vast expanse. At night, concentrate on the moon or stars. By the sea, focus on waves. Or shift your gaze between objects near and far, such as a nearby tree and a distant mountain.

Focus on a flower
Sit comfortably with your eyes closed. Imagine a garden with many flowers. Gradually, bring your attention to a single flower. Visualise its colour and explore its other qualities, such as texture, shape, and scent. Concentrate on the flower's qualities for as long as possible.

Positive Thinking and Meditation – Part II

Practical approach to meditation

Ask participants if they had chance to practice any of the concentration exercises then discuss and highlight:

- Meditation is a state of relaxed awareness

- The more care and attention you give to your preparation for meditation, the more positive the results will be

- Get the atmosphere right for meditation:

  Place: It is best to separate one portion of a room to use for your practice. Keep it clean and tidy, and place a candle or spiritually uplifting picture there. Burning incense can also help to create a meditative mood.

  Time: The best times for meditation are at dawn and dusk. Alternatively, find a time when you are free from daily activities and your mind can be calm.

  Habit: Practise every day at the same time. As your subconscious mind gets accustomed to the regularity, you will find it easier to settle and focus.

  Sitting position: Sit on the floor to meditate, in position that you can maintain comfortably, keeping your spine and neck straight but not tense. A simple, crossed-legged pose makes a firm base. Sitting on the cushion helps the thighs to relax and bring brings knees closer to the ground.

  If you cannot sit on the floor easily, sit on a comfortable chair with your ankles crossed.

DRAFT

Tillin T, *et al. BMJ Open* 2019; 9:e030119. doi: 10.1136/bmjopen-2019-030119

Imperial College London

Breathing: Once you are sitting comfortably, relax your body as much as possible. Broaden your chest and lift your rib cage to encourage abdominal breathing. Then inhale and exhale rhythmically for about 3 seconds each, gradually slowing your breath down.

Making Positive Changes in Your Life

Topic to reflect: Think about one change you would like to make (if any) to make your lifestyle healthier.

Ask if anyone would like to share their idea then suggest the following changes to make within first two months of practising yoga.

- **Proper exercise:** Try to practise asanas regularly
- **Proper breathing:** Practice deep abdominal breathing
- **Proper relaxation:** Learn Corpse pose and try to relax for 15 minutes daily
- **Reduce negative dietary habits:** Cut down or eliminate meat and cut down on fried food
- **Reinforcing positive dietary habits:** Drink 4 to 5 glasses of water and eat one raw salad daily
- **Eradicating negative habits:** If you smoke replace it with abdominal breathing
- **Concentration exercises:** Practice listening and hearing what others are saying
- **Positive thinking:** Refrain from using abusive language and try to spend time with people who have a positive outlook on life
- **Meditation:** Sit silently for at least 20 minutes daily with the mind focused on breath
- **Study:** Read something of inspiration daily

DRAFT

Tillin T, *et al. BMJ Open* 2019; 9:e030119. doi: 10.1136/bmjopen-2019-030119

Imperial College London

Appendix 1

Policy for cardiac rehabilitation in Ealing

INTRODUCTION

Cardiac disease is the leading cause of death in United Kingdom and is the leading cause of hospitalisation for both men and women. Cardiac rehabilitation programmes are recognised as a way to enhance recovery following acute cardiac events and encourage behaviour aimed at the secondary prevention of coronary artery disease. The key elements of cardiac rehabilitation are contained in the definition produced by the Scottish Intercollegiate Guidelines Network (SIGN): Cardiac rehabilitation is the process by which patients with cardiac disease, in partnership with a multidisciplinary team of health professionals, are encouraged and supported to achieve and maintain optimal physical and psychological health.

Cardiac rehabilitation is defined by the World Health Organisation as:
".. the sum of activities required to influence favourably the underlying cause of the disease, as well as the best possible, physical, mental and social conditions, so that they (people) may, by their own efforts preserve or resume when lost, as normal place as possible in the community. Rehabilitation cannot be regarded as an isolated form or stage of therapy but must be integrated within secondary prevention services of which it forms only one facet".

The provision of skilled help, support and supervision that is tailored to individual patients can: a) help people understand their illness and its treatment; b) provide psychological and emotional support; c) improve people's success in making beneficial lifestyle changes; and d) help people make the transition back to a full and as normal life as possible. (NSF: cardiac rehabilitation, 2007)

The 2007 NICE guidelines on "secondary prevention for patients following a myocardial infarction" state that cardiac rehabilitation should be equally accessible and relevant to all patients after an MI, particularly people from groups that are less likely to access this service. These include people from black and minority ethnic groups, older people, people from lower socioeconomic groups, women, people from rural communities and people with mental and physical health co morbidities.

The British Association of Cardiac Rehabilitation (BACR) standards 2007 defined the core components of cardiac rehabilitation as lifestyle (physical activity and exercise, diet and weight management, smoking cessation), education, risk factor management, psychosocial, cardio protective drug therapy and implantable devices, and long-term management strategies (4).

Four phases of cardiac rehabilitation were defined by the BACR and endorsed by the National Service Framework (2007) for CHD in England and Wales and SIGN for Scotland (2002). Each phase represents a different component of the journey of care. Phase 1 is generally concerned with the in-patient episode with Phases 2-4 following the patient from early discharge to long-term maintenance.

According to the NSF goal, every hospital should ensure that:
more than 85% of people discharged from hospital with a primary diagnosis of acute myocardial infarction or after coronary revascularisation are offered cardiac rehabilitation and one year after discharge at least 50% of people are non-smokers, exercise regularly and have a BMI <30 kg/m^2; these should be demonstrated by clinical audit data no more than 12 months old. Trusts should agree, implement and audit a detailed plan and protocol for identifying, treating and following up their patients who may benefit from cardiac rehabilitation.

THE CARDIAC REHABILIATION PROGRAMME IN EALING HOSPITAL NHS TRUST

The aim of the comprehensive cardiac rehabilitation programme is to reduce the risk of subsequent cardiac problems and to promote the return to a full and normal life. The provision of a cardiac rehabilitation service for all eligible patients is clearly desirable for health and economic reasons.

Tillin T, *et al. BMJ Open* 2019; 9:e030119. doi: 10.1136/bmjopen-2019-030119

DRAFT

Imperial College London

Comprehensive help with lifestyle modification involving education and psychological input as well as exercise training can reduce mortality by 20-25% over 3 years. (Oldridge et al 1988; O'Connor et al 1989)

1. TARGET CLIENT GROUPS

Patients admitted with or who have undergone the following will be eligible for the programme.

- NSTEMI
- STEMI
- Acute Coronary Syndrome
- Revascularisation
- CABG
- Valve surgery
- Heart Failure

All the above patients admitted to Ealing Hospitals will be offered a choice as to where their cardiac rehabilitation will take place.

2. IDENTIFYING PATIENTS

- Patients are identified through CCU/ITU, cardiology and general medical wards, cardiology out patients, and from waiting lists for revascularisation procedures.
- Referrals are accepted from other acute trusts (using North West London Cardiac Rehabilitation referral form); Ealing Hospital cardiac catheter laboratory, as well as the community referrals from GP's, Practice Nurses, Community Specialist Clinics.

3. PHASE 1 (Before discharge from hospital)

Where possible the Cardiac Rehabilitation Specialist Nurse will visit the patient and his / her family during the hospital stay. The following will be carried out during this phase:
• assessment of physical, psychological and social needs for cardiac rehabilitation
• negotiation of a written individual plan for meeting these identified needs
• initial advice on lifestyle e.g. smoking cessation, physical activity (including sexual activity), diet, alcohol consumption, driving and employment
• review of prescription of effective medication and education about its use, benefits and harms
• involvement of family members and/or relevant informal carer(s)
• provision of information about cardiac support groups
• provision of locally relevant written information about cardiac rehabilitation

It is important to establish rapport and therapeutic relationship with every patient and involve family or/and carers from this early stage. This will increase the likelihood or patient's participation in consecutive phases of cardiac rehabilitation programme and reduce a risk of DNA incidences.

The “Edinburgh Heart Manual” for education, exercise and stress management components can be given to eligible patients at this stage. Social needs and preferences of patients will be identified and taken into account for a purpose of structuring of individually tailored cardiac rehabilitation programmes.

Guidelines for Phase 1 Cardiac Rehabilitation Service will be followed (see Appendix 1\)

BACR Guidelines for Secondary Prevention will be followed. They are:

- Risk factors of each patient should be identified and managed accordingly.
- All patients who smoke should be offered structured anti-smoking advice and, if necessary, specific

Tillin T, *et al. BMJ Open* 2019; 9:e030119. doi: 10.1136/bmjopen-2019-030119

Imperial College London

treatment.

- All patients after acute myocardial infarction or coronary revascularisation should be treated, if necessary, with lipid lowering therapy (diet control, statin therapy, lifestyle changes to include regular exercises), antiplatelet therapy (with aspirin, dipyridamole or clopidogrel), beta blockers, ACE inhibitors, other secondary prevention measures (better control of diabetes, hypertension, body mass index (BMI)).
- All patients with heart failure should be given advice on fluid balance (daily weights, fluid intake, diuretic dosages), salt restriction, avoidance of ethanol consumption and smoking, influenza vaccination, and considered for therapy with ACE inhibitors, beta blockers, spironolactone, angiotensin receptor blockers for prognostic benefits.
- Patients with other conditions should receive appropriate advice and treatment as secondary prevention of their specific cardiac conditions (e.g. avoidance of caffeine and treatment with beta blockers in patients with cardiac arrhythmia).

4. PHASE 2 (Early post-discharge period)

During early post discharge period, support to patients can be provided by home visiting where appropriate, telephone contact and by supervised use of the Heart Manual.
Patient will be sent an invite letter for the first outpatient appointment in cardiac rehabilitation clinic of Ealing Hospital within 2 -3 weeks after discharge from hospital. The time tables with dates of currently run educational sessions will be included with an invite letter.(see Appendix 2). Where possible, patients will be asked to have their blood tests done in GP practices prior to appointment with cardiac rehabilitation specialist nurse. Patients will also be asked to bring their medication list and any outpatient appointments' letters with them.
During the consultation in OPD clinic, the individual needs, expectations of cardiac rehabilitation programme and wishes will be explored. The suitability for exercise programme (a component of comprehensive cardiac rehabilitation programme) can also be assessed at this point.

Following will be carried out:

- Provision of general advice about the cardiac condition(s) and complications that the patient has, including risk factor management, medication (what they are for, adjustment of doses and potential adverse reactions), presentation of further events and what actions to take; symptom control advise
- Patients' misconceptions and undue fears or anxieties will be identified and addressed
- Patients will be advised on the stages towards resuming normal life (e.g. physical activity levels, sexual function, driving, flight, return to work, weight control, fluid balance, alcohol consumption).
- Advice will be provided to patients to address vocational, social, cultural, educational needs, and referral for occupational therapy assessment and management.
- Measurement of patient's body weight, calculation of body mass index (BMI) and central obesity (girth measurements)
- Review of fasting lipid profile +- advice/ appropriate referrals to GP/lipid clinic.
- Review of fasting blood glucose +- advice/ referral to diabetic specialist nurse/GP
- Dietary habit assessment and advice on healthy eating
- Blood Pressure will be measured and heart rate record
- If known to have diabetes a urine sample for microalbuminurea will be collected.
- Assessment of wounds and advice as necessary (post surgery patients)
- HADS +-quality of life (QoL) assessments will be carried out to estimate patients' health perceptions and to help detect patients with inappropriate levels of anxiety or depression, a small proportion of whom may need referral for specialist evaluation and treatment
- Advice how to stop smoking- for those who smoke+- referral to specialist services
- Review involvement with cardiac support groups
- Offer resuscitation training for family members
- Encouraging patients' immediate family members to engage in health improvement and lifestyle modification

"Client Feedback and Goal Planning Form" (see Appendix 3), as well as "Agreed Action Plan" (see Appendix 4) forms will be given to those who were not seen at Ealing Hospital during Phase 1. Patients will be encouraged to

Tillin T, *et al. BMJ Open* 2019; 9:e030119. doi: 10.1136/bmjopen-2019-030119

Imperial College London

participate in joint (with nurse specialist) care planning, goal setting, time-allocation for improvement and exploration of barriers to achievement of desirable results. This process is very important for achievement of lifestyle modification and behavioural change.
Patients will be encouraged to bring those forms to meetings with cardiac rehabilitation nurse, or/and to educational, or/and exercise programmes, so appropriate to the area of concern specialist will be able to answer the questions and give patient-tailored advice and recommendations.
Patients will be also given comprehensive information regarding diagnosis, procedures, practical advice and risk factor modification in written form. Patients will be issued with a wallet sized card that allows each patient to keep a record of his or her risk factors, including blood pressure, cholesterol and glucose, lifestyle modifications, dates of procedures and current medication.

Patients and their partners will be invited to enter an 6-12 week health promotion programme where patients will receive 1) on-going risk factor monitoring/ advice/support, 2) exercise sessions in a gym, led by cardiac physiologist or home-based exercise programme, 3) health education lectures (led by cardiac rehabilitation sister; pharmacist; dietician; clinical psychologist; cardiac physiologist), 4) relaxation sessions, 5) guidance and supervised use of “Edinburgh Heart Manual”.

Patients will be asked about their preferences on exercise programme, whether they would like to join exercise programme run in local Gym(s) or to have home based exercise programme (e.g. using “Edinburgh Heart Manual” as guideline and/or using exercise plan prescribed by cardiac physiologist and/or using pedometers, etc).
Patients will be given information on forthcoming dates/ topics/ venues of educational programme. Patients themselves and their family members or carers will be encouraged to come to educational sessions together.
For patients whose English is limited, the interpreter services will be provided where possible. Patients will be encouraged to bring their English speaking relatives for a consultation. The information about each individual condition/treatment/recommendations can be ordered for patient from British Heart Foundation in a form of audio tape/video-tape/pocket size leaflets, etc
Those patients who will not respond to invitation letter to attend an OPD clinic will be contacted via a telephone. Rehabilitation staff will try to address the issues which might impede patient's decision about participation in a programme using individual approach. However, If a patient will state clearly that he/ she does not wish to participate, then patient's GP will be informed and patient will be discharged from CR service.

4.1.Referrals:

- Weight management and diet advice: All patients regardless of their cardiac condition will be referred to cardiac specialist dietician for cardio-protective diet advice and weight management programme if appropriate.
- Diabetes: Newly diagnosed and those patients with diabetes who are not well controlled will be referred to the community diabetes team.
- Erectile Dysfunction: patients experiencing sexual problems can be referred to the ED clinic at GSTT.
- Psychological Problems: Rehabilitation staff will do their best to identify and address cardiac misconceptions in patients with CHD in order to reduce possibility of anxiety or/and depression. Hospital anxiety and Depression Scale (HADS) will be used. Screening will take place at discharge (where possible), 6-12 weeks post MI or following a decision on surgical intervention; can be repeated every 3 months if necessary. Psychological interventions of cardiac rehabilitation programme, such as stress management, relaxation, goal setting, taking part in group exercise and education can relieve anxiety and mild depression. Patients who score persistently above 11 on the HAD scale can be considered for referral to a clinical psychologist for assessment/ interventions.

- **Health Education Talks** (please see appendix 2 for the current health education presentations)

Discussions will be given by a health care professional with specialist knowledge of the subject. This is an information giving session to increase patients knowledge.

The group discussion allows patients to explore the information given and how best to apply it to themselves and their families. These sessions are intentionally informal and encourage patients to recognise their own risk factors and develop strategies for change. The following topics are to be covered by the workshops:

1. Drugs for heart disease and how they work (presented by pharmacist)

DRAFT

Imperial College London

2. Managing Stress (by clinical psychologist)
3. Eating for a healthy heart (by cardiac specialist dietician)
4. Exercise and the benefits for your heart (cardiac exercise physiologist)
5. Risk factors and making lifestyle changes (by cardiac rehabilitation sister)
6. The heart and how it works (by cardiac rehabilitation sister)

5. PHASE 3 (Four weeks after an acute cardiac event / 4-6 weeks post surgery)

Structured exercise as a therapeutic intervention is central to cardiac rehabilitation. Exercise training should form a core element of cardiac rehabilitation programmes (SIGN,2002).
At this stage, patients and their family members/carers should be aware of all the benefits of the physical exercise programme and should be committed to participate. Most patients will benefit from and will be encouraged to undertake at least low to moderate intensity exercise. However, patients with clinically unstable cardiac disease or limiting co-morbid illness will be excluded from exercise training. People whose potential to exercise is limited may have much to gain from the non-exercise components of cardiac rehabilitation.

5.1. Contraindications to Exercise

- Unstable Angina
- Unstable Ischemia
- Active pericarditis or myocarditis
- Hypertrophic obstructive cardiomyopathy
- SBP >180 mmHg or DBP >100 mmHg
- BP drop 20 mmHg during incremental exercise
- Resting/uncontrolled tachycardia >100
- Severe and symptomatic aortic stenosis
- Uncontrolled atrial or ventricular arrhythmias
- Severe pulmonary hypertension
- Heart failure that is not compensated
- Recent embolism
- Thrombophlebitis
- Unstable diabetes
- 30 AV block (without pacemaker)
- Febrile illness

5.2. Exercise sessions.

- The exercise sessions are held twice a week for 1 hour in St.Bernard's and Southall sport centres each week and patients are encouraged to attend between 8-12 sessions.
- Those patients who wish to participate in the exercise programme need to sign a consent form
- All patients need to attend an initial screening appointment and perform a sub maximal functional capacity test prior to attending the classes.
- Patients will be risk stratified into low, medium and high risk categories as defined by the American Association of Cardiovascular & Pulmonary Rehabilitation (AACVPR) as recommended by American College of Sports Medicine**.** (Appendix 5);"low risk" patients will be enrolled to attend a community sport centre, "moderate/ high risk" patients will be invited to participate in an exercise programme, based in a gym which in located in close proximity to Ealing Hospital.
- Patients, who will not want to attend formal taught sessions will be offered home-based exercise plan.

Assessment before Exercise Classes:

- Prior to participation in exercise training patients will undertake a submaximal functional capacity test (e.g. the 6 minutes walk test or shuttle walk test). This will usually be carried out by cardiac physiologist during a separate appointment.
- Prior to submaximal testing of functional capacity a pre-screening checklist will be completed to ensure suitability, an end point of 80% HR max will be determined (adjusted as appropriate for high risk patients) and a rating of perceived exertion of 15, using the Borg Scale category ratio 6-20 scale (Appendix 4). Prior to participation in the exercise test the patient will be familiarized to the Borg scale as below

DRAFT

Imperial College London

- Target heart rates for the exercise classes will be set to between 60-75% of the maximal heart rate minus 30 if on Beta Blockers. This range can be adjusted based on risk stratification. The range will be written in patient's exercise plan.

Before the Class Begins

- Brief discussion with patients about their progress, home exercises, changes, concerns
- BP and pre-exercise heart rate will be recorded
- Blood glucose levels checked for diabetic patients
- Equipment set up in advance
- Those patients who will complain of feeling generally unwell or become symptomatic or clinically unstable can be excluded from a session for a day. Depending on a condition, the symptoms will be treated with existing medications; patient will be either accompanied to A&E (if severely unwell) or referred to GP.

The Exercise Components.

All sessions should include:

Warm-Up (15 minutes minimum): The warm-up period will include graduated low intensity aerobic exercise and short dynamic stretches to increase myocardial blood supply, soft tissue flexibility and mobilize joints.
Circuit (20-30 minutes): All patients will participate in a progressive exercise training programme, which is modified to meet individual need.
Cool Down (10 minutes): This will include graduated low intensity exercise and muscle stretching. Once complete HR will be rechecked and recorded (aim to be within 10 beats of pre-exercise rate)
Relaxation (15 minutes minimum): Following the exercise class, patients should be supervised for a 15 minute period.

Health and Safety Requirements.

- Each patient will be risk stratified as described above.
- Exercise will be delivered by experienced staff with training in exercise physiology and prescription and an understanding of the specific needs of cardiac patients in relation to exercise.
- 3 members of staff where possible - minimum ratio 1:5. This includes visitors. However priority for exercise is given to patients and if the numbers exceed the safety requirements, visitors will not be able to join in on that occasion.
- Cardiac physiologist to be trained BLS skills (minimum), cardiac rehabilitation nurse-to be present at each session and to be trained at ILS level (minimum)
- Resuscitation equipment available in the gym for the duration of the class.
- All visitors who wish to join in the exercise class need to complete a ParQ
- Venue must be suitable i.e. adequate space, temperature (65-72F,18-23C),
- Drinking water should be available.
- Immediate access to a telephone.
- Annual environmental risk assessment

Monitoring patients

- Heart rate monitors to be worn during a patient’s first session.
- Heart rates recorded at the beginning of each class, during the class and after the cool down.
- Borg scale of perceived exertion will be recorded during exercise.

Patients with diabetes

- Record blood glucose level before the start of the exercise
- Avoid exercise if glucose is over 16mmol/L.
- Avoid exercise if glucose is under 6 and no snack is available prior to exercise
- If glucose >13 and <16 then do warm up and retest – level should fall. If remains >13 and rising should not continue exercise until their status has been stabilised.
- Those taking insulin should avoid injecting into subcutaneous tissue of thigh i.e. avoid sites near to exercising muscle groups.
- Avoid exercise during peak insulin times.

DRAFT

Tillin T, *et al. BMJ Open* 2019; 9:e030119. doi: 10.1136/bmjopen-2019-030119

Medical Emergencies

- Nurse to stay with patient
- Exercise specialist to ensure safety of other patients
- Third person to call for help (999 and/or cardiologist)

DNA Policy

Patients will be informed about current DNA policy and their obligation to notify a cardiac rehabilitation nurse or cardiac physiologist if they have to miss a session. If a patient does not attend two consecutive classes, contact will be initiated by a member of the Cardiac Rehabilitation team and if no response is received then the patient is discharged from the programme and a letter sent to patient's GP as well as patient.

End of Programme.

On completion of the programme patient is given a Certificate stating patients achievements (see Appendix 6) and a re-screening appointment is made in 2-4 weeks. Patient's GP will receive a letter from Cardiac Rehabilitation Team with all relevant information.

5.3. Home-Based Programmes

Those patients who would prefer a home programme or are unable to attend the group sessions will be assessed by the cardiac physiologist and given a suitable physical activity programme. Progress will be monitored regularly and risk factor management will continue as required. This may involve the patient attending regular appointments with the cardiac nurse for blood pressure/heart rate/ blood results monitoring/relevant support and advice. The patient is offered the opportunity to attend the health education talks where possible.

6. RE-SCREENING OF PATIENTS

On completion of the health promotion sessions all patients and their families are invited back to the Cardiac Rehabilitation OPD clinic where they will be reassessed as follows:

- Cardiac Risks will be assessed again and progress recorded
- Blood pressure, heart rate, lipids and glucose levels are repeated and recorded
- Those with diabetes will have their HbA1c checked
- Current medications therapy is reviewed
- HAD and QOL is repeated
- 6 MWT or Shuttle walk test is repeated Diet is reassessed and long term recommendations made
- BMI and girth measurement is checked again and recorded

Patients with stable coronary disease will be encouraged to continue regular moderate intensity aerobic exercises. The relevant information about the exercises, stretching techniques, relaxation exercises, and all available sport/leisure centres in the area will be given to patients on discharge. Information about local yoga/dancing/swimming/golf classes, etc. will be available on request. Individual approach will apply, hence if someone will prefer to carry on home based exercises he/she will be supported in their decision. Others, who prefer formal class based cardiac exercise programmes can be referred to the Phase 4 exercise sessions held in St.Bernard and Southall sport centres. The exercise sessions are lead by BACR trained exercise physiologists.

7. PHASE 4 (Long-term maintenance of changed behaviour)

Long term follow-up in primary care will be arranged.
Involvement with local cardiac support groups or groups of interest (e.g. gardening, cooking, walking, cycling, etc.) will be offered.
Referral to specialist cardiac, behavioural (e.g. exercise, smoking cessation) or psychological services will be made, if clinically indicated.

8. ANNUAL REVIEW

DRAFT

Imperial College London

All patients are invited to attend a follow-up appointment one year after completion of the programme. At this appointment fasting lipids, glucose, and, if appropriate, and HbA1C are measured. Blood pressure is checked twice and anthropometry is recorded. A physical activity and brief dietary assessment are carried out. A summary is sent to the GP and patient with further recommendations if appropriate.

9. INTERGRATING CARE BETWEEN SECONDARY & PRIMARY CARE.

A seamless transition between hospital provision of cardiac rehabilitation and the continuing support provided by primary care practitioners requires good communication between all involved in the care of patients with CHD. The primary care team, with detailed knowledge of an individual's social and medical background, includes professionals who are likely to be aware of the implications of CHD for both the individual and their family. Accurate information shared between the various members of multidisciplinary teams across both primary and secondary care will enable the best possible care to be given to the patient.

10. AUDIT & EVALUATION (TO AGREE ON EVALUATION OF WORK)

The Cardiac Rehabilitation service will carry out clinical audit using routinely collected data. Long term goals can be monitored by observing changes over time in incidence and mortality from CHD.

Data will be collected onto the CR database and will also be exported to the national database annually as required for a purpose of NACR. On completion of the programme, patients will be asked to fill in a satisfaction questionnaire.

Standards that we need to follow:

The service should be referred to in the HImP and reflected in long term service agreements.

A clear description of the district cardiac rehabilitation programme should be available to the public, to service providers and to commissioners and should be cited in the HImP. This description should include details of:

• the patients to be offered cardiac rehabilitation
• staffing (including details of the skills and training required)
• the location and timetable of service provision
• audit criteria
• investment and resources.

Whatever the detail of local rehabilitation services, records should be kept so that the service can be audited against nationally recommended guidelines. This should include information about ethnicity so that it is possible to monitor equity of access. Audit will be easier to undertake if data is stored electronically in a way that allows ready analysis.
National Service Framework – Coronary Heart Disease

Clinical audit

Clinical audit – the systematic assessment of the quality of care – is an essential component of modern, high quality health care. It will also be an essential component of effective clinical governance embracing all health professionals.
Trusts should work with their local PCTs and their constituent practices to undertake clinical audit that allows them to review annually the items listed in **bold** below. They may also wish to review the other items when it becomes possible to collect these data.

1) number and % of patients discharged from hospital after coronary revascularisation OR with a primary diagnosis of AMI with documentation of arrangements for cardiac rehabilitation in discharge communication to GP (by Trust and PCG/PCT and by sex,

DRAFT

Tillin T, *et al. BMJ Open* 2019; 9:e030119. doi: 10.1136/bmjopen-2019-030119

Imperial College London

age 35-74iii years, and ethnic group)

2) number and % of patients discharged from hospital with a primary diagnosis of CHD recruited to a cardiac rehabilitation programme by Trust and PCG/PCT and by sex, age 35-74iii years, and ethnic group

3) total number and % of those recruited to cardiac rehabilitation who have an individualised plan for rehabilitation and secondary prevention before discharge from hospital
4) total number and % of those recruited to cardiac rehabilitation who, one year after discharge, report:
• regular physical activity of at least 30 minutes duration on average 5 times a week
• not smoking
• BMI < 30 kg/m2.

(NB. PCTs and rehabilitation services may wish to collaborate in the collection, analysis and interpretation of their audit data to avoid duplication of effort and to gain a more complete picture of the quality of rehabilitation and secondary prevention services.)

This Policy will be reviewed and updated if necessary on annual bases.

REFERENCES

1. Scottish Intercollegiate Guidelines Network (2002) Cardiac Rehabilitation. A national clinical guideline. SIGN guideline 57. http://tinyurl.com/27g33c

2. National Institute for Health and Clinical Excellence (2007) MI: secondary prevention. Clinical Guideline 48. May. http://tinyurl.com/38tom3

3. British Association of Cardiac Rehabilitation (2007) Standards and Core Components for Cardiac Rehabilitation. http://tinyurl.com/3ydagw

4. Department of Health (2000) Coronary Heart Disease: National Service Frameworks. HMSO: London

5. World Health Organisation (1993) Needs and action priorities in cardiac rehabilitation and secondary prevention in patients with CHD. Geneva: WHO regional office for Europe

6. American College of Sports Medicine (1991) Guidelines for exercise testing and prescription, 4th edn. Lea and Febinger, Philadelphia

DRAFT

Tillin T, *et al. BMJ Open* 2019; 9:e030119. doi: 10.1136/bmjopen-2019-030119

Imperial College London

Appendix 2

Ealing Cardiac Rehabilitation Health Education Talks

Drugs for heart disease and how they work

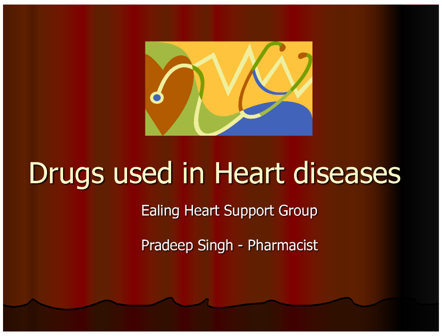

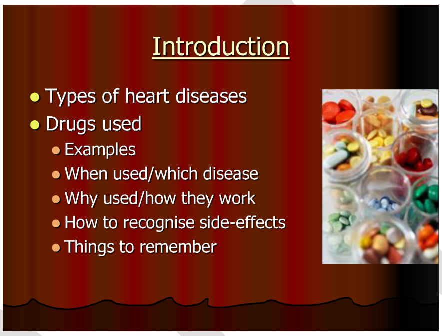

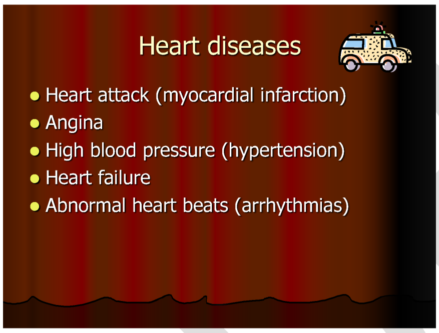

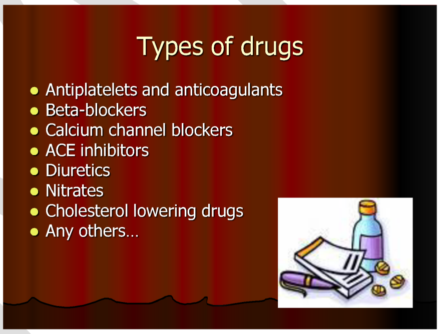

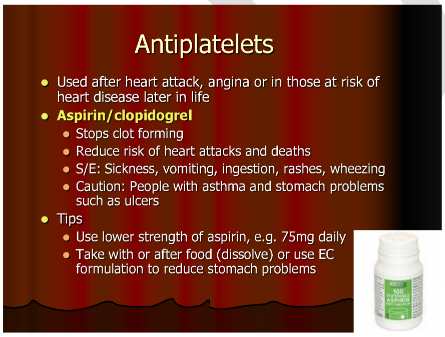

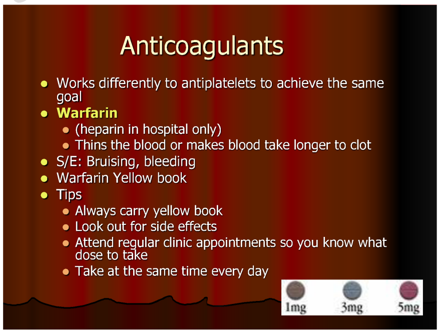

Imperial College London

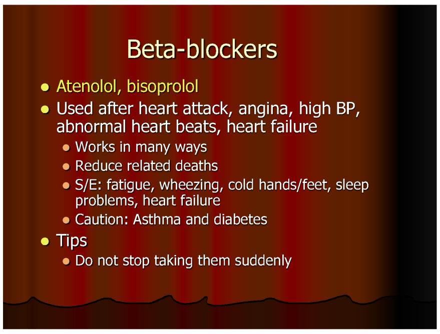

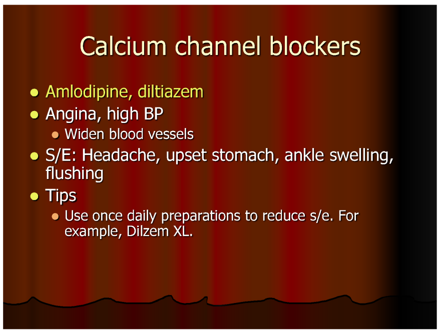

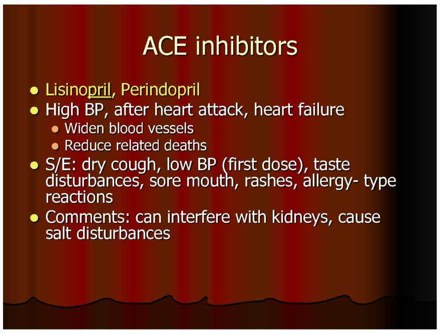

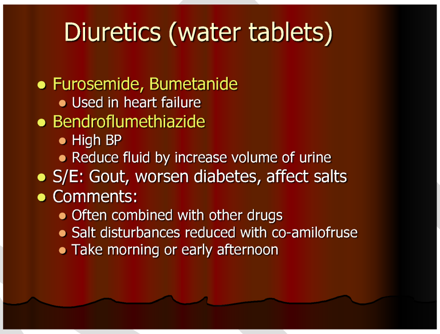

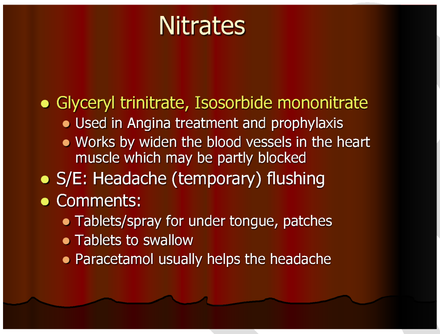

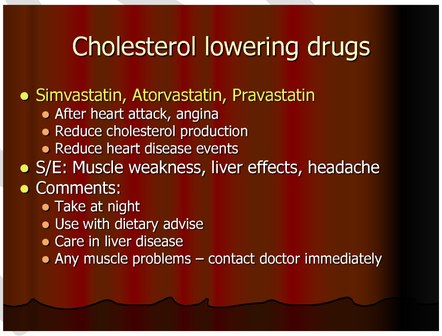

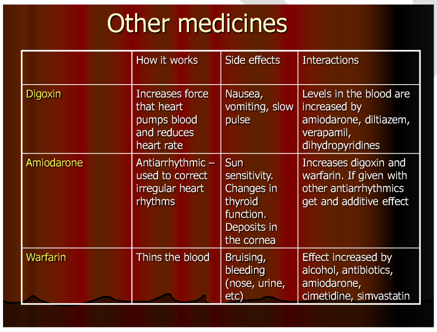

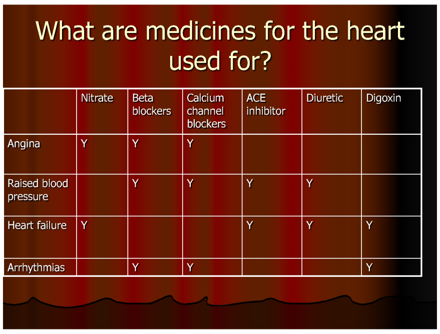

DRAFT

Tillin T, *et al. BMJ Open* 2019; 9:e030119. doi: 10.1136/bmjopen-2019-030119

Imperial College London

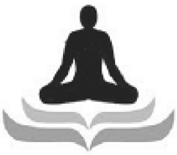

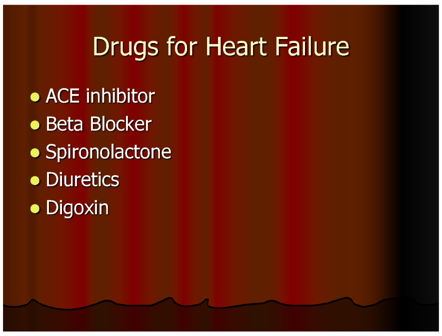

Managing Stress

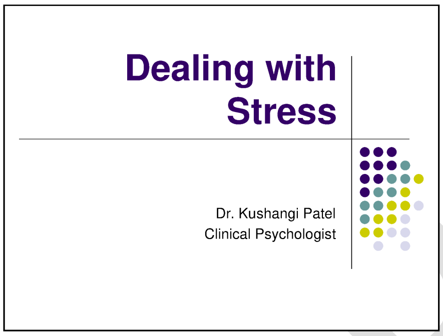

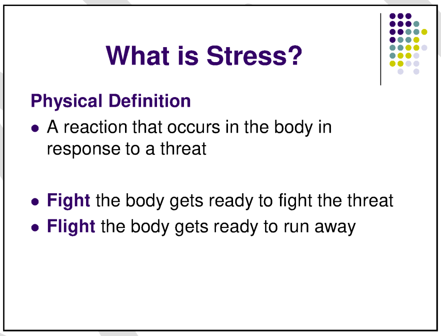

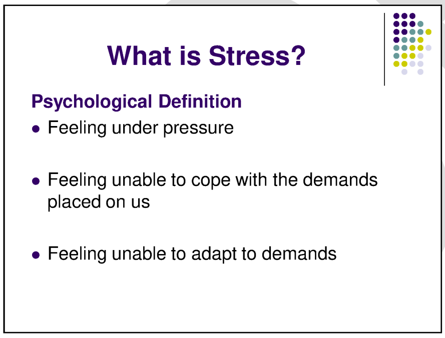

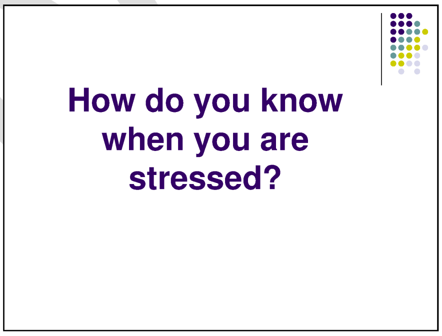

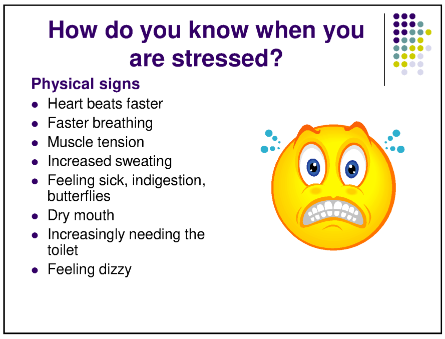

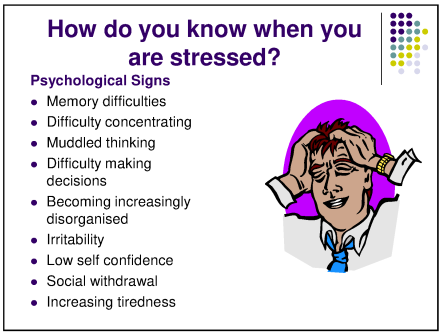

DRAFT

Tillin T, *et al. BMJ Open* 2019; 9:e030119. doi: 10.1136/bmjopen-2019-030119

Imperial College London

Purpose of stress

- Stress does have a purpose it's not all bad
- To protect ourselves from threat
- A small amount of stress helps increase our performance at things
- Stress can drive us to do things in life

What makes you stressed?

Environment

- Work, family, too many social demands, driving, money, not enough opportunity for enjoyment

Thoughts

- I can't cope! I haven't got time! If I don't get this done….. will happen!
- These are e.g's of negative thoughts and predicting worse case scenarios.

What causes stress in your life?

What makes you stressed

Emergency

- Threat to life/ safety
- Events that pose an immediate threat to us cause stress levels to increase sharply and then fall quickly

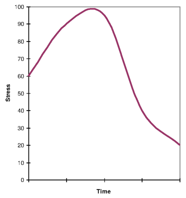

What makes you stressed

Daily Hassles

- Such as shopping, organising holidays/social events, keeping on top of daily tasks at home and/or work.
- These frequently affect our stress levels, increasing it and decreasing it as hassles are encountered and then dealt with.
- Watch out for daily hassles – these lead to a gradual but **regular** build up of stress.

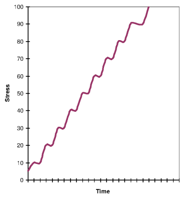

How stress causes health problems

- Stress causes heart rate and blood pressure to increase and hormones are released to enable us to fight or flight.
- If high and/or frequent stress is experienced and these hormones are not used up by physical activity, the increase in heart rate and blood pressure damages arteries.
- The body heals this damage but as a result the artery walls become thicker and scarred.
- This can affect the supply of blood and oxygen to the heart.

How health problems cause stress

- Health problems can affect all areas of life.
- Physical – feeling more tired, less able to do the things accomplished before the health problems started.
- Financial - time off work or a need to leave work altogether may have financial implications.
- Interests – a persons ability to engage in things they enjoy may be reduced temporarily or certain hobbies may have to be adapted.

How health problems cause stress

- Identity – we all have views of ourselves. E.g. independent, responsible, fun, healthy. A health problem may affect this view if we feel we are no longer able to "be" these things.
- All these aspects can lead to feelings of frustration, anger and sadness.
- This can all increase stress levels
- Talk to other people about these feelings and frustrations or seek help from professionals, GP, Cardiac Rehab Nurse.

DRAFT

Tillin T, *et al. BMJ Open* 2019; 9:e030119. doi: 10.1136/bmjopen-2019-030119

Imperial College London

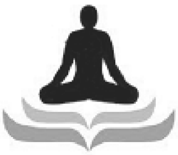

Health problems and stress

- Heart problems can knock self confidence so it is important to do things at a comfortable pace and build up gradually.
- Work to balance doing things at YOUR pace. Doing nothing and doing too much are both unhelpful.
- Take advice from GP's, cardiac team on how much you can do and when.
- Watch out for negative thoughts as they can lead you into a vicious cycle of inactivity and low mood which all can increase your stress levels.

Vicious cycle of negative thoughts

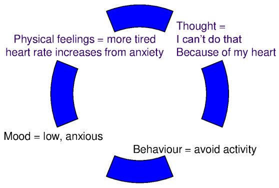

What are the consequences of stress?

Consequences of stress

- Health problems
- Difficulty in relationships
- Increase in sick leave
- Being unable to do things you need to do or enjoy
- Feeling upset, angry, frustrated, out of control

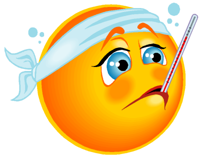

Exercise

- In pairs talk about how you have coped with stress.
- What sort of things have you done in response to stress?
- What has worked?
- What has not worked so well?

Coping with stress

Helpful ways

- Exercise
- Talk to someone
- Look at my thinking
- Prioritise tasks
- Share the load
- Relaxation
- Get guidance from other people or professionals involved in your care

Unhelpful Ways

- Increase alcohol/other drugs
- Work longer hours/ harder to finish tasks
- Shout
- Be on my own
- Ignore it

Coping with stress

Notice your early warning signs – these may be physical signs and/or psychological signs
Take action

- **Calm your body by** –
- Relaxation
- Regular physical activity helps keep overall stress levels low
- Eat healthily
- Do something you enjoy
- Rest if you're tired
- Avoid increasing alcohol, smoking or caffeine to cope

Coping with stress

- **Calm your mind by** –
- Relaxation
- Looking at your thoughts:
  Are you focusing on the negative?
  Are you predicting the worse case scenario without any evidence for it?
- Plan and prioritise
- Think about the long-term – is saying no now going to help you later on

It may be difficult to do all the techniques so find out what works for you!

DRAFT

Tillin T, *et al. BMJ Open* 2019; 9:e030119. doi: 10.1136/bmjopen-2019-030119

Eating for a healthy heart

This is an interactive session delivered by a cardiac specialist dietician – no presentation available.

DRAFT

Tillin T, *et al. BMJ Open* 2019; 9:e030119. doi: 10.1136/bmjopen-2019-030119

Imperial College London

DRAFT

Exercise and the benefits for your heart

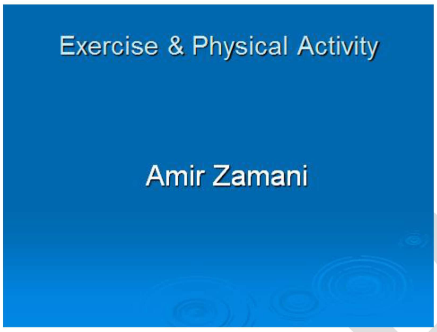

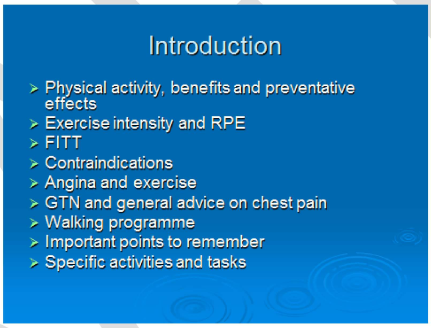

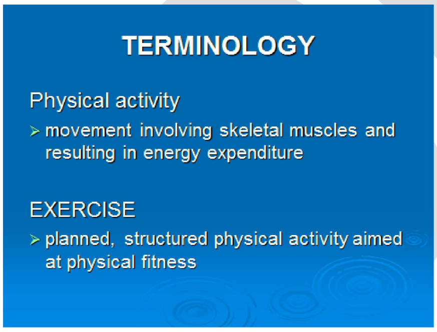

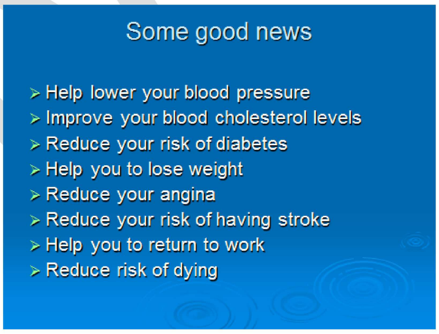

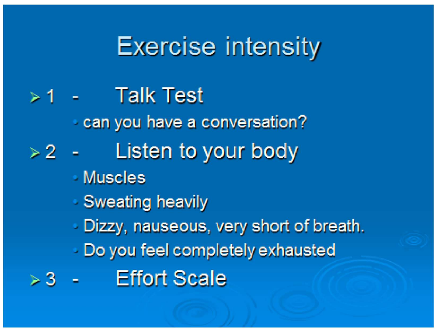

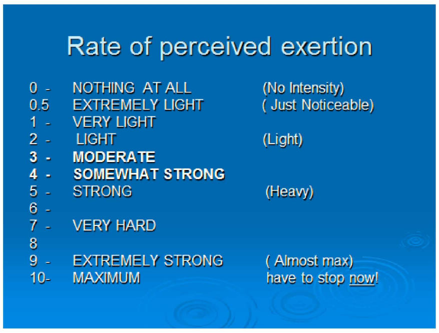

Imperial College London

FITT

- Frequency — Most days
- Intensity — Moderate
- Time — 30-40 minutes
- Type — Aerobic

STOP if you experiencing any:

- Undue shortness of breath
- Chest pain/discomfort (or pain in your neck/jaw/arm)
- Nausea/headaches/dizziness
- Inappropriate tiredness
- Persistent palpitations
- Feeling unwell

DRAFT

Angina and exercise

- Angina is often described as a tightness, heaviness or dull sensation in the chest
- It is usually brought on by exertion
- This is the way your heart saying that it is not getting enough oxygen
- It is particularly important to let your GP know if you are getting angina for the first time

What should I do if I get angina?

- The first thing that you need to do is STOP what you are doing and rest
- If you are given GTN spray or tablets it is important to use this medications

Remember the following

Angina pain/discomfort

Rest
1-2 GTN Spray/tablets under the tongue

If the pain is not relieved after 5 minutes
1-2 GTN Spray/tablets under the tongue

If the pain is not relieved after 5 minutes
1-2 GTN Spray/tablets under the tongue

If the pain is nor relieved after 5 minutes
Call 999 for an ambulance

General advice on chest pain

- If you have GTN spray carry it with you at all time
- If you have access to mobile phone, it may be a good idea to carry with you
- If you know of a activity that you know bring on angina, you can take your GTN before the commencing that activity
- Seated while you take your GTN
- Do not stop taking your GTN because of your headache
- Do not be afraid of using your GTN spray

How do I do Warm-Up?

Should be low level/
Nice and easy

10-15 minutes

Pulse raising activity and stretching

Warming Up and Cooling Down

WHY WARM UP?

Prepare muscles for
activity - ↓ injury

Prepare heart for activity
- ↓ angina
- ↓ disturbances in heart rhythm

Imperial College London

What sort of activity

- Aerobic, most beneficial activity for your heart
- Resistance or strength training

Walking programme

Stage of recovery	Length of walk (in minute)
(Approx. week 1)	5 Minutes: several times per day Strolling/leisurely pace
(approx. week 2)	10 minutes: twice a day, Leisurely pace
(approx. week 3)	15 minutes: daily, Leisurely/moderate pace
(approx. week 4)	20 minutes: daily, moderate pace
(approx. week 5)	25 minutes: daily, brisk pace
(approx. week 6)	30 minutes: daily brisk pace
Target	30-45 minutes: daily brisk walk

Cool Down

WHY COOL DOWN?

↓ Fainting and dizziness

↓ Disturbances to your heart

↓ Muscle soreness

How do I cool Down?

Goal is to return body to its resting state

Gradually slow down the activity you are doing and stretch

10 minutes

Sensible Precautions

- Do not exercise if you feel unwell
- Do not exercise on a full or empty stomach
  - Light meal/snack 1½ - 2 hours before
- Do not exercise in extreme temperatures
- Wear suitable clothing
- Take your medications
- Good days and Bad days
- Enjoy!

Have an active, healthy, happy life!

DRAFT

Imperial College London

Risk factors and making lifestyle changes

Risk Factors & Making Changes

Olivia Molloy
Cardiac Rehabilitation Sister

Modifiable and Non Modifiable Risk Factors

• Modifiable	• Non Modifiable
1. Smoking<br>2. High Blood Pressure<br>3. High Cholesterol<br>4. Physical Inactivity<br>5. Being Overweight<br>6. Diabetes<br>7. Alcohol consumption<br>8. Impaired glucose regulation	1. Family History of Heart Disease<br>2. Age<br>3. Ethnic Background

Smoking

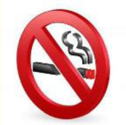

FACT

- Smoking is one of the major causes of cardiovascular disease. People who smoke are twice as likely to have a heart attack as to people who have never smoked

Smoking

- Smoking damages the lining of the arteries leading to build up of Atheroma.
- Carbon Monoxide in cigarette smoke reduces the amount of oxygen that the blood can carry to the heart and around the body
- Nicotine stimulates adrenaline which increases heart rate and raises blood pressure = harder work load for your heart
- Second Hand Smoking…..

Blood Pressure

- Blood Pressure represents the pressure of the blood in your arteries. We need a certain amount of pressure in our body in order to keep our blood (circulation) flowing
- High Blood Pressure is also known as **"Hypertension"**

This is when your blood pressure is higher than the recommended level.

Blood Pressure

- What does Systolic and Diastolic Pressure Mean?
- Readings:
  - 140/80mmhg = Non Diabetics
    - Diabetics = 130/80mmhg

Maximum

Imperial College London

Blood Pressure

- Why is High Blood Pressure dangerous

Remember

- High Blood Pressure does not make you feel Sick.

Cholesterol

- Cholesterol is a fatty substance which is found in the blood. Too much cholesterol is a contributing factor for Heart Disease.
- LDL
- HDL
- Total Cholesterol
- Triglycerides

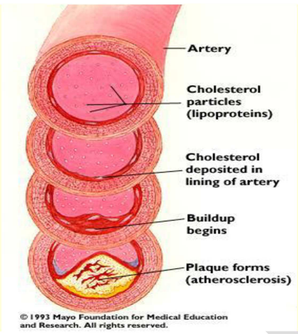

Excercise

- In the UK people who are not physically active are twice as likely to have a heart attack
- In the UK; 7 out of 10 people do not do physical activity to benefit their health
- What can you do?

Keeping a Healthy Weight & Shape

- Being overweight can increase your risk of developing Cardiovascular disease.
- Keeping close to a healthy weight will help you control your blood pressure and reduce the workload that you hear has to do.

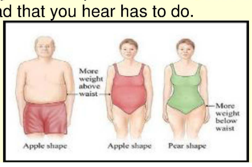

Family History

- If you have a family history of CVD your own risk of developing the condition is increased.
- A family history means if your

father or brother >55

or

mother or sister >65

- Non Modifiable risk factor

Eating Healthy For Your Heart

- Eat plenty of fruit and veg
- Choose healthier fats
- Eat oily fish regularly
- Reduce the amount of salt you eat

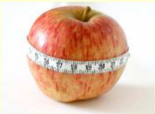

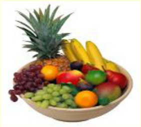

Alcohol

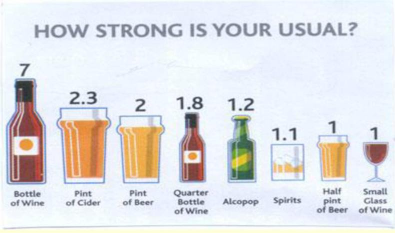

DRAFT

Imperial College London

Stress

- Stress is not a direct risk factor for CVD but it is possible that stress may contribute to it, or perhaps bring on some symptons.
- The way you deal with stress can encourage unhealthy behaviours e.g. smoking unhealthy eating , alcohol etc

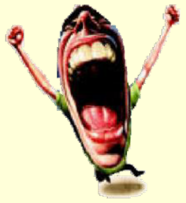

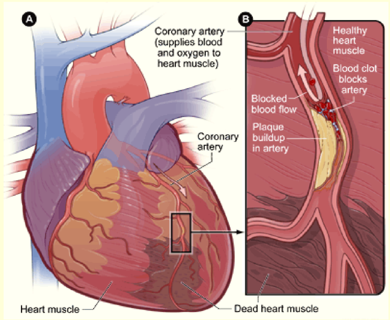

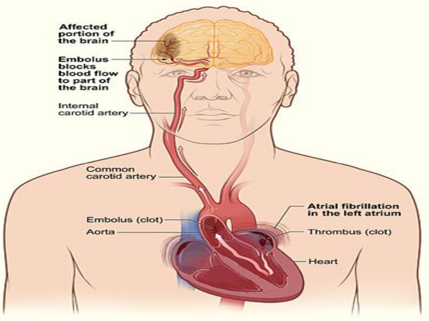

DRAFT

Imperial College London

The heart and how it works

The Heart & How it works

Olivia Molloy
Cardiac Rehabilitation Nurse Specialist

The Human Heart

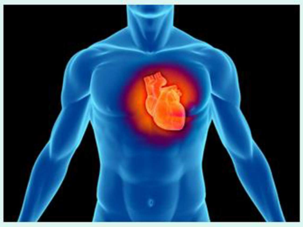

The Heart

- The heart is a fist sized organ which lies within the chest behind the sternum (breast bone). The heart sits on the diaphragm, the main muscle of breathing, which is found beneath the lungs. The heart is considered to have two 'sides' – the right side and the left side.

  The heart has four chambers – an atria and ventricle on each side.

- The atria are both supplied by large blood vessels that bring blood to the heart (see below for more details).

- Atria have special valves that open into the ventricles. The ventricles also have valves but in this case they open into blood vessels. The walls of the heart chambers are made mainly of special heart muscle. The different sections of the heart have to contract (squeeze) in the correct order for the heart to pump blood efficiently with each heartbeat

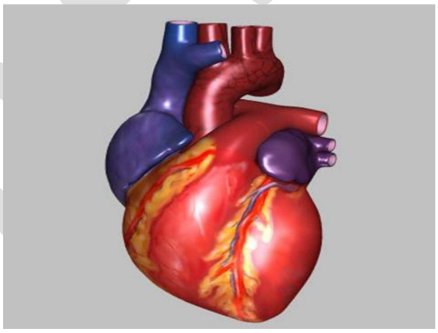

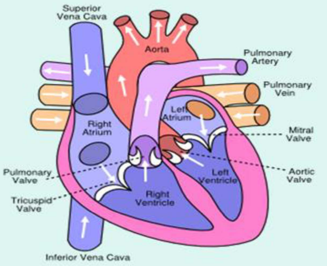

The Function of the Heart

- The heart is a muscular pump that pushes blood through blood vessels around the body.

- Essential to life, the heart beats continuously, pumping the equivalent of more than 14,000 litres of blood every day.

  Blood vessels form the living system of tubes that carry blood both to and from the heart.

- All cells in the body need oxygen and the vital nutrients found in blood. Without oxygen and these nutrients, the cells will die.

Imperial College London

The Heart Cont…

- The heart helps to provide oxygen and nutrients to the body's tissues and organs by ensuring a rich supply of blood.
- Not only do blood vessels carry oxygen and nutrients, but they also transport carbon dioxide and waste products away from our cells.
- Carbon dioxide is passed out of the body by the lungs, and most of the other waste products are disposed of by the kidneys.

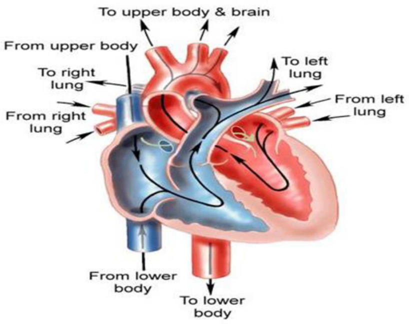

DRAFT

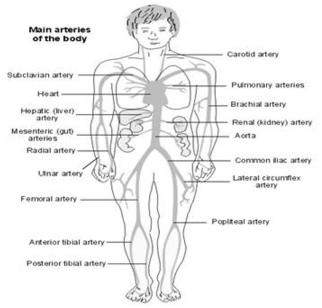

The Blood Supply to the Heart

- Like any other muscle, the heart muscle needs a good blood supply. The coronary arteries take blood to the heart muscle. These are the first arteries to branch off the aorta - the large artery that takes blood to the body from the left ventricle.

  The right coronary artery mainly supplies the muscle of the right ventricle.

  The left coronary artery quickly splits into two and supplies the rest of the heart muscle.

  The main coronary arteries divide into many smaller branches to supply all the heart

The coronary arteries of the heart

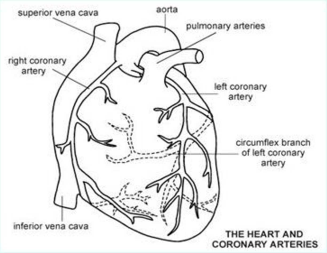

Angioplasty

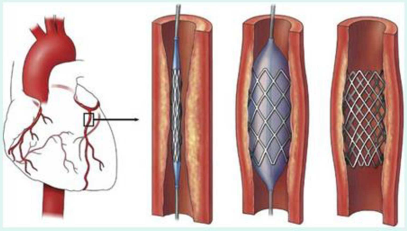

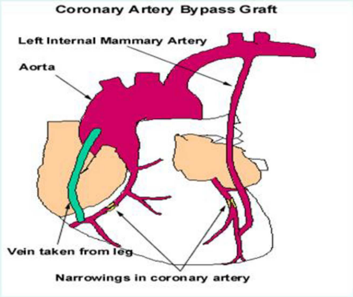

The Heart Valves

The heart also contains four valves.

- Their role is to ensure the blood flows in a forward direction and prevents a backward flow during any part of the pump action (or cardiac cycle).
- An atrioventricular valve sits on both the left and right sides of the heart between each atrium and ventricle.
- These are the tricuspid valve (right side) and the mitral valve (left side).
- The two remaining valves sit on the outflow tract of the left and right ventricles.

Imperial College London

Valves Cont….

- The pulmonary valve is between the right ventricle and the pulmonary artery, which takes deoxygenated blood to the lungs.
- The aortic valve sits between the left ventricle and the aorta, which takes oxygenated blood to the body's tissues.
- These latter two valves are semilunar; they contain three cusps which close to prevent the backward flow of blood from the outflow vessels during the diastolic (filling) phase of the cardiac cycle. The left side of the heart is inevitably under a much higher pressure than the right side, which delivers blood to the lungs only.

Heart Valves

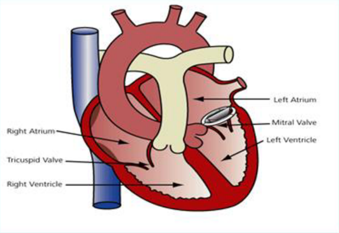

Chest Pain

- Stop what your doing
- Sit Down and rest
- If you have GTN spray or tablets, use the spray and take your tablets as instructed by your doctor or cardiac rehab nurse

Chest Pain Continued

- If you don't have GTN CALL 999 if pain does not go away.
- Aspirin , if you are not allergic to aspirin chew 300mgs until the ambulance arrives
- If the pain, discomfort or chest tightness continues especially if its gone on within 15 minutes
- DONT WAIT CALL 999 RIGHT AWAY

CALL THE AMBULANCE AND STAY RESTING

Basic Life Support

- Show DVD Recording here

Blood Pressure / Pulse

- Non Diabetic Patients — 140/80 mmhg
- Diabetic Patients — 130/80 mmhg
-
- Pulse for all — •60 – 100 bpm

Diabetics

- All Diabetics Blood Sugar should be less than

6

Imperial College London

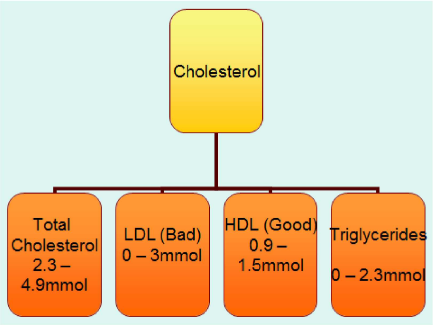

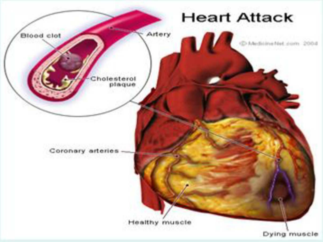

Cont….

- Arterioles are the smallest arteries in the body. They deliver blood to capillaries. Arterioles are also capable of constricting or dilating and by doing this they control how much blood enters the capillaries.

  Capillaries are tiny vessels that connect arterioles to venules. They have very thin walls which allow nutrients from the blood to pass into the body tissues. Waste products from body tissues can also pass into the capillaries. For this reason capillaries are known as exchange vessels.

  Groups of capillaries within a tissue reunite to form small veins called venules. Venules collect blood from capillaries and drain into veins.

  Veins are the blood vessels that carry blood back to the heart. They may contain valves which stop blood flowing away from the heart.

Cont….

- The right side of the heart receives deoxygenated blood (lacking oxygen) from the body. After passing through the right atrium and right ventricle this blood is pumped to the lungs. Here blood picks up oxygen and loses another gas called carbon dioxide. Once through the lungs, the blood flows back to the left atrium. It then passes into the left ventricle and gets pumped into the aorta, the main artery supplying the body. Oxygenated blood is then carried though blood vessels to all the body's tissues. Here oxygen and other nutrients pass into the cells where they are used to perform the body's essential functions.

  A blood vessels main function is to transport blood around the body. Blood vessels also play a role in controlling your blood pressure.

  Blood vessels are found throughout the body. There are five main types of blood vessels: arteries, arterioles, capillaries, venules and veins.

  Arteries carry blood away from the heart to other organs. They can vary in size. The largest arteries have special elastic fibres in their walls. This helps to complement the work of the heart, by squeezing blood along when heart muscle relaxes. Arteries also respond to signals from our nervous system, either constricting (tightening) or dilating (relaxing).

Blood Flow Through the Body

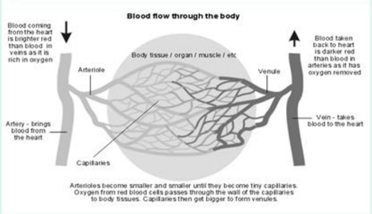

DRAFT

Imperial College
London

References

1. Sivananda: Yoga Teachers' Training Manual

2. Sivananda Yoga Vedanta Centre: Yoga-Your Home Practice Companion

3. Sivananda Yoga Vedanta centre: The Yoga Cookbook:Vegetarian Food for Body and Mind

4. H.David Coulter: Anatomy of Hatha Yoga

5. Swami Vishnu-Devananda: The Complete Illustrated Book of Yoga

6. www.yoga.about.com

7. www.innerhealthstudio.com

DRAFT
