# Supplementary material for: Yoga and Cardiovascular Health Trial (YACHT): a UK-based randomised mechanistic study of a yoga intervention plus usual care versus usual care alone following an acute coronary event
Source: BMJ Open. 2019 Nov 3;9(11):e030119. doi: 10.1136/bmjopen-2019-030119 (PMC6858127; doi:10.1136/bmjopen-2019-030119)
Supplement: Supplementary data [file bmjopen-2019-030119supp002.pdf]

SUPPLEMENTAL Table S1a. Characteristics at start of study (pre intervention) of those who completed/did not complete 3month follow-up: Usual care group

Means95%CI), number(%) unless otherwise stated	Usual Care, completed	Usual Care, dropped out
	N=35	N=5
Ethnicity: South Asian	22(63%)	4(80%)
Sex: Male	22(63%)	5(100%)
Age: years	57.2(54.0, 59.8)	54.5(38.3,73.6)
Days since coronary event	58(52, 65)	61(41,84)
Previous heart attack	5/33(15%)	0/5
Diabetes, self report of physician diagnosis	13(37%)	1(20%)
Heart failure, self report of physician diagnosis	6/31(19%)	1(20%)
Hypertension, self report of physician diagnosis	23/33 (70%)	1/4(25%)
Blood pressure lowering medication*	35(100%)	5(100%)
Number of blood pressure lowering medications*, median(IQR)	3(2,3)	3(2,3)
Beta blocker use*	27(77%)	5(100%)
Statin use *	31(89%)	5(100%)
Current smoker/ex smoker/never smoker, number	N=31<br>1/13/17	N=5<br>0/2/3
Alcohol, never/ever drinkers,<br>number<br>Units/week (ever drinkers), median(IQR)	N=30<br>7/23<br>2(1,6)	 3/2<br>7(7,7)
Reason given for dropout		Refused follow-up (4)<br>Unwell (1)

*self-reported either pre-intervention or at follow-up

Tillin T, *et al. BMJ Open* 2019; 9:e030119. doi: 10.1136/bmjopen-2019-030119

Table S1b. Characteristics at start of study (pre intervention) of those who completed/did not complete 3 month follow-up. Yoga+usual care group

Means(95%CI), number(%) unless otherwise stated	Yoga+usual care Completed 3 month follow-up +/- 18 sessions	Yoga+usual care Attended 3 month follow-up and at least 18 yoga sessions	Yoga+usual care Dropped out of yoga and follow-up	Yoga+usual care Attended 3 month follow-up but attended <18 yoga sessions
	N=29 (4 did not complete 18 sessions)	N=25	N=11	N=4
Ethnicity: South Asian	17(59%)	15(60%)	8(73%)	2(50%)
Sex: Male	21(72%)	18(72%)	7(64%)	3(75%)
Age: years	58.5(54.5, 62.5)	57.9(53.6, 62.2)	54.5(47.9, 61.2)	62.2(42.8, 81.7)
Days since coronary event	51(42,59)	50±24	49±22	54(32,75)
Previous heart attack	4/28 (14%)	4/24(17%)	3/7(30%)	0/4
Diabetes, self report of physician diagnosis	12(41%)	12(41%)	3 (27%)	3 (75%)
Heart failure, self report of physician diagnosis	4/27(15%)	2/23(9%)	3/9(33%)	2(50%)
Hypertension, self report of physician diagnosis	23/28(82%)	19/24(79%)	5/8(63%)	4(100%)
Blood pressure lowering medication*	29(100%)	25(100%)	10/10(100%)	4(100%)
Number of blood pressure lowering medications*, median(IQR)	3(3,3)	3(3, 3)	3(2,3)	3(2,4)
Beta blocker use*	25(86%)	22(88%)	8(73%)	3(75%)
Statin use *	28(97%)	24(96%)	8(73%)	4(100%)
Current smoker/ex smoker,<br>number	N=27<br>2/10	N=23<br>2/8	2/4	0/2
Alcohol, never/ever drinkers,<br>number<br>Units/week (ever drinkers), median(IQR)	N=26<br>8/18<br>2(0,5)	N=23<br>8/15<br>2(0,4)	N=10<br>5/5<br>11(1,12)	 0/4<br>9(1, 20)
Reason given for dropout			Refused follow-up (4)<br>Unwell(6)<br>Returned to work, unable to attend further classes/follow-up (1)	Refused further yoga(4)

*self-reported either pre-intervention or at follow-up

Tillin T, *et al. BMJ Open* 2019; 9:e030119. doi: 10.1136/bmjopen-2019-030119
