# Supplementary figures and images for: Yoga and Cardiovascular Health Trial (YACHT): a UK-based randomised mechanistic study of a yoga intervention plus usual care versus usual care alone following an acute coronary event
Source: BMJ Open. 2019 Nov 3;9(11):e030119. doi: 10.1136/bmjopen-2019-030119 (PMC6858127; doi:10.1136/bmjopen-2019-030119)

Figure S1

## YACHT Study Recruitment

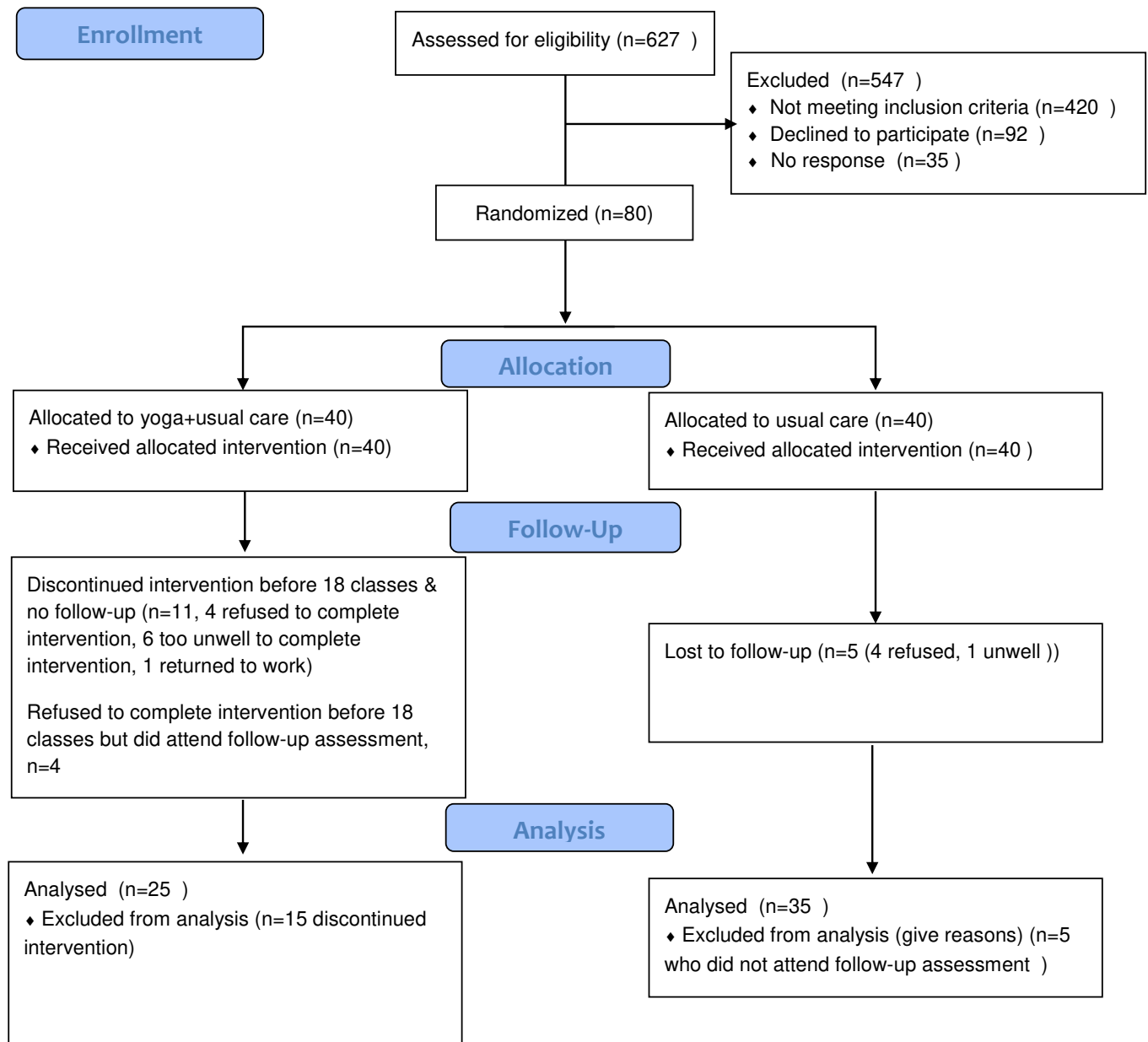

Supplement: Supplementary data [file bmjopen-2019-030119supp003.pdf]
