# Supplementary material for: Yoga and Cardiovascular Health Trial (YACHT): a UK-based randomised mechanistic study of a yoga intervention plus usual care versus usual care alone following an acute coronary event
Source: BMJ Open. 2019 Nov 3;9(11):e030119. doi: 10.1136/bmjopen-2019-030119 (PMC6858127; doi:10.1136/bmjopen-2019-030119)
Supplement: Author's manuscript [file bmjopen-2019-030119.draft_revisions.pdf]

BMJ Open is committed to open peer review. As part of this commitment we make the peer review history of every article we publish publicly available.

When an article is published we post the peer reviewers' comments and the authors' responses online. We also post the versions of the paper that were used during peer review. These are the versions that the peer review comments apply to.

The versions of the paper that follow are the versions that were submitted during the peer review process. They are not the versions of record or the final published versions. They should not be cited or distributed as the published version of this manuscript.

BMJ Open is an open access journal and the full, final, typeset and author-corrected version of record of the manuscript is available on our site with no access controls, subscription charges or pay-per-view fees (http://bmjopen.bmj.com).

If you have any questions on BMJ Open's open peer review process please email
info.bmjopen@bmj.com

BMJ Open

# Yoga and Cardiovascular Health Trial (YACHT): a UK-based randomised controlled trial of a yoga intervention plus usual care vs usual care alone following a coronary event

| | |
|---|---|
| Journal: | *BMJ Open* |
| Manuscript ID | bmjopen-2019-030119 |
| Article Type: | Original research |
| Date Submitted by the Author: | 28-Feb-2019 |
| Complete List of Authors: | Tillin, Therese; University College London, Institute of Cardiovascular Science<br>Tuson, Claire; York Hospitals NHS Trust<br>Sowa, Barbara; West London Mental Health NHS Trust<br>Chattopadhyay, Kaushik; University of Nottingham School of Health Sciences; London School of Hygiene and Tropical Medicine<br>Welsh, Paul; University of Glasgow<br>Sattar, Naveed; University of Glasgow<br>Roberts, Ian; London School of Hygiene and Tropical Medicine<br>Ebrahim, Shah; London School of Hygiene and Tropical Medicine<br>Kinra, Sanjay; London School of Hygiene and Tropical Medicine<br>Hughes, A; University College London, Institute of Cardiovascular Science<br>Chaturvedi, Nishi; University College London, Institute of Cardiovascular Science |
| Keywords: | Yoga, Cardiac rehabilitation, diastolic function, exercise, blood pressure, heart rate |
| | |

SCHOLARONE™
Manuscripts

# Yoga and Cardiovascular Health Trial (YACHT): a UK-based randomised controlled trial of a yoga intervention plus usual care vs usual care alone following a coronary event

Authors

Therese Tillin, Institute of Cardiovascular Science, UCL, corresponding author: email: t.tillin@ucl.ac.uk

Claire Tuson, York Teaching Hospital NHS Foundation Trust

Barbara Sowa, West London NHS Trust

Kaushik Chattopadhyay, Division of Epidemiology and Public Health, University of Nottingham

Naveed Sattar, Institute of Cardiovascular & Medical Sciences, University of Glasgow

Paul Welsh, Institute of Cardiovascular & Medical Sciences, University of Glasgow

Ian Roberts, London School of Hygiene and Tropical Medicine

Shah Ebrahim, London School of Hygiene and Tropical Medicine

Sanjay Kinra, London School of Hygiene and Tropical Medicine

Alun Hughes, Institute of Cardiovascular Science, UCL

Nishi Chaturvedi, Institute of Cardiovascular Science, UCL

For peer review only

Word count: abstract: 300 words, main text: 5082 words



For peer review only - http://bmjopen.bmj.com/site/about/guidelines.xhtml

**Abstract**

**Objective:** To determine effects of yoga practice on subclinical cardiovascular measures, risk factors and neuro-endocrine pathways following acute coronary events.

**Design:** 3-month, two arm (yoga+usual care vs usual care alone) parallel randomised controlled trial

**Setting:** Referrals from one general hospital and two primary care cardiac rehabilitation centres in London. Assessments were conducted at Imperial College London.

**Participants:** 80 participants, aged 35-80 years referred to cardiac rehabilitation programmes October 2012-April 2014. 68% were men, 60% were South Asian.

**Intervention:** The yoga intervention consisted of 18-24 group classes, conducted by a certified yoga teacher and included exercises in stretching, breathing, healing imagery and deep relaxation. It was pre-specified that yoga group participants should complete at least 18 classes for inclusion in analysis. Participants and partners in both groups were invited to attend once weekly a 6-12 week local standard NHS cardiac rehabilitation programme.

**Main outcome measures**: i) diastolic function, ii) distance walked, fatigue and breathlessness in a 6-minute walk test (6MWT), iii) BP, heart rate and peak $VO_2$ following a three-minute step exercise test. Effects on other measures of cardiac structure and function, the hypothalamus-pituitary-adrenal axis, autonomic function, body fat, blood lipids and glucose, stress and general health were also explored.

**Results** 25 participants in the yoga+usual care group and 35 participants in the usual care group completed the study. Following the 3-month intervention period, diastolic function was no better in the yoga group, (E/e': yoga: 8.81 (95% CI: 8.33,9.29), usual care: 8.26 (7.79,8.74)). The 6MWT and blood pressure, heart rate and peak $VO_2$ responses to the step exercise test and secondary outcomes showed no additional yoga-associated benefits.

**Conclusions** This study found that a structured 3-month yoga intervention added to usual care cardiac rehabilitation following an acute coronary event provided no evidence of additional beneficial effect on any cardiovascular or neuro-endocrine measures.

Keywords: Yoga, cardiac rehabilitation, diastolic function, exercise, blood pressure, heart rate

Trial registration https://clinicaltrials.gov/ct2/show/record/NCT01597960

For peer review only



For peer review only - http://bmjopen.bmj.com/site/about/guidelines.xhtml

## Article Summary

### Strengths and Limitations of this study

- Comprehensive clinical and subclinical cardiovascular measures before and after a yoga intervention (plus usual cardiac rehabilitation) vs usual cardiac rehabilitation
- Real world setting – older people following an acute coronary event
- High level of dropout, particularly in the yoga plus usual cardiac rehabilitation arm
- We can only assess the potential of yoga in addition to usual cardiac rehabilitation and not as an alternative.

## Introduction

The practice of yoga originated in ancient India as a form of exercise which includes breath control, the adoption of bodily postures and meditation which aim to increase strength and flexibility and to aid physical and mental wellbeing (1). Yoga has been shown to reduce stress and depression and is thought to improve biological cardiovascular risk factors. (2-4) However, despite claims of benefits, the effects of yoga on cardiovascular outcomes remain unclear. Previous systematic reviews (5-12) confirm that investigations of the health benefits of yoga and underlying mechanisms have often been hampered by poor study design, including small sample sizes, inadequate adjustment for confounders, lack of randomisation, unsatisfactory masking of outcomes to assessors, and publication bias. Also, many studies have been conducted in healthy young participants and it is not certain that these findings are generalisable to older adults with established disease.

In this UK-based randomised controlled study (Yoga and Cardiovascular Health Trial (YACHT)), we hypothesised that yoga would be associated primarily with improvements in cardiovascular function and exercise capacity both acutely and chronically. The chronic study compared cardiovascular measures at 3 months between two groups randomised either to usual care (cardiac rehabilitation) or to usual care plus a programme of yoga classes. Measures chosen for the acute study (before and after the first session of yoga) included blood pressure and heart rate before and after exercise as indicators of autonomic function which are associated with cardiovascular outcomes. (13, 14)

For the chronic study, where the emphasis was on rehabilitation following a coronary event, we focussed on the ratio between early mitral inflow and mitral annular early diastolic velocity (E/e') as our preferred single measure of diastolic function (15), a three-minute step test as a measure of cardiopulmonary fitness, and a 6-minute walk test (6MWT) as a measure of exercise tolerance, since these are reproducible and safe tests which are improved by cardiac rehabilitation. (16-18) All of these measures also predict outcomes in people with coronary heart disease (13-15, 19) and have been shown to be correlated with one another in hypertensive patients. (20, 21)

In addition to these primary outcome measures, we studied a range of other cardiovascular risk factors and measures which might be expected to improve following cardiac rehabilitation and provide mechanistic insight into any beneficial effect of yoga, these included markers of the hypothalamic–pituitary axis, measures of autonomic function, measures of cardiac structure and function, brachial and central resting and 24 hour ambulatory blood pressure, markers of atherosclerosis, blood glucose and lipids and self-reported health, lifestyle factors and perceived stress levels.



For peer review only - http://bmjopen.bmj.com/site/about/guidelines.xhtml

## Methods

Study population

Inclusion criteria included referral to cardiac rehabilitation programmes in north-west London following angioplasty, coronary artery bypass grafting or prescribed medical management only, as treatment for an acute coronary syndrome. Pre-specified inclusion criteria were age between 35 and 80 years, male or female, without co-morbid disease or mobility limitations that would preclude participation in cardiac rehabilitation and our investigations, and, given the north-west London area of recruitment, able to understand English or Punjabi. Ethnicity was self-defined, and verified by country of birth of all 4 grandparents. 80 participants were recruited following discharge from hospital and randomised in equal numbers to the yoga intervention plus their standard cardiac rehabilitation programme, or to standard cardiac rehabilitation programme (usual care) alone. Randomisation was performed by an independent researcher using a standard computerized algorithm (customised Java web application (srub)) and stratified by ethnicity (South Asian and non-South Asian), gender, 5 year age group and rehabilitation centre. The generated sequence was displayed only to the user at the time of assignment to the yoga intervention or usual care. 75% of participants were recruited from referrals to cardiac rehabilitation programmes at Ealing Hospital in west London, with the remainder recruited from two primary care cardiac rehabilitation programmes in north-west London (Harrow and Brent (Flexi-Heart Plan)). Recruitment of the planned 80 participants took place between October 2012 and April 2014, with the final participant seen for 3-month follow-up measures in July 2014.

Eligibility criteria were broadened in January 2013 and April 2013 respectively, with ethical approval, to include patients who had undergone coronary artery bypass grafting or who had received medical management only for their acute coronary event. The initial study plans were to recruit only patients referred to a cardiac rehabilitation programme post-angioplasty as treatment for an acute coronary syndrome. With cardiologist advice, it was felt that the earlier decision to exclude these patients based on safety grounds was unnecessary given the gentle and tailored nature of the exercises.

Patients and public were not involved in the study design, conduct, results, evaluation or dissemination.

Ethical approval for the study was granted by Camberwell St Giles Research Ethics Committee (Ref: 12/LO/0597). Informed written consent was obtained from all participants.

Yoga intervention

The yoga intervention was delivered on a twice-weekly group session basis for 12 weeks alongside the usual care, 6-12 week cardiac rehabilitation programme. There were 24 yoga classes in total. Participants' partners were invited to take part in each session as a method of improving adherence. The yoga session was designed and conducted by a teacher certified in yoga and cardiac rehabilitation, and included gentle exercises in deep relaxation, stretching, breathing, healing imagery and a healthy diet. A prescription of exercises with an accompanying DVD was provided to be performed regularly at home. Each session lasted approximately 75 minutes, divided into three equal parts: breathing exercises, yogic poses and meditations, education and discussion (details in supplemental material). Individuals randomised to the yoga arm had their standard cardiac rehabilitation care delivered at a separate time to those randomised to usual cardiac rehabilitation, (although delivered by the same teams), to reduce risks of contamination. Because the study was also designed to examine mechanisms underlying any beneficial effects of yoga (22), there was a pre-specified requirement for participants in the yoga + usual care group to complete at least 18 yoga classes.



For peer review only - http://bmjopen.bmj.com/site/about/guidelines.xhtml

Usual care

Usual care is described in the supplemental material and was similar in all centres in accordance with the UK's National Institute for Health and Care Excellence (NICE) guidelines (https://www.nice.org.uk/guidance/cg48, accessed 25/8/2017) and British Association for Cardiac Prevention and Rehabilitation (BACR) standards (23) with core components of lifestyle (physical activity, exercise, diet and weight management, smoking cessation), education, risk factor management, psychosocial, cardio-protective drug therapy and long-term management strategies. Patients and their partners were invited to attend once-weekly for a 6-12 week programme tailored to individual needs and including 1) on-going risk factor monitoring/advice/support, 2) exercise sessions in a gym, led by cardiac physiologist or a home-based exercise programme, 3) health education lectures (led by cardiac rehabilitation sister, pharmacist, dietician, clinical psychologist, cardiac physiologist), 4) relaxation sessions, 5) guidance and supervised use of the "Edinburgh Heart Manual" (http://www.theheartmanual.com/Pages/default.aspx, accessed 26/9/2017 ).

Outcome measures

Chronic study: All measures performed pre-intervention and 3 months post-intervention

Primary outcome measures

Left ventricular diastolic function

Transthoracic two-dimensional (2D) and Doppler echocardiography was performed as previously described (26). Transmitral flow velocity during the early filling phase (E) was acquired by pulsed Doppler and averaged from three consecutive cycles. Tissue Doppler Imaging was performed on the lateral and septal LV wall. Peak velocities during early diastole (e') were averaged from three consecutive representative cycles. The e' wave velocities measured from the lateral and septal walls were averaged. Intra- and inter-observer reproducibility of echocardiographic measures were assessed by separate scans (on different days) performed in 10 participants selected at random. Intra- and interobserver reproducibility were good (intraclass correlation coefficients >0.85).

The key measure of diastolic function was the ratio of early filling and early myocardial velocity (E/e') calculated as a non-invasive index of LV filling pressure.

Exercise capacity

Exercise capacity was measured during a 6-minute walk test conducted along a 30 m straight path in an outdoor covered area marked clearly with the beginning and end of each lap. Participants wore appropriate shoes and loose-fitting clothing and rested in a chair for 10 minutes before the start of the test. Fatigue and dyspnoea before the walk test were assessed using the Borg scale (27). Participants were asked to walk briskly as far as possible for a timed 6 minutes. Fatigue and dyspnoea were again assessed after the walk.

In addition, blood pressure and heart rate during a three minute step test (24) and peak $VO_2$ measured immediately post-exercise were measured pre-intervention and 3 months post-intervention as described for the acute study above.



For peer review only - http://bmjopen.bmj.com/site/about/guidelines.xhtml

Chronic study: Secondary outcome measures

Measures of cardiac structure and function were obtained as described under primary outcomes above and included left ventricular mass index, relative wall thickness, left atrial diameter, ejection fraction, mitral E/A ratio, s'(peak velocity during systole) and e'(peak velocity during early diastole).

Seated resting blood pressure and central blood pressure were measured using a Pulsecor BP+ device (Uscom Ltd, Sydney, Australia) (28) starting with the left arm and then repeated on the right arm. The average of the final 2 of 3 blood pressure readings for the right arm were used, unless the average SBP was more than 10 mm Hg greater than the average in the left arm, in which case the left arm average readings were used. A Vicorder oscillometric device (SMT Medical Germany/Skidmore Medical UK) (29) was used to measure carotid-femoral pulse wave velocity (PWV).

Ambulatory blood pressure monitoring was conducted using the oscillometric Mobilograph device (NuMed Healthcare, UK) (30) with an appropriately sised cuff worn on the non-dominant arm to record central blood pressure and heart rate for a 24 hour period; measurements were taken half-hourly between 0700 and 2100 hours and hourly during the night. Ambulatory blood pressure and heart rate analyses included the daytime period from 0900-2100 hours and the night-time period from 0100-0600 excluding the waking and bedtime periods of the day as these periods represent times during which bed rest is inconsistent and, therefore, cannot be categorised reliably. (31)

The HPA axis was assessed by salivary cortisol sampled at 5 points during the day pre-intervention and at 3 months follow-up as described for the acute study below. Fasting bloods were analysed for glucose and lipids at baseline and 3-month follow-up.

Heart rate variability (HRV) and baroreceptor sensitivity (BRS) were measured according to a published protocol (32). Briefly, these were measured in the recumbent position for a 10-minute period. Beat to beat arterial BP was recorded non-invasively using a Finometer (FMS Amsterdam, Netherlands), and the ECG was monitored using a 3 lead ECG. Signals were post processed as described in detail previously (32). For HRV we calculated the mean R-R interval, and mean spectral powers in the low frequency (LF: 0.04-0.15 Hz) and high frequency (HF: 0.15-0.4 Hz) bands for the R-R intervals. Frequency domain BRS was calculated as the alpha index given by the square root of the ratio between averaged powers of R-R and systolic BP for each frequency. Salivary amylase was measured at 5 time points during the day as described for cortisol below.

The full extra-cranial carotid artery was examined for the presence of plaque using an iE33 ultrasound machine (Philips) equipped with a linear-array transducer (L11_3) with concurrent recording of 3-lead ECG over 3-5 cardiac cycles. Carotid intima-media thickness (IMT) was measured in the distal 1 cm of the left common carotid artery from three longitudinal planes (anterior, lateral and posterior) in a region free of plaque with a clearly identified double-line pattern. Plaque was defined according to the Mannheim consensus as a focal structure encroaching into the arterial lumen by at least 0.5 mm or 50% of the surrounding IMT value, or a region of IMT having a thickness >1.5 mm. Analyses were performed using a validated semi-automated programme (AMS-II).

The GeneActiv wrist-worn waterproof accelerometry device was fitted at the end of the pre-intervention and 3-month follow-up visits and worn for 3 days after each visit. Analysis of the data was performed using a validated algorithm at the University of Newcastle (33) to provide average body acceleration (metric milli g where g is gravity) on days with more than 16 hours of valid readings.



For peer review only - http://bmjopen.bmj.com/site/about/guidelines.xhtml

For peer review only

Self-completion questionnaires were administered pre-interventions and at the 3-month follow-up as follows:

The international physical activity questionnaire (IPAQ) long version was administered and analysed according to the IPAQ guidelines (http://www.ipaq.ki.se/scoring.pdf, accessed Aug 25th 2017)

A self-completion questionnaire included items regarding frequency of alcohol consumption, number of units consumed and changes in drinking habits. Similar questions were included regarding smoking habits. A food frequency questionnaire, previously used in the SABRE tri-ethnic cohort study(34) covered the previous 7 days.

EQ-5D™ (https://euroqol.org/) is a standardised instrument for use as a measure of health outcome. It provides a simple descriptive profile a visual analogue scale to indicate self-rated health and a health status score based on UK population norms (there is no set of scores based on Indian Asian populations).

The perceived stress 10 item self-completion scale (35) was completed together with questions regarding sleep quality, snoring and breathlessness at night.

Acute study: Yoga+usual care group on day of first yoga session

Primary outcome measures - blood pressure and heart rate during a 3-minute step test and estimated peak oxygen consumption (peak $VO_2$) measured immediately post-exercise

This was performed directly before and after the first yoga session. Resting seated brachial blood pressure was measured after 5 minutes using an Omron 705CP device on the right arm. Participants then stood and were asked to step repeatedly on and off a step measuring 60x30x17.5 cm (length, width, height) for three minutes in time with a metronome set to 92 beats per minute (bpm). This corresponds to a rate of energy expenditure approximately 5 times the basal metabolic rate. (24) Standing BP and heart rate were measured on the right arm immediately afterwards and then again in the seated position after three minutes recovery. Peak $VO_2$ was estimated based on achieved heart rate in the immediate post-exercise period as described previously (25).

Secondary outcome measures

Saliva samples for amylase and cortisol were collected by the participants at home using a Salivette (www.salimetrics.com) collection kit at 5 time points during the day pre-intervention (waking, waking plus 30 minutes, waking plus 90 minutes, waking plus 12 hours, bedtime). For the acute study, waking, waking plus 12 hours and bedtime samples were taken on the day of the first yoga session. The latter two sampling points therefore occurred after the first yoga session. Samples were analysed using using indirect enzyme-linked immunosorbent assay kits (Salimetrics Europe Ltd., Suffolk, UK).

Blinding of observers

Post-processing of echocardiograms, carotid ultrasound scans, accelerometry, ambulatory blood pressure, heart rate variability and baroreceptor sensitivity, blood and saliva analyses were all conducted by observers blinded to participants' identity and study group. Clinic BP, vascular measurements and anthropometric measurements were conducted by clinic staff, who may have been aware of study group allocation, given the



For peer review only - http://bmjopen.bmj.com/site/about/guidelines.xhtml

nature of the interventions.

Location where data were collected

Data were collected at the International Centre for Circulatory Health on the St Mary's campus of Imperial College London (UK).

Statistical analyses

Sample size and power

The sample size was estimated for the primary outcome measures for the chronic effects of yoga, i.e. diastolic function on echocardiography and 6-minute walk test. Previous studies have reported at least half a standard deviation benefit associated with yoga on diastolic function (36). This approximates to an improvement of 1.1 in the E/e' ratio. For the 6-minute walk test, a median of 40±80 m (i.e. 0.5SD) is estimated as a clinically significant improvement in distance walked (37) – an improvement exceeded in a study of cardiac rehabilitation, where the distance walked increased by 62m. (38) Test-retest reliability of the test is good (0.9 for repeated measures). Statistical analyses were planned to be both unadjusted and to use regression modelling to adjust final measures for baseline differences, thus improving the precision of estimates of treatment effect, and shrinking the sample size requirement(39). This required knowledge of reproducibility or correlation of a given measure between baseline and follow-up, and was above 0.85 for repeated measures conducted by our own and other observers. (18) Using a conservative estimate of 0.70, and to allow for multivariable analysis, 33 completers were required in each arm of the study to detect a 0.5 standard deviation difference between groups (80% power and 5% significance). Thus, 40 people were recruited to each arm to allow for dropouts.

Statistical methods

Chronic study: summary descriptions of continuous pre-intervention characteristics are shown as means (95% CI) for Normally distributed data or as medians (95%CI of the median(CIM)) for non-Normally distributed variables or as number (%) for categorical variables. Pre-intervention characteristics are shown for the whole study group (Table 1) and for those who did and did not complete the study. (Table S1). Outcome analysis is restricted to those who attended the 3-month visit, and for the yoga group, additionally restricted to those who attended 18 out of the 24 yoga sessions, per protocol. A sensitivity analysis added 4 participants who did not complete the requisite number of yoga classes but who attended the 3-month study follow-up visit.

For the 3-minute step test which was conducted in three stages pre- and post-intervention, repeated measures ANOVA models were used to determine differences by intervention arm and timing (pre-intervention and 3 months follow-up for the chronic study) and for the acute study (pre- and post-first yoga session). Repeated measures ANOVA models were also used for salivary amylase and cortisol measured 5 times on 3 days (yoga + usual care group) or 2 days (usual care group).

The remaining measures were analysed using robust regression models, which are relatively efficient in the presence of outlier-prone error distributions. 3-month follow-up values were adjusted for the pre-intervention value of each Normally distributed measure, to provide adjusted mean (95%CI) values to allow comparison with pre-intervention observations. Where data were not Normally distributed pre-intervention, median regression provided comparable 3-month (median (95% CIM)) follow-up values adjusted for the pre-intervention value. Between- and within-group differences in categorical secondary outcome measures were tabulated and tested using the chi square test.



For peer review only - http://bmjopen.bmj.com/site/about/guidelines.xhtml

For heart rate variability and baroreceptor sensitivity, we conducted sensitivity analyses that excluded the few participants who were not receiving beta-blocker medication.

P values are shown for primary outcome data only and statistical significance accepted as $p<0.05$. Statistical analyses were performed using STATA version 15 software.

**Results**

80 participants were recruited and randomly assigned in equal numbers to the yoga plus usual care and usual care groups. Pre-intervention, average age was 57.1 (95% CI: 54.9, 59.4), 69% were male and 64% were of South Asian origin. Diabetes was present in 36%. The majority were receiving statins (90%) and/or anti-hypertensive medication (95%). (Table 1)

Thirty-five participants in the usual care arm (63% South Asian) and 25 participants in the yoga arm (59% South Asian) completed the study. Greater loss to follow-up occurred in the yoga group, mostly due to unwillingness to continue with yoga classes - participants frequently citing ill health as a reason, although one participant withdrew from the study because of return to work. (Consort Flow Diagram) Characteristics of those who completed the study and those who dropped out were similar pre-intervention. (Table S1) In addition to overall study dropout, several participants declined or were unable to undergo exercise testing either pre-or post-intervention, mostly due to mobility problems or elevated blood pressure (reasons are listed under Table 2).

No adverse events were reported. There was minimal change in the number and type of medications prescribed over the 3-month course of the study. (Tables 1 and S1)

Chronic study

Primary outcomes

Left ventricular diastolic function

At the three month follow-up, E/e' improved in both groups, but there was no evidence of yoga-related additional benefit in diastolic function (usual care: E/e' (adjusted for pre-intervention values) = 8.26 (95% CI: 7.79, 8.74), yoga+usual care: E/e'(adjusted for pre-intervention values) = 8.81 (8.33, 9.29), p=0.4). (Table 2)

6-minute walk test

The total distance walked increased in both groups at 3-months follow-up, but there was no evidence of yoga-related additional benefit (usual care: 491 (471, 512 )m, yoga+usual care: 488 (463, 513) m, p = 0.7; Table 2). Distance walked per minute also increased post-intervention to a similar level in both groups and there was no additional advantage related to yoga in the total number of minutes walked or in levels of fatigue and breathlessness. (Table 2)

3-minute step test

The results of the 3 minute step test at 3 month follow-up suggested some moderate improvements in immediate post-exercise BP, heart rate and peak $VO_2$ in both groups at follow-up, but there was no evidence of additional benefit associated with yoga. (Table 2)



For peer review only - http://bmjopen.bmj.com/site/about/guidelines.xhtml

Secondary outcomes

Other vascular measures

There was no evidence of yoga-related additional benefits for measures of clinic and ambulatory measures of brachial and central SBP at follow-up. Both groups showed improvements in resting brachial DBP and in resting central SBP. Pulse wave velocity was similar in the two groups at follow-up. (Table 3)

Carotid intima-media thickness

There was no evidence of additional yoga-related benefit on carotid IMT levels at 3 months. (Table 3)

Hypothalamic-Pituitary-Adrenal axis (HPA)

Salivary cortisol, as a marker of the HPA, decreased throughout the day in both groups pre-intervention and at 3 months follow-up. There was no evidence of additional yoga-related benefit  compared with usual care alone. (Table 3)

Autonomic function

There was no evidence of additional yoga-related benefit  compared with usual care alone on markers of heart rate variability baroreceptor sensitivity at 3 month follow-up and salivary amylase. (Table 4)

Metabolic measures

There was no evidence of additional yoga-related benefit  compared with usual care alone at 3 months follow-up in glucose, total cholesterol, LDL cholesterol. (Table 3)

Anthropometrics

Both groups had slightly lower waist to hip ratios at follow-up than at baseline, but with no evidence of yoga-related additional benefit compared with usual care alone. (Table 3)

Other measures

Accelerometry over 3 days showed that the usual care group modestly increased levels and the yoga group maintained levels of physical activity during the follow-up period. Self-reported physical activity (IPAQ) increased in both groups, with no evidence of additional yoga-related benefit  compared with usual care alone. (Table 3)

Similarly the EQ5D measures of health status or self-rated health at follow-up did not show any evidence of a treatment effect at follow-up.  The yoga group had lower stress scores than the usual care group  both pre-intervention and at follow-up and neither group reported appreciable change in stress score. (Table 3)

There were very few current smokers at baseline or follow-up and there were no between- group differences or within group changes. There were no between-group differences or significant within-group changes at follow-up in self-reported hours and quality of sleep, in alcohol consumption or in consumption of fresh fruit and vegetables. (not shown)

Sensitivity analyses



For peer review only

For peer review only - http://bmjopen.bmj.com/site/about/guidelines.xhtml

Sensitivity analyses of the primary outcomes which added those 4 participants who did not complete 18 yoga classes, but who did attend the 3 month follow-up clinic, did not alter findings. Likewise, exclusion of the few people who were not receiving beta blocker medication did not alter the findings for heart rate variability, baroreceptor sensitivity and salivary amylase.

Acute study (yoga arm only)

The 3-minute exercise step test was performed before and after the first yoga session (immediately post exercise and 3 minutes post-exercise). 27 participants undertook this test, 3 refused, 8 were unable to undertake exercise testing due to mobility problems and/or shortness of breath, one had unstable angina and in one case equipment failure resulted in loss of data. There was no convincing evidence of an acute effect of yoga on BP, heart rate or estimated peak $VO_2$ (Table 5a). Salivary cortisol and amylase were similar at the waking+12 hours and bedtime periods after the first yoga session compared with pre-intervention levels. (Table 5b)

Discussion

We show no additional cardiovascular benefit of a 3-month yoga intervention over and above usual cardiac rehabilitation in a randomised trial in participants who had experienced an acute coronary event. Specifically, there was no additional impact on our co-primary outcomes of exercise capacity and diastolic function, nor on a wide range of other outcome measures including cardiac structure and function, brachial, central and ambulatory blood pressure, blood pressure and heart rate responses to exercise, carotid intima media thickness, blood lipids and glucose, obesity measures including fat mass and body mass index, self-reported physical activity levels, distance walked in the 6 minute walk test alcohol, smoking and dietary intake. There was no convincing acute effect of yoga in exercise response in terms of blood pressure, heart rate and peak $VO_2$.

Of the cardiovascular risk factors studied to date, blood pressure appears the most consistently beneficially affected by yoga (8, 10, 40), with reports that reductions in blood pressure are similar to those obtained by anti-hypertensive medication(41). However, a community-based crossover study in India of non-pharmacological intervention showed that physical exercise (brisk walking for 50-60 minutes, 3-4 days a week for eight weeks) was the more effective method of reducing blood pressure, compared with yoga training or salt reduction, which had similar effects. (42) More recently, a community based randomised controlled trial in Sweden found equivalent reductions in resting blood pressure in control and yoga groups (following a 3-month yoga intervention)(43) - similar to our findings.

The acute effects of yoga on cardiovascular responses to exercise have not been well studied, although a study of 33 female college students in the USA suggested that salivary cortisol significantly decreased immediately after one hour sessions of power yoga or stretching, but decreased similarly following the control session which involved watching an educational movie for one hour (44). We saw little change in cortisol levels in the either group at 3-month follow-up or in the yoga group albeit later in the day following the first yoga session. At 3 months of follow-up, improvements in heart rate and peak $VO_2$ were seen in both groups compared with the pre-intervention levels. Likewise, both groups improved in terms of distance walked in the 6-minute walk test at 3 months of follow-up. A USA based study in heart failure patients reported a 0.5 SD improvement in exercise tolerance (+17% in 9 patients with heart failure enrolled to an 8 week yoga programme and –7% in 10 patients enrolled to receive standard medical therapy alone(45)). The same study also showed greater improvements in quality of life in the yoga group (scores improved by 26% in the yoga group and by 3% in the standard medical therapy group(45)). Again, our study participants, although unlikely to be directly



For peer review only - http://bmjopen.bmj.com/site/about/guidelines.xhtml

comparable (less than 20% reported previously diagnosed heart failure), demonstrated no evidence of yoga-related additional benefits. There was a small improvement in the EQ5D measure of health status based on UK population norms in the yoga group in our study, while there were small improvements in self-rated health and in the perceived stress score in both groups.

Measures of HRV are more sensitive to subtle changes than traditional tests of autonomic function, and improvements associated with yoga training or tai chi have been observed in systematic reviews for a number of parameters (46, 47) although we found little evidence for any yoga-related benefits in these parameters at follow-up.

Yoga has been variously shown to reduce fasting glucose and glycated haemoglobin, insulin, total and LDL cholesterol, triglyceride and weight, even in those without diabetes (48), although not all studies have shown a consistent benefit across these risk factors (7, 49, 50), and we found no yoga related benefits in blood lipids, glucose or obesity measures. Statin use was high (89%+) in both our study groups which may limit the measurable effect of the interventions on lipid levels.

Impacts of yoga on subclinical and clinical cardiovascular disease have been inconsistently studied, making it difficult to place in context our findings of little yoga-related additional benefit in cardiac diastolic function, although both groups appeared to have improved at follow-up. Improvements have been reported in diastolic function (diastolic time measured using a cuff-based device) in older hypertensive individuals in India (51) and, in a high risk subgroup of older individuals in the USA, in carotid intima media thickness (49). However, the latter effects were absent when the whole study population was analysed, and numbers randomised to comparator groups were small.

As noted earlier, there is a general difficulty in comparing studies due to the wide variation in study designs and populations. A recent systematic review of 306 randomised controlled trials of yoga found that 91% reached positive conclusions (52). The authors confirmed difficulty across all trials, regardless of where conducted, in comparison of results due to the common lack of *a priori* defined primary outcomes and often-even lack of between-group comparisons.

Strengths and limitations: This is the first study to our knowledge to adopt a comprehensive approach to measuring cardiovascular clinical and subclinical outcomes in response to a yoga intervention. It is also unusual in studying outcomes in a real world setting in an older group of people following acute coronary events. We acknowledge that the final numbers of completers in our study may have led us to be underpowered to detect small additional benefits of yoga, though given the lack of signal in many of our endpoints, we suggest that if additional benefits do exist, they are small. We did not adjust for multiple testing as we had identified *a priori* relevant primary outcomes for the trial. Additionally, adjustment for multiple testing would not have altered our interpretation given the null findings without adjustment. Dropout in the yoga arm of the study exceeded that in the usual care arm, (15 and 5 respectively), likely reflecting the dual burden of attending both yoga training and usual cardiac rehabilitation, which may have underestimated any potential effects of yoga. Cardiac rehabilitation is standard care in the UK, thus ethical reasons prevented comparison of yoga-based cardiac rehabilitation alone directly with usual cardiac rehabilitation- hence this study cannot tell us about the potential of yoga as an alternative to traditional cardiac rehabilitation. It should be noted that our study was designed as a mechanistic parallel study to the larger (around 4000 patients) Indian Council for Medical Research and Medical Research Council, UK funded study of yoga as a primary method of cardiac rehabilitation in India. (22)



For peer review only - http://bmjopen.bmj.com/site/about/guidelines.xhtml

Conclusion: In this UK-based randomised controlled trial of yoga classes plus usual cardiac rehabilitation compared with usual rehabilitation only following an acute coronary event or intervention, we found no evidence that the yoga programme conferred any additional benefits to cardiovascular or neuroendocrine health compared with usual cardiac rehabilitation care at 3 months of follow-up. We suggest that usual care cardiac rehabilitation programmes in the UK, which include exercise, and optimisation of medical therapy leave little additional scope for added benefits from a further intervention such as yoga.

Acknowledgements: We thank the cardiac rehabilitation teams at Ealing Hospital and the Flexi-Heart cardiac rehabilitation teams in Harrow and Brent for their help in identifying suitable participants; Daniel Key for building the study databases and managing the randomisation processes; Paula Carvelli, April McGowan for their help in recruitment and clinical measurements; Nadia do Couto Franscisco and Karikahan Manoharan for echocardiography measurements and analysis; and the participants for their valuable support.

Author contributions: All authors were involved in the design of the study. TT carried out the statistical analyses and wrote the first draft, with support from SK, AH, NC. All authors contributed to further drafts. The corresponding author attests that all listed authors meet authorship criteria and that no others meeting the criteria have been omitted. TT is the guarantor.

Transparency statement: The lead author (TT) affirms that the manuscript is an honest, accurate, and transparent account of the study being reported; that no important aspects of the study have been omitted; and that any discrepancies from the study as originally planned and registered have been explained.

Funding and role of the funding source: The study was funded by the UK Medical Research Council (MR/J000175/1). The funders and study sponsors have played no role in the study design; in the collection, analysis, and interpretation of data; in the writing of the report; nor in the decision to submit the article for publication. The researchers were independent from the funders and sponsors and all authors had full access to all of the data (including statistical reports and tables) in the study and can take responsibility for the integrity of the data and the accuracy of the data analysis.

Competing Interest statement: All authors have completed the ICMJE uniform disclosure form at http://www.icmje.org/coi_disclosure.pdf and declare no support from any organisation for the submitted work, no financial relationships with any organisations that might have an interest in the submitted work in the previous three years and no other relationships or activities that could appear to have influenced the submitted work.

Ethical approval was granted by Camberwell St Giles Research Ethics Committee (Ref: 12/LO/0597)

Data sharing statement: De-identified participant data are available upon reasonable request to Professor Nish Chaturvedi (n.chaturvedi@ucl.ac.uk)



For peer review only - http://bmjopen.bmj.com/site/about/guidelines.xhtml

References

1. Feuerstein G. The yoga tradition. Prescott: Hohm Press; 1998.
2. Choices N. A Guide to Yoga: Department of Health and Social Care, UK Government; [Available from: https://www.nhs.uk/live-well/exercise/guide-to-yoga/.
3. Chong CS, Tsunaka M, Tsang HW, Chan EP, Cheung WM. Effects of yoga on stress management in healthy adults: A systematic review. Alternative therapies in health and medicine. 2011;17(1):32-8.
4. Cramer H, Lauche R, Langhorst J, Dobos G. Yoga for depression: a systematic review and meta-analysis. Depression and anxiety. 2013;30(11):1068-83.
5. Cramer H, Langhorst J, Dobos G, Lauche R. Associated Factors and Consequences of Risk of Bias in Randomized Controlled Trials of Yoga: A Systematic Review. PLoS One. 2015;10(12):e0144125.
6. Cramer H, Lauche R, Haller H, Steckhan N, Michalsen A, Dobos G. Effects of yoga on cardiovascular disease risk factors: a systematic review and meta-analysis. Int J Cardiol. 2014;173(2):170-83.
7. Innes KE, Vincent HK. The influence of yoga-based programs on risk profiles in adults with type 2 diabetes mellitus: a systematic review. Evid Based Complement Alternat Med. 2007;4(4):469-86.
8. Jayasinghe SR. Yoga in cardiac health (a review). Eur J Cardiovasc Prev Rehabil. 2004;11(5):369-75.
9. Ross A, Thomas S. The health benefits of yoga and exercise: a review of comparison studies. J Altern Complement Med. 2010;16(1):3-12.
10. Yang K. A review of yoga programs for four leading risk factors of chronic diseases. Evid Based Complement Alternat Med. 2007;4(4):487-91.
11. Kwong JS, Lau HL, Yeung F, Chau PH. Yoga for secondary prevention of coronary heart disease. Cochrane Database Syst Rev. 2015(7):Cd009506.
12. Cramer H, Langhorst J, Dobos G, Lauche R. Yoga for metabolic syndrome: A systematic review and meta-analysis. Eur J Prev Cardiol. 2016;23(18):1982-93.
13. Filipovsky J, Ducimetiere P, Safar ME. Prognostic significance of exercise blood pressure and heart rate in middle-aged men. Hypertension. 1992;20(3):333-9.
14. Schultz MG, Otahal P, Cleland VJ, Blizzard L, Marwick TH, Sharman JE. Exercise-induced hypertension, cardiovascular events, and mortality in patients undergoing exercise stress testing: a systematic review and meta-analysis. Am J Hypertens. 2013;26(3):357-66.
15. Hillis GS, Moller JE, Pellikka PA, Gersh BJ, Wright RS, Ommen SR, et al. Noninvasive estimation of left ventricular filling pressure by E/e' is a powerful predictor of survival after acute myocardial infarction. Journal of the American College of Cardiology. 2004;43(3):360-7.
16. Sandercock G, Hurtado V, Cardoso F. Changes in cardiorespiratory fitness in cardiac rehabilitation patients: a meta-analysis. Int J Cardiol. 2013;167(3):894-902.
17. Bellet RN, Adams L, Morris NR. The 6-minute walk test in outpatient cardiac rehabilitation: validity, reliability and responsiveness--a systematic review. Physiotherapy. 2012;98(4):277-86.
18. Hanson LC, McBurney H, Taylor NF. The retest reliability of the six-minute walk test in patients referred to a cardiac rehabilitation programme. Physiotherapy research international : the journal for researchers and clinicians in physical therapy. 2012;17(1):55-61.
19. Beatty AL, Schiller NB, Whooley MA. Six-minute walk test as a prognostic tool in stable coronary heart disease: data from the heart and soul study. Arch Intern Med. 2012;172(14):1096-102.
20. Farag EM, Al-Daydamony MM, Gad MM. What is the association between left ventricular diastolic dysfunction and 6-minute walk test in hypertensive patients? J Am Soc Hypertens. 2017;11(3):158-64.
21. Yu CM, Li LS, Lam MF, Siu DC, Miu RK, Lau CP. Effect of a cardiac rehabilitation program on left ventricular diastolic function and its relationship to exercise capacity in patients with coronary heart disease: experience from a randomized, controlled study. Am Heart J. 2004;147(5):e24.
22. Chandrasekaran AM, Kinra S, Ajay VS, Chattopadhyay K, Singh K, Singh K, et al. Effectiveness and cost-effectiveness of a Yoga-based Cardiac Rehabilitation (Yoga-CaRe) program following acute myocardial infarction: Study rationale and design of a multi-center randomized controlled trial. Int J Cardiol. 2019.



For peer review only - http://bmjopen.bmj.com/site/about/guidelines.xhtml

23. BACPR. The BACPR Standards and Core Components for Cardiovascular Disease Prevention and Rehabilitation 2012. 2012.
24. Montoye HJ, Willis PW, 3rd, Cunningham DA, Keller JB. Heart rate response to a modified Harvard step test: males and females, age 10-69. Res Q. 1969;40(1):153-62.
25. Hughes AD, Chaturvedi N. Estimation of Maximal Oxygen Consumption and Heart Rate Recovery Using the Tecumseh Sub-Maximal Step Test and their Relationship to Cardiovascular Risk Factors. Artery Res. 2017;18:29-35.
26. Park CM, Tillin T, March K, Ghosh AK, Jones S, Wright A, et al. Hyperglycemia has a greater impact on left ventricle function in South Asians than in Europeans. Diabetes Care. 2014;37(4):1124-31.
27. Borg GA. Psychophysical bases of perceived exertion. Med Sci Sports Exerc. 1982;14(5):377-81.
28. Park CM, Korolkova O, Davies JE, Parker KH, Siggers JH, March K, et al. Arterial pressure: agreement between a brachial cuff-based device and radial tonometry. Journal of hypertension. 2014;32(4):865-72.
29. Pucci G, Cheriyan J, Hubsch A, Hickson SS, Gajendragadkar PR, Watson T, et al. Evaluation of the Vicorder, a novel cuff-based device for the noninvasive estimation of central blood pressure. Journal of hypertension. 2013;31(1):77-85.
30. Weiss W, Gohlisch C, Harsch-Gladisch C, Tolle M, Zidek W, van der Giet M. Oscillometric estimation of central blood pressure: validation of the Mobil-O-Graph in comparison with the SphygmoCor device. Blood pressure monitoring. 2012;17(3):128-31.
31. Staessen J, Bulpitt CJ, Fagard R, Mancia G, O'Brien ET, Thijs L, et al. Reference values for the ambulatory blood pressure and the blood pressure measured at home: a population study. Journal of human hypertension. 1991;5(5):355-61.
32. Bathula R, Hughes AD, Panerai R, Potter J, Thom SA, Francis DP, et al. Indian Asians have poorer cardiovascular autonomic function than Europeans: this is due to greater hyperglycaemia and may contribute to their greater risk of heart disease. Diabetologia. 2010;53(10):2120-8.
33. van Hees VT, Fang Z, Langford J, Assah F, Mohammad A, da Silva IC, et al. Autocalibration of accelerometer data for free-living physical activity assessment using local gravity and temperature: an evaluation on four continents. J Appl Physiol (1985 ). 2014;117(7):738-44.
34. Tillin T, Forouhi NG, McKeigue PM, Chaturvedi N. Southall And Brent REvisited: Cohort profile of SABRE, a UK population-based comparison of cardiovascular disease and diabetes in people of European, Indian Asian and African Caribbean origins. Int J Epidemiol. 2012;41(1):33-42.
35. Cohen S, Kamarck T, Mermelstein R. A global measure of perceived stress. J Health Soc Behav. 1983;24(4):385-96.
36. Konar D, Latha R, Bhuvaneswaran JS. Cardiovascular responses to head-down-body-up postural exercise (Sarvangasana). Indian J Physiol Pharmacol. 2000;44(4):392-400.
37. Schrover R, Evans K, Giugliani R, Noble I, Bhattacharya K. Minimal clinically important difference for the 6-min walk test: literature review and application to Morquio A syndrome. Orphanet journal of rare diseases. 2017;12(1):78.
38. Wright DJ, Khan KM, Gossage EM, Saltissi S. Assessment of a low-intensity cardiac rehabilitation programme using the six-minute walk test. Clinical rehabilitation. 2001;15(2):119-24.
39. Borm GF, Fransen J, Lemmens WA. A simple sample size formula for analysis of covariance in randomized clinical trials. Journal of clinical epidemiology. 2007;60(12):1234-8.
40. Hagins M, States R, Selfe T, Innes K. Effectiveness of yoga for hypertension: systematic review and meta-analysis. Evidence-based complementary and alternative medicine : eCAM. 2013;2013:649836.
41. Murugesan R, Govindarajulu N, Bera TK. Effect of selected yogic practices on the management of hypertension. Indian J Physiol Pharmacol. 2000;44(2):207-10.
42. Subramanian H, Soudarssanane MB, Jayalakshmy R, Thiruselvakumar D, Navasakthi D, Sahai A, et al. Non-pharmacological Interventions in Hypertension: A Community-based Cross-over Randomized Controlled Trial. Indian J Community Med. 2011;36(3):191-6.
43. Wolff M, Rogers K, Erdal B, Chalmers JP, Sundquist K, Midlov P. Impact of a short home-based yoga programme on blood pressure in patients with hypertension: a randomized controlled trial in primary care. Journal of human hypertension. 2016;30(10):599-605.

For peer review only



For peer review only - http://bmjopen.bmj.com/site/about/guidelines.xhtml

44. Sullivan M, Carberry A, Evans ES, Hall EE, Nepocatych S. The effects of power and stretch yoga on affect and salivary cortisol in women. Journal of health psychology. 2017:1359105317694487.
45. Pullen PR, Nagamia SH, Mehta PK, Thompson WR, Benardot D, Hammoud R, et al. Effects of yoga on inflammation and exercise capacity in patients with chronic heart failure. J Card Fail. 2008;14(5):407-13.
46. Zou L, Sasaki JE, Wei GX, Huang T, Yeung AS, Neto OB, et al. Effects of Mind(-)Body Exercises (Tai Chi/Yoga) on Heart Rate Variability Parameters and Perceived Stress: A Systematic Review with Meta-Analysis of Randomized Controlled Trials. Journal of clinical medicine. 2018;7(11).
47. Posadzki P, Kuzdzal A, Lee MS, Ernst E. Yoga for Heart Rate Variability: A Systematic Review and Meta-analysis of Randomized Clinical Trials. Applied psychophysiology and biofeedback. 2015;40(3):239-49.
48. Bijlani RL, Vempati RP, Yadav RK, Ray RB, Gupta V, Sharma R, et al. A brief but comprehensive lifestyle education program based on yoga reduces risk factors for cardiovascular disease and diabetes mellitus. J Altern Complement Med. 2005;11(2):267-74.
49. Fields JZ, Walton KG, Schneider RH, Nidich S, Pomerantz R, Suchdev P, et al. Effect of a multimodality natural medicine program on carotid atherosclerosis in older subjects: a pilot trial of Maharishi Vedic Medicine. Am J Cardiol. 2002;89(8):952-8.
50. Mahajan AS, Reddy KS, Sachdeva U. Lipid profile of coronary risk subjects following yogic lifestyle intervention. Indian Heart J. 1999;51(1):37-40.
51. Patil SG, Patil SS, Aithala MR, Das KK. Comparison of yoga and walking-exercise on cardiac time intervals as a measure of cardiac function in elderly with increased pulse pressure. Indian Heart J. 2017;69(4):485-90.
52. Cramer H, Lauche R, Langhorst J, Dobos G. Are Indian yoga trials more likely to be positive than those from other countries? A systematic review of randomized controlled trials. Contemp Clin Trials. 2015;41:269-72.

For peer review only



For peer review only - http://bmjopen.bmj.com/site/about/guidelines.xhtml

Table 1: Pre-intervention characteristics by randomisation group

| N(%) or means(95%CI) unless otherwise stated | Yoga + usual care | Usual Care |
|---|---|---|
| Pre-intervention | N=40 | N=40 |
| Ethnicity: South Asian | 25(63%) | 26(65%) |
| Sex: Male | 28(70%) | 26(67%) |
| Age: years | 57.4(54.1, 60.7), range(35, 77) | 56.9(53.8, 60.0), range(35, 78) |
| Days since coronary event | 50(43, 57) | 59(53, 65) |
| Diabetes* (self report of physician diagnosis/anti-diabetic medication) | 15(38%) | 14 (35%) |
| Hypertension*( self report of physician diagnosis) | 29/37 (78%) | 25/37(68%) |
| Heart Failure* (self report of physician diagnosis) | 7/29(19%) | 7/29(19%) |
| Antihypertensive medications* | 39(98%) | 36(90%) |
| Number of antihypertensive medications, median(interquartile range)* | 3(2,3) | 3(2,3) |
| Beta blockers* | 33(83%) | 32(80%) |
| Statins* | 36(90%) | 36(90%) |
| Current smoker/ex/never smoker, number* | 4/14/19 | 1/14/24 |
| Alcohol: never/ever drinkers, number* | N=36<br>Never drinkers: 13<br>Ever drinkers: 23 | N=35<br>Never drinkers: 10<br>Ever drinkers: 25 |
| Units/week (ever drinkers)*, median(IQR) | 2.5(0, 11) | 4(1, 7) |
| Currently employed* | N=35<br>15(43%) | N=32<br>15(47%) |

*self-reported, N=number of responses to questionnaire item if incomplete

For peer review only

Table 2: <u>Chronic study: **Primary outcomes**</u>: Recruitment and three month follow-up (includes only those who attended study clinics at both time points and attended at least 18 classes if in the yoga group: N=35 in usual care group and N=25 in yoga group, unless otherwise stated)

| | Pre-intervention Means(95%CI) | | 3 months follow-up Means(95%CI), adjusted for pre-intervention levels | | P value for between group difference, adjusted for pre-intervention levels |
|---|---|---|---|---|---|
| | Yoga + usual care | Usual care | Yoga + usual care | Usual care | |
| **Diastolic function** | N=25 | N=33* | N=25 | N=33* | |
| E/e' | 9.74(8.37, 11.12) | 8.72(7.76, 9.68) | 8.81(8.33, 9.29) | 8.26(7.79, 8.74) | 0.4 |
| **6-minute walk test** | N=19*** | N=30** | N=19*** | N=30** | |
| Total Distance, m | 462(449, 517) | 442 (402,482) | 488(463, 513) | 491(471, 512) | 0.7 |
| Total minutes walked | 6.0(6.0, 6.0) | 5.8(5.4, 6.0) | 6.0(5.7. 6.3) | 5.8(5.5, 6.0) | 0.5 |
| Distance, m/minute | 77(72, 82) | 77(71, 82) | 81(78, 83) | 81(78, 83) | 0.7 |
| Fatigue (Borg scale: 0-10) | | | | | |
| Pre test | 0.2(0, 0.7) | 0.2(0, 0.5) | 0.07(0, 0.16) | 0(0, 0.06) | 0.17 |
| Post-test | 0.6(0, 1.4) | 0.7(0.03, 1.4) | 0.08(0, 0.20) | 0.08(0, 0.19) | 0.9 |
| Dyspnoea (Borg Scale: 0-10) | | | | | |
| Pre-test | 0 (0, 0) | 0.07(0, 0.23) | 0(0, 0.09) | 0.04(0, 0.10) | 0.5 |
| Post-test | 0.6(0, 1.2) | 1.0(0.3, 1.7) | 0.2(0, 0.8) | 0.6(0.2, 1.0) | 0.5 |
| **Response to exercise:3-minute step test** | N=18*** | N=30** | N=18*** | N=30** | |
| <u>Pre-step test</u> | | | | | |
| Brachial SBP, mm Hg | 140(134, 146) | 135(130, 140) | 138(133, 140) | 131(126, 136) | 0.4 |
| Brachial DBP, mm Hg | 83(80, 85) | 80(78, 83) | 82(79, 84) | 79(77, 82) | 0.6 |
| Heart rate, bpm | 60(56, 64) | 62(59, 66) | 58(54, 61) | 60(57, 63) | 0.4 |
| <u>Immediately post-step test</u> | | | | | |
| Brachial SBP, mm Hg | 161(155, 167) | 152(147, 157) | 156(151, 162) | 149(145, 155) | 0.6 |
| Brachial DBP, mm Hg | 85(82, 87) | 80(78, 83) | 77(74, 80) | 77(74, 79) | 0.9 |
| Heart rate, bpm | 89 (85, 93) | 89(86,93) | 81(77, 85) | 85(82, 88) | 0.2 |
| Peak $VO_2$ ml/min/kg | N=14 35.7(31.5, 40.0) | N=27 38.2(34.3, 42.1) | N=14 41.3(38.0, 44.6) | N=27 42.6(39.3, 45.8) | 0.6 |
| <u>3 minutes post-step test</u> | | | | | |
| Brachial SBP, mm Hg | 143(137, 149) | 134(129, 139) | 144(138, 150) | 136(131, 140) | 0.4 |
| Brachial DBP, mm Hg | 83(80, 86) | 80(78, 83) | 80(77, 83) | 79(77, 82) | 1.0 |
| Heart rate, bpm | 66(62, 70) | 65(61, 68) | 61 (57, 65) | 60(58, 64) | 1.0 |

*2 missing cases due to error readings
**5 missing cases: test not performed due to poor mobility(3), recent myocardial infarction(1), refused(1)
***6/7missing cases: test not performed due to poor mobility(2), elevated blood pressure(>180/100mm Hg)(4), refused step test(1)

For peer review only



For peer review only - http://bmjopen.bmj.com/site/about/guidelines.xhtml

Table 3: <u>Chronic study**: Secondary outcomes**</u>: Pre-intervention and 3-month follow-up (includes only those who attended study clinics at both time points and attended at least 18 classes if in the yoga group).

N=35 in usual care group and N=25 in yoga group, unless otherwise stated

| | Pre-intervention Means (95%CI) unless stated otherwise | | 3 months follow-up Means (95%CI) adjusted for pre-intervention levels unless stated otherwise | |
|---|---|---|---|---|
| | Yoga + usual care | Usual care | Yoga + usual care | Usual care |
| **Cardiac structure** | | | | |
| LV mass index, g/m^2.7, median(95%CI) | 40.1(36.6, 51.2) | 38.7(35.6, 43.5) | 39.4(36.2, 42.5) | 39.3(36.1, 42.5) |
| Relative wall thickness, median(95%CI) | 0.45(0.38, 0.48) | 0.42(0.40, 0.47) | 0.41(0.39, 0.44) | 0.42(0.39, 0.44) |
| Left atrial diameter indexed to height, cm/m | 2.2(2.2, 2.3) | 2.3(2.2, 2.4) | 2.2(2.2, 2.3) | 2.2(2.2, 2.3) |
| **Cardiac function** | | | | |
| Ejection fraction, median(95%CI) | 0.54(0.44, 0.68) | 0.54(0.44, 0.64) | 0.54(0.44, 0.63) | 0.54(0.45, 0.63) |
| Mitral E:A ratio, median(95%CI) | 1.02(0.97, 1.12) | 1.16(0.95, 1.29) | 1.09(1.01, 1.18) | 1.14(1.07, 1.23) |
| s', peak velocity during systole, cm/sec, median(95%CI) | 7.11(6.49, 8.25) | 7.31(6.35, 7.64) | 7.25(6.90, 7.59) | 7.16(6. 82, 7.51) |
| e', peak velocity during early diastole, cm/sec, median(95%CI) | 7.56(6.77, 9.35) | 8.57(7.58, 9.44) | 8.20(7.73, 8.67) | 8.45(7.98, 8.92) |
| **Resting/ambulatory blood pressure and heart rate** | N=25 | N=35 | N=25 | N=35 |
| Heart rate, bpm | 60(55, 64) | 64(60, 68) | 59(57, 61) | 61(60, 63) |
| Brachial SBP, mm Hg | 134(125, 143) | 126(120, 133) | 127(124, 130) | 122(119, 125) |
| Brachial DBP, mm Hg | 77(74, 80) | 74(72, 76) | 75(73, 76) | 73(71, 74) |
| Central SBP, mm Hg | 128(119, 137) | 120(114, 125) | 122(119, 125) | 117(114, 120) |
| **24 hour ambulatory blood pressure** | N=20 | N=30 | N=20 | N=30 |
| Average day central SBP, mm Hg | 115(108, 122) | 113(109, 116) | 113(110, 116) | 112(109, 114) |
| Average night central SBP, mm Hg | 104(96, 112) | 104(98, 110) | 104(100,108) | 104(100, 108) |
| Average day heart rate, bpm | 65(60, 69) | 68(65, 71) | 64(63, 66) | 67(65, 69) |
| Average night heart rate, bpm | 59(54, 63) | 64(60, 67) | 59(57, 61) | 63(61, 65) |
| **Pulse wave velocity**, m/sec **medians(95%CIM))** | N=25<br>9.03(8.14, 9.67) | N=32<br>8.63(8.27, 9.10) | N=25<br>9.02(8.47, 9.59) | N=32<br>8.75(8.28, 9.22) |
| **Carotid intima media thickness**. Far wall, mm, maximum of means (without plaque &IMT<1.5mm) | N=18<br>0.754(0.691, 0.816) | N=28<br>0.746(0.681, 0.810) | N=18<br>0.764(0.699, 0.830) | N=28<br>0.761(0.696, 0.827) |
| **Bloods, fasting, medians(95%CIM)** | N=25 | N=31 | N=25 | N=31 |
| Triglycerides, mmol/l | 1.10 (0.94, 1.40) | 1.03(0.96, 12.4) | 1.11(1.01, 1.20) | 1.10(1.00, 1.19) |
| HDL cholesterol, mmol/l | 0.91(0.84, 1.07) | 0.91(0.79, 1.01) | 1.02(0.97, 1.06) | 0.97(0.92, 1.02) |
| Total cholesterol, mmol/l | 3.00(2.80, 3.50) | 3.20(2.90, 3.60) | 3.30(3.18, 3.41) | 3.36(3.25, 3.47) |
| Cholesterol:HDL ratio | 3.33(2.82, 3.67) | 3.40(3.23, 3.78) | 3.31(3.13, 3.48) | 3.52(3.34, 3.69) |
| LDL cholesterol, mmol/l | 1.59(1.35, 1.77) | 1.60(1.54, 1.73) | 1.76(1.66, 1.87) | 1.81(1.71, 1.92) |
| Glucose, mmol/l | 5.40(4.90, 6.0) | 5.50(4.90, 6.20) | 5.78(5.62, 5.94) | 5.68(5.52, 5.84) |
| **Anthropometrics** | N=25 | N=35 | N=25 | N=35 |
| BMI, kg/m$^2$, median(95%CIM) | 27.6 (25.1, 29.5) | 27.2(25.3, 29.6) | 27.6(27.4, 27.9) | 27.5(27.3, 27.7) |
| Waist:hip ratio | 0.99(0.96, 1.03) | 0.99(0.96, 1.02) | 0.98(0.97, 0.99) | 0.98(0.97, 0.98) |
| Fat mass percent | 28(25, 32) | 30(27, 33) | 28(27, 28) | 30(29, 30) |
| **HPA axis** | | | | |



For peer review only - http://bmjopen.bmj.com/site/about/guidelines.xhtml

For peer review only

| | Pre-intervention Means (95%CI) unless stated otherwise | | 3 months follow-up Means (95%CI) adjusted for pre-intervention levels unless stated otherwise | |
|---|---|---|---|---|
| | Yoga + usual care | Usual care | Yoga + usual care | Usual care |
| **Salivary cortisol nmol/L** | N=23 | N=29 | N=23 | N=29 |
| Waking | 11.2(8.5, 13.8) | 12.6(10.3, 14.9) | 11.2(8.7, 13.7) | 12.5(10.4, 14.7) |
| Waking+30 minutes | 10.6(8.0, 13.3) | 16.0(13.6, 18.3) | 10.4(8.0, 12.9) | 12.9(10.7, 15.0) |
| Waking +1hrs 30 minutes | 5.2(2.5, 7.8) | 8.0(5.6, 10.4) | 8.0(5.6, 10.4) | 7.5(5.3, 9.7) |
| Waking +12 hours | 3.9(1.2, 6.7) | 3.2(0.8, 5.7) | 4.1(1.7, 6.6) | 2.5(0.3, 4.7) |
| Bedtime | 2.3(0, 5.0) | 3.1(0.7, 5.5) | 3.7(1.3, 6.2) | 2.2(0.04, 4.3) |
| **Exercise/physical activity** | | | | |
| **Average body acceleration over 3 days**, milli g (GeneActiv) | N=20<br>25.1(20.4, 29.8) | N=28<br>22.5(19.5, 25.5) | N=20<br>24.9(22.7, 27.1) | N=28<br>23.5(21.3, 25.7) |
| **IPAQ Physical activity** self-report. Total met minutes/week. Median(95%CIM) | N=25<br>693(60, 1386) | N=34<br>1409(495, 2310) | N=25<br>2273(1434, 3112) | N=34<br>2899(2065, 3734) |
| **EQ5D** health status based on UK population norms(1= full health) median (95%CI) | N=14<br>0.77(0.69, 1.0) | N=21<br>0.80(0.73, 0.81) | N=14<br>0.83(0.70, 0.97) | N=21<br>0.80(0.66, 0.93) |
| **EQ5D** self-rated health thermometer (100=best possible) median(95%CIM) | N=21<br>70(60,75) | N=27<br>70(50, 80) | N=25<br>73(68, 78) | N=27<br>73(68, 78) |
| **Perceived stress score** (possible range: 0-40. 13 is considered average, high stress groups: 20+) | N=25<br>14.9(11.8, 18.0) | N=34<br>18.2(15.2, 21.2) | N=25<br>14.7(13.1, 16.4) | N=34<br>17.2(15.5, 18.8) |

For peer review only



For peer review only - http://bmjopen.bmj.com/site/about/guidelines.xhtml

Table 4. Chronic Study: Autonomic function: heart rate variability, baroreceptor sensitivity and salivary amylase

| | Pre-intervention | | 3 months follow-up adjusted for Pre-intervention level | |
|---|---|---|---|---|
| | Yoga + usual care | Usual care | Yoga + usual care | Usual care |
| **Heart rate variability and baroreceptor sensitivity 10 minute recording** Medians (95%CIM) | N=24 | N=33 | N=24 | N=33 |
| Number of beats | 677(605, 877) | 709(632, 798) | 644 (589, 698) | 638 (584, 693) |
| Number of ectopics | 19 (8, 71) | 22 (8, 48) | 29 (6, 53) | 26 (2, 49) |
| Mean RR interval , ms | 1016(888, 1128) | 977(925, 1073) | 1050(1012, 1089) | 1020(982, 1059) |
| SDNN, ms | 55.5(39.7, 78.6) | 53.4 (37.4, 69.2) | 48.2(36.7, 59.6) | 47.2(35.9, 58.6) |
| RMSSD, ms | 38.5(30.0, 60.0) | 38.8(30.2, 57.8) | 44.4(35.2, 53.5) | 39.9(30.8, 48.9) |
| NN50 | 40(18,57) | 49(24,67) | 49 (31, 66) | 51(34, 68) |
| pNN50 | 0.08 (0.03, 0.11)) | 0.08(0.04, 0.14) | 0.14(0.10, 0.17) | 0.11(0.07,0.15) |
| Triangular index | 179(149, 231) | 177(139, 235) | 189 (163, 216) | 187(160, 214) |
| Total RR interval power, ms2 | 1514(633, 2339) | 995(680, 2097) | 1157(653, 1662) | 1171(668, 1673) |
| LF RR interval power, ms2 | 340(119, 613) | 284 (177, 491) | 340(143, 537) | 340(143, 538) |
| HF RR interval power, ms2 | 251(77, 759) | 235(126, 317) | 265(113, 417) | 265(113, 16) |
| LF/HF power ratio | 1.5(0.8, 2.1) | 1.4(1.1, 1.7) | 1.2(0.9, 1.4) | 1.2 (0.9, 1.4) |
| LF RR interval power, normalised units(nu) | 0.23(0.16, 0.32) | 0.27(0.25, 0.30) | 0.28(0.24, 0.31) | 0.29(0.26, 0.32) |
| HF RR interval power, nu | 0.17(0.12, 0.33) | 0.19(0.14, 0.28) | 0.23(0.17, 0.29 | 0.23(0.17, 0.29) |
| LF alpha index, ms/mm Hg | 6.9 (4.6, 12.9) | 9.1(6.6, 13.6) | 10.1 (7.5, 12.7) | 10.4(7.8, 13.0) |
| HF alpha index, ms/mm Hg | 9.2(4.6, 25.6) | 13.9(9.2, 22.6) | 15.8 (7.7, 23.9) | 16.4(8.3, 24.5) |
| BRS on sequence analysis, ms/mmHg | 9.9 (6.3, 16.1) | 10.0(7.0, 13.0) | 10.6 (8.2, 13.1) | 10.6(8.2, 13.0) |
| **Salivary amylase, nmol/ml ,** (means(95%CI)) | N=23 | N=32 | N=23 | N=32 |
| Waking | 61(46, 82) | 88(69, 114) | 80(58,110) | 86(65, 115) |
| Waking+30 minutes | 56(42, 76) | 59(46, 76) | 56(41, 77) | 71(53, 94) |
| Waking +1hrs 30 minutes | 86(64, 115) | 131(101, 170) | 88(64, 121) | 88(66, 117) |
| Waking +12 hours | 100(74, 136) | 114(88, 147) | 73(53, 101) | 110(83, 146) |
| Bedtime | 83(61, 112) | 106(82, 136) | 95(69, 131) | 85(64, 113) |

For peer review only



For peer review only - http://bmjopen.bmj.com/site/about/guidelines.xhtml

Table 5a: Acute study, **Primary outcomes**: immediate post-exercise results following a 3-minute step test, before and after (same day) the first yoga class

| **N=27** | **Before first yoga session** Mean (95%CI) | **After first yoga session:** Mean (95% CI) | **P value for comparison between before and after first yoga session** |
|---|---|---|---|
| **Pre-exercise, resting seated** | | | |
| Systolic blood pressure, mm Hg | 132(128, 136) | 133(129, 136) | 0.9 |
| Diastolic blood pressure, mm Hg | 78(77, 81) | 79(77, 81) | 0.7 |
| Heart rate, bpm | 69(67, 71) | 68(66, 70) | 0.9 |
| **Immediately post-exercise, standing** | | | |
| Systolic blood pressure, mm Hg | 161(157, 164) | 156(148, 163) | 0.4 |
| Diastolic blood pressure, mm Hg | 80(78, 82) | 80(78, 82) | 0.8 |
| Heart rate, bpm | 92(90, 94) | 88(86, 90) | 0.4 |
| Peak $VO_2$, ml/min/kg (n=24) | 36.9(33.4, 40.4) | 38.9(35.4, 42.4) | 0.07 |
| **3 minutes post-exercise, seated** | | | |
| Systolic blood pressure, mm Hg | 133(129, 136) | 135(131, 139) | 0.7 |
| Diastolic blood pressure, mm Hg | 78(76, 80) | 79(77, 82) | 0.6 |
| Heart rate, bpm | 72(70, 74) | 69(67, 71) | 0.4 |

*12 participants did not participate in step test both before and after the first yoga session due to: mobility problems+/- shortness of breath (n=3), refused(n=3), frailty (n=2), unstable angina(n=1), other (high blood pressure/dizziness/weakness on left side/restricted movement; n=3). Blood pressure readings were unavailable due to equipment failure for 1 participant

Table 5b: Acute study: Secondary outcomes: salivary amylase and cortisol: pre-intervention and day of first yoga class

| | **Pre-intervention** **Means(95%CI)** | **Day of first yoga session** **Means(95%CI)** |
|---|---|---|
| | N=32* | N=32* |
| **Cortisol (nmol/l** | | |
| Waking | 12.7(11.0, 14.4) | 15.4(12.8, 17.9) |
| 12 hours after waking | 3.6(1.8, 5.4) | 4.4(1.7, 7.0) |
| Bedtime | 3.4(1.6, 5.2) | 3.0(0.3, 5.7) |
| **Amylase (microunits/ml)** | | |
| Waking | 82(69, 96) | 72(56, 93) |
| 12 hours after waking | 105(88, 126) | 98 (75, 128) |
| Bedtime | 99(83, 119) | 114(87, 149) |

*8 participants were unable to provide adequate saliva samples pre-recruitment and on the day of the first yoga session



For peer review only - http://bmjopen.bmj.com/site/about/guidelines.xhtml

For peer review only

SUPPLEMENTAL Table S1a. Characteristics at start of study (pre intervention) of those who completed/did not complete 3month follow-up: Usual care group

| Means95%CI), number(%) unless otherwise stated | Usual Care, completed | Usual Care, dropped out |
|---|---|---|
| | N=35 | N=5 |
| Ethnicity: South Asian | 22(63%) | 4(80%) |
| Sex: Male | 22(63%) | 5(100%) |
| Age: years | 57.2(54.0, 59.8) | 54.5(38.3,73.6) |
| Days since coronary event | 58(52, 65) | 61(41,84) |
| Previous heart attack | 5/33(15%) | 0/5 |
| Diabetes, self report of physician diagnosis | 13(37%) | 1(20%) |
| Heart failure, self report of physician diagnosis | 6/31(19%) | 1(20%) |
| Hypertension, self report of physician diagnosis | 23/33 (70%) | 1/4(25%) |
| Blood pressure lowering medication* | 35(100%) | 5(100%) |
| Number of blood pressure lowering medications*, median(IQR) | 3(2,3) | 3(2,3) |
| Beta blocker use* | 27(77%) | 5(100%) |
| Statin use * | 31(89%) | 5(100%) |
| Current smoker/ex smoker/never smoker, number | N=31<br>1/13/17 | N=5<br>0/2/3 |
| Alcohol, never/ever drinkers, number<br>Units/week (ever drinkers), median(IQR) | N=30<br>7/23<br>2(1,6) | <br>3/2<br>7(7,7) |
| Reason given for dropout | | Refused follow-up (4)<br>Unwell (1) |

*self-reported either pre-intervention or at follow-up

For peer review only

Table S1b. Characteristics at start of study (pre intervention) of those who completed/did not complete 3 month follow-up. Yoga+usual care group

| Means(95%CI), number(%) unless otherwise stated | Yoga+usual care Completed 3 month follow-up +/- 18 sessions | Yoga+usual care Attended 3 month follow-up and at least 18 yoga sessions | Yoga+usual care Dropped out of yoga and follow-up | Yoga+usual care Attended 3 month follow-up but attended <18 yoga sessions |
|---|---|---|---|---|
| | N=29 (4 did not complete 18 sessions) | N=25 | N=11 | N=4 |
| Ethnicity: South Asian | 17(59%) | 15(60%) | 8(73%) | 2(50%) |
| Sex: Male | 21(72%) | 18(72%) | 7(64%) | 3(75%) |
| Age: years | 58.5(54.5, 62.5) | 57.9(53.6, 62.2) | 54.5(47.9, 61.2) | 62.2(42.8, 81.7) |
| Days since coronary event | 51(42,59) | 50±24 | 49±22 | 54(32,75) |
| Previous heart attack | 4/28 (14%) | 4/24(17%) | 3/7(30%) | 0/4 |
| Diabetes, self report of physician diagnosis | 12(41%) | 12(41%) | 3 (27%) | 3 (75%) |
| Heart failure, self report of physician diagnosis | 4/27(15%) | 2/23(9%) | 3/9(33%) | 2(50%) |
| Hypertension, self report of physician diagnosis | 23/28(82%) | 19/24(79%) | 5/8(63%) | 4(100%) |
| Blood pressure lowering medication* | 29(100%) | 25(100%) | 10/10(100%) | 4(100%) |
| Number of blood pressure lowering medications*, median(IQR) | 3(3,3) | 3(3, 3) | 3(2,3) | 3(2,4) |
| Beta blocker use* | 25(86%) | 22(88%) | 8(73%) | 3(75%) |
| Statin use * | 28(97%) | 24(96%) | 8(73%) | 4(100%) |
| Current smoker/ex smoker, number | N=27<br>2/10 | N=23<br>2/8 | 2/4 | 0/2 |
| Alcohol, never/ever drinkers, number<br>Units/week (ever drinkers), median(IQR) | N=26<br>8/18<br>2(0,5) | N=23<br>8/15<br>2(0,4) | N=10<br>5/5<br>11(1,12) | <br>0/4<br>9(1, 20) |
| Reason given for dropout | | | Refused follow-up (4)<br>Unwell(6)<br>Returned to work, unable to attend further classes/follow-up (1) | Refused further yoga(4) |

*self-reported either pre-intervention or at follow-up



For peer review only - http://bmjopen.bmj.com/site/about/guidelines.xhtml

For peer review only



For peer review only - http://bmjopen.bmj.com/site/about/guidelines.xhtml

# YACHT

**Y**oga **A**nd **C**ardiovascular **H**ealth **T**rial

# Manual of Operations

Version 1.2

July 2012

**Compiled by Barbara Sowa and Claire Tuson**

review only

Imperial College London

# Table of Contents


**STUDY SUMMARY** ........ **3**

**OUTLINE STUDY DESIGN** ........ **4**

**YOGA SUMMARY** ........ **5**

**YOGA FULL DESCRIPTION** ........ **5**

Part 1: Initial relaxation and warm-up – 10 minutes ........ 5

*Initial relaxation* ........ *6*

*Warm-up* ........ *7*

Part II: Asanas - 20 minutes ........ 8

*Standing poses (High positions)* ........ *8*

*Kneeling & Sitting poses (Medium positions)* ........ *10*

*Lying down poses (Low positions)* ........ *13*

Part III: Cool Down: Breathing exercises and final relaxation - 20 minutes ........ 14

*Deep Abdominal Breathing* ........ *14*

*Full Yogic Breath* ........ *15*

*Final Relaxation* ........ *15*

*Alternative relaxation* ........ *16*

Part IV: Supervision of participants post-exercise and education - 20 minutes ........ 18

*Proper Exercise* ........ *18*

*Proper Breathing* ........ *18*

*Proper Relaxation-Savasana* ........ *19*

*Proper Diet-Vegetarian – Part I* ........ *19*

*Proper Diet-Vegetarian – Part II* ........ *20*

*Positive Thinking and Meditation – Part I* ........ *20*

*Positive Thinking and Meditation – Part II* ........ *21*

*Making Positive Changes in Your Life* ........ *22*

**APPENDIX 1** ........ **23**

Policy for cardiac rehabilitation in Ealing ........ 23

**APPENDIX 2** ........ **32**

Ealing Cardiac Rehabilitation Health Education Talks ........ 32

*Drugs for heart disease and how they work* ........ *32*

*Managing Stress* ........ *34*

*Eating for a healthy heart* ........ *37*

*Exercise and the benefits for your heart* ........ *38*

*Risk factors and making lifestyle changes* ........ *41*

*The heart and how it works* ........ *44*

**REFERENCES** ........ **48**


DRAFT
For peer review only



For peer review only - http://bmjopen.bmj.com/site/about/guidelines.xhtml

Imperial College London

## Study Summary

| | |
|---|---|
| **TITLE** | Yoga And Cardiovascular Health Trial (YACHT) |
| **DESIGN** | Epidemiological |
| **AIMS** | To perform a mechanistic study to determine the acute and chronic effects of yoga on neuro-endocrine pathways, and downstream effects on CVD risk factors and subclinical outcomes. This will provide complimentary information to a larger clinical trial in India designed to determine effects of yoga on cardiovascular morbidity and mortality in acute coronary syndromes. |
| **POPULATION** | Indian Asians and Europeans, 40 in each ethnic group, (self-defined, verified by country of birth of all 4 grandparents). Aged between 35 to 80 years, male or female, without co-morbid disease and mobility limitations that would preclude participation in cardiac rehabilitation and our investigations. |
| **ELIGIBILITY** | Referred to cardiac rehabilitation programmes in West London post-angioplasty as treatment for an acute coronary syndrome. Able to understand English or Punjabi, Hindi or Gujarati, but in order to be able to follow the yoga class instructions the participant will need to have a basic command of the English language. |
| **DURATION** | Recruitment is planned for 1 year. |

DRAFT
For peer review only

Imperial College London

## Outline Study Design

Indian Asians and Europeans, 40 in each ethnic group, (self-defined, verified by country of birth of all 4 grandparents), referred to cardiac rehabilitation programmes in West London post-angioplasty as treatment for an acute coronary syndrome, will be invited to participate when being discharged from hospital.

Those who agree will be randomized to the yoga intervention plus their standard cardiac rehabilitation programme (usual care), or usual care alone.

In order to evaluate the chronic effects of yoga, baseline and 3 month measurements will be performed on all participants as described below:

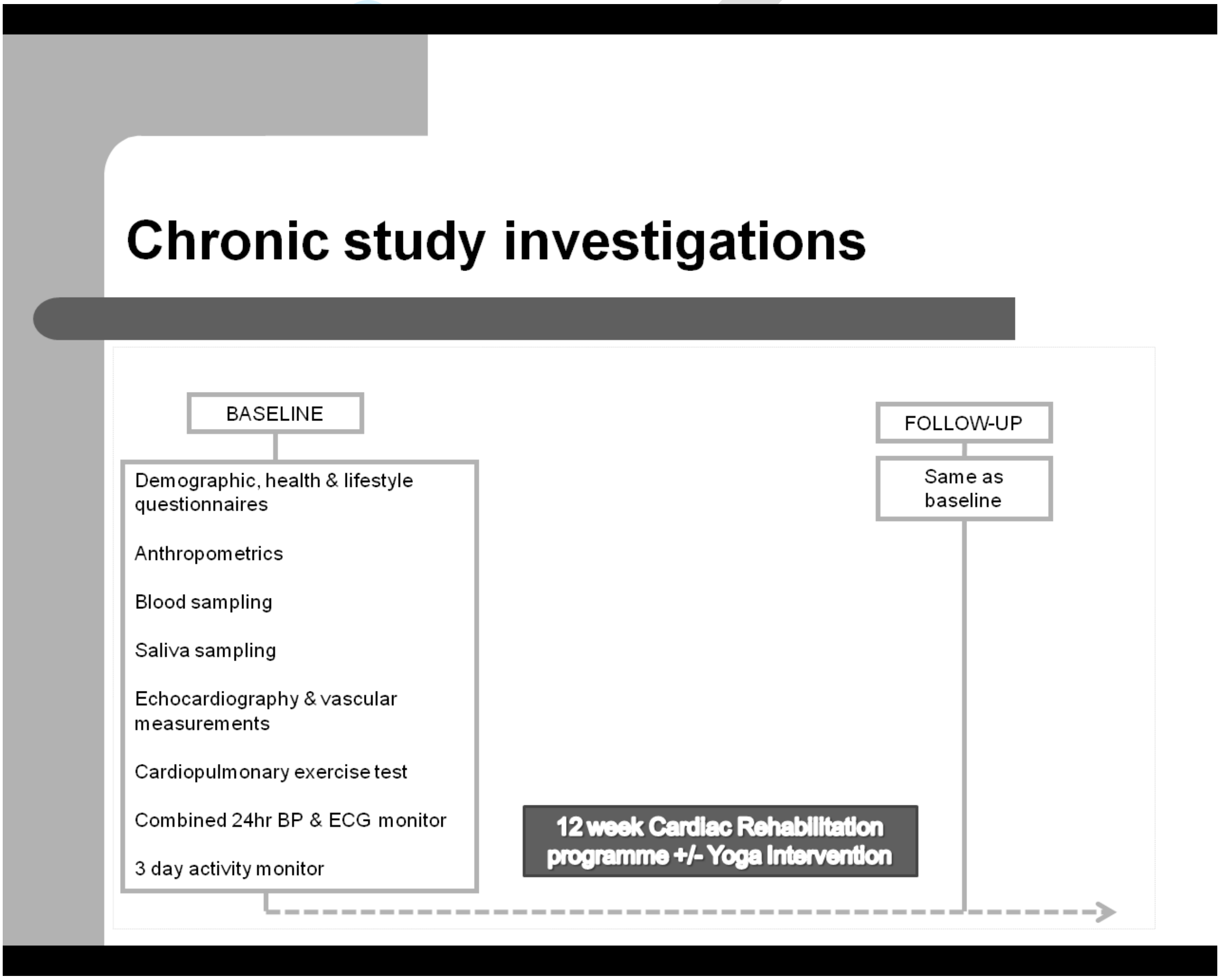




For peer review only - http://bmjopen.bmj.com/site/about/guidelines.xhtml

Imperial College London

# Yoga Summary

The yoga intervention will be delivered on a bi-weekly group session basis for 12 weeks alongside the cardiac rehabilitation programme. There will be 24 yoga classes in total, of which, each participant will be required to attend a minimum of 18. The yoga session will be designed and conducted by a teacher certified in yoga and cardiac rehabilitation, and will encompass physical fitness (yoga poses), stress reduction (breath control and meditation) and positive lifestyle changes (diet, smoking and alcohol).

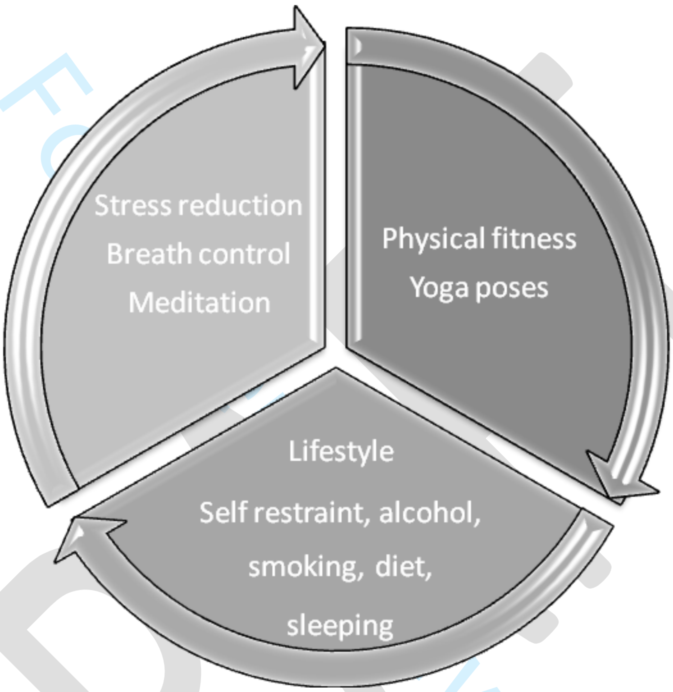


Each class will be approximately 1 hour and 15 minutes and in order to address the balance shown in the above diagram it will consist of the following parts:

- Yogic poses – approx 25 mins
- Breathing exercises and meditation – approx 25 mins
- Education and discussion – approx 25 mins

# Yoga Full Description

## *Part 1: Initial relaxation and warm-up – 10 minutes*

**Rationale:**


For peer review only - http://bmjopen.bmj.com/site/about/guidelines.xhtml

Imperial College London

The initial relaxation and warm-up should be 10 - 15 minutes in duration. Gradual increase in intensity triggers three mechanisms which increase coronary blood flow to match the increased myocardial demand. As a result the ischaemic threshold is extended and the risk of angina and the risk of arrhythmias is reduced.
Due to older average age of this group (compared with mainstream) a gradual progression of range of motion exercises is prescribed.
The short preparatory stretches are included to prepare the muscles for the range of movement involved in Asanas to reduce risk of injury and encourage good balance and alignments. Since incorporating a static stretching will result in fall in heart rate, stretches will be intersperse with some dynamic movement (walking on the spot) designed to maintain the elevated heart rate.

## Initial relaxation

1. Emphasise mood, breathing, and relaxation

2. Set the mood for the class. Explain what yoga is, how can help, how should be practiced. Concentration, breathing and relaxation in all yoga practice should be explained and repeated in each class. Put participants in a relaxed state emphasising body position, breathing and relaxation. Use voice to relax them.

3. Begin by finding a comfortable position standing position with your feet hip or shoulder-width apart. You can change positions any time during the relaxation exercises to make yourself more comfortable as needed. Start from breathing. Breathe in slowly and deeply through your nose. Continue to breathe slowly and gently. Allow your breathing to relax you.

The next relaxation exercise focuses on relaxing the muscles of your body.

1. Start with the large muscles of your legs. Tighten all the muscles of your legs. Hold it for a few moments and now relax. Let all the tension go.

2. Now focus on the muscles in your arms. Tighten your shoulders, upper arms, lower arms, and hands. Squeeze your hands into tight fists. Tense the muscles in your arms and hands as tightly as you can. Hold it for a few moments and release. Allow the muscles in your arms to relax completely.

3. Focus again on your breathing. Slow, even, regular breaths. Continue to breathe slowly and rhythmically.

4. Now focus on the muscles of your buttocks. Tighten these muscles as much as you can. Hold this tension and release. Relax your muscles.

5. Tighten the muscles of your back now. Feel your back tightening, pulling your shoulders back and tensing the muscles along your spine. Arch your back slightly as you tighten these muscles. Hold and relax. Let all the tension go. Feel your back comfortably relaxing into a good and healthy posture.

6. Turn your attention now to the muscles of your chest and stomach. Tighten and tense these muscles and release. Relax the muscles of your trunk.

7. Finally, tighten the muscles of your face. Scrunch your eyes shut tightly, wrinkle your nose, and tighten your cheeks and chin. Hold this tension in your face and relax. Release all the tension. Feel how relaxed your face is.

Notice all of the muscles in your body, notice how relaxed your muscles fee l. Allow any last bits of tension to drain away. Enjoy the relaxation you are experiencing. Notice your calm breathing and your relaxed muscles. Enjoy the relaxation for a few moments.

DRAFT
For peer review only

Imperial College London

When you are ready to return to your usual level of alertness and awareness, slowly begin to re-awaken your body. Wiggle your toes and fingers. Swing your arms gently. Shrug your shoulders.

## Warm-up

1. Now take a few steps on the spot and stand with your feet shoulder-width apart (set position), inhale and bend your knees and do mini-squat, exhale and come back to your set position. Repeat 4 times.

2. Take another few steps on the spot and come back to your set position. Inhale, roll your shoulders up, exhale bring them back and down. Repeat 4 times. During the last repetition hold your shoulders up for 4 to 8 seconds.

3. Take another few steps on the spot and come back to your set position. Inhale, roll your shoulders up, exhale bring them forward and down. Repeat 4 times. During the last repetition hold your shoulders up for 4 to 8 seconds.

4. Take another few steps on the spot and come back to your set position. Inhale, raise your arms to the side (shoulder height), exhale bring them down. Repeat 4 times. During the last repetition hold your arms up for 4 to 8 seconds.

5. Take another few steps on the spot and come back to your set position. Inhale, raise your arms to the front (shoulder height), exhale bring them down. Repeat 2 times. As above add heel raises at the same time if comfortable. Hold the last repetition for 4 to 8 seconds.

6. Take another few steps on the spot and come back to your set position. Inhale, raise your arms to the side (shoulder height), exhale and twist your trunk to the right. Inhale and come back to the centre, exhale and twist your trunk to the left. Repeat 2 times on each side. Hold the last repetition for 4 to 8 seconds.

7. Relax your arms and take another few steps on the spot and come back to set position. Inhale, raise your arms to the side (shoulder height) and look up. Exhale, bring your right arm crossover the chest to the left arm. Inhale, bring your right arm to the side. Exhale, bring your left arm crossover the chest to the right arm. Inhale, bring your left arm to the side. Repeat 2 times on each side. Hold the last repetition for 4 to 8 seconds.

8. Take another few steps on the spot and come back to your set position. Inhale and laterally bend your trunk to the right, exhale and come back to the centre.  . Inhale and laterally bend your trunk to the left, exhale and come back to the centre.  Repeat 2 times on each side. Hold the last repetition for 4 to 8 seconds.

DRAFT

For peer review only



For peer review only - http://bmjopen.bmj.com/site/about/guidelines.xhtml

Imperial College London

# *Part II: Asanas - 20 minutes*

**Rationale:**
Gentle progression from high to low positions is prescribed to avoid rapid changes of body positions in order to decrease the risk of arrhythmias or orthostatic hypotension in some individuals. Various 'chest opener' Asanas are prescribed to correct the round shouldered posture adopted by the population in response to discomfort in the area of the sterna incision.

## Standing poses (High positions)

### Mountain pose –Tad asana

1. Come to stand with the big toes touching.

2. Lift up all your toes and let them fan out, then drop them down creating a wide solid base. You can separate your heels slightly if your ankles are knocking together uncomfortably.

3. Bring your weight evenly onto all four corners of both feet.

4. Let the feet and the calves root down into the floor.

5. Engage the quadriceps and draw them upward, causing your knee caps to rise.

6. Rotate both thighs inward, creating a widening of the sit bones, and tuck your tailbone in between the sit bones.

7. Tone the belly, drawing it in slightly.

8. Widen the collar bones and make sure the shoulders are parallel to the pelvis.

9. The neck is long, the crown of the head rises toward the ceiling, and the shoulder blades slide down the back.

10. Hold for 5-10 breaths.

### Raised Hands Pose - Urdhva Hastasana

1. From Tadasana, bring your arms out to the side and up.

2. Press the palms together, keep the arms straight and take the gaze up toward your thumbs.

3. Slide the shoulder blades down the back.

4. Maintain your alignment.

5. Hold for 5-10 breaths.

### Awkward Chair Pose - Utkatasana

1. From  Tadasana bend the knees until the thighs are almost parallel to the floor.

2. Keep the butt low.


For peer review only - http://bmjopen.bmj.com/site/about/guidelines.xhtml

Imperial College London

3. Bring the arms up towards the ceiling.

4. Bring a slight back bend into the upper back.

5. Hold for 5-10 breaths.

**Beginners**: Work on bring the thighs closer and closer to parallel to the floor.

**Advanced**: Try this variation: Bring the hands into a prayer position at the heart. Twist to the right side, bringing the left elbow outside the right knee. Stay low in the pose and keep the knees pressing together. Come back to centre and then do the left side.

**Triangle Pose – Trikonasana**

1. From Tadasana take a big step backwards with your left leg.

2. Pivot on the ball of the left foot and drop the left heel onto the floor with the toes turned out about 45 degrees from the heel.

3. Bring the arms out to the side.

4. Slide the shoulder blades down the back.

5. Begin to reach the right arm forward, drawing the right thigh upwards and tucking the hip as you come forward.

6. Drop the right hand down to your shin or ankle, or if you are able, onto the floor inside or outside the right foot. Do whichever one feels most comfortable,

7. The left shoulder stacks on top of the right one as you open the chest reaching the left fingertips upwards while keeping the left shoulder rooted in the socket.

8. Take your gaze up towards the left fingertips.

9. Draw the right thigh muscle upwards, deepening the right hip crease.

10. Slightly bend the right knee.

12. Hold for 5-10 breaths

13. Repeat on the left side.

**Beginners**: Bring the right hand higher up on your leg or use a block on the floor to rest your hand on. It is more important to keep the right leg straight than to bring the right hand to the floor. Do not rest the hand directly on the knee, though, as this creates too much pressure on the knee.

**Advanced**: Line up the right heel with the arch of the left foot. For a variation, try dropping the left arm over the left ear so it comes parallel to the floor, while keeping the shoulder rooting into the socket.

**Tree Pose - Vrksasana**

1. Come to stand in Tadasana.

2. Feel your weight equally on all four corners of both feet.

DRAFT For peer review only

Imperial College London

3. Begin to shift the weight over to the right foot, lifting the left foot off the floor.

4. Bend the left knee, bringing the sole of the left foot high onto the inner right thigh.

5. Press the foot into the thigh and the thigh back into the foot.

6. Try not to let the right hip jut out. Keep both hips squared towards the front.

7. Focus on something that doesn't move to help you keep your balance.

8. Hold for 5-10 breaths.

9. Repeat the move while standing on the left foot.

**Beginners**: If you cannot bring the left foot high inside the right thigh, bring it lower on the right leg - but be careful to avoid placing the left foot directly on the right knee.

Use the wall for balance if necessary.

**Advanced**: Bring the arms up towards the ceiling with the palms touching. Open the arms out to side.

Try closing the eyes and see if you can stay balanced.

## Kneeling & Sitting poses (Medium positions)

### **Cat - Cow Stretch - Chakravakasana**

1. Start on all fours, bringing the wrists underneath the shoulders and the knees underneath the hips.

2. Think of the spine as a straight line connecting the shoulders to the hips. Try visualizing the line extending forward through the crown of the head and backwards through the tail bone.

3. Keep the neck the natural extension of the spine.

On an inhale:

1. Curl the toes under.

2. Drop the belly.

3. Take the gaze up toward the ceiling.

4. Let the movement in the spine start from the tailbone, so that that neck is the last part to move.

On the next exhale:

1. Release the tops of the feet to the floor.

2. Round the spine.

3. Drop the head.



For peer review only - http://bmjopen.bmj.com/site/about/guidelines.xhtml

DRAFT For peer review only

4. Take the gaze to the navel.

5. Repeat the Cat - Cow Stretch on each inhale and exhale, matching the movement to your own breath.

6. Continue for 5-10 breaths, moving the whole spine. After your final exhale, come back to a neutral spine.

## Hands and Knees Balance

1. Come on to all fours with the wrists underneath the shoulders and the knees underneath the hips.

2. Extend the right leg to the back of your mat and flex the foot.

3. Lift the right leg up to hip-level, keeping the hips squared towards the floor and the foot flexed.

4. Lift the left arm up to shoulder level.

5. Balance on the left knee and right hand, keeping the spine neutral and the neck long.

5. Stay 5-10 breaths before lowering the lifted hand and knee and doing the other side.

**Beginners**: Take care not to let the spine collapse while you are balancing.

**Advanced**: Bend the knee of the lifted leg. Reach around with the lifted arm and hold on to the inside of the lifted foot.

## Staff Pose - Dandasana

1. Sit with the legs outstretched straight in front.

2. Engage the thigh muscles and flex the feet. The heels may come up off the floor.

3. Make your spine long.

4. Stack the shoulders directly on top of the hips.

5. Hold for 5-10 breaths.

**Beginners**: Put padding under your sit bones, if necessary.

**Advanced**: This pose looks easy, but if you are really working the thighs, you can break a sweat.

## Seated Forward Bend - Paschimottanasana

1. From Dandasana bring the arms straight out to the sides and up over your head.

2. Inhale and draw the spine up long.

3. As you exhale, begin to come forward, hinging at the hips.

4. On each inhale, extend the spine, and on each exhale, come a bit farther into the forward bend.

5. Keep the neck at the natural extension of the spine.

6. Do not round the back.


For peer review only - http://bmjopen.bmj.com/site/about/guidelines.xhtml

7. Take hold of the ankles or shins, whichever you can reach.

8. Hold for 5-10 breaths.

**Beginners**: Put padding under the sit bones if necessary. Imagine the belly coming to rest on your thighs, rather than the nose coming to the knees - this will help you keep the spine long instead of curving over.

**Advanced**: If you can easily grab the soles of your feet, try taking a block in front of the feet and holding that instead.

## Crab pose- Catuspadapitham

**1.** From Dandasana, bend the knees bringing the feet flat on the floor hip width apart. Keep the arms behind your hips with the fingers pointed away from your body.

**2.** Lean back into the arms and slowly inhale and lift the hips up towards the ceiling. Make sure the toes and knees are pointing straight ahead. Look straight ahead, up at the ceiling or carefully drop the head back.

**3.** Press into the feet, squeezing the thighs and buttocks and engaging Mula Bandha to lift the hips high. Press into the hands and draw the shoulder blades towards each other to lift up high through the sternum.

**4.** Breathe and hold for 2-6 breaths.

**5.** To release: slowly exhale the hips back down to the floor.

**Beginners**: If there is pain or discomfort in the wrists, point the fingers in the opposite direction or make fists with the hands.

**Advanced**: Inhale one leg up towards the ceiling at a time, pressing out through the heel.

## Half Lord of the Fishes Pose (Half Spinal Twist) - Ardha Matsyendrasana

1. From Dandasana, bend your left knee and bring the sole of your left foot to the floor on the outside of the right thigh.

2. Bend the right knee, and tuck the right foot in near the left buttock.

3. Inhale and bring the right arm up near your right ear.

4. Exhale and twist the to the left, bringing the right elbow to the outside the of left knee and the left palm to the floor, just behind your sit bones.

5. Look out over the left shoulder, but don’t overturn the neck -- the twist originates in the belly, not the neck.

6. On each inhale, draw the spine long, and on each exhale, twist a little deeper.

7. Be sure to keep the sole of your left foot flat on the floor.

8. Hold for 5-10 breaths.

9. When you release the pose, take a slight counter twist to the opposite direction.

10. Release the legs and switch their position as you prepare to twist to the other side.


For peer review only - http://bmjopen.bmj.com/site/about/guidelines.xhtml

Imperial College London

**Beginners**: You may want to sit on some padding if you are uncomfortable. If you cannot bend it into the ideal position, you may also keep the right leg extended.

**Advanced**: Come into a bind with the arms. Thread the right arm back underneath the left knee. Reach the left arm behind your back, and clasp the left wrist with your right hand.

**Easy Pose - Sukhasana**

1. Arrange padding under your sit bones so that your hips come above your knees.

2. Come to sit in a comfortable, cross-legged position.

3. Bring one heel in towards your groin. The other foot may rest on the floor in front of you or you may bring it into your lap.

4. Root your seat down as your spine grows long. Stack the shoulders over the hips and slide the shoulder blades down your back. The crown of your head rises towards the ceiling.

**Neck Exercises**

1. In Sukhasana, with your back straight and your chest erect. Slowly bring your head forwards towards the chest to give the back of your neck a good stretch.

2. After a few breaths slowly lift your head and extend your neck back and bring your head to neutral position.

3. Lower your right ear close to your right shoulder, then repeat on the other side. Keep both shoulders level throughout. Repeat the exercise 5 times.

4. Turn your head to the right side. Contract the muscles on the right side of your neck and feel the stretch on the left side. Repeat on the opposite side. Repeat the exercise 5 times.

## Lying down poses (Low positions)

**Single Leg Lift**

1. Lie flat on your back with your legs together, arm next to your body, and palms face down.

2. Inhale and raise your left leg, keeping your knee straight, toes towards your head.

3. Exhale and lower your leg to the starting position.

4. Repeat up to 5 times on the each side.

**Head to Knee Raise**

1. Start from Single Leg Left Step 2.

2. With an exhalation, bend your left leg and clasp your hands around your knee.

3. With an inhalation, lift your head and try to bring your forehead against your left knee.

4. With an exhalation, lower your head, arms, and leg.

5. Repeat on the opposite side.


For peer review only - http://bmjopen.bmj.com/site/about/guidelines.xhtml
For peer review only DRAFT

Imperial College London

**Beginners**: Keep your head on the floor.

**Advanced**: Progress to Deep Stretch Single Leg Lift.

1. Start from Single Leg Lift Step 2.

2. With an exhalation, take hold of your leg with both hands, lift your back off the mat and try to bring your chest and head close to the raised leg.

## Happy Baby Pose - Ananda Balasana

1. Come to lie on the back.

2. Bend the knees into the chest.

3. Open the knees, bringing them towards the armpits.

4. Stack each ankle directly over the knee, so that the shins are perpendicular to the floor.

5. Flex the feet.

6. Hold the outer edges of the feet at you draw the knees towards the floor.

This pose is appropriate for both beginners and advanced students.

## Corpse Pose - Savasana

1. Come to lie down on the back with your arms and feet apart and your eyes closed.

2. Let the feet fall out to either side.

3. Turn the palms to face upwards.

4. Relax the whole body, including the face. Let the body feel heavy.

## *Part III: Cool Down: Breathing exercises and final relaxation - 20 minutes*

**Rationale:**
Twenty minutes Cool Down/Breathing/Relaxation period is prescribed to reduce risk of hypotension or arrhythmias and to allow the hart rate to return to pre-exercise rates.

**Deep Abdominal Breathing and Full Yogic Breath Practice-5 minutes**

### Deep Abdominal Breathing

1. In Corpse Pose place both hands on your abdomen with your fingers apart.

2. As you inhale, feel your abdomen and hands rising.

3. As you exhale, feel your abdomen and hands sinking.

4. Try to breathe rhythmically, with an inhalation lasting 3-5 seconds and exhalation of the same length.



For peer review only - http://bmjopen.bmj.com/site/about/guidelines.xhtml

Imperial College London

## Full Yogic Breath

1. In Corpse Pose place one hand on your chest and the other on your abdomen.

2. As you inhale, gradually expand the abdomen, then rise and open the rib cage, and finally lift the collar bones.

3. Begin the exhalation by relaxing the abdomen, then lower the rib cage, and finally slightly contract the abdomen to actively empty the lungs.

Alternate Nostril Breathing (Anuloma Viloma)- 5 minutes

Single Nostril Breathing

In Easy Pose, place your right hand in front of your face in Vishnu Mudra*. Close your right nostril with your thumb. Inhale for three seconds and exhale for six seconds through your left nostril. This is one round. Practice ten rounds. Repeat on the other nostril: close your left nostril with your ring finger, and inhale and exhale through your right nostril.

**Beginners**: Gradually increase the ratio of the inhalation to the exhalation lengthening to 4:8, 5:10 and 6:12.
**Advanced:** Progress to Simple Alternate Nostril Breathing

Simple Alternate Nostril Breathing

In Easy Pose close your right nostril with your thumb, inhale through the left nostril for three seconds, close your left nostril with your ring finger, open your right nostril and exhale through it for six seconds. Inhale through your right nostril for three seconds, then exhale through your left nostril for six seconds. Practice for ten rounds. Gradually increase the inhalation: exhalation ratio to 4:8, 5:10 and 6:12.

*Vishnu Mudra: Hold your right hand with the palm facing you and fold the first and second fingers into the palm. Try to keep your thumb and ring fingers straight.

## Final Relaxation

1. Inhale and lift your right leg a few inches off the mat. Tense your leg, then exhale and allow your leg to drop. Repeat with the left leg.

2. Inhale and lift both arms a few inches off the mat. Clench your fists, tense your arms, then exhale and allow your arms to drop to the mat.

3. Inhale and lift your hips and buttocks off the mat. Tense your buttocks and then exhale and release.

4. Inhale and lift your chest off the mat. Tense your shoulder blades, then exhale and release.

5. Inhale and pull your shoulders towards your ears. Exhale and release your shoulders.

6. Inhale and squeeze the muscles of your face tightly together. Exhale and release.

7. Inhale, open your mouth, stick your tongue out and look to your forehead. Exhale and release.

8. With an inhalation, slowly roll your head to one side; with an exhalation, roll it to the other side. End by bringing your head back to centre.


For peer review only - http://bmjopen.bmj.com/site/about/guidelines.xhtml

Imperial College London

Take a few slow rhythmic breaths using your abdomen, then follow this exercise in auto suggestion.

I'm relaxing my feet.....My feet are relaxed.....

I'm relaxing my ankles.....My ankles are relaxed.....

I'm relaxing my calves.....My calves are relaxed.....

I'm relaxing my knees.....My knees are relaxed.....

I'm relaxing my thighs.....My thighs are relaxed.....

I'm relaxing my hips and buttocks.....My hips and buttocks are relaxed.....

I'm relaxing my abdomen and chest.....My abdomen and chest are relaxed.....

I'm relaxing my lower and middle back.....My lower and middle back are relaxed.....

I'm relaxing my shoulders and neck.....My shoulders and neck are relaxed.....

I'm relaxing my hands and fingers.....My hands and fingers are relaxed.....

I'm relaxing my arms.....My arms are relaxed.....

I'm relaxing my mouth and eyes.....My mouth and eyes are relaxed.....

I'm relaxing my facial muscles and scalp.....My facial muscles and scalp are relaxed.....

I'm relaxing my internal organs: my kidneys, my livers, my intestines, my bladder, my pancreas, my stomach, my heart, my lungs and my brain.....My internal organs: my kidneys, my livers, my intestines, my bladder, my pancreas, my stomach, my heart, my lungs and my brain are relaxed.....

Continue abdominal breathing and relaxation. Visualise a calm lake, unruffled by waves. Picture the still water resting on your inner self, which is timeless and unchanging. Continue for a few more minutes.

Then take a few deep breaths, slowly move your legs and arms, and give your whole body a good stretch. Finally bring yourself slowly to sitting cross-legged position. Deeply inhale and exhale. Inhale and bring your hands into a prayer position and as you exhale bow your head thanking everyone for the practice.

## Alternative relaxation

In Savasana pose. For the next few moments, focus on calming your mind by focusing on your breathing. Allow you breathing to centre and relax you. Breathe in.... and out.

In..... out.....

In.... Out.....

Continue to breathe slowly and peacefully as you allow the tension to start to leave your body.

Release the areas of tension, feeling your muscles relax and become more comfortable with each breath.

Continue to let your breathing relax you....



For peer review only - http://bmjopen.bmj.com/site/about/guidelines.xhtml

Imperial College London

Breathe in....2...3...4.... hold....2.....3...... out...2...3....4...... 5

again....2.....3....4....hold....2....3.... out...2...3....4.... 5

Continue to breathe slowly, gently, comfortably.....

Let the rate of your breathing become gradually slower as your body relaxes.

Now begin to create a picture in your mind of a place where you can completely relax. Imagine what this place needs to be like in order for you to feel calm and relaxed.

Start with the physical layout of the place you are imagining..... where is this peaceful place? You might envision somewhere outdoors.... or indoors..... it may be a small place or large one..... create an image of this place.

(pause)

Now picture some more details about your peaceful place. Who is in this place? Are you alone? Or perhaps you are with someone else? Are there other people present? Animals? Birds? Imagine who is at your place, whether it is you only, or if you have company.

(pause)

Imagine even more detail about your surroundings. Focus now on the relaxing sounds around you in your peaceful place.

Now imagine any tastes and smells your place has to offer.

Imagine the sensations of touch... including the temperature, any breeze that may be present, the surface you are on.... imagine the details of this calming place in your mind.

Focus now on the sights of your place - colours, shapes.... objects.... plants..... water..... all of the beautiful things that make your place enjoyable.

To add further detail to this relaxing scene, imagine yourself there. What would you be doing in this calming place? Perhaps you are just sitting, enjoying this place, relaxing. Maybe you imagine walking around.... or doing any other variety of activities.

Picture yourself in this peaceful place. Imagine a feeling of calm..... of peace..... a place where you have no worries, cares, or concerns.... a place where you can simply rejuvenate, relax, and enjoy just being.

(pause)

Enjoy your peaceful place for a few moments more. Memorize the sights, sounds, and sensations around you. Know that you can return to this place in your mind whenever you need a break. You can take a mental vacation to allow yourself to relax and regroup before returning to your regular roles.

In these last few moments of relaxation, create a picture in your mind that you will return to the next time you need a quick relaxation break. Picture yourself in your peaceful place. This moment you are imagining now, you can picture again the next time you need to relax.

When you are ready to return to your day, file away the imaginary place in your mind, waiting for you the next time you need it.

Turn your attention back to the present. Notice your surroundings as your body and mind return to their usual level of alertness and wakefulness.


For peer review only - http://bmjopen.bmj.com/site/about/guidelines.xhtml
For peer review only
DRAFT

Imperial College London

Keep with you the feeling of calm from your peaceful place as you return to your everyday life.

## *Part IV: Supervision of participants post-exercise and education - 20 minutes*

**Rationale:**
Because of an increased risk of arrhythmia and hypotension following exercise, a period of 15-20 minutes supervision is adopted before participants go home. This time will be used for education sessions.

### *Proper Exercise*

*Yoga versus physical culture*

Aim:
- For the group to understand the importance of taking up physical activity in cardiac rehabilitation
- For the participants to understand that yoga improves not only flexibility but strength, balance and cardiovascular function

Brainstorm question: What are the differences between yoga and physical culture?

Explore the answers and highlight the following:

- Yoga regards the body as a vehicle for the soul in its journey towards perfection
- Yoga promotes gentle movement whereas physical culture emphasises violent muscle movements
- Muscle development does not necessarily mean a healthy body
- Health is a state wherein all organs function perfectly under intelligent control of mind
- Asanas are designed to develop not only the body but also broaden the mental faculties and spiritual capacities
- The body is as young as it is flexible, so yoga postures primary focus on the health of the spine, its strength and flexibility
- Asanas work on the internal machinery of the body, the glands and organs as well as the muscles
- Hand in hand with the practice of yoga postures we practise deep breathing and concentration of the mind

### *Proper Breathing*

*Yogic breathing*

Observe your breathing for a while. How would you describe your breath?

Ask participants to share their observation and then highlight the following:

- Yoga philosophy claims we are allotted a certain number of breaths per lifetime. How we choose to illustrate that then becomes our practice of longevity. Breathing is the first thing we do when we are born and the last thing we do when we die. Practice observing your breath as often as possible.
- Most people use only a fraction of their potential lung capacity when breathing



For peer review only - http://bmjopen.bmj.com/site/about/guidelines.xhtml

Imperial College London

- There are three types of breathing: clavicular, intercostal and deep abdominal.
- A full yogic breath combines all three types of breathing
- Yogic breathing exercises are called pranayama which means to control the prana-subtle energy. Pranayama begins by controlling the motion of the lungs, by which the prana is control.
- Yogic breathing exercises might be very useful in process of quitting smoking

## *Proper Relaxation-Savasana*

Brainstorm question: How do we relax?

Explore the answers and highlight the following:

- When the body and mind and the mind are constantly overworked, their natural efficiency diminishes
- Modern social life and entertainment make it difficult for people to relax by over stimulating the nervous system
- By learning to relax we learn to economise the energy produced by our body as well as regulate and balance the work of the body and mind
- In ordered to achieve perfect relaxation, three methods are used for yogis: physical, mental and spiritual relaxation
- The relaxation position is known as Savasana, the 'Corpse pose'

## *Proper Diet-Vegetarian – Part I*

*'You are what you eat'*

Discuss and highlight the following:

- Proper yogic diet is lactovegetarian one based on simple, natural and wholesome food
- According to yogic philosophy all of Nature, including our diet, is categorised into three qualities (Gunas): sattvic (pure), rajasic (overstimulating) and tamasic (putrified)
- Sattvic food increases vitality, energy, vigor, health and joy
- Food should be as fresh and natural as possible, preferably organically grown
- Sattvic food include:

  **Grains:** corn, barley, wheat, unpolished rice, oat, millet and quinoa.
  Grains supply necessary carbohydrates, the main source of energy for the body, and they also contain about half the amino acids that are needed to form protein.
  **Protein foods:** legumes, nuts and seeds

  **Fruits:** both fresh and dried, as well as pure fruit juices


For peer review only - http://bmjopen.bmj.com/site/about/guidelines.xhtml
DRAFT
For peer review only

**Vegetables:** they contain minerals, vitamins and fibre. There are best eaten raw or cooked as lightly as possible

**Herbs:** for seasoning and herbal tea

**Natural sweeteners:** honey, molasses, maple syrup, and apple juice concentrate. White sugar is best avoided in a healthy diet.

**Dairy products:** milk, butter, cheese and yogurt

## *Proper Diet-Vegetarian – Part II*

*Guidelines for healthy eating*

Recap from the previous 'Proper diet' session and then highlight the following:

- Always respect your food and maintain a peaceful attitude during meals
- Do not eat when you are angry
- Do not eat food that is too hot or too cold, as this will upset your stomach
- Do not force yourself to eat anything you do not like, but also do not only eat foods that you like the most
- Abandon too many mixtures or combination of foods as they are difficult to digest
- Try to refrain from drinking during meals as this will dilute the gastric juice
- Eat slowly and savour your food
- Eat moderately, do not overload your stomach
- Try to eat at fixed times and try to refrain from eating between meals
- Try not to eat large meals at night
- Take some lemon and honey in the morning for health and energy and to purify the blood
- Do not practise asanas immediately after eating, nor when you are hungry
- Try sitting in Vajra Asana (sitting on the heels with knees and feet together) for 10 minutes after a meal to assist digestion

## *Positive Thinking and Meditation – Part I*

*Practical approach to meditation*

Discuss and highlight the following:

- Before we can learn to meditate we have to be able to concentrate
- Concentration means attending fully to one thought or object for a substantial length of time


For peer review only - http://bmjopen.bmj.com/site/about/guidelines.xhtml

Imperial College London

- Concentration exercises energise the mind, boosting efficiency at work and in the other tasks, while building will-power and the ability to influence other people positively

**Exercise: Listen to a sound**
Now listen carefully to the ticking of a watch. When your mind wanders, bring it back to the sound. How long can you concentrate on that sound?
*Exercises to practice at home-leaflet to be given*

**Lose yourself in a book**
Read two or three pages of a book, giving them your full attention. Then test your concentration by stopping at the end of a page. How much do you remember of the story? Can you classify, group or compare the facts you have been reading about?

**Contemplate nature**
During the day, concentrate on the sky. Feel your mind expand as you reflect on its vast expanse. At night, concentrate on the moon or stars. By the sea, focus on waves. Or shift your gaze between objects near and far, such as a nearby tree and a distant mountain.

**Focus on a flower**
Sit comfortably with your eyes closed.  Imagine a garden with many flowers. Gradually, bring your attention to a single flower. Visualise its colour and explore its other qualities, such as texture, shape, and scent. Concentrate on the flower's qualities for as long as possible.

## *Positive Thinking and Meditation – Part II*

*Practical approach to meditation*

Ask participants if they had chance to practice any of the concentration exercises then discuss and highlight:

- Meditation is a state of relaxed awareness
- The more care and attention you give to your preparation for meditation, the more positive the results will be
- Get the atmosphere right for meditation:

  **Place**: It is best to separate one portion of a room to use for your practice. Keep it clean and tidy, and place a candle or spiritually uplifting picture there. Burning incense can also help to create a meditative mood.

  **Time**: The best times for meditation are at dawn and dusk. Alternatively, find a time when you are free from daily activities and your mind can be calm.

  **Habit**: Practise every day at the same time. As your subconscious mind gets accustomed to the regularity, you will find it easier to settle and focus.

  **Sitting position**: Sit on the floor to meditate, in position that you can maintain comfortably, keeping your spine and neck straight but not tense. A simple, crossed-legged pose makes a firm base. Sitting on the cushion helps the thighs to relax and bring brings knees closer to the ground.

  If you cannot sit on the floor easily, sit on a comfortable chair with your ankles crossed.

DRAFT
For peer review only

Imperial College London

**Breathing**: Once you are sitting comfortably, relax your body as much as possible. Broaden your chest and lift your rib cage to encourage abdominal breathing. Then inhale and exhale rhythmically for about 3 seconds each, gradually slowing your breath down.

## *Making Positive Changes in Your Life*

Topic to reflect: Think about one change you would like to make (if any) to make your lifestyle healthier.

Ask if anyone would like to share their idea then suggest the following changes to make within first two months of practising yoga.

- **Proper exercise:** Try to practise asanas regularly
- **Proper breathing:** Practice deep abdominal breathing
- **Proper relaxation:** Learn Corpse pose and try to relax for 15 minutes daily
- **Reduce negative dietary habits:** Cut down or eliminate meat and cut down on fried food
- **Reinforcing positive dietary habits:** Drink 4 to 5 glasses of water and eat one raw salad daily
- **Eradicating negative habits:** If you smoke replace it with abdominal breathing
- **Concentration exercises:** Practice listening and hearing what others are saying
- **Positive thinking:** Refrain from using abusive language and try to spend time with people who have a positive outlook on life
- **Meditation:** Sit silently for at least 20 minutes daily with the mind focused on breath
- **Study:** Read something of inspiration daily

DRAFT


For peer review only - http://bmjopen.bmj.com/site/about/guidelines.xhtml

Imperial College London

# Appendix 1

## *Policy for cardiac rehabilitation in Ealing*

### INTRODUCTION

Cardiac disease is the leading cause of death in United Kingdom and is the leading cause of hospitalisation for both men and women. Cardiac rehabilitation programmes are recognised as a way to enhance recovery following acute cardiac events and encourage behaviour aimed at the secondary prevention of coronary artery disease. The key elements of cardiac rehabilitation are contained in the definition produced by the Scottish Intercollegiate Guidelines Network (SIGN): Cardiac rehabilitation is the process by which patients with cardiac disease, in partnership with a multidisciplinary team of health professionals, are encouraged and supported to achieve and maintain optimal physical and psychological health.

Cardiac rehabilitation is defined by the World Health Organisation as:
*".. the sum of activities required to influence favourably the underlying cause of the disease, as well as the best possible, physical, mental and social conditions, so that they (people) may, by their own efforts preserve or resume when lost, as normal place as possible in the community. Rehabilitation cannot be regarded as an isolated form or stage of therapy but must be integrated within secondary prevention services of which it forms only one facet".*

The provision of skilled help, support and supervision that is tailored to individual patients can: a) help people understand their illness and its treatment; b) provide psychological and emotional support; c) improve people's success in making beneficial lifestyle changes; and d) help people make the transition back to a full and as normal life as possible. (NSF: cardiac rehabilitation, 2007)

The 2007 NICE guidelines on "secondary prevention for patients following a myocardial infarction" state that cardiac rehabilitation should be equally accessible and relevant to all patients after an MI, particularly people from groups that are less likely to access this service. These include people from black and minority ethnic groups, older people, people from lower socioeconomic groups, women, people from rural communities and people with mental and physical health co morbidities.

The British Association of Cardiac Rehabilitation (BACR) standards 2007 defined the core components of cardiac rehabilitation as lifestyle (physical activity and exercise, diet and weight management, smoking cessation), education, risk factor management, psychosocial, cardio protective drug therapy and implantable devices, and long-term management strategies (4).

Four phases of cardiac rehabilitation were defined by the BACR and endorsed by the National Service Framework (2007) for CHD in England and Wales and SIGN for Scotland (2002). Each phase represents a different component of the journey of care. Phase 1 is generally concerned with the in-patient episode with Phases 2-4 following the patient from early discharge to long-term maintenance.

According to the NSF goal, every hospital should ensure that:
more than 85% of people discharged from hospital with a primary diagnosis of acute myocardial infarction or after coronary revascularisation are offered cardiac rehabilitation and one year after discharge at least 50% of people are non-smokers, exercise regularly and have a BMI <30 kg/m$^2$; these should be demonstrated by clinical audit data no more than 12 months old. Trusts should agree, implement and audit a detailed plan and protocol for identifying, treating and following up their patients who may benefit from cardiac rehabilitation.

### THE CARDIAC REHABILIATION PROGRAMME IN EALING HOSPITAL NHS TRUST

The aim of the comprehensive cardiac rehabilitation programme is to reduce the risk of subsequent cardiac problems and to promote the return to a full and normal life. The provision of a cardiac rehabilitation service for all eligible patients is clearly desirable for health and economic reasons.


For peer review only - http://bmjopen.bmj.com/site/about/guidelines.xhtml

Imperial College London

Comprehensive help with lifestyle modification involving education and psychological input as well as exercise training can reduce mortality by 20-25% over 3 years. ( Oldridge et al 1988; O'Connor et al 1989)

**1. TARGET CLIENT GROUPS**

Patients admitted with or who have undergone the following will be eligible for the programme.

- NSTEMI
- STEMI
- Acute Coronary Syndrome
- Revascularisation
- CABG
- Valve surgery
- Heart Failure

All the above patients admitted to Ealing Hospitals will be offered a choice as to where their cardiac rehabilitation will take place.

**2. IDENTIFYING PATIENTS**

- Patients are identified through CCU/ITU, cardiology and general medical wards, cardiology out patients, and from waiting lists for revascularisation procedures.
- Referrals are accepted from other acute trusts (using North West London Cardiac Rehabilitation referral form); Ealing Hospital cardiac catheter laboratory, as well as the community referrals from GP's, Practice Nurses, Community Specialist Clinics.

**3. PHASE 1** (Before discharge from hospital)

Where possible the Cardiac Rehabilitation Specialist Nurse will visit the patient and his / her family during the hospital stay. The following will be carried out during this phase:
• assessment of physical, psychological and social needs for cardiac rehabilitation
• negotiation of a written individual plan for meeting these identified needs
• initial advice on lifestyle e.g. smoking cessation, physical activity (including sexual activity), diet, alcohol consumption, driving and employment
• review of prescription of effective medication and education about its use, benefits and harms
• involvement of family members and/or relevant informal carer(s)
• provision of information about cardiac support groups
• provision of locally relevant written information about cardiac rehabilitation

It is important to establish rapport and therapeutic relationship with every patient and involve family or/and carers from this early stage. This will increase the likelihood or patient's participation in consecutive phases of cardiac rehabilitation programme and reduce a risk of DNA incidences.

The "Edinburgh Heart Manual" for education, exercise and stress management components can be given to eligible patients at this stage. Social needs and preferences of patients will be identified and taken into account for a purpose of structuring of individually tailored cardiac rehabilitation programmes.

Guidelines for Phase 1 Cardiac Rehabilitation Service will be followed ( see Appendix 1\)

BACR Guidelines for Secondary Prevention will be followed. They are:

- Risk factors of each patient should be identified and managed accordingly.
- All patients who smoke should be offered structured anti-smoking advice and, if necessary, specific

For peer review only


For peer review only - http://bmjopen.bmj.com/site/about/guidelines.xhtml

Imperial College London

treatment.

- All patients after acute myocardial infarction or coronary revascularisation should be treated, if necessary, with lipid lowering therapy (diet control, statin therapy, lifestyle changes to include regular exercises), antiplatelet therapy (with aspirin, dipyridamole or clopidogrel), beta blockers, ACE inhibitors, other secondary prevention measures (better control of diabetes, hypertension, body mass index (BMI)).
- All patients with heart failure should be given advice on fluid balance (daily weights, fluid intake, diuretic dosages), salt restriction, avoidance of ethanol consumption and smoking, influenza vaccination, and considered for therapy with ACE inhibitors, beta blockers, spironolactone, angiotensin receptor blockers for prognostic benefits.
- Patients with other conditions should receive appropriate advice and treatment as secondary prevention of their specific cardiac conditions (e.g. avoidance of caffeine and treatment with beta blockers in patients with cardiac arrhythmia).

**4. PHASE 2** (Early post-discharge period)

During early post discharge period, support to patients can be provided by home visiting where appropriate, telephone contact and by supervised use of the Heart Manual.
Patient will be sent an invite letter for the first outpatient appointment in cardiac rehabilitation clinic of Ealing Hospital within 2 -3 weeks after discharge from hospital. The time tables with dates of currently run educational sessions will be included with an invite letter.( see Appendix 2). Where possible, patients will be asked to have their blood tests done in GP practices prior to appointment with cardiac rehabilitation specialist nurse. Patients will also be asked to bring their medication list and any outpatient appointments' letters with them.
During the consultation in OPD clinic, the individual needs, expectations of cardiac rehabilitation programme and wishes will be explored. The suitability for exercise programme ( a component of comprehensive cardiac rehabilitation programme) can also be assessed at this point.

Following will be carried out:

- Provision of general advice about the cardiac condition(s) and complications that the patient has, including risk factor management, medication (what they are for, adjustment of doses and potential adverse reactions), presentation of further events and what actions to take; symptom control advise
- Patients' misconceptions and undue fears or anxieties will be identified and addressed
- Patients will be advised on the stages towards resuming normal life (e.g. physical activity levels, sexual function, driving, flight, return to work, weight control, fluid balance, alcohol consumption).
- Advice will be provided to patients to address vocational, social, cultural, educational needs, and referral for occupational therapy assessment and management.
- Measurement of patient's body weight, calculation of body mass index (BMI) and central obesity (girth measurements)
- Review of fasting lipid profile +- advice/ appropriate referrals to GP/lipid clinic.
- Review of fasting blood glucose +- advice/ referral to diabetic specialist nurse/GP
- Dietary habit assessment and advice on healthy eating
- Blood Pressure will be measured and heart rate record
- If known to have diabetes a urine sample for microalbuminurea will be collected.
- Assessment of wounds and advice as necessary (post surgery patients)
- HADS +-quality of life (QoL) assessments will be carried out to estimate patients' health perceptions and to help detect patients with inappropriate levels of anxiety or depression, a small proportion of whom may need referral for specialist evaluation and treatment
- Advice how to stop smoking- for those who smoke+- referral to specialist services
- Review involvement with cardiac support groups
- Offer resuscitation training for family members
- Encouraging patients' immediate family members to engage in health improvement and lifestyle modification

"Client Feedback and Goal Planning Form" (see Appendix 3), as well as "Agreed Action Plan" (see Appendix 4) forms will be given to those who were not seen at Ealing Hospital during Phase 1. Patients will be encouraged to



For peer review only - http://bmjopen.bmj.com/site/about/guidelines.xhtml

participate in joint (with nurse specialist) care planning, goal setting, time-allocation for improvement and exploration of barriers to achievement of desirable results. This process is very important for achievement of lifestyle modification and behavioural change.
Patients will be encouraged to bring those forms to meetings with cardiac rehabilitation nurse, or/and to educational, or/and exercise programmes, so appropriate to the area of concern specialist will be able to answer the questions and give patient-tailored advice and recommendations.
Patients will be also given comprehensive information regarding diagnosis, procedures, practical advice and risk factor modification in written form. Patients will be issued with a wallet sized card that allows each patient to keep a record of his or her risk factors, including blood pressure, cholesterol and glucose, lifestyle modifications, dates of procedures and current medication.

Patients and their partners will be invited to enter an 6-12 week health promotion programme where patients will receive 1) on-going risk factor monitoring/ advice/support, 2) exercise sessions in a gym, led by cardiac physiologist or home-based exercise programme, 3) health education lectures (led by cardiac rehabilitation sister; pharmacist; dietician; clinical psychologist; cardiac physiologist), 4) relaxation sessions, 5) guidance and supervised use of “Edinburgh Heart Manual”.

Patients will be asked about their preferences on exercise programme, whether they would like to join exercise programme run in local Gym(s) or to have home based exercise programme ( e.g. using “Edinburgh Heart Manual” as guideline and/or using exercise plan prescribed by cardiac physiologist and/or using pedometers, etc).
Patients will be given information on forthcoming dates/ topics/ venues of educational programme. Patients themselves and their family members or carers will be encouraged to come to educational sessions together.
For patients whose English is limited, the interpreter services will be provided where possible. Patients will be encouraged to bring their English speaking relatives for a consultation. The information about each individual condition/treatment/recommendations can be ordered for patient from British Heart Foundation in a form of audio tape/video-tape/pocket size leaflets, etc
Those patients who will not respond to invitation letter to attend an OPD clinic will be contacted via a telephone. Rehabilitation staff will try to address the issues which might impede patient's decision about participation in a programme using individual approach. However, If a patient will state clearly that he/ she does not wish to participate, then patient's GP will be informed and patient will be discharged from CR service.

**4.1.Referrals:**

- Weight management and diet advice: All patients regardless of their cardiac condition will be referred to cardiac specialist dietician for cardio-protective diet advice and weight management programme if appropriate.
- Diabetes: Newly diagnosed and those patients with diabetes who are not well controlled will be referred to the community diabetes team.
- Erectile Dysfunction: patients experiencing sexual problems can be referred to the ED clinic at GSTT.
- Psychological Problems: Rehabilitation staff will do their best to identify and address cardiac misconceptions in patients with CHD in order to reduce possibility of anxiety or/and depression. Hospital anxiety and Depression Scale (HADS) will be used. Screening will take place at discharge (where possible), 6-12 weeks post MI or following a decision on surgical intervention; can be repeated every 3 months if necessary. Psychological interventions of cardiac rehabilitation programme, such as stress management, relaxation, goal setting, taking part in group exercise and education can relieve anxiety and mild depression. Patients who score persistently above 11 on the HAD scale can be considered for referral to a clinical psychologist for assessment/ interventions.

- **Health Education Talks** (please see appendix 2 for the current health education presentations)

Discussions will be given by a health care professional with specialist knowledge of the subject. This is an information giving session to increase patients knowledge.

The group discussion allows patients to explore the information given and how best to apply it to themselves and their families. These sessions are intentionally informal and encourage patients to recognise their own risk factors and develop strategies for change. The following topics are to be covered by the workshops:

1. Drugs for heart disease and how they work (presented by pharmacist)


For peer review only - http://bmjopen.bmj.com/site/about/guidelines.xhtml

Imperial College London

2. Managing Stress (by clinical psychologist)
3. Eating for a healthy heart (by cardiac specialist dietician)
4. Exercise and the benefits for your heart (cardiac exercise physiologist)
5. Risk factors and making lifestyle changes (by cardiac rehabilitation sister)
6. The heart and how it works (by cardiac rehabilitation sister)

**5. PHASE 3 (**Four weeks after an acute cardiac event / 4-6 weeks post surgery)

Structured exercise as a therapeutic intervention is central to cardiac rehabilitation. Exercise training should form a core element of cardiac rehabilitation programmes (SIGN,2002).

At this stage, patients and their family members/carers should be aware of all the benefits of the physical exercise programme and should be committed to participate. Most patients will benefit from and will be encouraged to undertake at least low to moderate intensity exercise. However, patients with clinically unstable cardiac disease or limiting co-morbid illness will be excluded from exercise training. People whose potential to exercise is limited may have much to gain from the non-exercise components of cardiac rehabilitation.

**5.1. Contraindications to Exercise**

- Unstable Angina
- Unstable Ischemia
- Active pericarditis or myocarditis
- Hypertrophic obstructive cardiomyopathy
- SBP >180 mmHg or DBP >100 mmHg
- BP drop 20 mmHg during incremental exercise
- Resting/uncontrolled tachycardia >100
- Severe and symptomatic aortic stenosis
- Uncontrolled atrial or ventricular arrhythmias
- Severe pulmonary hypertension
- Heart failure that is not compensated
- Recent embolism
- Thrombophlebitis
- Unstable diabetes
- 30 AV block (without pacemaker)
- Febrile illness

**5.2. Exercise sessions.**

- The exercise sessions are held twice a week for 1 hour in St.Bernard's and Southall sport centres each week and patients are encouraged to attend between 8-12 sessions.
- Those patients who wish to participate in the exercise programme need to sign a consent form
- All patients need to attend an initial screening appointment and perform a sub maximal functional capacity test prior to attending the classes.
- Patients will be risk stratified into low, medium and high risk categories as defined by the American Association of Cardiovascular & Pulmonary Rehabilitation (AACVPR) as recommended by American College of Sports Medicine**.** (Appendix 5);"low risk" patients will be enrolled to attend a community sport centre, "moderate/ high risk" patients will be invited to participate in an exercise programme, based in a gym which in located in close proximity to Ealing Hospital.
- Patients, who will not want to attend formal taught sessions will be offered home-based exercise plan.

**Assessment before Exercise Classes:**

- Prior to participation in exercise training patients will undertake a submaximal functional capacity test (e.g. the 6 minutes walk test or shuttle walk test). This will usually be carried out by cardiac physiologist during a separate appointment.
- Prior to submaximal testing of functional capacity a pre-screening checklist will be completed to ensure suitability, an end point of 80% HR max will be determined (adjusted as appropriate for high risk patients) and a rating of perceived exertion of 15, using the Borg Scale category ratio 6-20 scale (Appendix 4). Prior to participation in the exercise test the patient will be familiarized to the Borg scale as below


For peer review only - http://bmjopen.bmj.com/site/about/guidelines.xhtml

Imperial College London

- Target heart rates for the exercise classes will be set to between 60-75% of the maximal heart rate minus 30 if on Beta Blockers. This range can be adjusted based on risk stratification. The range will be written in patient's exercise plan.

**Before the Class Begins**

- Brief discussion with patients about their progress, home exercises, changes, concerns
- BP and pre-exercise heart rate will be recorded
- Blood glucose levels checked for diabetic patients
- Equipment set up in advance
- Those patients who will complain of feeling generally unwell or become symptomatic or clinically unstable can be excluded from a session for a day. Depending on a condition, the symptoms will be treated with existing medications; patient will be either accompanied to A&E ( if severely unwell) or referred to GP.

**The Exercise Components.**
All sessions should include:

**Warm-Up (15 minutes minimum):** The warm-up period will include graduated low intensity aerobic exercise and short dynamic stretches to increase myocardial blood supply, soft tissue flexibility and mobilize joints.
**Circuit ( 20-30 minutes):** All patients will participate in a progressive exercise training programme, which is modified to meet individual need.
**Cool Down (10 minutes):** This will include graduated low intensity exercise and muscle stretching. Once complete HR will be rechecked and recorded (aim to be within 10 beats of pre-exercise rate)
**Relaxation (15 minutes minimum):** Following the exercise class, patients should be supervised for a 15 minute period.

**Health and Safety Requirements.**

- Each patient will be risk stratified as described above.
- Exercise will be delivered by experienced staff with training in exercise physiology and prescription and an understanding of the specific needs of cardiac patients in relation to exercise.
- 3 members of staff where possible - minimum ratio 1:5. This includes visitors. However priority for exercise is given to patients and if the numbers exceed the safety requirements, visitors will not be able to join in on that occasion.
- Cardiac physiologist to be trained BLS skills (minimum), cardiac rehabilitation nurse-to be present at each session and to be trained at ILS level (minimum)
- Resuscitation equipment available in the gym for the duration of the class.
- All visitors who wish to join in the exercise class need to complete a ParQ
- Venue must be suitable i.e. adequate space, temperature (65-72F,18-23C),
- Drinking water should be available.
- Immediate access to a telephone.
- Annual environmental risk assessment

**Monitoring patients**

- Heart rate monitors to be worn during a patient's first session.
- Heart rates recorded at the beginning of each class, during the class and after the cool down.
- Borg scale of perceived exertion will be recorded during exercise.

**Patients with diabetes**

- Record blood glucose level before the start of the exercise
- Avoid exercise if glucose is over 16mmol/L.
- Avoid exercise if glucose is under 6 and no snack is available prior to exercise
- If glucose >13 and <16 then do warm up and retest – level should fall. If remains >13 and rising should not continue exercise until their status has been stabilised.
- Those taking insulin should avoid injecting into subcutaneous tissue of thigh i.e. avoid sites near to exercising muscle groups.
- Avoid exercise during peak insulin times.


For peer review only - http://bmjopen.bmj.com/site/about/guidelines.xhtml

**Medical Emergencies**

- Nurse to stay with patient
- Exercise specialist to ensure safety of other patients
- Third person to call for help (999 and/or cardiologist)

**DNA Policy**

Patients will be informed about current DNA policy and their obligation to notify a cardiac rehabilitation nurse or cardiac physiologist if they have to miss a session. If a patient does not attend two consecutive classes, contact will be initiated by a member of the Cardiac Rehabilitation team and if no response is received then the patient is discharged from the programme and a letter sent to patient's GP as well as patient.

**End of Programme.**

On completion of the programme patient is given a Certificate stating patients achievements ( see Appendix 6) and a re-screening appointment is made in 2-4 weeks. Patient's GP will receive a letter from Cardiac Rehabilitation Team with all relevant information.

### 5.3. Home-Based Programmes

Those patients who would prefer a home programme or are unable to attend the group sessions will be assessed by the cardiac physiologist and given a suitable physical activity programme. Progress will be monitored regularly and risk factor management will continue as required. This may involve the patient attending regular appointments with the cardiac nurse for blood pressure/heart rate/ blood results monitoring/relevant support and advice. The patient is offered the opportunity to attend the health education talks where possible.

## 6. RE-SCREENING OF PATIENTS

On completion of the health promotion sessions all patients and their families are invited back to the Cardiac Rehabilitation OPD clinic where they will be reassessed as follows:

- Cardiac Risks will be assessed again and progress recorded
- Blood pressure, heart rate, lipids and glucose levels are repeated and recorded
- Those with diabetes will have their HbA1c checked
- Current medications therapy is reviewed
- HAD and QOL is repeated
- 6 MWT or Shuttle walk test is repeated Diet is reassessed and long term recommendations made
- BMI and girth measurement is checked again and recorded

Patients with stable coronary disease will be encouraged to continue regular moderate intensity aerobic exercises. The relevant information about the exercises, stretching techniques, relaxation exercises, and all available sport/leisure centres in the area will be given to patients on discharge. Information about local yoga/dancing/swimming/golf classes, etc. will be available on request. Individual approach will apply, hence if someone will prefer to carry on home based exercises he/she will be supported in their decision. Others, who prefer formal class based cardiac exercise programmes can be referred to the Phase 4 exercise sessions held in St.Bernard and Southall sport centres. The exercise sessions are lead by BACR trained exercise physiologists.

## 7. PHASE 4 (Long-term maintenance of changed behaviour)

Long term follow-up in primary care will be arranged.
Involvement with local cardiac support groups or groups of interest (e.g. gardening, cooking, walking, cycling, etc.) will be offered.
Referral to specialist cardiac, behavioural (e.g. exercise, smoking cessation) or psychological services will be made, if clinically indicated.

## 8. ANNUAL REVIEW


For peer review only - http://bmjopen.bmj.com/site/about/guidelines.xhtml

Imperial College London

All patients are invited to attend a follow-up appointment one year after completion of the programme. At this appointment fasting lipids, glucose, and, if appropriate, and HbA1C are measured. Blood pressure is checked twice and anthropometry is recorded. A physical activity and brief dietary assessment are carried out. A summary is sent to the GP and patient with further recommendations if appropriate.

**9. INTERGRATING CARE BETWEEN SECONDARY & PRIMARY CARE.**

A seamless transition between hospital provision of cardiac rehabilitation and the continuing support provided by primary care practitioners requires good communication between all involved in the care of patients with CHD. The primary care team, with detailed knowledge of an individual's social and medical background, includes professionals who are likely to be aware of the implications of CHD for both the individual and their family. Accurate information shared between the various members of multidisciplinary teams across both primary and secondary care will enable the best possible care to be given to the patient.

**10. AUDIT & EVALUATION ( TO AGREE ON EVALUATION OF WORK)**

The Cardiac Rehabilitation service will carry out clinical audit using routinely collected data. Long term goals can be monitored by observing changes over time in incidence and mortality from CHD.

Data will be collected onto the CR database and will also be exported to the national database annually as required for a purpose of NACR. On completion of the programme, patients will be asked to fill in a satisfaction questionnaire.

Standards that we need to follow:

The service should be referred to in the HImP and reflected in long term service agreements.

A clear description of the district cardiac rehabilitation programme should be available to the public, to service providers and to commissioners and should be cited in the HImP.
This description should include details of:

• the patients to be offered cardiac rehabilitation
• staffing (including details of the skills and training required)
• the location and timetable of service provision
• audit criteria
• investment and resources.

Whatever the detail of local rehabilitation services, records should be kept so that the service can be audited against nationally recommended guidelines. This should include information about ethnicity so that it is possible to monitor equity of access. Audit will be easier to undertake if data is stored electronically in a way that allows ready analysis.
National Service Framework – Coronary Heart Disease

**Clinical audit**

Clinical audit – the systematic assessment of the quality of care – is an essential component of modern, high quality health care. It will also be an essential component of effective clinical governance embracing all health professionals.
Trusts should work with their local PCTs and their constituent practices to undertake clinical audit that allows them to review annually the items listed in **bold** below. They may also wish to review the other items when it becomes possible to collect these data.

1) number and % of patients discharged from hospital after coronary revascularisation OR with a primary diagnosis of AMI with documentation of arrangements for cardiac rehabilitation in discharge communication to GP (by Trust and PCG/PCT and by sex,


DRAFT
For peer review only
For peer review only - http://bmjopen.bmj.com/site/about/guidelines.xhtml

Imperial College London

age 35-74iii years, and ethnic group)

2) number and % of patients discharged from hospital with a primary diagnosis of CHD recruited to a cardiac rehabilitation programme by Trust and PCG/PCT and by sex, age 35-74iii years, and ethnic group

3) total number and % of those recruited to cardiac rehabilitation who have an individualised plan for rehabilitation and secondary prevention before discharge from hospital
4) total number and % of those recruited to cardiac rehabilitation who, one year after discharge, report:
• regular physical activity of at least 30 minutes duration on average 5 times a week
• not smoking
• BMI < 30 kg/m2.

(NB. PCTs and rehabilitation services may wish to collaborate in the collection, analysis and interpretation of their audit data to avoid duplication of effort and to gain a more complete picture of the quality of rehabilitation and secondary prevention services.)

This Policy will be reviewed and updated if necessary on annual bases.

**REFERENCES**

1. Scottish Intercollegiate Guidelines Network (2002) Cardiac Rehabilitation. A national clinical guideline. SIGN guideline 57. http://tinyurl.com/27g33c

2. National Institute for Health and Clinical Excellence (2007) MI: secondary prevention. Clinical Guideline 48. May. http://tinyurl.com/38tom3

3. British Association of Cardiac Rehabilitation (2007) Standards and Core Components for Cardiac Rehabilitation. http://tinyurl.com/3ydagw

4. Department of Health (2000) Coronary Heart Disease: National Service Frameworks. HMSO: London

5. World Health Organisation (1993) Needs and action priorities in cardiac rehabilitation and secondary prevention in patients with CHD. Geneva: WHO regional office for Europe

6. American College of Sports Medicine (1991) Guidelines for exercise testing and prescription, 4th edn. Lea and Febinger, Philadelphia



For peer review only - http://bmjopen.bmj.com/site/about/guidelines.xhtml

# Appendix 2

## *Ealing Cardiac Rehabilitation Health Education Talks*

*Drugs for heart disease and how they work*

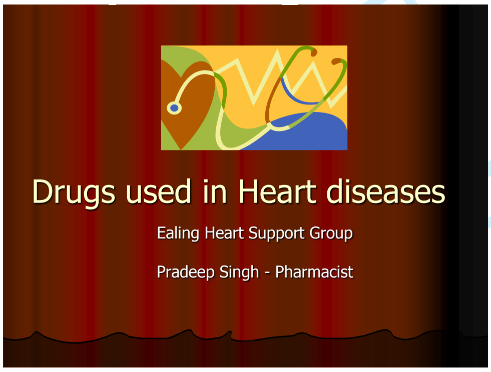


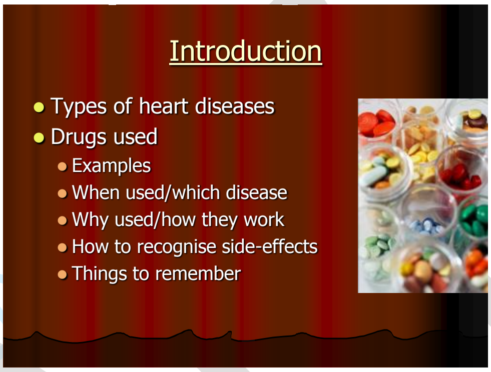


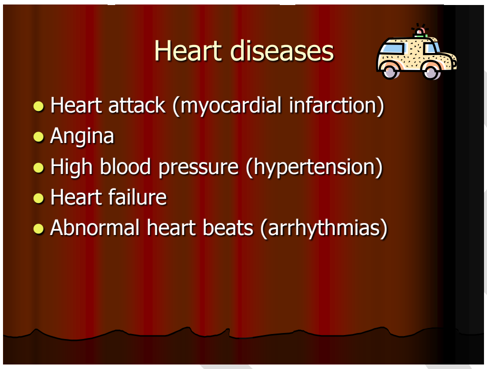


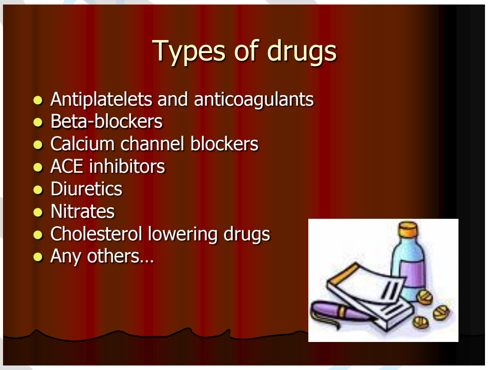


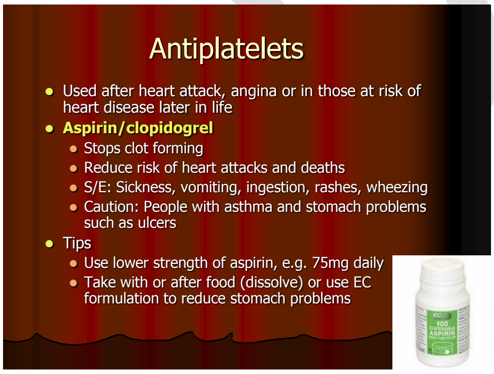


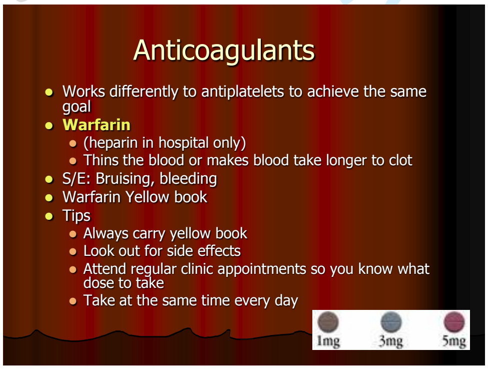




For peer review only - http://bmjopen.bmj.com/site/about/guidelines.xhtml

Imperial College London

## Beta-blockers

- Atenolol, bisoprolol
- Used after heart attack, angina, high BP, abnormal heart beats, heart failure
  - Works in many ways
  - Reduce related deaths
  - S/E: fatigue, wheezing, cold hands/feet, sleep problems, heart failure
  - Caution: Asthma and diabetes
- Tips
  - Do not stop taking them suddenly

## Calcium channel blockers

- Amlodipine, diltiazem
- Angina, high BP
  - Widen blood vessels
- S/E: Headache, upset stomach, ankle swelling, flushing
- Tips
  - Use once daily preparations to reduce s/e. For example, Dilzem XL.

## ACE inhibitors

- Lisinopril, Perindopril
- High BP, after heart attack, heart failure
  - Widen blood vessels
  - Reduce related deaths
- S/E: dry cough, low BP (first dose), taste disturbances, sore mouth, rashes, allergy- type reactions
- Comments: can interfere with kidneys, cause salt disturbances

## Diuretics (water tablets)

- Furosemide, Bumetanide
  - Used in heart failure
- Bendroflumethiazide
  - High BP
  - Reduce fluid by increase volume of urine
- S/E: Gout, worsen diabetes, affect salts
- Comments:
  - Often combined with other drugs
  - Salt disturbances reduced with co-amilofruse
  - Take morning or early afternoon

## Nitrates

- Glyceryl trinitrate, Isosorbide mononitrate
  - Used in Angina treatment and prophylaxis
  - Works by widen the blood vessels in the heart muscle which may be partly blocked
- S/E: Headache (temporary) flushing
- Comments:
  - Tablets/spray for under tongue, patches
  - Tablets to swallow
  - Paracetamol usually helps the headache

## Cholesterol lowering drugs

- Simvastatin, Atorvastatin, Pravastatin
  - After heart attack, angina
  - Reduce cholesterol production
  - Reduce heart disease events
- S/E: Muscle weakness, liver effects, headache
- Comments:
  - Take at night
  - Use with dietary advise
  - Care in liver disease
  - Any muscle problems – contact doctor immediately

## Other medicines

| | How it works | Side effects | Interactions |
|---|---|---|---|
| Digoxin | Increases force that heart pumps blood and reduces heart rate | Nausea, vomiting, slow pulse | Levels in the blood are increased by amiodarone, diltiazem, verapamil, dihydropyridines |
| Amiodarone | Antiarrhythmic – used to correct irregular heart rhythms | Sun sensitivity. Changes in thyroid function. Deposits in the cornea | Increases digoxin and warfarin. If given with other antiarrhythmics get and additive effect |
| Warfarin | Thins the blood | Bruising, bleeding (nose, urine, etc) | Effect increased by alcohol, antibiotics, amiodarone, cimetidine, simvastatin |

## What are medicines for the heart used for?

| | Nitrate | Beta blockers | Calcium channel blockers | ACE inhibitor | Diuretic | Digoxin |
|---|---|---|---|---|---|---|
| Angina | Y | Y | Y | | | |
| Raised blood pressure | | Y | Y | Y | Y | |
| Heart failure | Y | | | Y | Y | Y |
| Arrhythmias | | Y | Y | | | Y |


For peer review only - http://bmjopen.bmj.com/site/about/guidelines.xhtml

Imperial College London

## Drugs for Heart Failure

- ACE inhibitor
- Beta Blocker
- Spironolactone
- Diuretics
- Digoxin

*Managing Stress*

## Dealing with Stress

Dr. Kushangi Patel
Clinical Psychologist

## What is Stress?

**Physical Definition**

- A reaction that occurs in the body in response to a threat
- **Fight** the body gets ready to fight the threat
- **Flight** the body gets ready to run away

## What is Stress?

**Psychological Definition**

- Feeling under pressure
- Feeling unable to cope with the demands placed on us
- Feeling unable to adapt to demands

## How do you know when you are stressed?

## How do you know when you are stressed?

**Physical signs**

- Heart beats faster
- Faster breathing
- Muscle tension
- Increased sweating
- Feeling sick, indigestion, butterflies
- Dry mouth
- Increasingly needing the toilet
- Feeling dizzy

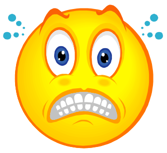

## How do you know when you are stressed?

**Psychological Signs**

- Memory difficulties
- Difficulty concentrating
- Muddled thinking
- Difficulty making decisions
- Becoming increasingly disorganised
- Irritability
- Low self confidence
- Social withdrawal
- Increasing tiredness

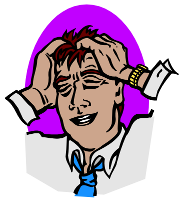



For peer review only - http://bmjopen.bmj.com/site/about/guidelines.xhtml

Imperial College London

## Purpose of stress

- Stress does have a purpose it's not all bad
- To protect ourselves from threat
- A small amount of stress helps increase our performance at things
- Stress can drive us to do things in life

## What makes you stressed?

**Environment**

- Work, family, too many social demands, driving, money, not enough opportunity for enjoyment

**Thoughts**

- I can't cope! I haven't got time! If I don't get this done….. will happen!
- These are e.g's of negative thoughts and predicting worse case scenarios.

## What causes stress in your life?

## What makes you stressed

**Emergency**

- Threat to life/ safety
- Events that pose an immediate threat to us cause stress levels to increase sharply and then fall quickly

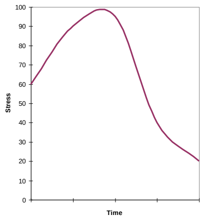


## What makes you stressed

**Daily Hassles**

- Such as shopping, organising holidays/social events, keeping on top of daily tasks at home and/or work.
- These frequently affect our stress levels, increasing it and decreasing it as hassles are encountered and then dealt with.
- Watch out for daily hassles – these lead to a gradual but **regular** build up of stress.

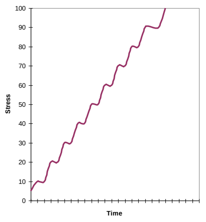


## How stress causes health problems

- Stress causes heart rate and blood pressure to increase and hormones are released to enable us to fight or flight.
- If high and/or frequent stress is experienced and these hormones are not used up by physical activity, the increase in heart rate and blood pressure damages arteries.
- The body heals this damage but as a result the artery walls become thicker and scarred.
- This can affect the supply of blood and oxygen to the heart.

## How health problems cause stress

- Health problems can affect all areas of life.
- Physical – feeling more tired, less able to do the things accomplished before the health problems started.
- Financial - time off work or a need to leave work altogether may have financial implications.
- Interests – a persons ability to engage in things they enjoy may be reduced temporarily or certain hobbies may have to be adapted.

## How health problems cause stress

- Identity – we all have views of ourselves. E.g. independent, responsible, fun, healthy. A health problem may affect this view if we feel we are no longer able to "be" these things.
- All these aspects can lead to feelings of frustration, anger and sadness.
- This can all increase stress levels
- Talk to other people about these feelings and frustrations or seek help from professionals, GP, Cardiac Rehab Nurse.



For peer review only - http://bmjopen.bmj.com/site/about/guidelines.xhtml

Imperial College London

## Health problems and stress

- Heart problems can knock self confidence so it is important to do things at a comfortable pace and build up gradually.
- Work to balance doing things at YOUR pace. Doing nothing and doing too much are both unhelpful.
- Take advice from GP's, cardiac team on how much you can do and when.
- Watch out for negative thoughts as they can lead you into a vicious cycle of inactivity and low mood which all can increase your stress levels.

## Vicious cycle of negative thoughts

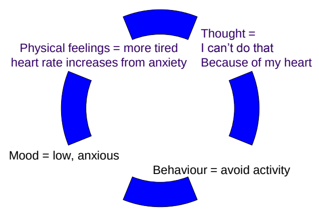


## What are the consequences of stress?

## Consequences of stress

- Health problems
- Difficulty in relationships
- Increase in sick leave
- Being unable to do things you need to do or enjoy
- Feeling upset, angry, frustrated, out of control

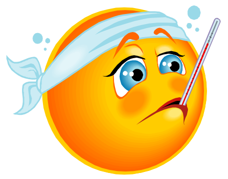

## Exercise

- In pairs talk about how you have coped with stress.
- What sort of things have you done in response to stress?
- What has worked?
- What has not worked so well?

## Coping with stress

**Helpful ways**

- Exercise
- Talk to someone
- Look at my thinking
- Prioritise tasks
- Share the load
- Relaxation
- Get guidance from other people or professionals involved in your care

**Unhelpful Ways**

- Increase alcohol/other drugs
- Work longer hours/ harder to finish tasks
- Shout
- Be on my own
- Ignore it

## Coping with stress

**Notice your early warning signs** – these may be physical signs and/or psychological signs

**Take action**

- **Calm your body by** –
- Relaxation
- Regular physical activity helps keep overall stress levels low
- Eat healthily
- Do something you enjoy
- Rest if you're tired
- Avoid increasing alcohol, smoking or caffeine to cope

## Coping with stress

- **Calm your mind by** –
- Relaxation
- Looking at your thoughts:
  Are you focusing on the negative?
  Are you predicting the worse case scenario without any evidence for it?
- Plan and prioritise
- Think about the long-term – is saying no now going to help you later on

**It may be difficult to do all the techniques so find out what works for you!**


For peer review only - http://bmjopen.bmj.com/site/about/guidelines.xhtml

Imperial College London

*Eating for a healthy heart*

This is an interactive session delivered by a cardiac specialist dietician – no presentation available.

DRAFT
For peer review only

Imperial College London

*Exercise and the benefits for your heart*

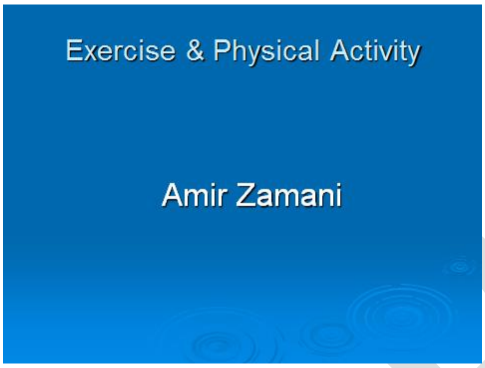


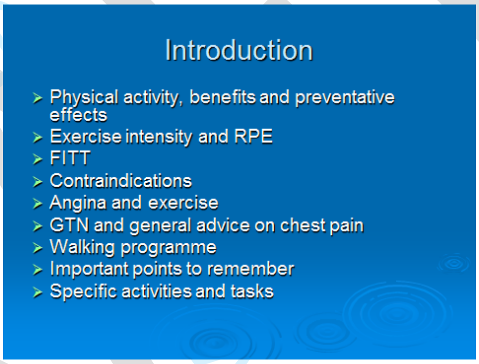


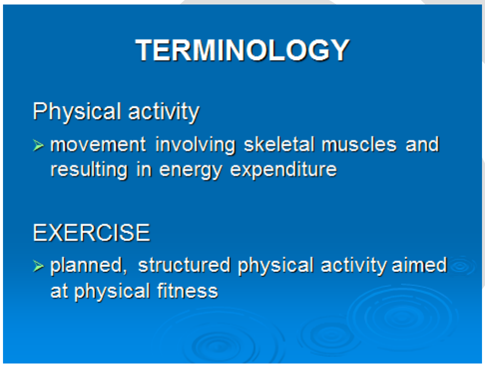


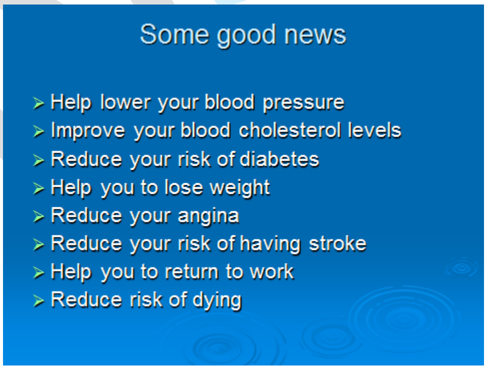


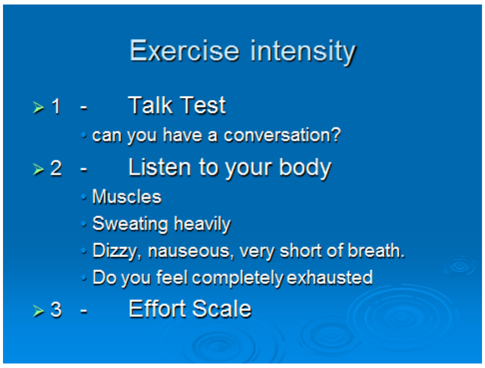


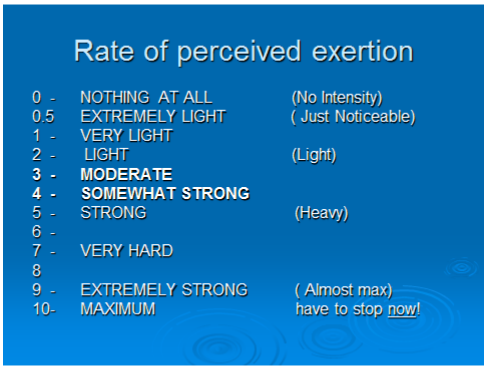

Imperial College London

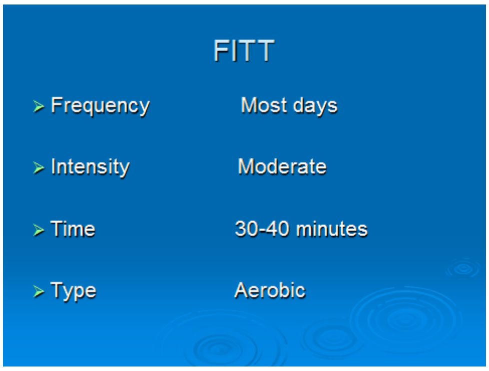


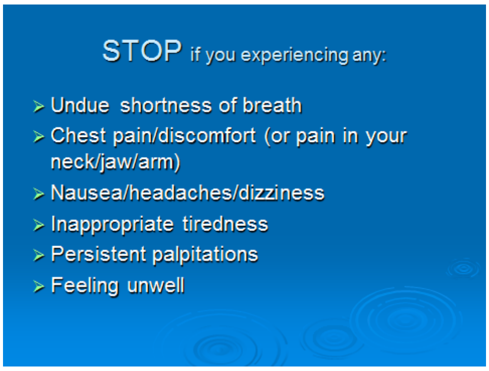


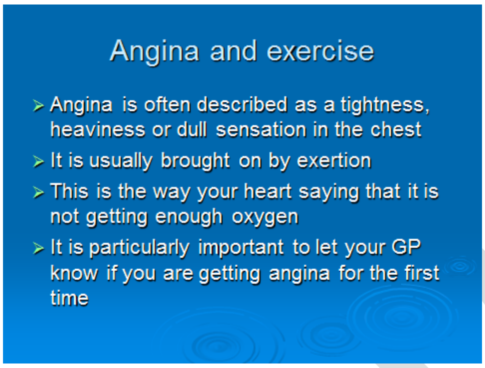


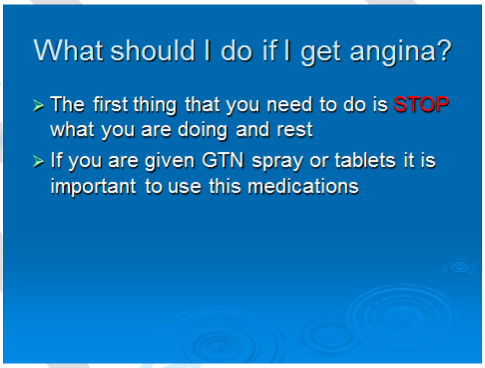


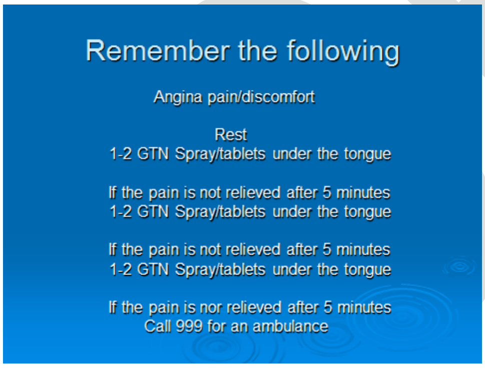


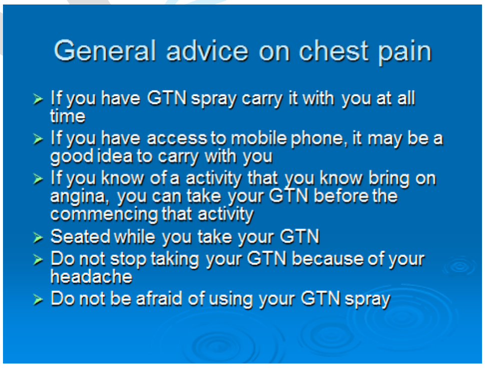


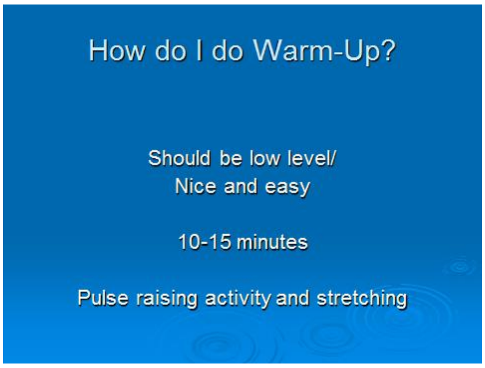


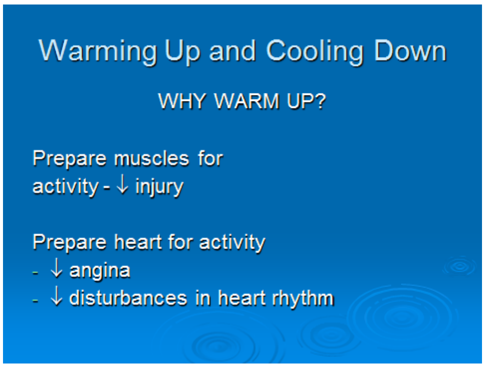

Imperial College London

## What sort of activity

- Aerobic, most beneficial activity for your heart
- Resistance or strength training

## Walking programme

| Stage of recovery | Length of walk (in minute) |
|---|---|
| (Approx. week 1) | 5 Minutes: several times per day Strolling/leisurely pace |
| (approx. week 2) | 10 minutes: twice a day, Leisurely pace |
| (approx. week 3) | 15 minutes: daily, Leisurely/moderate pace |
| (approx. week 4) | 20 minutes: daily, moderate pace |
| (approx. week 5) | 25 minutes: daily, brisk pace |
| (approx. week 6) | 30 minutes: daily brisk pace |
| Target | 30-45 minutes: daily brisk walk |

## Cool Down

WHY COOL DOWN?

↓ Fainting and dizziness

↓ Disturbances to your heart

↓ Muscle soreness

## How do I cool Down?

Goal is to return body to its resting state

Gradually slow down the activity you are doing and stretch

10 minutes

## Sensible Precautions

- Do not exercise if you feel unwell
- Do not exercise on a full or empty stomach
  - Light meal/snack 1½ - 2 hours before
- Do not exercise in extreme temperatures
- Wear suitable clothing
- Take your medications
- Good days and Bad days
- Enjoy!

*Have an active, healthy, happy life!*

Imperial College London

*Risk factors and making lifestyle changes*

## Risk Factors & Making Changes

Olivia Molloy
Cardiac Rehabilitation Sister

## Modifiable and Non Modifiable Risk Factors

| • Modifiable | • Non Modifiable |
|---|---|
| 1. Smoking<br>2. High Blood Pressure<br>3. High Cholesterol<br>4. Physical Inactivity<br>5. Being Overweight<br>6. Diabetes<br>7. Alcohol consumption<br>8. Impaired glucose regulation | 1. Family History of Heart Disease<br>2. Age<br>3. Ethnic Background |

## Smoking

FACT

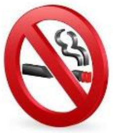

- Smoking is one of the major causes of cardiovascular disease. People who smoke are twice as likely to have a heart attack as to people who have never smoked

## Smoking

- Smoking damages the lining of the arteries leading to build up of Atheroma.
- Carbon Monoxide in cigarette smoke reduces the amount of oxygen that the blood can carry to the heart and around the body
- Nicotine stimulates adrenaline which increases heart rate and raises blood pressure = harder work load for your heart
- Second Hand Smoking…..

## Blood Pressure

- Blood Pressure represents the pressure of the blood in your arteries. We need a certain amount of pressure in our body in order to keep our blood ( circulation) flowing
  - High Blood Pressure is also known as **“Hypertension”**

This is when your blood pressure is higher than the recommended level.

## Blood Pressure

- What does Systolic and Diastolic Pressure Mean?
- Readings:
  - 140/80mmhg = Non Diabetics
  - Diabetics = 130/80mmhg

**Maximum**

Imperial College London

## Blood Pressure

- Why is High Blood Pressure dangerous

Remember

- High Blood Pressure does not make you feel Sick.

## Cholesterol

- Cholesterol is a fatty substance which is found in the blood. Too much cholesterol is a contributing factor for Heart Disease.
- **LDL**
- **HDL**
- **Total Cholesterol**
- **Triglycerides**

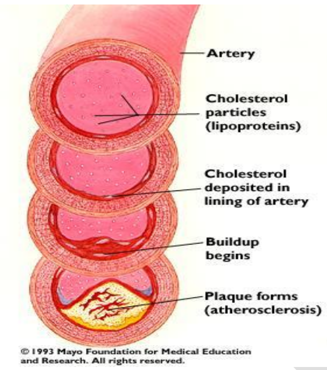


## Excercise

- In the UK people who are not physically active are twice as likely to have a heart attack
- In the UK; 7 out of 10 people do not do physical activity to benefit their health
- What can you do?

## Keeping a Healthy Weight & Shape

- Being overweight can increase your risk of developing Cardiovascular disease.
- Keeping close to a healthy weight will help you control your blood pressure and reduce the workload that you hear has to do.

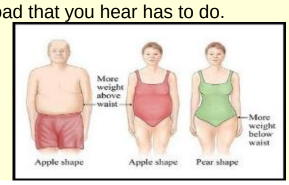


## Family History

- If you have a family history of CVD your own risk of developing the condition is increased.
- A family history means if your

  father or brother >55

  or

  mother or sister >65
- Non Modifiable risk factor

## Eating Healthy For Your Heart

- Eat plenty of fruit and veg
- Choose healthier fats
- Eat oily fish regularly
- Reduce the amount of salt you eat

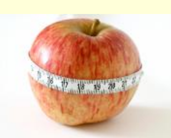

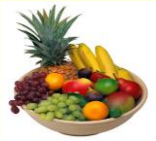

## Alcohol

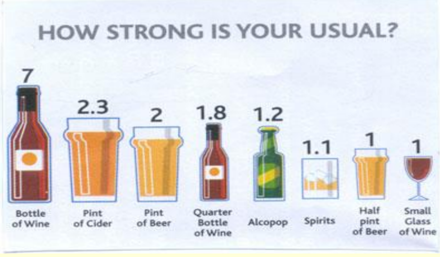




For peer review only - http://bmjopen.bmj.com/site/about/guidelines.xhtml

Imperial College London

## Stress

- Stress is not a direct risk factor for CVD but it is possible that stress may contribute to it, or perhaps bring on some symptons.
- The way you deal with stress can encourage unhealthy behaviours e.g. smoking unhealthy eating , alcohol etc

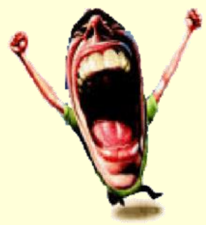

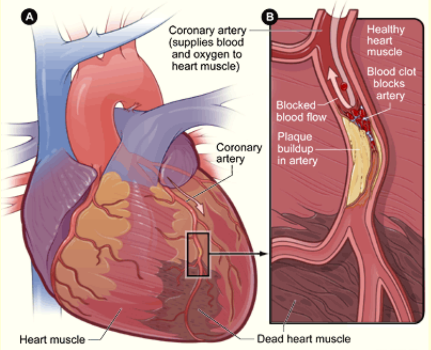


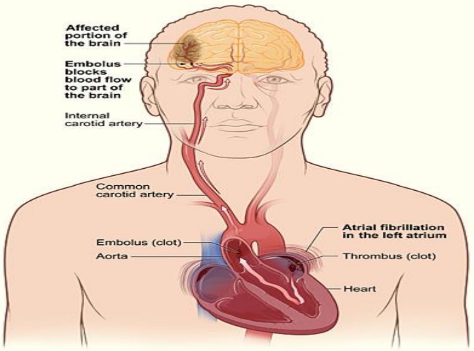


For peer review only
DRAFT

Imperial College London

*The heart and how it works*

## The Heart & How it works

Olivia Molloy
Cardiac Rehabilitation Nurse Specialist

## The Human Heart

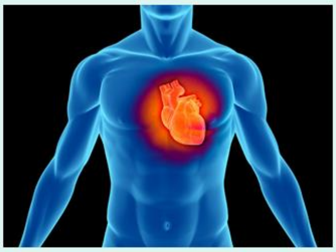

## The Heart

- The heart is a fist sized organ which lies within the chest behind the sternum (breast bone). The heart sits on the diaphragm, the main muscle of breathing, which is found beneath the lungs. The heart is considered to have two 'sides' – the right side and the left side.

  The heart has four chambers – an atria and ventricle on each side.

- The atria are both supplied by large blood vessels that bring blood to the heart (see below for more details).

- Atria have special valves that open into the ventricles. The ventricles also have valves but in this case they open into blood vessels. The walls of the heart chambers are made mainly of special heart muscle. The different sections of the heart have to contract (squeeze) in the correct order for the heart to pump blood efficiently with each heartbeat

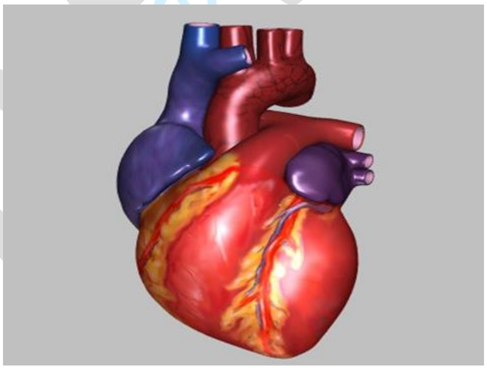

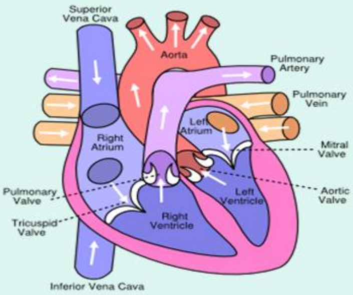


## The Function of the Heart

- The heart is a muscular pump that pushes blood through blood vessels around the body.

- Essential to life, the heart beats continuously, pumping the equivalent of more than 14,000 litres of blood every day.

  Blood vessels form the living system of tubes that carry blood both to and from the heart.

- All cells in the body need oxygen and the vital nutrients found in blood. Without oxygen and these nutrients, the cells will die.



For peer review only - http://bmjopen.bmj.com/site/about/guidelines.xhtml

Imperial College London

## The Heart Cont…

- The heart helps to provide oxygen and nutrients to the body's tissues and organs by ensuring a rich supply of blood.
- Not only do blood vessels carry oxygen and nutrients, but they also transport carbon dioxide and waste products away from our cells.
- Carbon dioxide is passed out of the body by the lungs, and most of the other waste products are disposed of by the kidneys.

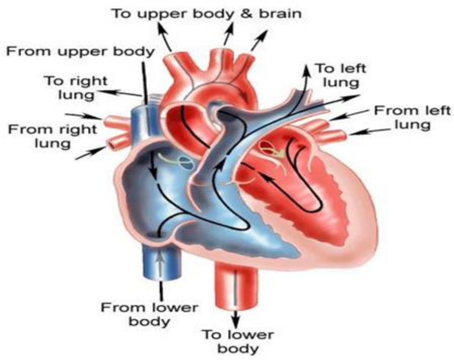


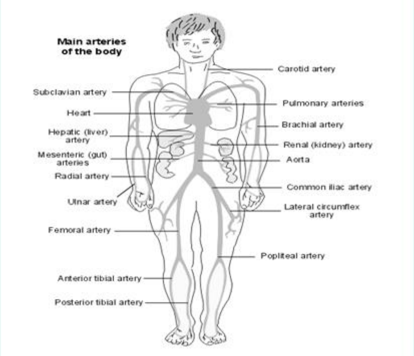


## The Blood Supply to the Heart

- Like any other muscle, the heart muscle needs a good blood supply. The coronary arteries take blood to the heart muscle. These are the first arteries to branch off the aorta - the large artery that takes blood to the body from the left ventricle.

  The right coronary artery mainly supplies the muscle of the right ventricle.

  The left coronary artery quickly splits into two and supplies the rest of the heart muscle.

  The main coronary arteries divide into many smaller branches to supply all the heart

## The coronary arteries of the heart

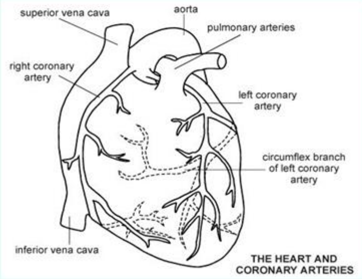


## Angioplasty

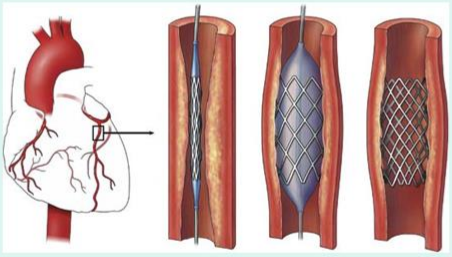

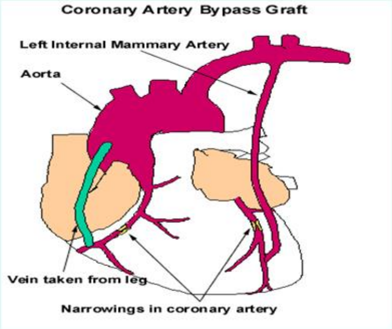


## The Heart Valves

The heart also contains four valves.

- Their role is to ensure the blood flows in a forward direction and prevents a backward flow during any part of the pump action (or cardiac cycle).
- An atrioventricular valve sits on both the left and right sides of the heart between each atrium and ventricle.
- These are the tricuspid valve (right side) and the mitral valve (left side).
- The two remaining valves sit on the outflow tract of the left and right ventricles.



For peer review only - http://bmjopen.bmj.com/site/about/guidelines.xhtml

Imperial College London

## Valves Cont….

- The pulmonary valve is between the right ventricle and the pulmonary artery, which takes deoxygenated blood to the lungs.
- The aortic valve sits between the left ventricle and the aorta, which takes oxygenated blood to the body's tissues.
- These latter two valves are semilunar; they contain three cusps which close to prevent the backward flow of blood from the outflow vessels during the diastolic (filling) phase of the cardiac cycle. The left side of the heart is inevitably under a much higher pressure than the right side, which delivers blood to the lungs only.

## Heart Valves

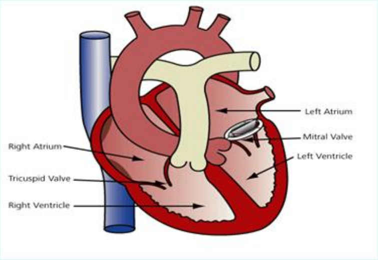


## Chest Pain

- Stop what your doing
- Sit Down and rest
- If you have GTN spray or tablets, use the spray and take your tablets as instructed by your doctor or cardiac rehab nurse

## Chest Pain Continued

- If you don't have GTN CALL 999 if pain does not go away.
- Aspirin , if you are not allergic to aspirin chew 300mgs until the ambulance arrives
- If the pain, discomfort or chest tightness continues especially if its gone on within 15 minutes
- DONT WAIT CALL 999 RIGHT AWAY

CALL THE AMBULANCE AND STAY RESTING

## Basic Life Support

- Show DVD Recording here

## Blood Pressure / Pulse

- Non Diabetic Patients — 140/80 mmhg
- Diabetic Patients — 130/80 mmhg
- 
- Pulse for all — •60 – 100 bpm

## Diabetics

- All Diabetics Blood Sugar should be less than

6



For peer review only - http://bmjopen.bmj.com/site/about/guidelines.xhtml

Imperial College London

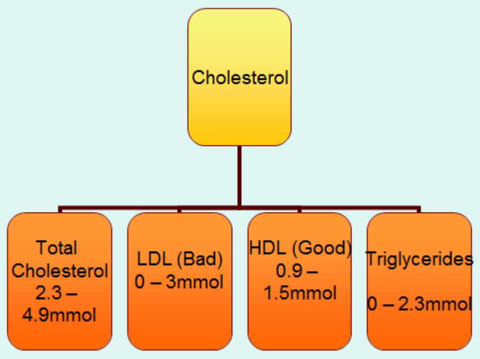


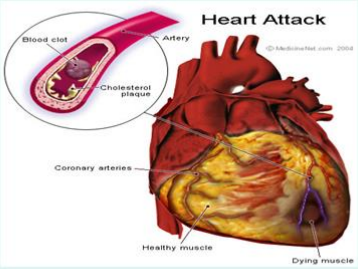


## Cont….

- Arterioles are the smallest arteries in the body. They deliver blood to capillaries. Arterioles are also capable of constricting or dilating and by doing this they control how much blood enters the capillaries.

  Capillaries are tiny vessels that connect arterioles to venules. They have very thin walls which allow nutrients from the blood to pass into the body tissues. Waste products from body tissues can also pass into the capillaries. For this reason capillaries are known as exchange vessels.

  Groups of capillaries within a tissue reunite to form small veins called venules. Venules collect blood from capillaries and drain into veins.

  Veins are the blood vessels that carry blood back to the heart. They may contain valves which stop blood flowing away from the heart.

## Cont….

- The right side of the heart receives deoxygenated blood (lacking oxygen) from the body. After passing through the right atrium and right ventricle this blood is pumped to the lungs. Here blood picks up oxygen and loses another gas called carbon dioxide. Once through the lungs, the blood flows back to the left atrium. It then passes into the left ventricle and gets pumped into the aorta, the main artery supplying the body. Oxygenated blood is then carried though blood vessels to all the body's tissues. Here oxygen and other nutrients pass into the cells where they are used to perform the body's essential functions.

  A blood vessels main function is to transport blood around the body. Blood vessels also play a role in controlling your blood pressure.

  Blood vessels are found throughout the body. There are five main types of blood vessels: arteries, arterioles, capillaries, venules and veins.

  Arteries carry blood away from the heart to other organs. They can vary in size. The largest arteries have special elastic fibres in their walls. This helps to complement the work of the heart, by squeezing blood along when heart muscle relaxes. Arteries also respond to signals from our nervous system, either constricting (tightening) or dilating (relaxing).

## Blood Flow Through the Body

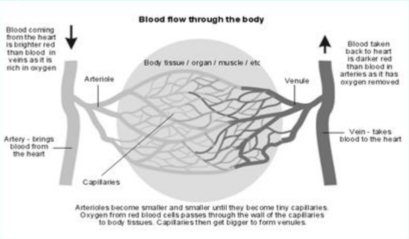

Imperial College London

For peer review only
DRAFT

## References

1. Sivananda: Yoga Teachers' Training Manual

2. Sivananda Yoga Vedanta Centre: Yoga-Your Home Practice Companion

3. Sivananda Yoga Vedanta centre: The Yoga Cookbook:Vegetarian Food for Body and Mind

4. H.David Coulter: Anatomy of Hatha Yoga

5. Swami Vishnu-Devananda: The Complete Illustrated Book of Yoga

6. www.yoga.about.com

7. www.innerhealthstudio.com



For peer review only - http://bmjopen.bmj.com/site/about/guidelines.xhtml

**Modified CONSORT flow diagram for individual randomized controlled trials of nonpharmacologic treatments.**

An extra box per intervention group relating to care providers and centers has been added.
IQR = interquartile range; max = maximum; min = minimum

**Enrollment**

Assessed for eligibility (n =627 …)

Excluded (n = 547)

- Not meeting inclusion criteria (n = 420)
- Refused to participate (n =92)
- No response (n =35)

Randomized (n =80.)

**Allocation : Patients**

Yoga plus usual NHS cardiac rehabilitation care

Allocated to intervention (n = 40)

Received allocated intervention (n = 40)

Usual NHS cardiac rehabilitation care alone

Allocated to intervention (n =40)

Received allocated intervention (n = 40)

**Allocation: Care Providers**

Yoga: 1 yoga teacher, I centre, n=40
Usual care providers (n =2 ), teams (n =? ), centers (n=4 ) providing usual NHS cardiac rehabilitation care

Number of patients: NACR 2018 audit figures for cardiac rehab reports that in the year 2016-2017, there were 33 programmes in London seeing 11, 384 patients, suggesting on average each London programme(provider) sees about 350 patients per year.
https://www.bhf.org.uk/informationsupport/publications/statistics/national-audit-of-cardiac-rehabilitation-quality-and-outcomes-report-2018

Usual care providers (n = 2), teams (n = ?), centers (n=4) providing usual NHS cardiac rehabilitation care.

Number of patients: NACR 2018 audit figures for cardiac rehab reports that in the year 2016-2017, there were 33 programmes in London seeing 11, 384 patients, suggesting on average that each London programme (provider) sees about 350 patients per year.
https://www.bhf.org.uk/informationsupport/publications/statistics/national-audit-of-cardiac-rehabilitation-quality-and-outcomes-report-2018

**Follow-up Patients**

Discontinued intervention before 18 classes & no follow-up (n=11, 4 refused to complete yoga intervention, 6 too unwell to complete intervention, 1 returned to work)
Refused to complete intervention before 18 classes but did attend follow-up assessment, n=4
NB. Attendance at usual cardiac rehab was not monitored as part of this study

Lost to follow-up (n=5 (4 refused, 1 unwell )

NB. Attendance at usual cardiac rehab was not monitored as part of this study

**Analysis Patients**

Analysed (n=25 )
Excluded from analysis (n=15 discontinued yoga intervention before 18 classes (per protocol pre-specified condition)

Analysed (n=35 )
Excluded from analysis (n=5 who did not attend follow-up assessment )

**Cite as:** Boutron I, Altman DG, Moher D, Schulz KF, Ravaud P. CONSORT Statement for Randomized Trials of Nonpharmacologic Treatments: A 2017 Update and a CONSORT Extension for Nonpharmacologic Trial Abstracts. Annals of Internal Medicine. 2017 Jul 4;167(1):40–7.

For peer review only - http://bmjopen.bmj.com/site/about/guidelines.xhtml

# CONSORT 2010 checklist of information to include when reporting a randomised trial*

| Section/Topic | Item No | Checklist item | Reported on page No |
|---|---|---|---|
| **Title and abstract** | | | |
| | 1a | Identification as a randomised trial in the title | 1 |
| | 1b | Structured summary of trial design, methods, results, and conclusions (for specific guidance see CONSORT for abstracts) | 2 |
| **Introduction** | | | |
| Background and objectives | 2a | Scientific background and explanation of rationale | 3 |
| | 2b | Specific objectives or hypotheses | 3 |
| **Methods** | | | |
| Trial design | 3a | Description of trial design (such as parallel, factorial) including allocation ratio | 3, 4 |
| | 3b | Important changes to methods after trial commencement (such as eligibility criteria), with reasons | 4 |
| Participants | 4a | Eligibility criteria for participants | 3, 4 |
| | 4b | Settings and locations where the data were collected | 7 |
| Interventions | 5 | The interventions for each group with sufficient details to allow replication, including how and when they were actually administered | 4, and supplementary files, 'Yacht Study package_v1_2, which includes 'Policy for Rehabilitation in Ealing' |
| Outcomes | 6a | Completely defined pre-specified primary and secondary outcome measures, including how and when they were assessed | 5-7 |
| | 6b | Any changes to trial outcomes after the trial commenced, with reasons | N/A |
| Sample size | 7a | How sample size was determined | 7, 8 |
| | 7b | When applicable, explanation of any interim analyses and stopping guidelines | N/A |

For peer review only

| | | | |
|---|---|---|---|
| Randomisation: | | | |
| Sequence generation | 8a | Method used to generate the random allocation sequence | 3-4 |
| | 8b | Type of randomisation; details of any restriction (such as blocking and block size) | 3-4 |
| Allocation concealment mechanism | 9 | Mechanism used to implement the random allocation sequence (such as sequentially numbered containers), describing any steps taken to conceal the sequence until interventions were assigned | 3-4 |
| Implementation | 10 | Who generated the random allocation sequence, who enrolled participants, and who assigned participants to interventions | 3-4 |
| Blinding | 11a | If done, who was blinded after assignment to interventions (for example, participants, care providers, those assessing outcomes) and how | 7 |
| | 11b | If relevant, description of the similarity of interventions | N/A |
| Statistical methods | 12a | Statistical methods used to compare groups for primary and secondary outcomes | 8 |
| | 12b | Methods for additional analyses, such as subgroup analyses and adjusted analyses | 8 |
| **Results** | | | |
| Participant flow (a diagram is strongly recommended) | 13a | For each group, the numbers of participants who were randomly assigned, received intended treatment, and were analysed for the primary outcome | Figure 1 |
| | 13b | For each group, losses and exclusions after randomisation, together with reasons | Figure 1 d supplemental tables |
| Recruitment | 14a | Dates defining the periods of recruitment and follow-up | 4 |
| | 14b | Why the trial ended or was stopped | 4 |
| Baseline data | 15 | A table showing baseline demographic and clinical characteristics for each group | Table 1, Supplemental tables |
| Numbers analysed | 16 | For each group, number of participants (denominator) included in each analysis and whether the analysis was by original assigned groups | Figure 1 |
| Outcomes and estimation | 17a | For each primary and secondary outcome, results for each group, and the estimated effect size and its precision (such as 95% confidence interval) | Tables 2-5 |
| | 17b | For binary outcomes, presentation of both absolute and relative effect sizes is recommended | N/A |
| Ancillary analyses | 18 | Results of any other analyses performed, including subgroup analyses and adjusted analyses, distinguishing pre-specified from exploratory | N/A |
| Harms | 19 | All important harms or unintended effects in each group (for specific guidance see CONSORT for harms) | 9 |



For peer review only - http://bmjopen.bmj.com/site/about/guidelines.xhtml

For peer review only

| | | | |
|---|---|---|---|
| **Discussion** | | | |
| Limitations | 20 | Trial limitations, addressing sources of potential bias, imprecision, and, if relevant, multiplicity of analyses | 12 |
| Generalisability | 21 | Generalisability (external validity, applicability) of the trial findings | 12 |
| Interpretation | 22 | Interpretation consistent with results, balancing benefits and harms, and considering other relevant evidence | 12 |
| **Other information** | | | |
| Registration | 23 | Registration number and name of trial registry | 1 |
| Protocol | 24 | Where the full trial protocol can be accessed, if available | Available on request |
| Funding | 25 | Sources of funding and other support (such as supply of drugs), role of funders | 12 |

*We strongly recommend reading this statement in conjunction with the CONSORT 2010 Explanation and Elaboration for important clarifications on all the items. If relevant, we also recommend reading CONSORT extensions for cluster randomised trials, non-inferiority and equivalence trials, non-pharmacological treatments, herbal interventions, and pragmatic trials. Additional extensions are forthcoming: for those and for up to date references relevant to this checklist, see www.consort-statement.org.

For peer review only



For peer review only - http://bmjopen.bmj.com/site/about/guidelines.xhtml

BMJ Open

# Yoga and Cardiovascular Health Trial (YACHT): a UK-based randomised controlled trial of a yoga intervention plus usual care vs usual care alone following an acute coronary event



| | |
|---|---|
| Journal: | *BMJ Open* |
| Manuscript ID | bmjopen-2019-030119.R1 |
| Article Type: | Original research |
| Date Submitted by the Author: | 01-Sep-2019 |
| Complete List of Authors: | Tillin, Therese; University College London, Institute of Cardiovascular Science<br>Tuson, Claire; York Hospitals NHS Trust<br>Sowa, Barbara; West London Mental Health NHS Trust<br>Chattopadhyay, Kaushik; University of Nottingham School of Health Sciences; London School of Hygiene and Tropical Medicine<br>Sattar, Naveed; University of Glasgow<br>Welsh, Paul; University of Glasgow<br>Roberts, Ian; London School of Hygiene and Tropical Medicine<br>Ebrahim, Shah; London School of Hygiene and Tropical Medicine<br>Kinra, Sanjay; London School of Hygiene and Tropical Medicine<br>Hughes, A; University College London, Institute of Cardiovascular Science<br>Chaturvedi, Nishi; University College London, Institute of Cardiovascular Science |
| <b>Primary Subject Heading</b>: | Cardiovascular medicine |
| Secondary Subject Heading: | Rehabilitation medicine |
| Keywords: | Yoga, Cardiac rehabilitation, diastolic function, exercise, blood pressure, heart rate |
| | |



SCHOLARONE™
Manuscripts

# Yoga and Cardiovascular Health Trial (YACHT): a UK-based randomised controlled trial of a yoga intervention plus usual care vs usual care alone following an acute coronary event

Authors

Therese Tillin, Institute of Cardiovascular Science, UCL, corresponding author: email: t.tillin@ucl.ac.uk

Claire Tuson, York Teaching Hospital NHS Foundation Trust

Barbara Sowa, West London NHS Trust

Kaushik Chattopadhyay, Division of Epidemiology and Public Health, University of Nottingham

Naveed Sattar, Institute of Cardiovascular & Medical Sciences, University of Glasgow

Paul Welsh, Institute of Cardiovascular & Medical Sciences, University of Glasgow

Ian Roberts, London School of Hygiene and Tropical Medicine

Shah Ebrahim, London School of Hygiene and Tropical Medicine

Sanjay Kinra, London School of Hygiene and Tropical Medicine

Alun Hughes, Institute of Cardiovascular Science, UCL

Nishi Chaturvedi, Institute of Cardiovascular Science, UCL

Word count: abstract: 292 words, main text: 5504 words

For peer review only



For peer review only - http://bmjopen.bmj.com/site/about/guidelines.xhtml

## Abstract

**Objective:** To determine effects of yoga practice on subclinical cardiovascular measures, risk factors and neuro-endocrine pathways in patients undergoing cardiac rehabilitation following an acute coronary event.

**Design:** 3-month, two arm (yoga+usual care vs usual care alone) parallel randomised controlled trial

**Setting:** Referrals from one general hospital and two primary care cardiac rehabilitation centres in London. Assessments were conducted at Imperial College London.

**Participants:** 80 participants, aged 35-80 years referred to cardiac rehabilitation programmes October 2012-April 2014. 68% were men, 60% were South Asian.

**Intervention:** The yoga intervention consisted of 18-24 group classes, conducted by a certified yoga teacher and included exercises in stretching, breathing, healing imagery and deep relaxation. It was pre-specified that yoga group participants should complete at least 18 classes for inclusion in analysis. Participants and partners in both groups were invited to attend once weekly a 6-12 week local standard NHS cardiac rehabilitation programme.

**Main outcome measures**: i) estimated left ventricular filling pressure (E/e'), ii) distance walked, fatigue and breathlessness in a 6-minute walk test (6MWT), iii) BP, heart rate and estimated peak $VO_2$ following a three-minute step-test. Effects on other measures of cardiac structure and function, the hypothalamus-pituitary-adrenal axis, autonomic function, body fat, blood lipids and glucose, stress and general health were also explored.

**Results** 25 participants in the yoga+usual care group and 35 participants in the usual care group completed the study. Following the 3-month intervention period, E/e' was not improved by yoga (E/e': between group difference: yoga minus usual care:-0.40(-1.40, 0.61) The 6MWT and blood pressure, heart rate and peak $VO_2$ and secondary outcomes also showed no benefits of yoga.

**Conclusions** We found no evidence that a structured 3-month yoga intervention added to usual care following an acute coronary event improved any cardiovascular or neuro-endocrine measures.


Keywords: Yoga, cardiac rehabilitation, exercise, blood pressure, heart rate

Trial registration https://clinicaltrials.gov/ct2/show/record/NCT01597960

For peer review only

Article Summary

Strengths and Limitations of this study

- Comprehensive clinical and subclinical cardiovascular measures before and after a yoga intervention (plus usual cardiac rehabilitation) vs usual cardiac rehabilitation
- Real world setting – older people following an acute coronary event
- High level of dropout, particularly in the yoga plus usual cardiac rehabilitation arm
- We can only assess the potential of yoga in addition to usual cardiac rehabilitation following an acute coronary event.

Introduction

The practice of yoga originated in ancient India as a form of exercise which includes breath control, the adoption of bodily postures and meditation which aim to increase strength and flexibility and to aid physical and mental wellbeing.(1) Yoga has been reported to reduce stress and depression and is thought to improve biological cardiovascular risk factors.(2-4) However, despite claims of benefits, the effects of yoga on cardiovascular outcomes remain unclear. Previous systematic reviews (5-12) confirm that investigations of the health benefits of yoga and underlying mechanisms have often been hampered by poor study design, including small sample sizes, inadequate adjustment for confounders, lack of randomisation, unsatisfactory masking of outcomes to assessors, and publication bias. Also, many studies have been conducted in healthy young participants and it is not certain that these findings are generalisable to older adults with established disease.

Cardiac rehabilitation (CR) has been shown to improve cardiovascular mortality and hospital re-admissions in patients with coronary heart disease.(13) However, for myocardial infarction (MI), coronary bypass grafts (CABG) and percutaneous coronary intervention (PCI) patients uptake to CR across in UK was only ~45% in 2012-3 with low representation of ethnic minority people.(14) Yoga could therefore be a useful adjunct to CR.

In this UK-based randomised controlled study (Yoga and Cardiovascular Health Trial (YACHT)), we hypothesised that yoga would be associated primarily with improvements in cardiovascular function and exercise capacity both chronically and acutely in people eligible for CR. The chronic study compared cardiovascular measures at 3 months between two groups randomised either to usual care (including CR) or to usual care plus a programme of yoga classes. For the chronic study, where the emphasis was on rehabilitation following a coronary event, we focussed on the ratio between early mitral inflow and mitral annular early diastolic velocity (E/e') as the primary cardiac measure. E/e' provides an estimate of left ventricular (LV) filling pressure, (15) an aspect of LV diastolic function that predicts survival after myocardial infarction.(16) We also performed a 6-minute walk test (6MWT) as a measure of exercise tolerance and a three-minute step-test as a measure of cardiopulmonary fitness. These measures were chosen as they are reproducible and safe tests which are improved by CR,(17-20) and predict outcomes in people with coronary heart disease.(16, 21-23) Measures chosen for the acute study (before and after the first session of yoga) included blood pressure (BP) and heart rate before and after exercise as indicators of cardiovascular and autonomic function which are associated with cardiovascular outcomes. (21, 23)

In addition to these primary outcome measures, we studied a range of other cardiovascular risk factors and measures which might be expected to improve following CR and provide mechanistic insight into any beneficial effect of yoga; these included markers of the hypothalamic–pituitary axis, measures of autonomic function, measures of cardiac structure and function, brachial and central resting and 24 hour ambulatory



For peer review only - http://bmjopen.bmj.com/site/about/guidelines.xhtml

blood pressure, markers of atherosclerosis, blood glucose and lipids and self-reported health, lifestyle factors and perceived stress levels.

For peer review only



For peer review only - http://bmjopen.bmj.com/site/about/guidelines.xhtml

**Methods**

Study population

Inclusion criteria included referral to CR programmes in north-west London following an acute coronary syndrome (myocardial infarction, percutaneous coronary intervention, coronary artery bypass grafting). Pre-specified inclusion criteria were age between 35 and 80 years, male or female, without co-morbid disease or mobility limitations that would preclude participation in CR and our investigations, and, given the north-west London area of recruitment, able to understand English or Punjabi. Ethnicity was self-defined, and verified by country of birth of all 4 grandparents. 80 participants were recruited following discharge from hospital and randomised in equal numbers to the yoga intervention plus their standard CR programme, or to standard CR programme (usual care) alone. Randomisation was performed by an independent researcher using a standard computerized algorithm (customised Java web application (srub)) and stratified by ethnicity (South Asian and non-South Asian), gender, 5 year age group and rehabilitation centre. The generated sequence was displayed only to the user at the time of assignment to the yoga intervention or usual care. 75% of participants were recruited from referrals to CR programmes at Ealing Hospital in west London, with the remainder recruited from two primary care CR programmes in north-west London (Harrow and Brent (Flexi-Heart Plan)). Recruitment of the planned 80 participants took place between October 2012 and April 2014, with the final participant seen for 3-month follow-up measures in July 2014.

Eligibility criteria were broadened in January 2013 and April 2013 respectively, with ethical approval, to include patients who had undergone coronary artery bypass grafting or who had received medical management only for their acute coronary event. The initial study plans were to recruit only patients referred to a CR programme post-angioplasty as treatment for an acute coronary syndrome. With cardiologist advice, it was felt that the earlier decision to exclude these patients based on safety grounds was unnecessary given the gentle and tailored nature of the exercises.

Patient and Public Involvement

Patients and public were not involved in the study design, conduct, results, evaluation or dissemination.

Ethical approval for the study was granted by Camberwell St Giles Research Ethics Committee (Ref: 12/LO/0597). Informed written consent was obtained from all participants.

Yoga intervention

The yoga intervention was delivered on a twice-weekly group session basis for 12 weeks alongside the usual care, 6-12 week CR programme. There were 24 yoga classes in total. Participants' partners were invited to take part in each session as a method of improving adherence. The yoga session was designed and conducted by a teacher certified in yoga and CR, and included gentle exercises in deep relaxation, stretching, breathing, healing imagery and a healthy diet. A prescription of exercises with an accompanying DVD was provided to be performed regularly at home. Each session lasted approximately 75 minutes, divided into three equal parts: breathing exercises, yogic poses and meditations, education and discussion (details in supplemental material: YACHT study package_v1.2.pdf ). Individuals randomised to the yoga arm had their standard CR care delivered at a separate time to those randomised to usual CR, (although delivered by the same teams), to reduce risks of contamination. Because the study was also



For peer review only - http://bmjopen.bmj.com/site/about/guidelines.xhtml

designed to examine mechanisms underlying any beneficial effects of yoga (24), there was a pre-specified requirement for participants in the yoga + usual care group to complete at least 18 yoga classes.

Usual care

Usual care is described in the supplemental material (YACHT study package_v1.2.pdf) and was similar in all centres in accordance with the UK's National Institute for Health and Care Excellence (NICE) guidelines (https://www.nice.org.uk/guidance/cg48, accessed 25/8/2017) and British Association for Cardiac Prevention and Rehabilitation (BACR) standards (25) with core components of lifestyle (physical activity, exercise, diet and weight management, smoking cessation), education, risk factor management, psychosocial, cardio-protective drug therapy and long-term management strategies. Patients and their partners were invited to attend once-weekly for a 6-12 week programme tailored to individual needs and including 1) on-going risk factor monitoring/advice/support, 2) exercise sessions in a gym, led by cardiac physiologist or a home-based exercise programme, 3) health education lectures (led by CR sister, pharmacist, dietician, clinical psychologist, cardiac physiologist), 4) relaxation sessions, 5) guidance and supervised use of the "Edinburgh Heart Manual" (http://www.theheartmanual.com/Pages/default.aspx, accessed 26/9/2017 ).

Outcome measures

Chronic study: All measures performed pre-intervention and 3 months post-intervention

Primary outcome measures

Echocardiography

Transthoracic two-dimensional (2D) and Doppler echocardiography was performed as previously described (26). Transmitral flow velocity during the early filling phase (E) was acquired by pulsed Doppler and averaged from three consecutive cycles. Tissue Doppler Imaging was performed on the lateral and septal LV wall. Peak velocities during early diastole (e') were averaged from three consecutive representative cycles. The e' wave velocities measured from the lateral and septal walls were averaged. The primary cardiac outcome was the ratio of early filling and early myocardial velocity (E/e'), a non-invasive estimate of LV filling pressure.(15)

Exercise capacity and physical fitness

Exercise capacity was measured by a 6-minute walk test conducted along a 30 m straight path in an outdoor covered area marked clearly with the beginning and end of each lap. Participants wore appropriate shoes and loose-fitting clothing and rested in a chair for 10 minutes before the start of the test. Participants were asked to walk briskly as far as possible for a timed 6 minutes. Fatigue and dyspnoea before and after the walk test were assessed using the Borg scale (27).

Physical fitness was measured using a Tecumseh step-test (28) Participants were asked to step repeatedly on and off a step measuring 60x30x17.5 cm (length, width, height) for three minutes in time with a metronome set to 92 beats per minute (bpm). This corresponds to a rate of energy expenditure approximately 5 times the basal metabolic rate. (29)Heart rate were measured on the right arm immediately afterwards and then again in the seated position after three minutes recovery using an Omron 705CP device. Estimated peak oxygen consumption (peak $VO_2$) was calculated based on achieved heart rate in the immediate post-exercise period as described previously.(28)



For peer review only - http://bmjopen.bmj.com/site/about/guidelines.xhtml

Chronic study: Secondary outcome measures

Measures of cardiac structure and function were obtained as described under primary outcomes above and included left ventricular mass index, relative wall thickness, left atrial diameter, ejection fraction, mitral E/A ratio, s'(peak velocity during systole) and e'(peak velocity during early diastole).

Resting seated brachial and central blood pressure was measured after 5 minutes seated rest using a Pulsecor BP+ device (Uscom Ltd, Sydney, Australia) (30) starting with the left arm and then repeated on the right arm. The average of the final 2 of 3 blood pressure readings for the right arm were used, unless the average SBP was more than 10 mm Hg greater than the average in the left arm, in which case the left arm average readings were used as the measure of clinic BP. BP (standing) before, immediately after and after 3 minutes recovery following the step-test was measured using an Omron 705CP device on the right arm.

A Vicorder oscillometric device (SMT Medical Germany/Skidmore Medical UK) (31) was used to measure carotid-femoral pulse wave velocity (PWV).

Ambulatory blood pressure monitoring was conducted using the oscillometric Mobilograph device (NuMed Healthcare, UK) (32) with an appropriately sized cuff worn on the non-dominant arm to record central blood pressure and heart rate for a 24 hour period; measurements were taken half-hourly between 0700 and 2100 hours and hourly during the night. Ambulatory blood pressure and heart rate analyses included the daytime period from 0900-2100 hours and the night-time period from 0100-0600 excluding the waking and bedtime periods of the day as these periods represent times during which bed rest is inconsistent and, therefore, cannot be categorised reliably.(33)

Heart rate variability (HRV) and baroreceptor sensitivity (BRS) were measured according to a published protocol (34). Briefly, these were measured in the recumbent position for a 10-minute period. Beat to beat arterial BP was recorded non-invasively using a Finometer (FMS Amsterdam, Netherlands), and the ECG was monitored using a 3 lead ECG. Signals were post processed as described in detail previously (34). For HRV we calculated the mean R-R interval, and mean spectral powers in the low frequency (LF: 0.04-0.15 Hz) and high frequency (HF: 0.15-0.4 Hz) bands for the R-R intervals. Frequency domain BRS was calculated as the alpha index given by the square root of the ratio between averaged powers of R-R and systolic BP for each frequency.

Fasting bloods were analysed for glucose and lipids at baseline and 3-month follow-up. The HPA axis was assessed by salivary cortisol sampled at 5 points during the day pre-intervention and at 3 months follow-up as described for the acute study below. Salivary amylase, as a marker of sympathetic activity, was measured at 5 time points during the day, as described for cortisol.

The full extra-cranial carotid artery was examined for the presence of plaque using an iE33 ultrasound machine (Philips) equipped with a linear-array transducer (L11_3) with concurrent recording of 3-lead ECG over 3-5 cardiac cycles. Carotid intima-media thickness (IMT) was measured in the distal 1 cm of the left common carotid artery from three longitudinal planes (anterior, lateral and posterior) in a region free of plaque with a clearly identified double-line pattern. Plaque was defined according to the Mannheim consensus as a focal structure encroaching into the arterial lumen by at least 0.5 mm or 50% of the surrounding IMT value, or a region of IMT having a thickness >1.5 mm. Analyses were performed using a validated semi-automated programme (AMS-II).



For peer review only - http://bmjopen.bmj.com/site/about/guidelines.xhtml

The GeneActiv wrist-worn waterproof accelerometry device was fitted at the end of the pre-intervention and 3-month follow-up visits and worn for 3 days after each visit. Analysis of the data was performed using a validated algorithm at the University of Newcastle (35) to provide average body acceleration (metric milli g where g is gravity) on days with more than 16 hours of valid readings.

Self-completion questionnaires were administered pre-interventions and at the 3-month follow-up as follows:

The international physical activity questionnaire (IPAQ) long version was administered and analysed according to the IPAQ guidelines (http://www.ipaq.ki.se/scoring.pdf, accessed Aug 25th 2017)

A self-completion questionnaire included items regarding frequency of alcohol consumption, number of units consumed and changes in drinking habits. Similar questions were included regarding smoking habits. A food frequency questionnaire, previously used in the SABRE tri-ethnic cohort study(36) covered the previous 7 days.

EQ-5D™ (https://euroqol.org/) is a standardised instrument for use as a measure of health outcome. It provides a simple descriptive profile a visual analogue scale to indicate self-rated health and a health status score based on UK population norms (there is no set of scores based on Indian Asian populations).

The perceived stress 10 item self-completion scale (37) was completed together with questions regarding sleep quality, snoring and breathlessness at night.

Acute study: Yoga+usual care group on day of first yoga session

Primary outcome measures - blood pressure and heart rate at rest and following a 3-minute step-test and estimated peak oxygen consumption (peak $VO_2$) measured immediately post-exercise

Seated brachial blood pressure was measured after 5 minutes rest using an Omron 705CP device on the right arm. Blood pressure, heart rate at rest and following the 3-minute step-test were performed immediately before and after the first yoga session as described above for the chronic study; estimated peak $V0_2$ was also calculated. (28, 29)

Secondary outcome measures

Saliva samples for amylase and cortisol were collected by the participants at home using a Salivette (www.salimetrics.com) collection kit at 5 time points during the day pre-intervention (waking, waking plus 30 minutes, waking plus 90 minutes, waking plus 12 hours, bedtime). For the acute study, waking, waking plus 12 hours and bedtime samples were taken on the day of the first yoga session. The latter two sampling points therefore occurred after the first yoga session. Samples were analysed using using indirect enzyme-linked immunosorbent assay kits (Salimetrics Europe Ltd., Suffolk, UK).

Blinding of observers

Post-processing of echocardiograms, carotid ultrasound scans, accelerometry, ambulatory blood pressure, heart rate variability and baroreceptor sensitivity, blood and saliva analyses were all conducted by observers blinded to participants' identity and study group. Clinic BP, vascular measurements and



For peer review only - http://bmjopen.bmj.com/site/about/guidelines.xhtml

anthropometric measurements were conducted by clinic staff, who may have been aware of study group allocation, given the nature of the interventions.

Location where data were collected

Data were collected at the International Centre for Circulatory Health on the St Mary's campus of Imperial College London (UK).

Statistical analyses

Sample size and power

The sample size was estimated for the primary outcome measures for the chronic effects of yoga, i.e. E/e' echocardiography and 6-minute walk test. Previous studies have reported at least half a standard deviation benefit associated with yoga on diastolic function and exercise testing (38, 39) corresponding to a 1.1 improvement in E/e'(38, 39), and a study in people with preserved ejection fraction heart failure reported more than double this effect (-3.2) following a 3 month exercise programme.(20) For the 6-minute walk test, a distance of 40m was considered a clinically significant improvement in distance walked (40). This improvement was exceeded in a study of CR, where the distance walked increased by 62m. (41) In both cases these minimum important differences corresponded to approximately 0.5 standard deviations and the sample size was estimated to detect an effect of this magnitude. Statistical analyses were planned to use regression modelling to adjust final measures for baseline differences, thus improving the precision of estimates of treatment effect, and shrinking the sample size requirement.(42) The intraclass correlation coefficient (ICC) of the primary outcomes was ≥0.85 based on our own data (n = 10) and other observers'.(19) Using a conservative estimate of ICC = 0.70, and allowing for multivariable analysis, 33 completers were required in each arm of the study to detect a 0.5 standard deviation difference between groups (80% power and 5% significance). Thus, 40 people were recruited to each arm to allow for dropouts.

Statistical methods

Chronic study: summary descriptions of continuous pre-intervention characteristics are shown as means (95% CI) for Normally distributed data or as medians (95%CI of the median(CIM)) for non-Normally distributed variables or as number (%) for categorical variables. Pre-intervention characteristics are shown for the whole study group (Table 1) and for those who did and did not complete the study. (Table S1). Outcome analysis is restricted to those who attended the 3-month visit, and for the yoga group, additionally restricted to those who attended 18 out of the 24 yoga sessions, per protocol. A sensitivity analysis added 4 participants who did not complete the requisite number of yoga classes but who attended the 3-month study follow-up visit.

For the 3-minute step-test which was conducted in three stages pre- and post-intervention, repeated measures ANOVA models were used to determine differences by intervention arm and timing (pre-intervention and 3 months follow-up for the chronic study) and for the acute study (pre- and post-first yoga session). Repeated measures ANOVA models were also used for salivary amylase (log transformed) and cortisol measured 5 times on 3 days (yoga + usual care group) or 2 days (usual care group).

The remaining measures were analysed using robust regression models, (43) which are relatively efficient in the presence of outlier-prone error distributions. 3-month follow-up values were adjusted for the pre-intervention value of each Normally distributed measure, to provide adjusted mean (95%CI) values to



For peer review only - http://bmjopen.bmj.com/site/about/guidelines.xhtml

allow comparison with pre-intervention observations. Where data were not Normally distributed pre-intervention, median regression provided comparable 3-month (median (95% CI of the median)) follow-up values adjusted for the pre-intervention value. We show between group differences (95% CI) for all outcome measures, together with p values for primary outcome measures. Sensitivity analyses for primary outcomes included adjustment for informative baseline covariates (age, sex, diabetes, body mass index plus height for the 6 minute walk test). Between- and within-group differences in categorical secondary outcome measures were tabulated and tested using the chi square test.

For heart rate variability and baroreceptor sensitivity, we conducted sensitivity analyses that excluded the few participants who were not receiving beta-blocker medication.

P values are shown for primary outcome data only and statistical significance accepted as $p<0.05$. Statistical analyses were performed using STATA version 15 software.

**Results**

80 participants were recruited and randomly assigned in equal numbers to the yoga plus usual care and usual care groups. Pre-intervention, average age was 57.1 (95% CI: 54.9, 59.4), 69% were male and 64% were of South Asian origin. Diabetes was present in 36%. The majority were receiving statins (90%) and/or anti-hypertensive medication (95%). (Table 1)

Thirty-five participants in the usual care arm (63% South Asian) and 25 participants in the yoga arm (59% South Asian) completed the study. Greater loss to follow-up occurred in the yoga group, mostly due to unwillingness to continue with yoga classes - participants frequently citing ill health as a reason, although one participant withdrew from the study because of return to work. (Supplemental figure S1) Characteristics of those who completed the study and those who dropped out were similar pre-intervention. (Table S1) In addition to overall study dropout, several participants declined or were unable to undergo exercise testing either pre-or post-intervention, mostly due to mobility problems or elevated blood pressure (reasons are listed under Table 2).

No adverse events were reported. There was minimal change in the number and type of medications prescribed over the 3-month course of the study. (Tables 1 and S1)

<u>Chronic study</u>

<u>Primary outcomes</u>

<u>Left ventricular diastolic function</u>

At the three month follow-up, E/e' improved in both groups, but there was no evidence of yoga-related additional benefit in diastolic function (E/e': between group difference: yoga minus usual care:-0.40(-1.40, 0.61) (adjusted for pre-intervention values) p=0.4 (Table 2)

<u>6-minute walk test</u>

The total distance walked increased in both groups at 3-months follow-up, but there was no evidence of yoga-related additional benefit (between group difference yoga minus usual care: -7 (-40, 25) m, p = 0.7; Table 2). Distance walked per minute also increased post-intervention to a similar level in both groups and



For peer review only - http://bmjopen.bmj.com/site/about/guidelines.xhtml

there was no additional advantage related to yoga in the total number of minutes walked or in levels of fatigue and breathlessness. (Table 2)

3-minute step-test

The results of the 3 minute step-test at 3 month follow-up suggested some moderate improvements in immediate post-exercise BP, heart rate and peak $VO_2$ in both groups at follow-up, but there was no evidence of additional benefit associated with yoga. (Table 2)

Secondary outcomes

Other vascular measures

There was no evidence of yoga-related additional benefits for measures of clinic and ambulatory measures of brachial and central SBP at follow-up. Both groups showed improvements in resting brachial DBP and in resting central SBP. Pulse wave velocity was similar in the two groups at follow-up. (Table 3)

Carotid intima-media thickness

There was no evidence of additional yoga-related benefit on carotid IMT levels at 3 months. (Table 3)

Hypothalamic-Pituitary-Adrenal axis (HPA)

Salivary cortisol, as a marker of the HPA, decreased throughout the day in both groups pre-intervention and at 3 months follow-up. There was no evidence of additional yoga-related benefit compared with usual care alone. (Table 3)

Autonomic function

There was no evidence of additional yoga-related benefit compared with usual care alone on markers of heart rate variability baroreceptor sensitivity at 3 month follow-up and salivary amylase. (Table 4)

Metabolic measures

There was no evidence of additional yoga-related benefit compared with usual care alone at 3 months follow-up in glucose, total cholesterol, LDL cholesterol. (Table 3)

Anthropometrics

Both groups had slightly lower waist to hip ratios at follow-up than at baseline, but with no evidence of yoga-related additional benefit compared with usual care alone. (Table 3)

Other measures

Accelerometry over 3 days showed that the usual care group modestly increased levels and the yoga group maintained levels of physical activity during the follow-up period. Self-reported physical activity (IPAQ) increased in both groups, with no evidence of additional benefit from yoga compared with usual care alone. (Table 3)

Similarly the EQ5D measures of health status or self-rated health at follow-up did not show any convincing evidence of a treatment effect at follow-up, although there was a small increase in EQ5D health status based on UK population norms in people randomized to yoga compared with those receiving usual care.



For peer review only - http://bmjopen.bmj.com/site/about/guidelines.xhtml

For peer review only

The EQ5D self-rated health thermometer improved to equal extents in both yoga and usual care groups over the 3 month period. The yoga group had lower stress scores than the usual care group both pre-intervention and at follow-up and there was no convincing evidence of change in stress score in either treatment group. (Table 3)

There were very few current smokers at baseline or follow-up and there were no between- group differences or within group changes. There were no between-group differences or significant within-group changes at follow-up in self-reported hours and quality of sleep, in alcohol consumption or in consumption of fresh fruit and vegetables (not shown).

<u>Sensitivity analyses</u>

Sensitivity analyses of the primary outcomes which added those 4 participants who did not complete 18 yoga classes, but who did attend the 3 month follow-up clinic, did not alter findings. Likewise, exclusion of the few people who were not receiving beta blocker medication did not alter the findings for heart rate variability, baroreceptor sensitivity and salivary amylase.  Additional adjustment of primary outcome measures for selected informative  baseline covariates (age, sex, diabetes, body mass index (and height for the 6 minute walk test) did not alter conclusions, e.g. adjusted between group difference in E/e' was -0.18 (-1.28, 0.92) compared with -0.38(-1.38, 0.58) when adjusted only for baseline E/e'.  the three minute step test and six minute walk test findings were little changed on adjustment for these baseline covariates

<u>Acute study (yoga arm only)</u>

A 3-minute step-test was performed before and after the first yoga session  and blood pressure was measured pre-exercise, immediately post exercise and 3 minutes post-exercise. 27 participants undertook this test, 3 refused, 8 were unable to undertake exercise testing due to mobility problems and/or shortness of breath, one had unstable angina and in one case equipment failure resulted in loss of data. There was no convincing evidence of an acute effect of yoga on BP, heart rate or estimated peak $VO_2$. (Table 5a) Salivary cortisol and amylase were similar at the waking+12 hours and bedtime periods after the first yoga session compared with pre-intervention levels. (Table 5b)

<u>Discussion</u>

We show no additional cardiovascular benefit of a 3-month yoga intervention over and above usual care including CR in a randomised trial in people who had experienced an acute coronary event.  Specifically, there was no additional impact on our co-primary outcomes of E/e' or exercise capacity, nor on a wide range of other secondary outcomes including measures of  cardiac structure and function, brachial, central and ambulatory blood pressure, blood pressure and heart rate responses to exercise, estimated peak $VO_2$, carotid intima media thickness,  blood lipids and glucose, obesity measures including  fat mass and body mass index,  self-reported physical activity levels, distance walked in the 6 minute walk test alcohol, smoking and dietary intake.

Of the cardiovascular risk factors studied to date, blood pressure appears the most consistently beneficially affected by yoga,(8, 10, 44) with reports that reductions in blood pressure are similar to those obtained by anti-hypertensive medication.(45)  However, a community-based crossover study in India of non-pharmacological interventions showed that physical exercise (brisk walking for 50-60 minutes, 3-4 days a week for eight weeks) was a more effective method of reducing blood pressure, compared with yoga training or salt reduction, which both had similar smaller effects.(46)  More recently, a community



For peer review only - http://bmjopen.bmj.com/site/about/guidelines.xhtml

based randomised controlled trial in Sweden found no evidence of reductions in resting blood pressure due to a 3-month yoga intervention(47) - similar to our findings.

The acute effects of yoga on cardiovascular responses to exercise have not been well studied, although a study of 33 female college students in the USA reported that reduction in salivary cortisol following one hour sessions of power yoga, stretch yoga or control (watching an educational movie for one hour) were similar between interventions.(48) We saw little change in cortisol levels in the either group at 3-month follow-up or in the yoga group albeit later in the day following the first yoga session. At 3 months of follow-up, improvements in heart rate and estimated peak $VO_2$ were seen in both groups compared with the pre-intervention levels but there was no evidence of benefit from yoga. Likewise, both groups improved in terms of distance walked in the 6-minute walk test at 3 months of follow-up but addition of yoga to usual care had no effect compared with usual care alone. A USA based study in heart failure patients reported a 0.5 SD improvement in exercise tolerance (+17% in 9 patients with heart failure enrolled to an 8 week yoga programme and –7% in 10 patients enrolled to receive standard medical therapy alone(39)). The same study also showed greater improvements in quality of life in the yoga group (scores improved by 26% in the yoga group and by 3% in the standard medical therapy group(39)). Heart failure was relatively infrequent in our study participants, (less than 20% reported previously diagnosed heart failure and the median ejection fraction was 54% which is only slightly below the reported lower limit of the normal reference ranges for male and female Europeans (55.8% in men and 57.3% in women)(49)); but whether this can account for differences between study findings must remain speculative. There was a small improvement in the EQ5D measure of health status based on UK population norms in the yoga group in our study but the 95% confidence interval of the difference included the null.

Measures of HRV may be more sensitive to subtle changes than traditional tests of autonomic function, and improvements associated with yoga training or tai chi have been observed in systematic reviews for a number of parameters;(50, 51) however, we found little evidence for any yoga-related benefits in these parameters over 3 months.

Yoga has been variously shown to reduce fasting glucose and glycated haemoglobin, insulin, total and LDL cholesterol, triglyceride and weight, even in those without diabetes (52), although not all studies have shown a consistent benefit across these risk factors (7, 53, 54). We found no evidence of yoga-related benefits in blood lipids, glucose or obesity measures. Statin use was high (89%+) in both our study groups which may limit the measurable effect of interventions on lipid levels.

Impacts of yoga on subclinical and clinical cardiovascular disease have been inconsistently studied, making it difficult to place in context our findings of no evidence of improvement in E/e' due to yoga, although E/e' appeared lower in both groups at follow-up compared with baseline. Although our participants had pre-intervention levels of E/e' that could be considered 'normal', it is important to note that increased cardiovascular risk increases linearly across the entire range of E/e' and there is evidence that exercise can improve diastolic function even in healthy individuals. (55) Yoga in comparison with walking has been reported to improve cardiac function in older hypertensive individuals in India (56) and, in a high risk subgroup of older individuals in the USA. A multimodality intervention including yoga reduced carotid intima media thickness to a greater extent than usual care or dietary and exercise advice; (53) however, no effect of the intervention was seen in the whole group, and numbers in the high risk subgroups were small.

As noted earlier, there is a general difficulty in comparing studies due to the wide variation in study designs and populations. A recent systematic review of 306 randomised controlled trials of yoga found that 91% reached positive conclusions (57). The authors confirmed difficulty in comparison of results



For peer review only - http://bmjopen.bmj.com/site/about/guidelines.xhtml

For peer review only

across all trials, due to the common lack of *a priori* defined primary outcomes and appropriate group comparisons.

Strengths and limitations: This is the first study to our knowledge to adopt a comprehensive approach to measuring cardiovascular clinical and subclinical outcomes in response to a yoga intervention. It is also unusual in studying outcomes in a real world setting in an older group of people eligible for CR following an acute coronary event; however the trial was not designed to establish whether yoga may have benefits in terms of cardiovascular events or angina, for this much larger studies would be required. Also for some outcomes, such as IMT, it is likely that 3 months is too short a time to observed substantial regression. Dropout in the yoga arm of the study exceeded that in the usual care arm, (15 and 5 respectively), possibly reflecting the dual burden of attending both yoga training and usual CR. Consequently, the study will not have achieved the planned statistical power, although given the measured effect sizes and confidence intervals, we believe if there are benefits of yoga on the measured outcomes, they are likely to be small. We did not adjust for multiple testing as we had identified *a priori* relevant primary outcomes for the trial, but in practice adjustment for multiple testing would not have altered our interpretation given the null findings. Cardiac rehabilitation is standard care following an acute coronary event in the UK, thus ethical reasons prevented comparison of yoga-based CR directly with usual CR alone - hence this study cannot tell us about the potential of yoga as an alternative to traditional CR. Our findings also cannot be generalized to other conditions that may benefit from cardiac rehabilitation, such as heart failure, post-valve replacement, stable angina pectoris, or symptomatic peripheral artery disease.(25) It should also be noted that our study was designed as a mechanistic study to complement a larger (around 4000 patients) Indian Council for Medical Research and Medical Research Council, UK funded study of yoga as a primary method of CR in India, which may shed light on some of the issues discussed above. (24)

Conclusion: In this UK-based randomised controlled trial of yoga classes plus usual CR compared with usual rehabilitation only in people who had experienced an acute coronary event, we found no evidence that the yoga programme conferred any additional benefits to cardiovascular or neuroendocrine health compared with usual CR care at 3 months of follow-up. We suggest that usual care CR programmes in the UK, which include exercise, and optimisation of medical therapy may leave little additional scope for added benefits from a further intervention such as yoga.

Acknowledgements: We thank the cardiac rehabilitation teams at Ealing Hospital and the Flexi-Heart cardiac rehabilitation teams in Harrow and Brent for their help in identifying suitable participants; Daniel Key for building the study databases and managing the randomisation processes; Paula Carvelli, April McGowan for their help in recruitment and clinical measurements; Nadia do Couto Franscisco and Karikahan Manoharan for echocardiography measurements and analysis; Vincent van Hees (www.esciencecenter.nl) for performing analyses of GeneActiv physical activity data; and the participants for their valuable support.

Author contributions: SK, NC, AH, SE and IR were involved in the conception and design of the study. CT was responsible for data acquisition. BS, KC, SK and CT designed the yoga intervention. NS and PW contributed to data acquisition by performing salivary analyses. TT carried out the statistical analyses and wrote the first draft, with support from SK, AH, NC. All authors critically reviewed and revised all drafts and approved and are accountable for the accuracy and integrity of the final version. The corresponding author attests that all listed authors meet authorship criteria and that no others meeting the criteria have been omitted. TT is the guarantor.



For peer review only - http://bmjopen.bmj.com/site/about/guidelines.xhtml

Transparency statement: The lead author (TT) affirms that the manuscript is an honest, accurate, and transparent account of the study being reported; that no important aspects of the study have been omitted; and that any discrepancies from the study as originally planned and registered have been explained.

Funding and role of the funding source: The study was funded by the UK Medical Research Council (MR/J000175/1). The funders and study sponsors have played no role in the study design; in the collection, analysis, and interpretation of data; in the writing of the report; nor in the decision to submit the article for publication. The researchers were independent from the funders and sponsors and all authors had full access to all of the data (including statistical reports and tables) in the study and can take responsibility for the integrity of the data and the accuracy of the data analysis.

Competing Interest statement: All authors have completed the ICMJE uniform disclosure form at http://www.icmje.org/coi_disclosure.pdf and declare no support from any organisation for the submitted work, no financial relationships with any organisations that might have an interest in the submitted work in the previous three years and no other relationships or activities that could appear to have influenced the submitted work.

Ethical approval was granted by Camberwell St Giles Research Ethics Committee (Ref: 12/LO/0597)

Data sharing statement: De-identified participant data are available upon reasonable request to Professor Nish Chaturvedi (n.chaturvedi@ucl.ac.uk)

References

1. Feuerstein G. The yoga tradition. Prescott: Hohm Press; 1998.
2. Choices N. A Guide to Yoga: Department of Health and Social Care, UK Government; [Available from: https://www.nhs.uk/live-well/exercise/guide-to-yoga/.
3. Chong CS, Tsunaka M, Tsang HW, Chan EP, Cheung WM. Effects of yoga on stress management in healthy adults: A systematic review. Alternative therapies in health and medicine. 2011;17(1):32-8.
4. Cramer H, Lauche R, Langhorst J, Dobos G. Yoga for depression: a systematic review and meta-analysis. Depression and anxiety. 2013;30(11):1068-83.
5. Cramer H, Langhorst J, Dobos G, Lauche R. Associated Factors and Consequences of Risk of Bias in Randomized Controlled Trials of Yoga: A Systematic Review. PLoS One. 2015;10(12):e0144125.
6. Cramer H, Lauche R, Haller H, Steckhan N, Michalsen A, Dobos G. Effects of yoga on cardiovascular disease risk factors: a systematic review and meta-analysis. Int J Cardiol. 2014;173(2):170-83.
7. Innes KE, Vincent HK. The influence of yoga-based programs on risk profiles in adults with type 2 diabetes mellitus: a systematic review. Evid Based Complement Alternat Med. 2007;4(4):469-86.
8. Jayasinghe SR. Yoga in cardiac health (a review). Eur J Cardiovasc Prev Rehabil. 2004;11(5):369-75.
9. Ross A, Thomas S. The health benefits of yoga and exercise: a review of comparison studies. J Altern Complement Med. 2010;16(1):3-12.
10. Yang K. A review of yoga programs for four leading risk factors of chronic diseases. Evid Based Complement Alternat Med. 2007;4(4):487-91.
11. Kwong JS, Lau HL, Yeung F, Chau PH. Yoga for secondary prevention of coronary heart disease. Cochrane Database Syst Rev. 2015(7):Cd009506.



For peer review only - http://bmjopen.bmj.com/site/about/guidelines.xhtml

12. Cramer H, Langhorst J, Dobos G, Lauche R. Yoga for metabolic syndrome: A systematic review and meta-analysis. Eur J Prev Cardiol. 2016;23(18):1982-93.
13. Anderson L, Thompson DR, Oldridge N, Zwisler AD, Rees K, Martin N, et al. Exercise-based cardiac rehabilitation for coronary heart disease. Cochrane Database Syst Rev. 2016(1):Cd001800.
14. NACR. The National Audit of Cardiac Rehabilitation—Annual Statistical Report 2014.; 2015.
15. Nagueh SF, Appleton CP, Gillebert TC, Marino PN, Oh JK, Smiseth OA, et al. Recommendations for the evaluation of left ventricular diastolic function by echocardiography. J Am Soc Echocardiogr. 2009;22(2):107-33.
16. Hillis GS, Moller JE, Pellikka PA, Gersh BJ, Wright RS, Ommen SR, et al. Noninvasive estimation of left ventricular filling pressure by E/e' is a powerful predictor of survival after acute myocardial infarction. Journal of the American College of Cardiology. 2004;43(3):360-7.
17. Sandercock G, Hurtado V, Cardoso F. Changes in cardiorespiratory fitness in cardiac rehabilitation patients: a meta-analysis. Int J Cardiol. 2013;167(3):894-902.
18. Bellet RN, Adams L, Morris NR. The 6-minute walk test in outpatient cardiac rehabilitation: validity, reliability and responsiveness--a systematic review. Physiotherapy. 2012;98(4):277-86.
19. Hanson LC, McBurney H, Taylor NF. The retest reliability of the six-minute walk test in patients referred to a cardiac rehabilitation programme. Physiotherapy research international : the journal for researchers and clinicians in physical therapy. 2012;17(1):55-61.
20. Edelmann F, Gelbrich G, Dungen HD, Frohling S, Wachter R, Stahrenberg R, et al. Exercise training improves exercise capacity and diastolic function in patients with heart failure with preserved ejection fraction: results of the Ex-DHF (Exercise training in Diastolic Heart Failure) pilot study. J Am Coll Cardiol. 2011;58(17):1780-91.
21. Schultz MG, Otahal P, Cleland VJ, Blizzard L, Marwick TH, Sharman JE. Exercise-induced hypertension, cardiovascular events, and mortality in patients undergoing exercise stress testing: a systematic review and meta-analysis. Am J Hypertens. 2013;26(3):357-66.
22. Beatty AL, Schiller NB, Whooley MA. Six-minute walk test as a prognostic tool in stable coronary heart disease: data from the heart and soul study. Arch Intern Med. 2012;172(14):1096-102.
23. Filipovsky J, Ducimetiere P, Safar ME. Prognostic significance of exercise blood pressure and heart rate in middle-aged men. Hypertension. 1992;20(3):333-9.
24. Chandrasekaran AM, Kinra S, Ajay VS, Chattopadhyay K, Singh K, Singh K, et al. Effectiveness and cost-effectiveness of a Yoga-based Cardiac Rehabilitation (Yoga-CaRe) program following acute myocardial infarction: Study rationale and design of a multi-center randomized controlled trial. Int J Cardiol. 2019.
25. BACPR. The BACPR Standards and Core Components for Cardiovascular Disease Prevention and Rehabilitation 2012. 2012.
26. Park CM, Tillin T, March K, Ghosh AK, Jones S, Wright A, et al. Hyperglycemia has a greater impact on left ventricle function in South Asians than in Europeans. Diabetes Care. 2014;37(4):1124-31.
27. Borg GA. Psychophysical bases of perceived exertion. Med Sci Sports Exerc. 1982;14(5):377-81.
28. Hughes AD, Chaturvedi N. Estimation of Maximal Oxygen Consumption and Heart Rate Recovery Using the Tecumseh Sub-Maximal Step Test and their Relationship to Cardiovascular Risk Factors. Artery Res. 2017;18:29-35.
29. Montoye HJ, Willis PW, 3rd, Cunningham DA, Keller JB. Heart rate response to a modified Harvard step test: males and females, age 10-69. Res Q. 1969;40(1):153-62.
30. Park CM, Korolkova O, Davies JE, Parker KH, Siggers JH, March K, et al. Arterial pressure: agreement between a brachial cuff-based device and radial tonometry. Journal of hypertension. 2014;32(4):865-72.
31. Pucci G, Cheriyan J, Hubsch A, Hickson SS, Gajendragadkar PR, Watson T, et al. Evaluation of the Vicorder, a novel cuff-based device for the noninvasive estimation of central blood pressure. Journal of hypertension. 2013;31(1):77-85.
32. Weiss W, Gohlisch C, Harsch-Gladisch C, Tolle M, Zidek W, van der Giet M. Oscillometric estimation of central blood pressure: validation of the Mobil-O-Graph in comparison with the SphygmoCor device. Blood pressure monitoring. 2012;17(3):128-31.



For peer review only - http://bmjopen.bmj.com/site/about/guidelines.xhtml

33. Staessen J, Bulpitt CJ, Fagard R, Mancia G, O'Brien ET, Thijs L, et al. Reference values for the ambulatory blood pressure and the blood pressure measured at home: a population study. Journal of human hypertension. 1991;5(5):355-61.
34. Bathula R, Hughes AD, Panerai R, Potter J, Thom SA, Francis DP, et al. Indian Asians have poorer cardiovascular autonomic function than Europeans: this is due to greater hyperglycaemia and may contribute to their greater risk of heart disease. Diabetologia. 2010;53(10):2120-8.
35. van Hees VT, Fang Z, Langford J, Assah F, Mohammad A, da Silva IC, et al. Autocalibration of accelerometer data for free-living physical activity assessment using local gravity and temperature: an evaluation on four continents. J Appl Physiol (1985 ). 2014;117(7):738-44.
36. Tillin T, Forouhi NG, McKeigue PM, Chaturvedi N. Southall And Brent REvisited: Cohort profile of SABRE, a UK population-based comparison of cardiovascular disease and diabetes in people of European, Indian Asian and African Caribbean origins. Int J Epidemiol. 2012;41(1):33-42.
37. Cohen S, Kamarck T, Mermelstein R. A global measure of perceived stress. J Health Soc Behav. 1983;24(4):385-96.
38. Konar D, Latha R, Bhuvaneswaran JS. Cardiovascular responses to head-down-body-up postural exercise (Sarvangasana). Indian J Physiol Pharmacol. 2000;44(4):392-400.
39. Pullen PR, Nagamia SH, Mehta PK, Thompson WR, Benardot D, Hammoud R, et al. Effects of yoga on inflammation and exercise capacity in patients with chronic heart failure. J Card Fail. 2008;14(5):407-13.
40. Schrover R, Evans K, Giugliani R, Noble I, Bhattacharya K. Minimal clinically important difference for the 6-min walk test: literature review and application to Morquio A syndrome. Orphanet journal of rare diseases. 2017;12(1):78.
41. Wright DJ, Khan KM, Gossage EM, Saltissi S. Assessment of a low-intensity cardiac rehabilitation programme using the six-minute walk test. Clinical rehabilitation. 2001;15(2):119-24.
42. Borm GF, Fransen J, Lemmens WA. A simple sample size formula for analysis of covariance in randomized clinical trials. Journal of clinical epidemiology. 2007;60(12):1234-8.
43. Huber PJ, Ronchetti E. Robust statistics. 2nd ed. ed. Oxford: Wiley-Blackwell; 2009.
44. Hagins M, States R, Selfe T, Innes K. Effectiveness of yoga for hypertension: systematic review and meta-analysis. Evidence-based complementary and alternative medicine : eCAM. 2013;2013:649836.
45. Murugesan R, Govindarajulu N, Bera TK. Effect of selected yogic practices on the management of hypertension. Indian J Physiol Pharmacol. 2000;44(2):207-10.
46. Subramanian H, Soudarssanane MB, Jayalakshmy R, Thiruselvakumar D, Navasakthi D, Sahai A, et al. Non-pharmacological Interventions in Hypertension: A Community-based Cross-over Randomized Controlled Trial. Indian J Community Med. 2011;36(3):191-6.
47. Wolff M, Rogers K, Erdal B, Chalmers JP, Sundquist K, Midlov P. Impact of a short home-based yoga programme on blood pressure in patients with hypertension: a randomized controlled trial in primary care. Journal of human hypertension. 2016;30(10):599-605.
48. Sullivan M, Carberry A, Evans ES, Hall EE, Nepocatych S. The effects of power and stretch yoga on affect and salivary cortisol in women. Journal of health psychology. 2017:1359105317694487.
49. Kou S, Caballero L, Dulgheru R, Voilliot D, De Sousa C, Kacharava G, et al. Echocardiographic reference ranges for normal cardiac chamber size: results from the NORRE study. Eur Heart J Cardiovasc Imaging. 2014;15(6):680-90.
50. Zou L, Sasaki JE, Wei GX, Huang T, Yeung AS, Neto OB, et al. Effects of Mind(-)Body Exercises (Tai Chi/Yoga) on Heart Rate Variability Parameters and Perceived Stress: A Systematic Review with Meta-Analysis of Randomized Controlled Trials. Journal of clinical medicine. 2018;7(11).
51. Posadzki P, Kuzdzal A, Lee MS, Ernst E. Yoga for Heart Rate Variability: A Systematic Review and Meta-analysis of Randomized Clinical Trials. Applied psychophysiology and biofeedback. 2015;40(3):239-49.
52. Bijlani RL, Vempati RP, Yadav RK, Ray RB, Gupta V, Sharma R, et al. A brief but comprehensive lifestyle education program based on yoga reduces risk factors for cardiovascular disease and diabetes mellitus. J Altern Complement Med. 2005;11(2):267-74.
53. Fields JZ, Walton KG, Schneider RH, Nidich S, Pomerantz R, Suchdev P, et al. Effect of a multimodality natural medicine program on carotid atherosclerosis in older subjects: a pilot trial of Maharishi Vedic Medicine. Am J Cardiol. 2002;89(8):952-8.



For peer review only

For peer review only - http://bmjopen.bmj.com/site/about/guidelines.xhtml

54. Mahajan AS, Reddy KS, Sachdeva U. Lipid profile of coronary risk subjects following yogic lifestyle intervention. Indian Heart J. 1999;51(1):37-40.
55. Levy WC, Cerqueira MD, Harp GD, Johannessen KA, Abrass IB, Schwartz RS, et al. Effect of endurance exercise training on heart rate variability at rest in healthy young and older men. The American journal of cardiology. 1998;82(10):1236-41.
56. Patil SG, Patil SS, Aithala MR, Das KK. Comparison of yoga and walking-exercise on cardiac time intervals as a measure of cardiac function in elderly with increased pulse pressure. Indian Heart J. 2017;69(4):485-90.
57. Cramer H, Lauche R, Langhorst J, Dobos G. Are Indian yoga trials more likely to be positive than those from other countries? A systematic review of randomized controlled trials. Contemp Clin Trials. 2015;41:269-72.

For peer review only



For peer review only - http://bmjopen.bmj.com/site/about/guidelines.xhtml

Table 1: Pre-intervention characteristics by randomisation group (unadjusted)

| N(%) or means(95%CI) unless otherwise stated | Yoga + usual care | Usual Care |
|---|---|---|
| Pre-intervention | N=40 | N=40 |
| Ethnicity: South Asian | 25(63%) | 26(65%) |
| Sex: Male | 28(70%) | 26(67%) |
| Age: years | 57.4(54.1, 60.7), range(35, 77) | 56.9(53.8, 60.0), range(35, 78) |
| Days since coronary event | 50(43, 57) | 59(53, 65) |
| Diabetes* (self report of physician diagnosis/anti-diabetic medication) | 15(38%) | 14 (35%) |
| Hypertension*( self report of physician diagnosis) | 29/37 (78%) | 25/37(68%) |
| Heart Failure* (self report of physician diagnosis) | 7/29(19%) | 7/29(19%) |
| Antihypertensive medications* | 39(98%) | 36(90%) |
| Number of antihypertensive medications, median(95%CI)* | 3(3,3) | 3(3,3) |
| Beta blockers* | 33(83%) | 32(80%) |
| Statins* | 36(90%) | 36(90%) |
| Current smoker/ex/never smoker, number* | 4/14/19 | 1/14/24 |
| Alcohol: never/ever drinkers, number* | N=36<br>Never drinkers: 13<br>Ever drinkers: 23 | N=35<br>Never drinkers: 10<br>Ever drinkers: 25 |
| Units/week (ever drinkers)*, median(95% CI) | 2.5(0, 10) | 4(1, 7) |
| Currently employed* | N=35<br>15(43%) | N=32<br>15(47%) |

*self-reported, N=number of responses to questionnaire item if incomplete

For peer review only

Table 2: Chronic study: **Primary outcomes**: Recruitment and three month follow-up (includes only those who attended study clinics at both time points and attended at least 18 classes if in the yoga group: N=35 in usual care group and N=25 in yoga group, unless otherwise stated)

| | Pre-intervention Means(95%CI) | | 3 months follow-up Means(95%CI), adjusted for pre-intervention levels | | Between group difference adjusted for pre-intervention levels (95%CI) | P value for between group difference, adjusted for pre-intervention levels |
|---|---|---|---|---|---|---|
| | Yoga + usual care | Usual care | Yoga + usual care | Usual care | Yoga minus usual care | |
| **Diastolic function** | N=25 | N=33* | N=25 | N=33* | | |
| E/e'. Median(95%CI) | 9.74(8.37, 11.12) | 8.72(7.76, 9.68) | 8.81(8.33, 9.29) | 8.26(7.79, 8.74) | -0.40 (-1.38, 0.58) | 0.4 |
| **6-minute walk test** | N=19*** | N=30** | N=19*** | N=30** | | |
| Total Distance, m | 462 (449, 517) | 442 (402,482) | 488 (463, 513) | 491 (471, 512) | -7 (-39, 26) | 0.7 |
| Total minutes walked | 6.0(6.0, 6.0) | 5.8(5.4, 6.0) | 5.9(5.7, 6.1) | 5.8(5.6, 6.0) | 0.1(-0.3, 0.5) | 0.5 |
| Distance, m/minute | 77(72, 82) | 77(71, 82) | 81(78, 83) | 81(78, 83) | 0.8(-4.6, 6.2) | 0.8 |
| Fatigue (Borg scale: 0-10) | | | | | | |
| Pre test | 0.2 (0, 0.7) | 0.2 (0, 0.5) | 0.02 (-0.03, 0.07) | 0 (0, 0.06) | 0.07 (-0.03, 0.2) | 0.17 0.9 |
| Post-test | 0.6 (0, 1.4) | 0.7 (0.03, 1.4) | 0.08 (-0.06, 0.20) | 0.08 (-0.04, 0.19) | -0.01 (-0.3,0.2) | |
| Dyspnoea (Borg Scale: 0-10) | | | | | | |
| Pre-test | 0 (0, 0) | 0.07(0, 0.23) | 0(0, 0.09) | 0.04(0, 0.10) | -0.04(-0.1, 0.07) | 0.5 |
| Post-test | 0.6(0, 1.2) | 1.0(0.3, 1.7) | 0.2(-0.3, 0.8) | 0.6(0.2, 1.0) | -0.2(-0.9, 0.5) | 0.5 |
| **Response to exercise:3-minute step test** | N=18*** | N=30** | N=18*** | N=30** | | |
| Pre-step test | | | | | | |
| Brachial SBP, mm Hg | 140 (134, 146) | 135 (130, 140) | 13 8(133, 140) | 131 (126, 136) | 6 (-5, 18) | 0.3 |
| Brachial DBP, mm Hg | 83 (80, 85) | 80 (78, 83) | 82 (79, 84) | 79 (77, 82) | 2 (-4, 9) | 0.4 |
| Heart rate, bpm | 60(56, 64) | 62(59, 66) | 58(54, 61) | 60(57, 63) | -2(-8,5) ) | 0.6 |
| Immediately post-step test | | | | | | |
| Brachial SBP, mm Hg | 161 (155, 167) | 152 (147, 157) | 156 (151, 162) | 149 (145, 155) | 2 (-10, 14) | 0.7 |
| Brachial DBP, mm Hg | 85(82, 87) | 80(78, 83) | 77(74, 80) | 77(74, 79) | -1(-8, 5) | 0.7 |
| Heart rate, bpm | 89 (85, 93) | 89(86,93) | 81(77, 85) | 85(82, 88) | -4(-11, 3) | 0.2 |
| Peak $VO_2$ | N=14 | N=27 | N=14 | N=27 | | |
| ml/min/kg | 35.7 | 38.2 | 41.3 | 42.6 | 0.4 | 0.9 |



For peer review only - http://bmjopen.bmj.com/site/about/guidelines.xhtml

| | Pre-intervention Means(95%CI) | | 3 months follow-up Means(95%CI), adjusted for pre-intervention levels | | Between group difference adjusted for pre-intervention levels (95%CI) | P value for between group difference, adjusted for pre-intervention levels |
|---|---|---|---|---|---|---|
| | Yoga + usual care | Usual care | Yoga + usual care | Usual care | Yoga minus usual care | |
| | (31.5, 40.0) | (34.3, 42.1) | (38.0, 44.6) | (39.3, 45.8) | (-5.9, 6.6) | |
| 3 minutes post-step test | | | | | | |
| Brachial SBP, mm Hg | 143 (137, 149) | 134 (129, 139) | 144 (138, 150) | 136 (131, 140) | 4(-8, 15) | 0.6 |
| Brachial DBP, mm Hg | 83(80, 86) | 80(78, 83) | 80(77, 83) | 79(77, 82) | -1(-3, 9) | 0.8 |
| Heart rate, bpm | 66(62, 70) | 65(61, 68) | 61 (57, 65) | 60(58, 64) | 0(-7, 7) | 1.0 |

*2 missing cases due to error readings
**5 missing cases: test not performed due to poor mobility(3), recent myocardial infarction(1), refused(1)
***7 missing cases: test not performed due to poor mobility(2), elevated blood pressure(>180/100mm Hg)(4), refused step-test(1)

For peer review only



For peer review only - http://bmjopen.bmj.com/site/about/guidelines.xhtml

Table 3: Chronic study: **Secondary outcomes**: Pre-intervention and 3-month follow-up (includes only those who attended study clinics at both time points and attended at least 18 classes if in the yoga group).
N=35 in usual care group and N=25 in yoga group, unless otherwise stated

| | Pre-intervention Means (95%CI) unless stated otherwise | | 3 months follow-up, adjusted for pre-intervention levels. Means (95%CI) unless stated otherwise | | Between group difference adjusted for pre-intervention levels (95% CI) |
|---|---|---|---|---|---|
| | Yoga + usual care | Usual care | Yoga + usual care | Usual care | Yoga minus usual care |
| **Cardiac structure** | | | | | |
| Left ventricular mass indexed to height $^{2.7}$, g/m $^{2.7}$, median(95%CI) | 40.1 (36.6, 51.2) | 38.7 (35.6, 43.5) | 39.4 (36.2, 42.5) | 39.3 (36.1, 42.5) | 3.4 (-3.3, 10.1) |
| Relative wall thickness, median(95%CI) | 0.45 (0.38, 0.48) | 0.42 (0.40, 0.47) | 0.41 (0.39, 0.44) | 0.42 (0.39, 0.44) | -0.01 (-0.07, 0.04) |
| Left atrial diameter indexed to height, cm/m | 2.2 (2.2, 2.3) | 2.3 (2.2, 2.4) | 2.2 (2.2, 2.3) | 2.2 (2.2, 2.3) | -0.08 (-0.21, 0.05) |
| **Cardiac function** | | | | | |
| Ejection fraction, %, median(95%CI) | 54(44, 68) | 54(44, 64) | 54(44, 63) | 54(45, 63) | -0.5(-19, 18) |
| Mitral E:A ratio(of peak velocity in early diastole to peak velocity in late diastole, median(95%CI) | 1.02 (0.97, 1.12) | 1.16 (0.95, 1.29) | 1.09 (1.01, 1.18) | 1.14 (1.07, 1.23) | -0.01 (-0.20, 0.19) |
| s', peak velocity during systole, cm/sec, median(95%CI) | 7.11 (6.49, 8.25) | 7.31 (6.35, 7.64) | 7.25 (6.90, 7.59) | 7.16 (6. 82, 7.51) | 0.10 (-0.56, 0.75) |
| e', peak velocity during early diastole, cm/sec, median(95%CI) | 7.56 (6.77, 9.35) | 8.57 (7.58, 9.44) | 8.20 (7.73, 8.67) | 8.45 (7.98, 8.92) | 0.14 (-0.72, 0.99) |
| **Resting blood pressure and heart rate** | N=25 | N=35 | N=25 | N=35 | |
| Heart rate, beats per minute(bpm) | 60(55, 64) | 64(60, 68) | 59(57, 61) | 61(60, 63) | -0.4(-4, 3) |
| Brachial SBP, mm Hg | 134 (125, 143) | 126(120, 133) | 127(124, 130) | 122(119, 125) | -2(-8, 4) |
| Brachial DBP, mm Hg | 77(74, 80) | 74(72, 76) | 75(73, 76) | 73(71, 74) | -0.8(-3, 2) |
| Central SBP, mm Hg | 128(119, 137) | 120(114, 125) | 122(119, 125) | 117(114, 120) | -1(-8, 6) |
| **24 hour ambulatory blood pressure** | N=20 | N=30 | N=20 | N=30 | |
| Average day central SBP, mm Hg | 115 (108, 122) | 113 (109, 116) | 113 (110, 116) | 112 (109, 114) | 2(-4, 8) |
| Average night central SBP, mm Hg | 104(96, 112) | 104(98, 110) | 104(100,108) | 104(100, 108) | 1(-7, 10) |
| Average day heart rate, bpm | 65(60, 69) | 68(65, 71) | 64(63, 66) | 67(65, 69) | 2(-2, 5) |
| Average night heart rate, bpm | 59(54, 63) | 64(60, 67) | 59(57, 61) | 63(61, 65) | 4(-0.4, 7) |
| **Pulse wave velocity**, m/sec median(95%CI) | N=25 9.03(8.14, 9.67) | N=32 8.63(8.27, 9.10) | N=25 9.02(8.47, 9.59) | N=32 8.75(8.28, 9.22) | 0.5(-0.7, 1.7) |
| **Carotid intima media thickness (CIMT)**. Far wall, mm, maximum of means (without plaque &CIMT<1.5mm) | N=18 0.754 (0.691, 0.816) | N=28 0.746 (0.681, 0.810) | N=18 0.764 (0.699, 0.830) | N=28 0.761 (0.696, 0.827) | -0.1 (-0.2, 0.1) |
| **Bloods, fasting, medians(95%CIM)** | N=25 | N=31 | N=25 | N=31 | |
| Triglycerides, mmol/l | 1.10 (0.94, 1.40) | 1.03 (0.96, 12.4) | 1.11 (1.01, 1.20) | 1.10 (1.00, 1.19) | -0.04 (-0.24, 0.17) |



For peer review only - http://bmjopen.bmj.com/site/about/guidelines.xhtml

For peer review only

| | Pre-intervention Means (95%CI) unless stated otherwise | | 3 months follow-up, adjusted for pre-intervention levels. Means (95%CI) unless stated otherwise | | Between group difference adjusted for pre-intervention levels (95% CI) |
|---|---|---|---|---|---|
| | Yoga + usual care | Usual care | Yoga + usual care | Usual care | Yoga minus usual care |
| HDL cholesterol, mmol/l | 0.91 (0.84, 1.07) | 0.91 (0.79, 1.01) | 1.02 (0.97, 1.06) | 0.97 (0.92, 1.02) | 0.05 (-0.04, 0.14) |
| Total cholesterol, mmol/l | 3.00 (2.80, 3.50) | 3.20 (2.90, 3.60) | 3.30 (3.18, 3.41) | 3.36 (3.25, 3.47) | 0.07 (-0.29, 0.15) |
| Cholesterol:HDL ratio | 3.33 (2.82, 3.67) | 3.40 (3.23, 3.78) | 3.31 (3.13, 3.48) | 3.52 (3.34, 3.69) | -0.06 (-0.38, 0.26) |
| LDL cholesterol, mmol/l | 1.59 (1.35, 1.77) | 1.60 (1.54, 1.73) | 1.76 (1.66, 1.87) | 1.81 (1.71, 1.92) | 0.01 (-0.19, 0.20) |
| Glucose, mmol/l | 5.40 (4.90, 6.0) | 5.50 (4.90, 6.20) | 5.78 (5.62, 5.94) | 5.68 (5.52, 5.84) | 0.05 (-0.23, 0.33) |
| **Anthropometrics** | N=25 | N=35 | N=25 | N=35 | |
| Body mass indexl, kg/m$^2$, median(95%CI) | 27.6 (25.1, 29.5) | 27.2 (25.3, 29.6) | 27.6 (27.4, 27.9) | 27.5 (27.3, 27.7) | 0(-0.5, 0.5) |
| Waist:hip ratio | 0.99 (0.96, 1.03) | 0.99 (0.96, 1.02) | 0.98 (0.97, 0.99) | 0.98 (0.97, 0.98) | 0.01 (-0.01, 0.02) |
| Fat mass percent | 28(25, 32) | 30(27, 33) | 28(27, 28) | 30(29, 30) | 0.2(-1, 2) |
| **HPA axis** | | | | | |
| **Salivary cortisol nmol/L** | N=23 | N=29 | N=23 | N=29 | |
| Waking | 11.2 (8.5,13.8) | 12.6 (10.3,14.9) | 11.2 (8.7, 13.7) | 12.5 (10.4, 14.7) | -1.3 (-4.2, 1.6) |
| Waking+30 minutes | 10.6 (8.0, 13.3) | 16.0(13.6, 18.3) | 10.4 (8.0, 12.9) | 12.9 (10.7, 15.0) | -2.5 (-5.4, 10.5) |
| Waking +1hrs 30 minutes | 5.2(2.5, 7.8) | 8.0(5.6, 10.4) | 8.0(5.6, 10.4) | 7.5(5.3, 9.7) | 0.5(-2.4, 3.4) |
| Waking +12 hours | 3.9(1.2, 6.7) | 3.2(0.8, 5.7) | 4.1(1.7, 6.6) | 2.5(0.3, 4.7) | 1.6(-1.4, 4.5) |
| Bedtime | 2.3(0, 5.0) | 3.1(0.7, 5.5) | 3.7(1.3, 6.2) | 2.2(0.04, 4.3) | 1.6(-1.3, 4.5) |
| **Exercise/physical activity** | | | | | |
| **Average body acceleration over 3 days**, milli g (GeneActiv) | N=20 25.1 (20.4, 29.8) | N=28 22.5 (19.5, 25.5) | N=20 24.9 (22.7, 27.1) | N=28 23.5 (21.3, 25.7) | -4.0 (-8.3, 0.23) |
| **International Physical Activity Questionnaire(IPAQ)** self-report. Total metabolic equivalent(met) minutes/week. Median(95%CI) | N=25 693 (60, 1386) | N=34 1409 (495, 2310) | N=25 2273 (1434, 3112) | N=34 2899 (2065, 3734) | 179 (-1542, 1900) |
| **EQ5D** health status based on UK population norms(1= full health) median (95%CI) | N=14 0.77 (0.69,1.0) | N=21 0.80 (0.73,.81) | N=14 0.83 (0.70,0.97) | N=21 0.80 (0.66,0.93) | 0 (-0.24, 0.24) |
| **EQ5D** self-rated health thermometer (100=best possible), median(95%CI) | N=21 70(60,75) | N=27 70(50, 80) | N=25 73(68, 78) | N=27 73(68, 78) | 2.5(-5.8, 10.9) |
| **Perceived stress score** (possible range: 0-40. 13 is considered average, high stress groups: 20+) | N=25 14.9 (11.8, 18.0) | N=34 18.2 (15.2, 21.2) | N=25 14.7 (13.1, 16.4) | N=34 17.2 (15.5, 18.8) | -2.7(-6.1,5.1) |

For peer review only



For peer review only - http://bmjopen.bmj.com/site/about/guidelines.xhtml

Table 4. Chronic Study: Autonomic function: heart rate variability, baroreceptor sensitivity and salivary amylase

| | Pre-intervention | | 3 months follow-up adjusted for Pre-intervention level | | Between group difference adjusted for pre-intervention levels (95% CI) |
|---|---|---|---|---|---|
| | Yoga + usual care | Usual care | Yoga + usual care | Usual care | Yoga minus usual care |
| **Heart rate variability and baroreceptor sensitivity 10 minute recording** Medians (95%CIM) | N=24 | N=33 | N=24 | N=33 | |
| Number of beats | 677(605, 877) | 709(632, 798) | 644 (589, 698) | 638 (584, 693) | -1(-110, 108) |
| Number of ectopics | 19 (8, 71) | 22 (8, 48) | 29 (6, 53) | 26 (2, 49) | -1(-46, 44) |
| Mean RR interval , millisecond(ms) | 1016(888, 1128) | 977(925, 1073) | 1050(1012, 1089) | 1020(982, 1059) | 13(-62, 87) |
| SDNN, ms | 55.5 (39.7, 78.6) | 53.4 (37.4, 69.2) | 48.2 (36.7, 59.6) | 47.2 (35.9, 58.6) | 3.6 (-20.4, 27.5) |
| RMSSD, ms | 38.5 (30.0, 60.0) | 38.8 (30.2, 57.8) | 44.4 (35.2, 53.5) | 39.9 (30.8, 48.9) | 4.4 (-13.8, 22.8) |
| NN50 | 40(18,57) | 49(24,67) | 49 (31, 66) | 51(34, 68) | 17(-20, 54) |
| pNN50 | 0.08 (0.03, 0.11)) | 0.08 (0.04, 0.14) | 0.14 (0.10, 0.17) | 0.11 (0.07,0.15) | 0.01 (-0.06, -0.08) |
| Triangular index | 179(149, 231) | 177(139, 235) | 189 (163, 216) | 187(160, 214) | 20(-37, 77) |
| Total RR interval power, $ms^2$ | 1514 (633, 2339) | 995 (680, 2097) | 1157 (653, 1662) | 1171 (668, 1673) | 401 (-591, 1393) |
| LF RR interval power, $ms^2$ | 340(119, 613) | 284 (177, 491) | 340(143, 537) | 340(143, 538) | 20(-391, 430) |
| HF RR interval power, $ms^2$ | 251(77, 759) | 235(126, 317) | 265(113, 417) | 265(113, 16) | 101(-256, 457) |
| LF/HF power ratio | 1.5(0.8, 2.1) | 1.4(1.1, 1.7) | 1.2(0.9, 1.4) | 1.2 (0.9, 1.4) | 0.04(-0.5, 0.6) |
| LF RR interval power, normalised units(nu) | 0.23 (0.16, 0.32) | 0.27 (0.25, 0.30) | 0.28 (0.24, 0.31) | 0.29 (0.26, 0.32) | 0.03 (-0.03, 0.09) |
| HF RR interval power, nu | 0.17 (0.12, 0.33) | 0.19 (0.14, 0.28) | 0.23 (0.17, 0.29 | 0.23 (0.17, 0.29) | -0.02 (-0.13, 0.10) |
| LF alpha index, ms/mm Hg | 6.9 (4.6, 12.9) | 9.1(6.6, 13.6) | 10.1 (7.5, 12.7) | 10.4(7.8, 13.0) | 0.8(-4.4, 6.1) |
| HF alpha index, ms/mm Hg | 9.2(4.6, 25.6) | 13.9(9.2, 22.6) | 15.8 (7.7, 23.9) | 16.4(8.3, 24.5) | 3.7(-9.4, 16.8) |
| Baroreceptor sensitivity on sequence analysis, ms/mmHg | 9.9 (6.3, 16.1) | 10.0(7.0, 13.0) | 10.6 (8.2, 13.1) | 10.6(8.2, 13.0) | 1.5(-2.6, 5.6) |
| | | | | | |
| **Salivary amylase, log microunits/L ,** (means(95%CI)) | N=23 | N=32 | N=23 | N=32 | |
| Waking | 4.19 (3.83, 4.41) | 4.48 (4.23, 4.73) | 4.39 (4.06, 4.71) | 4.45 (4.17, 4.74) | -0.05 (-0.68, 0.58) |
| Waking+30 minutes | 4.03 (3.74, 4.33) | 4.08 (3.83, 4.33) | 4.03 (3.71, 4.34) | 4.26 (3.98, 4.54) | -0.23 (-0.84, 0.39) |



For peer review only - http://bmjopen.bmj.com/site/about/guidelines.xhtml

| | Pre-intervention | | 3 months follow-up adjusted for Pre-intervention level | | Between group difference adjusted for pre-intervention levels (95% CI) |
|---|---|---|---|---|---|
| | Yoga + usual care | Usual care | Yoga + usual care | Usual care | Yoga minus usual care |
| Waking +1hrs 30 minutes | 4.45 (4.16, 4.74) | 4.88 (4.62, 5.13) | 4.48 (4.16, 4.79) | 4.47 (4.19, 4.76) | -0.01 (-0.63, .61) |
| Waking +12 hours | 4.61 (4.30, 4.92) | 4.74 (4.48, 4.99) | 4.29 (3.97, 4.61) | 4.70 (4.42, 4.98) | -0.42 (-1.0, 0.21) |
| Bedtime | 4.42 (4.12, 4.72) | 4.66 (4.41, 4.92) | 4.56 (4.23, 4.88) | 4.44 (4.16, 4.73) | 0.11 (-0.52, 0.73) |

Table abbreviations:
Mean RR interval: time in milliseconds (ms) between successive R waves on ECG
SDNN: standard deviation of N-N intervals, ms
RMSSD: root mean square of successive R-R interval differences, ms
NN50: number of successive N-N intervals that differ by more than 50 ms
pNN50: Proportion of successive RR intervals that differ by more than 50 ms
LF RR interval power: absolute power of the low-frequency band (0.04–0.15 Hz), $ms^2$
HF RR interval power: absolute power of the high-frequency band (0.15–0.4 Hz, $ms^2$
LF/HF power ratio: Ratio of LF-to-HF power
LF RR interval power: Relative power of the low-frequency band (0.04–0.15 Hz) , normalised units(nu)
HF RR interval power: Relative power of the high-frequency band (0.15–0.4 Hz), nu
LF alpha index: Low frequency alpha index, ms/mm Hg
HF alpha index: High frequency alpha index, ms/mm Hg

For peer review only



For peer review only - http://bmjopen.bmj.com/site/about/guidelines.xhtml

Table 5a: Acute study, **Primary outcomes**: immediate post-exercise results following a 3-minute step-test, before and after (same day) the first yoga class

| **N=27** | **Before first yoga session** Mean (95%CI) | **After first yoga session:** Mean (95% CI) | **Post –pre first yoga session difference** Mean (95% CI) | **P value for comparison between before and after first yoga session** |
|---|---|---|---|---|
| **Resting seated** | | | | |
| Systolic blood pressure, mm Hg | 132(128, 136) | 133(129, 136) | 1(-9, 11) | 0.9 |
| Diastolic blood pressure, mm Hg | 78(77, 81) | 79(77, 81) | 1(-5, 7) | 0.7 |
| Heart rate, bpm | 69(67, 71) | 68(66, 70) | -1(-8, 6) | 0.9 |
| **Immediately post-step-test, standing** | | | | |
| Systolic blood pressure, mm Hg | 161(157, 164) | 156(148, 163) | -5(-15, 6) | 0.4 |
| Diastolic blood pressure, mm Hg | 80(78, 82) | 80(78, 82) | -1(-7, 5) | 0.8 |
| Heart rate, bpm | 92(90, 94) | 88(86, 90) | -3(-10, 4) | 0.4 |
| Peak $VO_2$, ml/min/kg (n=24) | 36.9(33.4, 40.4) | 38.9(35.4, 42.4) | 2.0(-0.2, 4.1) | 0.07 |
| **3 minutes post-step-test, seated** | | | | |
| Systolic blood pressure, mm Hg | 133(129, 136) | 135(131, 139) | 2(-8, 13) | 0.7 |
| Diastolic blood pressure, mm Hg | 78(76, 80) | 79(77, 82) | 1(-5, 8) | 0.6 |
| Heart rate, bpm | 72(70, 74) | 69(67, 71) | -3(-10, 4) | 0.4 |

*12 participants did not participate in the step-test both before and after the first yoga session due to: mobility problems+/- shortness of breath (n=3), refused (n=3), frailty (n=2), unstable angina (n=1), other (high blood pressure/dizziness/weakness on left side/restricted movement; n=3). Blood pressure readings were unavailable for 1 participant due to equipment failure.

Table 5b: Acute study: Secondary outcomes: salivary amylase and cortisol: pre-intervention and day of first yoga class

| | **Pre-intervention** Means(95%CI) | **Day of first yoga session** Means(95%CI) | **Post –pre first yoga session difference Means(95% CI)** |
|---|---|---|---|
| | N=32* | N=32* | |
| **Cortisol, nmol/l** | | | |
| Waking | 12.7(11.0, 14.4) | 15.4(12.8, 17.9)(pre yoga) | N/A |
| 12 hours after waking | 3.6(1.8, 5.4) | 4.4(1.7, 7.0) (post yoga) | 0.5(-3.5, 4.5) |
| Bedtime | 3.4(1.6, 5.2) | 3.0(0.3, 5.7) (post yoga) | -0.3(-4.3, 3.7) |
| **Amylase, log microunits/L** | | | |
| Waking | 4.40 (4.23, 4.57) | 4.28(4.02, 4.54) (pre yoga) | N/A |
| 12 hours after waking | 4.66(4.47, 4.84) | 4.58(4.32, 4.85) (post yoga) | -0.08(-0.54, 0.38) |
| Bedtime | 4.60(4.42, 4.78) | 4.74(4.47, 5.01) (post yoga) | 0.13(-0.32, 0.59) |

*8 participants were unable to provide adequate saliva samples pre-recruitment and on the day of the first yoga session. N/A, not available.



For peer review only - http://bmjopen.bmj.com/site/about/guidelines.xhtml

SUPPLEMENTAL Table S1a. Characteristics at start of study (pre intervention) of those who completed/did not complete 3month follow-up: Usual care group

| Means95%CI), number(%) unless otherwise stated | Usual Care, completed | Usual Care, dropped out |
|---|---|---|
| | N=35 | N=5 |
| Ethnicity: South Asian | 22(63%) | 4(80%) |
| Sex: Male | 22(63%) | 5(100%) |
| Age: years | 57.2(54.0, 59.8) | 54.5(38.3,73.6) |
| Days since coronary event | 58(52, 65) | 61(41,84) |
| Previous heart attack | 5/33(15%) | 0/5 |
| Diabetes, self report of physician diagnosis | 13(37%) | 1(20%) |
| Heart failure, self report of physician diagnosis | 6/31(19%) | 1(20%) |
| Hypertension, self report of physician diagnosis | 23/33 (70%) | 1/4(25%) |
| Blood pressure lowering medication* | 35(100%) | 5(100%) |
| Number of blood pressure lowering medications*, median(IQR) | 3(2,3) | 3(2,3) |
| Beta blocker use* | 27(77%) | 5(100%) |
| Statin use * | 31(89%) | 5(100%) |
| Current smoker/ex smoker/never smoker, number | N=31<br>1/13/17 | N=5<br>0/2/3 |
| Alcohol, never/ever drinkers, number<br>Units/week (ever drinkers), median(IQR) | N=30<br>7/23<br>2(1,6) | <br>3/2<br>7(7,7) |
| Reason given for dropout | | Refused follow-up (4)<br>Unwell (1) |

*self-reported either pre-intervention or at follow-up

For peer review only

Table S1b. Characteristics at start of study (pre intervention) of those who completed/did not complete 3 month follow-up. Yoga+usual care group

| Means(95%CI), number(%) unless otherwise stated | Yoga+usual care Completed 3 month follow-up +/- 18 sessions | Yoga+usual care Attended 3 month follow-up and at least 18 yoga sessions | Yoga+usual care Dropped out of yoga and follow-up | Yoga+usual care Attended 3 month follow-up but attended <18 yoga sessions |
|---|---|---|---|---|
| | N=29 (4 did not complete 18 sessions) | N=25 | N=11 | N=4 |
| Ethnicity: South Asian | 17(59%) | 15(60%) | 8(73%) | 2(50%) |
| Sex: Male | 21(72%) | 18(72%) | 7(64%) | 3(75%) |
| Age: years | 58.5(54.5, 62.5) | 57.9(53.6, 62.2) | 54.5(47.9, 61.2) | 62.2(42.8, 81.7) |
| Days since coronary event | 51(42,59) | 50±24 | 49±22 | 54(32,75) |
| Previous heart attack | 4/28 (14%) | 4/24(17%) | 3/7(30%) | 0/4 |
| Diabetes, self report of physician diagnosis | 12(41%) | 12(41%) | 3 (27%) | 3 (75%) |
| Heart failure, self report of physician diagnosis | 4/27(15%) | 2/23(9%) | 3/9(33%) | 2(50%) |
| Hypertension, self report of physician diagnosis | 23/28(82%) | 19/24(79%) | 5/8(63%) | 4(100%) |
| Blood pressure lowering medication* | 29(100%) | 25(100%) | 10/10(100%) | 4(100%) |
| Number of blood pressure lowering medications*, median(IQR) | 3(3,3) | 3(3, 3) | 3(2,3) | 3(2,4) |
| Beta blocker use* | 25(86%) | 22(88%) | 8(73%) | 3(75%) |
| Statin use * | 28(97%) | 24(96%) | 8(73%) | 4(100%) |
| Current smoker/ex smoker, number | N=27<br>2/10 | N=23<br>2/8 | 2/4 | 0/2 |
| Alcohol, never/ever drinkers, number<br>Units/week (ever drinkers), median(IQR) | N=26<br>8/18<br>2(0,5) | N=23<br>8/15<br>2(0,4) | N=10<br>5/5<br>11(1,12) | 0/4<br>9(1, 20) |
| Reason given for dropout | | | Refused follow-up (4)<br>Unwell(6)<br>Returned to work, unable to attend further classes/follow-up (1) | Refused further yoga(4) |

*self-reported either pre-intervention or at follow-up



For peer review only - http://bmjopen.bmj.com/site/about/guidelines.xhtml

For peer review only



For peer review only - http://bmjopen.bmj.com/site/about/guidelines.xhtml

# Figure S1

## YACHT Study Recruitment

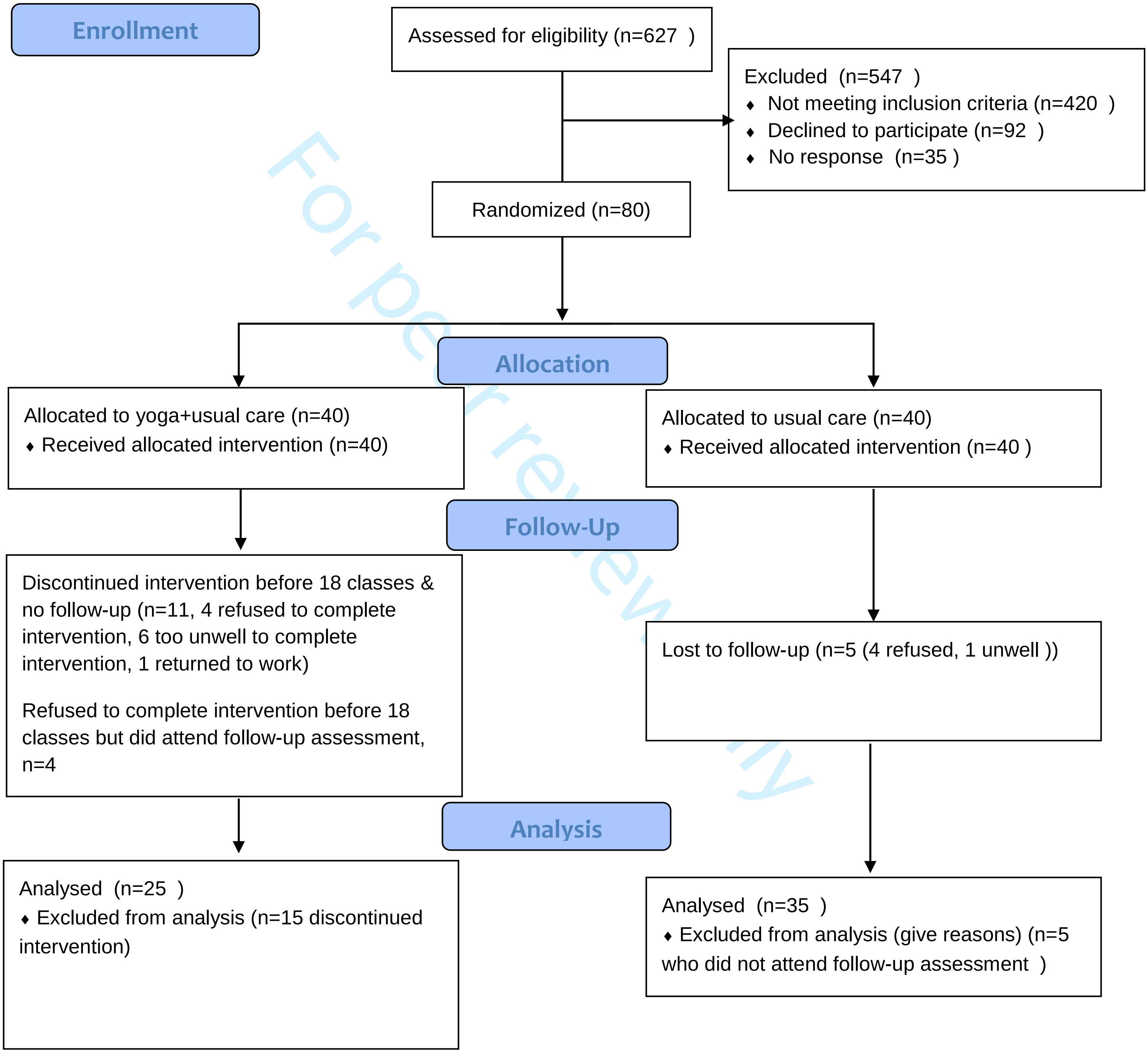


For peer review only

# YACHT

**Y**oga **A**nd **C**ardiovascular **H**ealth **T**rial

# Manual of Operations

Version 1.2

July 2012

**Compiled by Barbara Sowa and Claire Tuson**

review only

Imperial College London

# Table of Contents

**STUDY SUMMARY** ..... 3
**OUTLINE STUDY DESIGN** ..... 4
**YOGA SUMMARY** ..... 5
**YOGA FULL DESCRIPTION** ..... 5
PART 1: INITIAL RELAXATION AND WARM-UP – 10 MINUTES ..... 5
*Initial relaxation* ..... 6
*Warm-up* ..... 7
PART II: ASANAS - 20 MINUTES ..... 8
*Standing poses (High positions)* ..... 8
*Kneeling & Sitting poses (Medium positions)* ..... 10
*Lying down poses (Low positions)* ..... 13
PART III: COOL DOWN: BREATHING EXERCISES AND FINAL RELAXATION - 20 MINUTES ..... 14
*Deep Abdominal Breathing* ..... 14
*Full Yogic Breath* ..... 15
*Final Relaxation* ..... 15
*Alternative relaxation* ..... 16
PART IV: SUPERVISION OF PARTICIPANTS POST-EXERCISE AND EDUCATION - 20 MINUTES ..... 18
*Proper Exercise* ..... 18
*Proper Breathing* ..... 18
*Proper Relaxation-Savasana* ..... 19
*Proper Diet-Vegetarian – Part I* ..... 19
*Proper Diet-Vegetarian – Part II* ..... 20
*Positive Thinking and Meditation – Part I* ..... 20
*Positive Thinking and Meditation – Part II* ..... 21
*Making Positive Changes in Your Life* ..... 22
**APPENDIX 1** ..... 23
POLICY FOR CARDIAC REHABILITATION IN EALING ..... 23
**APPENDIX 2** ..... 32
EALING CARDIAC REHABILITATION HEALTH EDUCATION TALKS ..... 32
*Drugs for heart disease and how they work* ..... 32
*Managing Stress* ..... 34
*Eating for a healthy heart* ..... 37
*Exercise and the benefits for your heart* ..... 38
*Risk factors and making lifestyle changes* ..... 41
*The heart and how it works* ..... 44
**REFERENCES** ..... 48

DRAFT
For peer review only

Imperial College London

## Study Summary

| | |
|---|---|
| **TITLE** | Yoga And Cardiovascular Health Trial (YACHT) |
| **DESIGN** | Epidemiological |
| **AIMS** | To perform a mechanistic study to determine the acute and chronic effects of yoga on neuro-endocrine pathways, and downstream effects on CVD risk factors and subclinical outcomes. This will provide complimentary information to a larger clinical trial in India designed to determine effects of yoga on cardiovascular morbidity and mortality in acute coronary syndromes. |
| **POPULATION** | Indian Asians and Europeans, 40 in each ethnic group, (self-defined, verified by country of birth of all 4 grandparents). Aged between 35 to 80 years, male or female, without co-morbid disease and mobility limitations that would preclude participation in cardiac rehabilitation and our investigations. |
| **ELIGIBILITY** | Referred to cardiac rehabilitation programmes in West London post-angioplasty as treatment for an acute coronary syndrome. Able to understand English or Punjabi, Hindi or Gujarati, but in order to be able to follow the yoga class instructions the participant will need to have a basic command of the English language. |
| **DURATION** | Recruitment is planned for 1 year. |

DRAFT
For peer review only

Imperial College London

# Outline Study Design

Indian Asians and Europeans, 40 in each ethnic group, (self-defined, verified by country of birth of all 4 grandparents), referred to cardiac rehabilitation programmes in West London post-angioplasty as treatment for an acute coronary syndrome, will be invited to participate when being discharged from hospital.

Those who agree will be randomized to the yoga intervention plus their standard cardiac rehabilitation programme (usual care), or usual care alone.

In order to evaluate the chronic effects of yoga, baseline and 3 month measurements will be performed on all participants as described below:

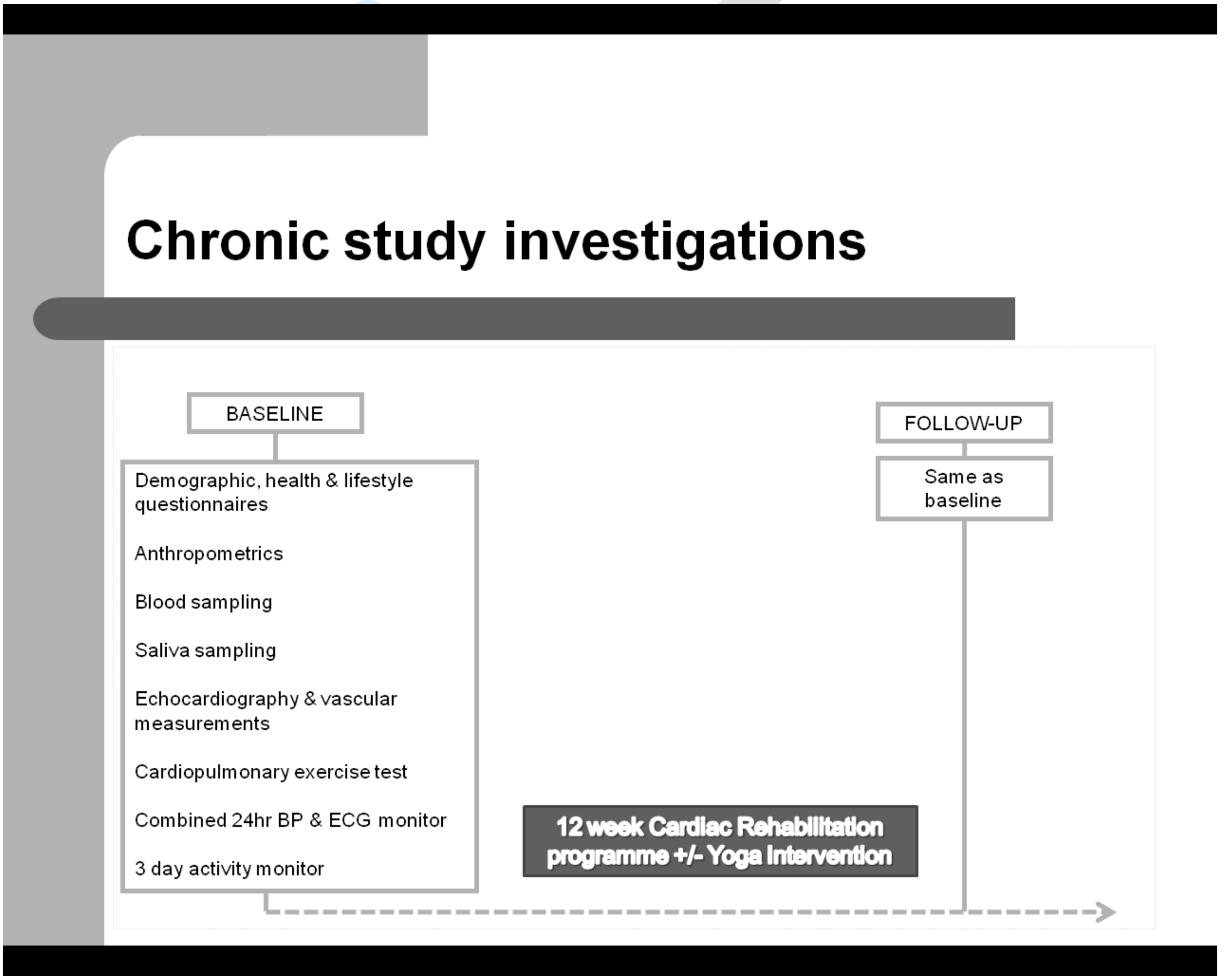

Imperial College London

## Yoga Summary

The yoga intervention will be delivered on a bi-weekly group session basis for 12 weeks alongside the cardiac rehabilitation programme. There will be 24 yoga classes in total, of which, each participant will be required to attend a minimum of 18. The yoga session will be designed and conducted by a teacher certified in yoga and cardiac rehabilitation, and will encompass physical fitness (yoga poses), stress reduction (breath control and meditation) and positive lifestyle changes (diet, smoking and alcohol).

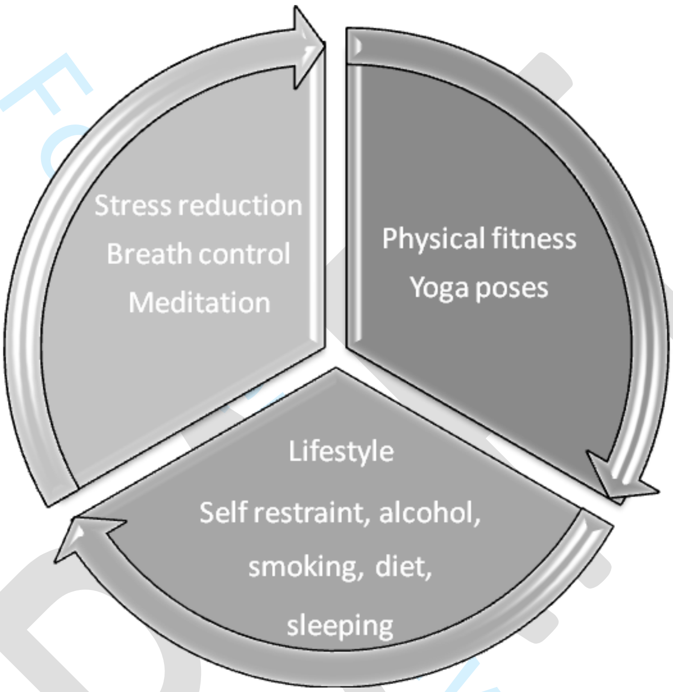


Each class will be approximately 1 hour and 15 minutes and in order to address the balance shown in the above diagram it will consist of the following parts:

- Yogic poses – approx 25 mins
- Breathing exercises and meditation – approx 25 mins
- Education and discussion – approx 25 mins

## Yoga Full Description

### *Part 1: Initial relaxation and warm-up – 10 minutes*

**Rationale:**


For peer review only - http://bmjopen.bmj.com/site/about/guidelines.xhtml

Imperial College London

The initial relaxation and warm-up should be 10 - 15 minutes in duration. Gradual increase in intensity triggers three mechanisms which increase coronary blood flow to match the increased myocardial demand. As a result the ischaemic threshold is extended and the risk of angina and the risk of arrhythmias is reduced.
Due to older average age of this group (compared with mainstream) a gradual progression of range of motion exercises is prescribed.
The short preparatory stretches are included to prepare the muscles for the range of movement involved in Asanas to reduce risk of injury and encourage good balance and alignments. Since incorporating a static stretching will result in fall in heart rate, stretches will be intersperse with some dynamic movement (walking on the spot) designed to maintain the elevated heart rate.

## Initial relaxation

1. Emphasise mood, breathing, and relaxation

2. Set the mood for the class. Explain what yoga is, how can help, how should be practiced. Concentration, breathing and relaxation in all yoga practice should be explained and repeated in each class. Put participants in a relaxed state emphasising body position, breathing and relaxation. Use voice to relax them.

3. Begin by finding a comfortable position standing position with your feet hip or shoulder-width apart. You can change positions any time during the relaxation exercises to make yourself more comfortable as needed. Start from breathing. Breathe in slowly and deeply through your nose. Continue to breathe slowly and gently. Allow your breathing to relax you.

The next relaxation exercise focuses on relaxing the muscles of your body.

1. Start with the large muscles of your legs. Tighten all the muscles of your legs. Hold it for a few moments and now relax. Let all the tension go.

2. Now focus on the muscles in your arms. Tighten your shoulders, upper arms, lower arms, and hands. Squeeze your hands into tight fists. Tense the muscles in your arms and hands as tightly as you can. Hold it for a few moments and release. Allow the muscles in your arms to relax completely.

3. Focus again on your breathing. Slow, even, regular breaths. Continue to breathe slowly and rhythmically.

4. Now focus on the muscles of your buttocks. Tighten these muscles as much as you can. Hold this tension and release. Relax your muscles.

5. Tighten the muscles of your back now. Feel your back tightening, pulling your shoulders back and tensing the muscles along your spine. Arch your back slightly as you tighten these muscles. Hold and relax. Let all the tension go. Feel your back comfortably relaxing into a good and healthy posture.

6. Turn your attention now to the muscles of your chest and stomach. Tighten and tense these muscles and release. Relax the muscles of your trunk.

7. Finally, tighten the muscles of your face. Scrunch your eyes shut tightly, wrinkle your nose, and tighten your cheeks and chin. Hold this tension in your face and relax. Release all the tension. Feel how relaxed your face is.

Notice all of the muscles in your body, notice how relaxed your muscles fee l. Allow any last bits of tension to drain away. Enjoy the relaxation you are experiencing. Notice your calm breathing and your relaxed muscles. Enjoy the relaxation for a few moments.


For peer review only - http://bmjopen.bmj.com/site/about/guidelines.xhtml

Imperial College London

When you are ready to return to your usual level of alertness and awareness, slowly begin to re-awaken your body. Wiggle your toes and fingers. Swing your arms gently. Shrug your shoulders.

## Warm-up

1. Now take a few steps on the spot and stand with your feet shoulder-width apart (set position), inhale and bend your knees and do mini-squat, exhale and come back to your set position. Repeat 4 times.

2. Take another few steps on the spot and come back to your set position. Inhale, roll your shoulders up, exhale bring them back and down. Repeat 4 times. During the last repetition hold your shoulders up for 4 to 8 seconds.

3. Take another few steps on the spot and come back to your set position. Inhale, roll your shoulders up, exhale bring them forward and down. Repeat 4 times. During the last repetition hold your shoulders up for 4 to 8 seconds.

4. Take another few steps on the spot and come back to your set position. Inhale, raise your arms to the side (shoulder height), exhale bring them down. Repeat 4 times. During the last repetition hold your arms up for 4 to 8 seconds.

5. Take another few steps on the spot and come back to your set position. Inhale, raise your arms to the front (shoulder height), exhale bring them down. Repeat 2 times. As above add heel raises at the same time if comfortable. Hold the last repetition for 4 to 8 seconds.

6. Take another few steps on the spot and come back to your set position. Inhale, raise your arms to the side (shoulder height), exhale and twist your trunk to the right. Inhale and come back to the centre, exhale and twist your trunk to the left. Repeat 2 times on each side. Hold the last repetition for 4 to 8 seconds.

7. Relax your arms and take another few steps on the spot and come back to set position. Inhale, raise your arms to the side (shoulder height) and look up. Exhale, bring your right arm crossover the chest to the left arm. Inhale, bring your right arm to the side. Exhale, bring your left arm crossover the chest to the right arm. Inhale, bring your left arm to the side. Repeat 2 times on each side. Hold the last repetition for 4 to 8 seconds.

8. Take another few steps on the spot and come back to your set position. Inhale and laterally bend your trunk to the right, exhale and come back to the centre.  . Inhale and laterally bend your trunk to the left, exhale and come back to the centre.  Repeat 2 times on each side. Hold the last repetition for 4 to 8 seconds.

DRAFT

For peer review only



For peer review only - http://bmjopen.bmj.com/site/about/guidelines.xhtml

Imperial College London

*Part II: Asanas - 20 minutes*

**Rationale:**
Gentle progression from high to low positions is prescribed to avoid rapid changes of body positions in order to decrease the risk of arrhythmias or orthostatic hypotension in some individuals. Various 'chest opener' Asanas are prescribed to correct the round shouldered posture adopted by the population in response to discomfort in the area of the sterna incision.

## Standing poses (High positions)

**Mountain pose –Tad asana**

1. Come to stand with the big toes touching.

2. Lift up all your toes and let them fan out, then drop them down creating a wide solid base. You can separate your heels slightly if your ankles are knocking together uncomfortably.

3. Bring your weight evenly onto all four corners of both feet.

4. Let the feet and the calves root down into the floor.

5. Engage the quadriceps and draw them upward, causing your knee caps to rise.

6. Rotate both thighs inward, creating a widening of the sit bones, and tuck your tailbone in between the sit bones.

7. Tone the belly, drawing it in slightly.

8. Widen the collar bones and make sure the shoulders are parallel to the pelvis.

9. The neck is long, the crown of the head rises toward the ceiling, and the shoulder blades slide down the back.

10. Hold for 5-10 breaths.

**Raised Hands Pose - Urdhva Hastasana**

1. From Tadasana, bring your arms out to the side and up.

2. Press the palms together, keep the arms straight and take the gaze up toward your thumbs.

3. Slide the shoulder blades down the back.

4. Maintain your alignment.

5. Hold for 5-10 breaths.

**Awkward Chair Pose - Utkatasana**

1. From  Tadasana bend the knees until the thighs are almost parallel to the floor.

2. Keep the butt low.


For peer review only - http://bmjopen.bmj.com/site/about/guidelines.xhtml

3. Bring the arms up towards the ceiling.

4. Bring a slight back bend into the upper back.

5. Hold for 5-10 breaths.

**Beginners**: Work on bring the thighs closer and closer to parallel to the floor.

**Advanced**: Try this variation: Bring the hands into a prayer position at the heart. Twist to the right side, bringing the left elbow outside the right knee. Stay low in the pose and keep the knees pressing together. Come back to centre and then do the left side.

## Triangle Pose – Trikonasana

1. From Tadasana take a big step backwards with your left leg.

2. Pivot on the ball of the left foot and drop the left heel onto the floor with the toes turned out about 45 degrees from the heel.

3. Bring the arms out to the side.

4. Slide the shoulder blades down the back.

5. Begin to reach the right arm forward, drawing the right thigh upwards and tucking the hip as you come forward.

6. Drop the right hand down to your shin or ankle, or if you are able, onto the floor inside or outside the right foot. Do whichever one feels most comfortable,

7. The left shoulder stacks on top of the right one as you open the chest reaching the left fingertips upwards while keeping the left shoulder rooted in the socket.

8. Take your gaze up towards the left fingertips.

9. Draw the right thigh muscle upwards, deepening the right hip crease.

10. Slightly bend the right knee.

12. Hold for 5-10 breaths

13. Repeat on the left side.

**Beginners**: Bring the right hand higher up on your leg or use a block on the floor to rest your hand on. It is more important to keep the right leg straight than to bring the right hand to the floor. Do not rest the hand directly on the knee, though, as this creates too much pressure on the knee.

**Advanced**: Line up the right heel with the arch of the left foot. For a variation, try dropping the left arm over the left ear so it comes parallel to the floor, while keeping the shoulder rooting into the socket.

## Tree Pose - Vrksasana

1. Come to stand in Tadasana.

2. Feel your weight equally on all four corners of both feet.



For peer review only - http://bmjopen.bmj.com/site/about/guidelines.xhtml

Imperial College London

3. Begin to shift the weight over to the right foot, lifting the left foot off the floor.

4. Bend the left knee, bringing the sole of the left foot high onto the inner right thigh.

5. Press the foot into the thigh and the thigh back into the foot.

6. Try not to let the right hip jut out. Keep both hips squared towards the front.

7. Focus on something that doesn't move to help you keep your balance.

8. Hold for 5-10 breaths.

9. Repeat the move while standing on the left foot.

**Beginners**: If you cannot bring the left foot high inside the right thigh, bring it lower on the right leg - but be careful to avoid placing the left foot directly on the right knee.

Use the wall for balance if necessary.

**Advanced**: Bring the arms up towards the ceiling with the palms touching. Open the arms out to side.

Try closing the eyes and see if you can stay balanced.

## Kneeling & Sitting poses (Medium positions)

### <u>**Cat - Cow Stretch - Chakravakasana**</u>

1. Start on all fours, bringing the wrists underneath the shoulders and the knees underneath the hips.

2. Think of the spine as a straight line connecting the shoulders to the hips. Try visualizing the line extending forward through the crown of the head and backwards through the tail bone.

3. Keep the neck the natural extension of the spine.

On an inhale:

1. Curl the toes under.

2. Drop the belly.

3. Take the gaze up toward the ceiling.

4. Let the movement in the spine start from the tailbone, so that that neck is the last part to move.

On the next exhale:

1. Release the tops of the feet to the floor.

2. Round the spine.

3. Drop the head.


For peer review only - http://bmjopen.bmj.com/site/about/guidelines.xhtml
DRAFT
For peer review only

Imperial College London

4. Take the gaze to the navel.

5. Repeat the Cat - Cow Stretch on each inhale and exhale, matching the movement to your own breath.

6. Continue for 5-10 breaths, moving the whole spine. After your final exhale, come back to a neutral spine.

## Hands and Knees Balance

1. Come on to all fours with the wrists underneath the shoulders and the knees underneath the hips.

2. Extend the right leg to the back of your mat and flex the foot.

3. Lift the right leg up to hip-level, keeping the hips squared towards the floor and the foot flexed.

4. Lift the left arm up to shoulder level.

5. Balance on the left knee and right hand, keeping the spine neutral and the neck long.

5. Stay 5-10 breaths before lowering the lifted hand and knee and doing the other side.

**Beginners**: Take care not to let the spine collapse while you are balancing.

**Advanced**: Bend the knee of the lifted leg. Reach around with the lifted arm and hold on to the inside of the lifted foot.

## Staff Pose - Dandasana

1. Sit with the legs outstretched straight in front.

2. Engage the thigh muscles and flex the feet. The heels may come up off the floor.

3. Make your spine long.

4. Stack the shoulders directly on top of the hips.

5. Hold for 5-10 breaths.

**Beginners**: Put padding under your sit bones, if necessary.

**Advanced**: This pose looks easy, but if you are really working the thighs, you can break a sweat.

## Seated Forward Bend - Paschimottanasana

1. From Dandasana bring the arms straight out to the sides and up over your head.

2. Inhale and draw the spine up long.

3. As you exhale, begin to come forward, hinging at the hips.

4. On each inhale, extend the spine, and on each exhale, come a bit farther into the forward bend.

5. Keep the neck at the natural extension of the spine.

6. Do not round the back.



For peer review only - http://bmjopen.bmj.com/site/about/guidelines.xhtml

Imperial College London

7. Take hold of the ankles or shins, whichever you can reach.

8. Hold for 5-10 breaths.

**Beginners**: Put padding under the sit bones if necessary. Imagine the belly coming to rest on your thighs, rather than the nose coming to the knees - this will help you keep the spine long instead of curving over.

**Advanced**: If you can easily grab the soles of your feet, try taking a block in front of the feet and holding that instead.

## Crab pose- Catuspadapitham

**1.** From Dandasana, bend the knees bringing the feet flat on the floor hip width apart. Keep the arms behind your hips with the fingers pointed away from your body.

**2.** Lean back into the arms and slowly inhale and lift the hips up towards the ceiling. Make sure the toes and knees are pointing straight ahead. Look straight ahead, up at the ceiling or carefully drop the head back.

**3.** Press into the feet, squeezing the thighs and buttocks and engaging Mula Bandha to lift the hips high. Press into the hands and draw the shoulder blades towards each other to lift up high through the sternum.

**4.** Breathe and hold for 2-6 breaths.

**5.** To release: slowly exhale the hips back down to the floor.

**Beginners**: If there is pain or discomfort in the wrists, point the fingers in the opposite direction or make fists with the hands.

**Advanced**: Inhale one leg up towards the ceiling at a time, pressing out through the heel.

## Half Lord of the Fishes Pose (Half Spinal Twist) - Ardha Matsyendrasana

1. From Dandasana, bend your left knee and bring the sole of your left foot to the floor on the outside of the right thigh.

2. Bend the right knee, and tuck the right foot in near the left buttock.

3. Inhale and bring the right arm up near your right ear.

4. Exhale and twist the to the left, bringing the right elbow to the outside the of left knee and the left palm to the floor, just behind your sit bones.

5. Look out over the left shoulder, but don't overturn the neck -- the twist originates in the belly, not the neck.

6. On each inhale, draw the spine long, and on each exhale, twist a little deeper.

7. Be sure to keep the sole of your left foot flat on the floor.

8. Hold for 5-10 breaths.

9. When you release the pose, take a slight counter twist to the opposite direction.

10. Release the legs and switch their position as you prepare to twist to the other side.


For peer review only - http://bmjopen.bmj.com/site/about/guidelines.xhtml

**Beginners**: You may want to sit on some padding if you are uncomfortable. If you cannot bend it into the ideal position, you may also keep the right leg extended.

**Advanced**: Come into a bind with the arms. Thread the right arm back underneath the left knee. Reach the left arm behind your back, and clasp the left wrist with your right hand.

### Easy Pose - Sukhasana

1. Arrange padding under your sit bones so that your hips come above your knees.

2. Come to sit in a comfortable, cross-legged position.

3. Bring one heel in towards your groin. The other foot may rest on the floor in front of you or you may bring it into your lap.

4. Root your seat down as your spine grows long. Stack the shoulders over the hips and slide the shoulder blades down your back. The crown of your head rises towards the ceiling.

### Neck Exercises

1. In Sukhasana, with your back straight and your chest erect. Slowly bring your head forwards towards the chest to give the back of your neck a good stretch.

2. After a few breaths slowly lift your head and extend your neck back and bring your head to neutral position.

3. Lower your right ear close to your right shoulder, then repeat on the other side. Keep both shoulders level throughout. Repeat the exercise 5 times.

4. Turn your head to the right side. Contract the muscles on the right side of your neck and feel the stretch on the left side. Repeat on the opposite side. Repeat the exercise 5 times.

## Lying down poses (Low positions)

### Single Leg Lift

1. Lie flat on your back with your legs together, arm next to your body, and palms face down.

2. Inhale and raise your left leg, keeping your knee straight, toes towards your head.

3. Exhale and lower your leg to the starting position.

4. Repeat up to 5 times on the each side.

### Head to Knee Raise

1. Start from Single Leg Left Step 2.

2. With an exhalation, bend your left leg and clasp your hands around your knee.

3. With an inhalation, lift your head and try to bring your forehead against your left knee.

4. With an exhalation, lower your head, arms, and leg.

5. Repeat on the opposite side.

DRAFT
For peer review only

Imperial College London

**Beginners**: Keep your head on the floor.

**Advanced**: Progress to Deep Stretch Single Leg Lift.

1. Start from Single Leg Lift Step 2.

2. With an exhalation, take hold of your leg with both hands, lift your back off the mat and try to bring your chest and head close to the raised leg.

## Happy Baby Pose - Ananda Balasana

1. Come to lie on the back.

2. Bend the knees into the chest.

3. Open the knees, bringing them towards the armpits.

4. Stack each ankle directly over the knee, so that the shins are perpendicular to the floor.

5. Flex the feet.

6. Hold the outer edges of the feet at you draw the knees towards the floor.

This pose is appropriate for both beginners and advanced students.

## Corpse Pose - Savasana

1. Come to lie down on the back with your arms and feet apart and your eyes closed.

2. Let the feet fall out to either side.

3. Turn the palms to face upwards.

4. Relax the whole body, including the face. Let the body feel heavy.

## *Part III: Cool Down: Breathing exercises and final relaxation - 20 minutes*

**Rationale:**
Twenty minutes Cool Down/Breathing/Relaxation period is prescribed to reduce risk of hypotension or arrhythmias and to allow the hart rate to return to pre-exercise rates.

**Deep Abdominal Breathing and Full Yogic Breath Practice-5 minutes**

### Deep Abdominal Breathing

1. In Corpse Pose place both hands on your abdomen with your fingers apart.

2. As you inhale, feel your abdomen and hands rising.

3. As you exhale, feel your abdomen and hands sinking.

4. Try to breathe rhythmically, with an inhalation lasting 3-5 seconds and exhalation of the same length.

DRAFT
For peer review only

Imperial College London

## Full Yogic Breath

1. In Corpse Pose place one hand on your chest and the other on your abdomen.

2. As you inhale, gradually expand the abdomen, then rise and open the rib cage, and finally lift the collar bones.

3. Begin the exhalation by relaxing the abdomen, then lower the rib cage, and finally slightly contract the abdomen to actively empty the lungs.

## Alternate Nostril Breathing (Anuloma Viloma)- 5 minutes

### Single Nostril Breathing

In Easy Pose, place your right hand in front of your face in Vishnu Mudra*. Close your right nostril with your thumb. Inhale for three seconds and exhale for six seconds through your left nostril. This is one round. Practice ten rounds. Repeat on the other nostril: close your left nostril with your ring finger, and inhale and exhale through your right nostril.

**Beginners**: Gradually increase the ratio of the inhalation to the exhalation lengthening to 4:8, 5:10 and 6:12.
**Advanced:** Progress to Simple Alternate Nostril Breathing

### Simple Alternate Nostril Breathing

In Easy Pose close your right nostril with your thumb, inhale through the left nostril for three seconds, close your left nostril with your ring finger, open your right nostril and exhale through it for six seconds. Inhale through your right nostril for three seconds, then exhale through your left nostril for six seconds. Practice for ten rounds. Gradually increase the inhalation: exhalation ratio to 4:8, 5:10 and 6:12.

*Vishnu Mudra: Hold your right hand with the palm facing you and fold the first and second fingers into the palm. Try to keep your thumb and ring fingers straight.

## Final Relaxation

1. Inhale and lift your right leg a few inches off the mat. Tense your leg, then exhale and allow your leg to drop. Repeat with the left leg.

2. Inhale and lift both arms a few inches off the mat. Clench your fists, tense your arms, then exhale and allow your arms to drop to the mat.

3. Inhale and lift your hips and buttocks off the mat. Tense your buttocks and then exhale and release.

4. Inhale and lift your chest off the mat. Tense your shoulder blades, then exhale and release.

5. Inhale and pull your shoulders towards your ears. Exhale and release your shoulders.

6. Inhale and squeeze the muscles of your face tightly together. Exhale and release.

7. Inhale, open your mouth, stick your tongue out and look to your forehead. Exhale and release.

8. With an inhalation, slowly roll your head to one side; with an exhalation, roll it to the other side. End by bringing your head back to centre.

DRAFT For peer review only


For peer review only - http://bmjopen.bmj.com/site/about/guidelines.xhtml

Imperial College London

Take a few slow rhythmic breaths using your abdomen, then follow this exercise in auto suggestion.

I'm relaxing my feet.....My feet are relaxed.....

I'm relaxing my ankles.....My ankles are relaxed.....

I'm relaxing my calves.....My calves are relaxed.....

I'm relaxing my knees.....My knees are relaxed.....

I'm relaxing my thighs.....My thighs are relaxed.....

I'm relaxing my hips and buttocks.....My hips and buttocks are relaxed.....

I'm relaxing my abdomen and chest.....My abdomen and chest are relaxed.....

I'm relaxing my lower and middle back.....My lower and middle back are relaxed.....

I'm relaxing my shoulders and neck.....My shoulders and neck are relaxed.....

I'm relaxing my hands and fingers.....My hands and fingers are relaxed.....

I'm relaxing my arms.....My arms are relaxed.....

I'm relaxing my mouth and eyes.....My mouth and eyes are relaxed.....

I'm relaxing my facial muscles and scalp.....My facial muscles and scalp are relaxed.....

I'm relaxing my internal organs: my kidneys, my livers, my intestines, my bladder, my pancreas, my stomach, my heart, my lungs and my brain.....My internal organs: my kidneys, my livers, my intestines, my bladder, my pancreas, my stomach, my heart, my lungs and my brain are relaxed.....

Continue abdominal breathing and relaxation. Visualise a calm lake, unruffled by waves. Picture the still water resting on your inner self, which is timeless and unchanging. Continue for a few more minutes.

Then take a few deep breaths, slowly move your legs and arms, and give your whole body a good stretch. Finally bring yourself slowly to sitting cross-legged position. Deeply inhale and exhale. Inhale and bring your hands into a prayer position and as you exhale bow your head thanking everyone for the practice.

## Alternative relaxation

In Savasana pose. For the next few moments, focus on calming your mind by focusing on your breathing. Allow you breathing to centre and relax you. Breathe in.... and out.

In..... out.....

In.... Out.....

Continue to breathe slowly and peacefully as you allow the tension to start to leave your body.

Release the areas of tension, feeling your muscles relax and become more comfortable with each breath.

Continue to let your breathing relax you....

DRAFT
For peer review only


For peer review only - http://bmjopen.bmj.com/site/about/guidelines.xhtml

Breathe in....2...3...4.... hold....2.....3...... out...2...3....4...... 5

again....2.....3....4....hold....2....3.... out...2...3....4.... 5

Continue to breathe slowly, gently, comfortably.....

Let the rate of your breathing become gradually slower as your body relaxes.

Now begin to create a picture in your mind of a place where you can completely relax. Imagine what this place needs to be like in order for you to feel calm and relaxed.

Start with the physical layout of the place you are imagining..... where is this peaceful place? You might envision somewhere outdoors.... or indoors..... it may be a small place or large one..... create an image of this place.

(pause)

Now picture some more details about your peaceful place. Who is in this place? Are you alone? Or perhaps you are with someone else? Are there other people present? Animals? Birds? Imagine who is at your place, whether it is you only, or if you have company.

(pause)

Imagine even more detail about your surroundings. Focus now on the relaxing sounds around you in your peaceful place.

Now imagine any tastes and smells your place has to offer.

Imagine the sensations of touch... including the temperature, any breeze that may be present, the surface you are on.... imagine the details of this calming place in your mind.

Focus now on the sights of your place - colours, shapes.... objects.... plants..... water..... all of the beautiful things that make your place enjoyable.

To add further detail to this relaxing scene, imagine yourself there. What would you be doing in this calming place? Perhaps you are just sitting, enjoying this place, relaxing. Maybe you imagine walking around.... or doing any other variety of activities.

Picture yourself in this peaceful place. Imagine a feeling of calm..... of peace..... a place where you have no worries, cares, or concerns.... a place where you can simply rejuvenate, relax, and enjoy just being.

(pause)

Enjoy your peaceful place for a few moments more. Memorize the sights, sounds, and sensations around you. Know that you can return to this place in your mind whenever you need a break. You can take a mental vacation to allow yourself to relax and regroup before returning to your regular roles.

In these last few moments of relaxation, create a picture in your mind that you will return to the next time you need a quick relaxation break. Picture yourself in your peaceful place. This moment you are imagining now, you can picture again the next time you need to relax.

When you are ready to return to your day, file away the imaginary place in your mind, waiting for you the next time you need it.

Turn your attention back to the present. Notice your surroundings as your body and mind return to their usual level of alertness and wakefulness.



For peer review only - http://bmjopen.bmj.com/site/about/guidelines.xhtml

Imperial College London

Keep with you the feeling of calm from your peaceful place as you return to your everyday life.

## *Part IV: Supervision of participants post-exercise and education - 20 minutes*

**Rationale:**
Because of an increased risk of arrhythmia and hypotension following exercise, a period of 15-20 minutes supervision is adopted before participants go home. This time will be used for education sessions.

### *Proper Exercise*

*Yoga versus physical culture*

Aim:

- For the group to understand the importance of taking up physical activity in cardiac rehabilitation
- For the participants to understand that yoga improves not only flexibility but strength, balance and cardiovascular function

Brainstorm question: What are the differences between yoga and physical culture?

Explore the answers and highlight the following:

- Yoga regards the body as a vehicle for the soul in its journey towards perfection
- Yoga promotes gentle movement whereas physical culture emphasises violent muscle movements
- Muscle development does not necessarily mean a healthy body
- Health is a state wherein all organs function perfectly under intelligent control of mind
- Asanas are designed to develop not only the body but also broaden the mental faculties and spiritual capacities
- The body is as young as it is flexible, so yoga postures primary focus on the health of the spine, its strength and flexibility
- Asanas work on the internal machinery of the body, the glands and organs as well as the muscles
- Hand in hand with the practice of yoga postures we practise deep breathing and concentration of the mind

### *Proper Breathing*

*Yogic breathing*

Observe your breathing for a while. How would you describe your breath?

Ask participants to share their observation and then highlight the following:

- Yoga philosophy claims we are allotted a certain number of breaths per lifetime. How we choose to illustrate that then becomes our practice of longevity. Breathing is the first thing we do when we are born and the last thing we do when we die. Practice observing your breath as often as possible.
- Most people use only a fraction of their potential lung capacity when breathing



For peer review only - http://bmjopen.bmj.com/site/about/guidelines.xhtml

Imperial College London

- There are three types of breathing: clavicular, intercostal and deep abdominal.
- A full yogic breath combines all three types of breathing
- Yogic breathing exercises are called pranayama which means to control the prana-subtle energy. Pranayama begins by controlling the motion of the lungs, by which the prana is control.
- Yogic breathing exercises might be very useful in process of quitting smoking

## *Proper Relaxation-Savasana*

Brainstorm question: How do we relax?

Explore the answers and highlight the following:

- When the body and mind and the mind are constantly overworked, their natural efficiency diminishes
- Modern social life and entertainment make it difficult for people to relax by over stimulating the nervous system
- By learning to relax we learn to economise the energy produced by our body as well as regulate and balance the work of the body and mind
- In ordered to achieve perfect relaxation, three methods are used for yogis: physical, mental and spiritual relaxation
- The relaxation position is known as Savasana, the 'Corpse pose'

## *Proper Diet-Vegetarian – Part I*

*'You are what you eat'*

Discuss and highlight the following:

- Proper yogic diet is lactovegetarian one based on simple, natural and wholesome food
- According to yogic philosophy all of Nature, including our diet, is categorised into three qualities (Gunas): sattvic (pure), rajasic (overstimulating) and tamasic (putrified)
- Sattvic food increases vitality, energy, vigor, health and joy
- Food should be as fresh and natural as possible, preferably organically grown
- Sattvic food include:

  **Grains:** corn, barley, wheat, unpolished rice, oat, millet and quinoa.
  Grains supply necessary carbohydrates, the main source of energy for the body, and they also contain about half the amino acids that are needed to form protein.
  **Protein foods:** legumes, nuts and seeds

  **Fruits:** both fresh and dried, as well as pure fruit juices


For peer review only - http://bmjopen.bmj.com/site/about/guidelines.xhtml

**Vegetables:** they contain minerals, vitamins and fibre. There are best eaten raw or cooked as lightly as possible

**Herbs:** for seasoning and herbal tea

**Natural sweeteners:** honey, molasses, maple syrup, and apple juice concentrate. White sugar is best avoided in a healthy diet.

**Dairy products:** milk, butter, cheese and yogurt

## *Proper Diet-Vegetarian – Part II*

*Guidelines for healthy eating*

Recap from the previous 'Proper diet' session and then highlight the following:

- Always respect your food and maintain a peaceful attitude during meals
- Do not eat when you are angry
- Do not eat food that is too hot or too cold, as this will upset your stomach
- Do not force yourself to eat anything you do not like, but also do not only eat foods that you like the most
- Abandon too many mixtures or combination of foods as they are difficult to digest
- Try to refrain from drinking during meals as this will dilute the gastric juice
- Eat slowly and savour your food
- Eat moderately, do not overload your stomach
- Try to eat at fixed times and try to refrain from eating between meals
- Try not to eat large meals at night
- Take some lemon and honey in the morning for health and energy and to purify the blood
- Do not practise asanas immediately after eating, nor when you are hungry
- Try sitting in Vajra Asana (sitting on the heels with knees and feet together) for 10 minutes after a meal to assist digestion

## *Positive Thinking and Meditation – Part I*

*Practical approach to meditation*

Discuss and highlight the following:

- Before we can learn to meditate we have to be able to concentrate
- Concentration means attending fully to one thought or object for a substantial length of time


For peer review only - http://bmjopen.bmj.com/site/about/guidelines.xhtml

Imperial College London

- Concentration exercises energise the mind, boosting efficiency at work and in the other tasks, while building will-power and the ability to influence other people positively

**Exercise: Listen to a sound**
Now listen carefully to the ticking of a watch. When your mind wanders, bring it back to the sound. How long can you concentrate on that sound?
*Exercises to practice at home-leaflet to be given*

**Lose yourself in a book**
Read two or three pages of a book, giving them your full attention. Then test your concentration by stopping at the end of a page. How much do you remember of the story? Can you classify, group or compare the facts you have been reading about?

**Contemplate nature**
During the day, concentrate on the sky. Feel your mind expand as you reflect on its vast expanse. At night, concentrate on the moon or stars. By the sea, focus on waves. Or shift your gaze between objects near and far, such as a nearby tree and a distant mountain.

**Focus on a flower**
Sit comfortably with your eyes closed.  Imagine a garden with many flowers. Gradually, bring your attention to a single flower. Visualise its colour and explore its other qualities, such as texture, shape, and scent. Concentrate on the flower's qualities for as long as possible.

## *Positive Thinking and Meditation – Part II*

*Practical approach to meditation*

Ask participants if they had chance to practice any of the concentration exercises then discuss and highlight:

- Meditation is a state of relaxed awareness
- The more care and attention you give to your preparation for meditation, the more positive the results will be
- Get the atmosphere right for meditation:

  **Place**: It is best to separate one portion of a room to use for your practice. Keep it clean and tidy, and place a candle or spiritually uplifting picture there. Burning incense can also help to create a meditative mood.

  **Time**: The best times for meditation are at dawn and dusk. Alternatively, find a time when you are free from daily activities and your mind can be calm.

  **Habit**: Practise every day at the same time. As your subconscious mind gets accustomed to the regularity, you will find it easier to settle and focus.

  **Sitting position**: Sit on the floor to meditate, in position that you can maintain comfortably, keeping your spine and neck straight but not tense. A simple, crossed-legged pose makes a firm base. Sitting on the cushion helps the thighs to relax and bring brings knees closer to the ground.

  If you cannot sit on the floor easily, sit on a comfortable chair with your ankles crossed.


For peer review only - http://bmjopen.bmj.com/site/about/guidelines.xhtml

Imperial College London

**Breathing**: Once you are sitting comfortably, relax your body as much as possible. Broaden your chest and lift your rib cage to encourage abdominal breathing. Then inhale and exhale rhythmically for about 3 seconds each, gradually slowing your breath down.

## *Making Positive Changes in Your Life*

Topic to reflect: Think about one change you would like to make (if any) to make your lifestyle healthier.

Ask if anyone would like to share their idea then suggest the following changes to make within first two months of practising yoga.

- **Proper exercise:** Try to practise asanas regularly
- **Proper breathing:** Practice deep abdominal breathing
- **Proper relaxation:** Learn Corpse pose and try to relax for 15 minutes daily
- **Reduce negative dietary habits:** Cut down or eliminate meat and cut down on fried food
- **Reinforcing positive dietary habits:** Drink 4 to 5 glasses of water and eat one raw salad daily
- **Eradicating negative habits:** If you smoke replace it with abdominal breathing
- **Concentration exercises:** Practice listening and hearing what others are saying
- **Positive thinking:** Refrain from using abusive language and try to spend time with people who have a positive outlook on life
- **Meditation:** Sit silently for at least 20 minutes daily with the mind focused on breath
- **Study:** Read something of inspiration daily

DRAFT
For peer review only

Imperial College London

# Appendix 1

## *Policy for cardiac rehabilitation in Ealing*

### INTRODUCTION

Cardiac disease is the leading cause of death in United Kingdom and is the leading cause of hospitalisation for both men and women. Cardiac rehabilitation programmes are recognised as a way to enhance recovery following acute cardiac events and encourage behaviour aimed at the secondary prevention of coronary artery disease. The key elements of cardiac rehabilitation are contained in the definition produced by the Scottish Intercollegiate Guidelines Network (SIGN): Cardiac rehabilitation is the process by which patients with cardiac disease, in partnership with a multidisciplinary team of health professionals, are encouraged and supported to achieve and maintain optimal physical and psychological health.

Cardiac rehabilitation is defined by the World Health Organisation as:
*".. the sum of activities required to influence favourably the underlying cause of the disease, as well as the best possible, physical, mental and social conditions, so that they (people) may, by their own efforts preserve or resume when lost, as normal place as possible in the community. Rehabilitation cannot be regarded as an isolated form or stage of therapy but must be integrated within secondary prevention services of which it forms only one facet".*

The provision of skilled help, support and supervision that is tailored to individual patients can: a) help people understand their illness and its treatment; b) provide psychological and emotional support; c) improve people's success in making beneficial lifestyle changes; and d) help people make the transition back to a full and as normal life as possible. (NSF: cardiac rehabilitation, 2007)

The 2007 NICE guidelines on "secondary prevention for patients following a myocardial infarction" state that cardiac rehabilitation should be equally accessible and relevant to all patients after an MI, particularly people from groups that are less likely to access this service. These include people from black and minority ethnic groups, older people, people from lower socioeconomic groups, women, people from rural communities and people with mental and physical health co morbidities.

The British Association of Cardiac Rehabilitation (BACR) standards 2007 defined the core components of cardiac rehabilitation as lifestyle (physical activity and exercise, diet and weight management, smoking cessation), education, risk factor management, psychosocial, cardio protective drug therapy and implantable devices, and long-term management strategies (4).

Four phases of cardiac rehabilitation were defined by the BACR and endorsed by the National Service Framework (2007) for CHD in England and Wales and SIGN for Scotland (2002). Each phase represents a different component of the journey of care. Phase 1 is generally concerned with the in-patient episode with Phases 2-4 following the patient from early discharge to long-term maintenance.

According to the NSF goal, every hospital should ensure that:
more than 85% of people discharged from hospital with a primary diagnosis of acute myocardial infarction or after coronary revascularisation are offered cardiac rehabilitation and one year after discharge at least 50% of people are non-smokers, exercise regularly and have a BMI <30 kg/m$^2$; these should be demonstrated by clinical audit data no more than 12 months old. Trusts should agree, implement and audit a detailed plan and protocol for identifying, treating and following up their patients who may benefit from cardiac rehabilitation.

### THE CARDIAC REHABILIATION PROGRAMME IN EALING HOSPITAL NHS TRUST

The aim of the comprehensive cardiac rehabilitation programme is to reduce the risk of subsequent cardiac problems and to promote the return to a full and normal life. The provision of a cardiac rehabilitation service for all eligible patients is clearly desirable for health and economic reasons.


For peer review only - http://bmjopen.bmj.com/site/about/guidelines.xhtml

Imperial College London

Comprehensive help with lifestyle modification involving education and psychological input as well as exercise training can reduce mortality by 20-25% over 3 years. ( Oldridge et al 1988; O'Connor et al 1989)

**1. TARGET CLIENT GROUPS**
Patients admitted with or who have undergone the following will be eligible for the programme.

- NSTEMI
- STEMI
- Acute Coronary Syndrome
- Revascularisation
- CABG
- Valve surgery
- Heart Failure

All the above patients admitted to Ealing Hospitals will be offered a choice as to where their cardiac rehabilitation will take place.

**2. IDENTIFYING PATIENTS**

- Patients are identified through CCU/ITU, cardiology and general medical wards, cardiology out patients, and from waiting lists for revascularisation procedures.
- Referrals are accepted from other acute trusts (using North West London Cardiac Rehabilitation referral form); Ealing Hospital cardiac catheter laboratory, as well as the community referrals from GP's, Practice Nurses, Community Specialist Clinics.

**3. PHASE 1** (Before discharge from hospital)

Where possible the Cardiac Rehabilitation Specialist Nurse will visit the patient and his / her family during the hospital stay. The following will be carried out during this phase:
• assessment of physical, psychological and social needs for cardiac rehabilitation
• negotiation of a written individual plan for meeting these identified needs
• initial advice on lifestyle e.g. smoking cessation, physical activity (including sexual activity), diet, alcohol consumption, driving and employment
• review of prescription of effective medication and education about its use, benefits and harms
• involvement of family members and/or relevant informal carer(s)
• provision of information about cardiac support groups
• provision of locally relevant written information about cardiac rehabilitation

It is important to establish rapport and therapeutic relationship with every patient and involve family or/and carers from this early stage. This will increase the likelihood or patient's participation in consecutive phases of cardiac rehabilitation programme and reduce a risk of DNA incidences.

The "Edinburgh Heart Manual" for education, exercise and stress management components can be given to eligible patients at this stage. Social needs and preferences of patients will be identified and taken into account for a purpose of structuring of individually tailored cardiac rehabilitation programmes.

Guidelines for Phase 1 Cardiac Rehabilitation Service will be followed ( see Appendix 1\)

BACR Guidelines for Secondary Prevention will be followed. They are:

- Risk factors of each patient should be identified and managed accordingly.
- All patients who smoke should be offered structured anti-smoking advice and, if necessary, specific


For peer review only - http://bmjopen.bmj.com/site/about/guidelines.xhtml

treatment.

- All patients after acute myocardial infarction or coronary revascularisation should be treated, if necessary, with lipid lowering therapy (diet control, statin therapy, lifestyle changes to include regular exercises), antiplatelet therapy (with aspirin, dipyridamole or clopidogrel), beta blockers, ACE inhibitors, other secondary prevention measures (better control of diabetes, hypertension, body mass index (BMI)).
- All patients with heart failure should be given advice on fluid balance (daily weights, fluid intake, diuretic dosages), salt restriction, avoidance of ethanol consumption and smoking, influenza vaccination, and considered for therapy with ACE inhibitors, beta blockers, spironolactone, angiotensin receptor blockers for prognostic benefits.
- Patients with other conditions should receive appropriate advice and treatment as secondary prevention of their specific cardiac conditions (e.g. avoidance of caffeine and treatment with beta blockers in patients with cardiac arrhythmia).

**4. PHASE 2** (Early post-discharge period)

During early post discharge period, support to patients can be provided by home visiting where appropriate, telephone contact and by supervised use of the Heart Manual.
Patient will be sent an invite letter for the first outpatient appointment in cardiac rehabilitation clinic of Ealing Hospital within 2 -3 weeks after discharge from hospital. The time tables with dates of currently run educational sessions will be included with an invite letter.( see Appendix 2). Where possible, patients will be asked to have their blood tests done in GP practices prior to appointment with cardiac rehabilitation specialist nurse. Patients will also be asked to bring their medication list and any outpatient appointments' letters with them.
During the consultation in OPD clinic, the individual needs, expectations of cardiac rehabilitation programme and wishes will be explored. The suitability for exercise programme ( a component of comprehensive cardiac rehabilitation programme) can also be assessed at this point.

Following will be carried out:

- Provision of general advice about the cardiac condition(s) and complications that the patient has, including risk factor management, medication (what they are for, adjustment of doses and potential adverse reactions), presentation of further events and what actions to take; symptom control advise
- Patients' misconceptions and undue fears or anxieties will be identified and addressed
- Patients will be advised on the stages towards resuming normal life (e.g. physical activity levels, sexual function, driving, flight, return to work, weight control, fluid balance, alcohol consumption).
- Advice will be provided to patients to address vocational, social, cultural, educational needs, and referral for occupational therapy assessment and management.
- Measurement of patient's body weight, calculation of body mass index (BMI) and central obesity (girth measurements)
- Review of fasting lipid profile +- advice/ appropriate referrals to GP/lipid clinic.
- Review of fasting blood glucose +- advice/ referral to diabetic specialist nurse/GP
- Dietary habit assessment and advice on healthy eating
- Blood Pressure will be measured and heart rate record
- If known to have diabetes a urine sample for microalbuminurea will be collected.
- Assessment of wounds and advice as necessary (post surgery patients)
- HADS +-quality of life (QoL) assessments will be carried out to estimate patients' health perceptions and to help detect patients with inappropriate levels of anxiety or depression, a small proportion of whom may need referral for specialist evaluation and treatment
- Advice how to stop smoking- for those who smoke+- referral to specialist services
- Review involvement with cardiac support groups
- Offer resuscitation training for family members
- Encouraging patients' immediate family members to engage in health improvement and lifestyle modification

"Client Feedback and Goal Planning Form" (see Appendix 3), as well as "Agreed Action Plan" (see Appendix 4) forms will be given to those who were not seen at Ealing Hospital during Phase 1. Patients will be encouraged to



For peer review only - http://bmjopen.bmj.com/site/about/guidelines.xhtml

For peer review only

participate in joint (with nurse specialist) care planning, goal setting, time-allocation for improvement and exploration of barriers to achievement of desirable results. This process is very important for achievement of lifestyle modification and behavioural change.
Patients will be encouraged to bring those forms to meetings with cardiac rehabilitation nurse, or/and to educational, or/and exercise programmes, so appropriate to the area of concern specialist will be able to answer the questions and give patient-tailored advice and recommendations.
Patients will be also given comprehensive information regarding diagnosis, procedures, practical advice and risk factor modification in written form. Patients will be issued with a wallet sized card that allows each patient to keep a record of his or her risk factors, including blood pressure, cholesterol and glucose, lifestyle modifications, dates of procedures and current medication.

Patients and their partners will be invited to enter an 6-12 week health promotion programme where patients will receive 1) on-going risk factor monitoring/ advice/support, 2) exercise sessions in a gym, led by cardiac physiologist or home-based exercise programme, 3) health education lectures (led by cardiac rehabilitation sister; pharmacist; dietician; clinical psychologist; cardiac physiologist), 4) relaxation sessions, 5) guidance and supervised use of “Edinburgh Heart Manual”.

Patients will be asked about their preferences on exercise programme, whether they would like to join exercise programme run in local Gym(s) or to have home based exercise programme ( e.g. using “Edinburgh Heart Manual” as guideline and/or using exercise plan prescribed by cardiac physiologist and/or using pedometers, etc).
Patients will be given information on forthcoming dates/ topics/ venues of educational programme. Patients themselves and their family members or carers will be encouraged to come to educational sessions together.
For patients whose English is limited, the interpreter services will be provided where possible. Patients will be encouraged to bring their English speaking relatives for a consultation. The information about each individual condition/treatment/recommendations can be ordered for patient from British Heart Foundation in a form of audio tape/video-tape/pocket size leaflets, etc
Those patients who will not respond to invitation letter to attend an OPD clinic will be contacted via a telephone. Rehabilitation staff will try to address the issues which might impede patient's decision about participation in a programme using individual approach. However, If a patient will state clearly that he/ she does not wish to participate, then patient's GP will be informed and patient will be discharged from CR service.

**4.1.Referrals:**

- Weight management and diet advice: All patients regardless of their cardiac condition will be referred to cardiac specialist dietician for cardio-protective diet advice and weight management programme if appropriate.
- Diabetes: Newly diagnosed and those patients with diabetes who are not well controlled will be referred to the community diabetes team.
- Erectile Dysfunction: patients experiencing sexual problems can be referred to the ED clinic at GSTT.
- Psychological Problems: Rehabilitation staff will do their best to identify and address cardiac misconceptions in patients with CHD in order to reduce possibility of anxiety or/and depression. Hospital anxiety and Depression Scale (HADS) will be used. Screening will take place at discharge (where possible), 6-12 weeks post MI or following a decision on surgical intervention; can be repeated every 3 months if necessary. Psychological interventions of cardiac rehabilitation programme, such as stress management, relaxation, goal setting, taking part in group exercise and education can relieve anxiety and mild depression. Patients who score persistently above 11 on the HAD scale can be considered for referral to a clinical psychologist for assessment/ interventions.

- **Health Education Talks** (please see appendix 2 for the current health education presentations)

Discussions will be given by a health care professional with specialist knowledge of the subject. This is an information giving session to increase patients knowledge.

The group discussion allows patients to explore the information given and how best to apply it to themselves and their families. These sessions are intentionally informal and encourage patients to recognise their own risk factors and develop strategies for change. The following topics are to be covered by the workshops:

1. Drugs for heart disease and how they work (presented by pharmacist)

DRAFT

For peer review only

2. Managing Stress (by clinical psychologist)
3. Eating for a healthy heart (by cardiac specialist dietician)
4. Exercise and the benefits for your heart (cardiac exercise physiologist)
5. Risk factors and making lifestyle changes (by cardiac rehabilitation sister)
6. The heart and how it works (by cardiac rehabilitation sister)

**5. PHASE 3 (**Four weeks after an acute cardiac event / 4-6 weeks post surgery)

Structured exercise as a therapeutic intervention is central to cardiac rehabilitation. Exercise training should form a core element of cardiac rehabilitation programmes (SIGN,2002).
At this stage, patients and their family members/carers should be aware of all the benefits of the physical exercise programme and should be committed to participate. Most patients will benefit from and will be encouraged to undertake at least low to moderate intensity exercise. However, patients with clinically unstable cardiac disease or limiting co-morbid illness will be excluded from exercise training. People whose potential to exercise is limited may have much to gain from the non-exercise components of cardiac rehabilitation.

**5.1. Contraindications to Exercise**

- Unstable Angina
- Unstable Ischemia
- Active pericarditis or myocarditis
- Hypertrophic obstructive cardiomyopathy
- SBP >180 mmHg or DBP >100 mmHg
- BP drop 20 mmHg during incremental exercise
- Resting/uncontrolled tachycardia >100
- Severe and symptomatic aortic stenosis
- Uncontrolled atrial or ventricular arrhythmias
- Severe pulmonary hypertension
- Heart failure that is not compensated
- Recent embolism
- Thrombophlebitis
- Unstable diabetes
- 30 AV block (without pacemaker)
- Febrile illness

**5.2. Exercise sessions.**

- The exercise sessions are held twice a week for 1 hour in St.Bernard's and Southall sport centres each week and patients are encouraged to attend between 8-12 sessions.
- Those patients who wish to participate in the exercise programme need to sign a consent form
- All patients need to attend an initial screening appointment and perform a sub maximal functional capacity test prior to attending the classes.
- Patients will be risk stratified into low, medium and high risk categories as defined by the American Association of Cardiovascular & Pulmonary Rehabilitation (AACVPR) as recommended by American College of Sports Medicine**.** (Appendix 5);"low risk" patients will be enrolled to attend a community sport centre, "moderate/ high risk" patients will be invited to participate in an exercise programme, based in a gym which in located in close proximity to Ealing Hospital.
- Patients, who will not want to attend formal taught sessions will be offered home-based exercise plan.

**Assessment before Exercise Classes:**

- Prior to participation in exercise training patients will undertake a submaximal functional capacity test (e.g. the 6 minutes walk test or shuttle walk test). This will usually be carried out by cardiac physiologist during a separate appointment.
- Prior to submaximal testing of functional capacity a pre-screening checklist will be completed to ensure suitability, an end point of 80% HR max will be determined (adjusted as appropriate for high risk patients) and a rating of perceived exertion of 15, using the Borg Scale category ratio 6-20 scale (Appendix 4). Prior to participation in the exercise test the patient will be familiarized to the Borg scale as below

DRAFT
For peer review only

Imperial College London

- Target heart rates for the exercise classes will be set to between 60-75% of the maximal heart rate minus 30 if on Beta Blockers. This range can be adjusted based on risk stratification. The range will be written in patient's exercise plan.

**Before the Class Begins**
- Brief discussion with patients about their progress, home exercises, changes, concerns
- BP and pre-exercise heart rate will be recorded
- Blood glucose levels checked for diabetic patients
- Equipment set up in advance
- Those patients who will complain of feeling generally unwell or become symptomatic or clinically unstable can be excluded from a session for a day. Depending on a condition, the symptoms will be treated with existing medications; patient will be either accompanied to A&E ( if severely unwell) or referred to GP.

**The Exercise Components.**
All sessions should include:

**Warm-Up (15 minutes minimum):** The warm-up period will include graduated low intensity aerobic exercise and short dynamic stretches to increase myocardial blood supply, soft tissue flexibility and mobilize joints.
**Circuit ( 20-30 minutes):** All patients will participate in a progressive exercise training programme, which is modified to meet individual need.
**Cool Down (10 minutes):** This will include graduated low intensity exercise and muscle stretching. Once complete HR will be rechecked and recorded (aim to be within 10 beats of pre-exercise rate)
**Relaxation (15 minutes minimum):** Following the exercise class, patients should be supervised for a 15 minute period.

**Health and Safety Requirements.**
- Each patient will be risk stratified as described above.
- Exercise will be delivered by experienced staff with training in exercise physiology and prescription and an understanding of the specific needs of cardiac patients in relation to exercise.
- 3 members of staff where possible - minimum ratio 1:5. This includes visitors. However priority for exercise is given to patients and if the numbers exceed the safety requirements, visitors will not be able to join in on that occasion.
- Cardiac physiologist to be trained BLS skills (minimum), cardiac rehabilitation nurse-to be present at each session and to be trained at ILS level (minimum)
- Resuscitation equipment available in the gym for the duration of the class.
- All visitors who wish to join in the exercise class need to complete a ParQ
- Venue must be suitable i.e. adequate space, temperature (65-72F,18-23C),
- Drinking water should be available.
- Immediate access to a telephone.
- Annual environmental risk assessment

**Monitoring patients**
- Heart rate monitors to be worn during a patient's first session.
- Heart rates recorded at the beginning of each class, during the class and after the cool down.
- Borg scale of perceived exertion will be recorded during exercise.

**Patients with diabetes**
- Record blood glucose level before the start of the exercise
- Avoid exercise if glucose is over 16mmol/L.
- Avoid exercise if glucose is under 6 and no snack is available prior to exercise
- If glucose >13 and <16 then do warm up and retest – level should fall. If remains >13 and rising should not continue exercise until their status has been stabilised.
- Those taking insulin should avoid injecting into subcutaneous tissue of thigh i.e. avoid sites near to exercising muscle groups.
- Avoid exercise during peak insulin times.


For peer review only - http://bmjopen.bmj.com/site/about/guidelines.xhtml

Imperial College London

**Medical Emergencies**

- Nurse to stay with patient
- Exercise specialist to ensure safety of other patients
- Third person to call for help (999 and/or cardiologist)

**DNA Policy**

Patients will be informed about current DNA policy and their obligation to notify a cardiac rehabilitation nurse or cardiac physiologist if they have to miss a session. If a patient does not attend two consecutive classes, contact will be initiated by a member of the Cardiac Rehabilitation team and if no response is received then the patient is discharged from the programme and a letter sent to patient's GP as well as patient.

**End of Programme.**

On completion of the programme patient is given a Certificate stating patients achievements ( see Appendix 6) and a re-screening appointment is made in 2-4 weeks. Patient's GP will receive a letter from Cardiac Rehabilitation Team with all relevant information.

**5.3. Home-Based Programmes**

Those patients who would prefer a home programme or are unable to attend the group sessions will be assessed by the cardiac physiologist and given a suitable physical activity programme. Progress will be monitored regularly and risk factor management will continue as required. This may involve the patient attending regular appointments with the cardiac nurse for blood pressure/heart rate/ blood results monitoring/relevant support and advice. The patient is offered the opportunity to attend the health education talks where possible.

**6. RE-SCREENING OF PATIENTS**

On completion of the health promotion sessions all patients and their families are invited back to the Cardiac Rehabilitation OPD clinic where they will be reassessed as follows:

- Cardiac Risks will be assessed again and progress recorded
- Blood pressure, heart rate, lipids and glucose levels are repeated and recorded
- Those with diabetes will have their HbA1c checked
- Current medications therapy is reviewed
- HAD and QOL is repeated
- 6 MWT or Shuttle walk test is repeated Diet is reassessed and long term recommendations made
- BMI and girth measurement is checked again and recorded

Patients with stable coronary disease will be encouraged to continue regular moderate intensity aerobic exercises. The relevant information about the exercises, stretching techniques, relaxation exercises, and all available sport/leisure centres in the area will be given to patients on discharge. Information about local yoga/dancing/swimming/golf classes, etc. will be available on request. Individual approach will apply, hence if someone will prefer to carry on home based exercises he/she will be supported in their decision. Others, who prefer formal class based cardiac exercise programmes can be referred to the Phase 4 exercise sessions held in St.Bernard and Southall sport centres. The exercise sessions are lead by BACR trained exercise physiologists.

**7. PHASE 4** (Long-term maintenance of changed behaviour)

Long term follow-up in primary care will be arranged.
Involvement with local cardiac support groups or groups of interest (e.g. gardening, cooking, walking, cycling, etc.) will be offered.
Referral to specialist cardiac, behavioural (e.g. exercise, smoking cessation) or psychological services will be made, if clinically indicated.

**8. ANNUAL REVIEW**



For peer review only - http://bmjopen.bmj.com/site/about/guidelines.xhtml

All patients are invited to attend a follow-up appointment one year after completion of the programme. At this appointment fasting lipids, glucose, and, if appropriate, and HbA1C are measured. Blood pressure is checked twice and anthropometry is recorded. A physical activity and brief dietary assessment are carried out. A summary is sent to the GP and patient with further recommendations if appropriate.

## 9. INTERGRATING CARE BETWEEN SECONDARY & PRIMARY CARE.

A seamless transition between hospital provision of cardiac rehabilitation and the continuing support provided by primary care practitioners requires good communication between all involved in the care of patients with CHD. The primary care team, with detailed knowledge of an individual's social and medical background, includes professionals who are likely to be aware of the implications of CHD for both the individual and their family. Accurate information shared between the various members of multidisciplinary teams across both primary and secondary care will enable the best possible care to be given to the patient.

## 10. AUDIT & EVALUATION ( TO AGREE ON EVALUATION OF WORK)

The Cardiac Rehabilitation service will carry out clinical audit using routinely collected data. Long term goals can be monitored by observing changes over time in incidence and mortality from CHD.

Data will be collected onto the CR database and will also be exported to the national database annually as required for a purpose of NACR. On completion of the programme, patients will be asked to fill in a satisfaction questionnaire.

Standards that we need to follow:

The service should be referred to in the HImP and reflected in long term service agreements.

A clear description of the district cardiac rehabilitation programme should be available to the public, to service providers and to commissioners and should be cited in the HImP. This description should include details of:

- the patients to be offered cardiac rehabilitation
- staffing (including details of the skills and training required)
- the location and timetable of service provision
- audit criteria
- investment and resources.

Whatever the detail of local rehabilitation services, records should be kept so that the service can be audited against nationally recommended guidelines. This should include information about ethnicity so that it is possible to monitor equity of access. Audit will be easier to undertake if data is stored electronically in a way that allows ready analysis.
National Service Framework – Coronary Heart Disease

**Clinical audit**

Clinical audit – the systematic assessment of the quality of care – is an essential component of modern, high quality health care. It will also be an essential component of effective clinical governance embracing all health professionals.
Trusts should work with their local PCTs and their constituent practices to undertake clinical audit that allows them to review annually the items listed in **bold** below. They may also wish to review the other items when it becomes possible to collect these data.

1) number and % of patients discharged from hospital after coronary revascularisation OR with a primary diagnosis of AMI with documentation of arrangements for cardiac rehabilitation in discharge communication to GP (by Trust and PCG/PCT and by sex,

Imperial College London

age 35-74iii years, and ethnic group)

2) number and % of patients discharged from hospital with a primary diagnosis of CHD recruited to a cardiac rehabilitation programme by Trust and PCG/PCT and by sex, age 35-74iii years, and ethnic group

3) total number and % of those recruited to cardiac rehabilitation who have an individualised plan for rehabilitation and secondary prevention before discharge from hospital
4) total number and % of those recruited to cardiac rehabilitation who, one year after discharge, report:
• regular physical activity of at least 30 minutes duration on average 5 times a week
• not smoking
• BMI < 30 kg/m2.

(NB. PCTs and rehabilitation services may wish to collaborate in the collection, analysis and interpretation of their audit data to avoid duplication of effort and to gain a more complete picture of the quality of rehabilitation and secondary prevention services.)

This Policy will be reviewed and updated if necessary on annual bases.

**REFERENCES**


1. Scottish Intercollegiate Guidelines Network (2002) Cardiac Rehabilitation. A national clinical guideline. SIGN guideline 57. http://tinyurl.com/27g33c

2. National Institute for Health and Clinical Excellence (2007) MI: secondary prevention. Clinical Guideline 48. May. http://tinyurl.com/38tom3

3. British Association of Cardiac Rehabilitation (2007) Standards and Core Components for Cardiac Rehabilitation. http://tinyurl.com/3ydagw

4. Department of Health (2000) Coronary Heart Disease: National Service Frameworks. HMSO: London

5. World Health Organisation (1993) Needs and action priorities in cardiac rehabilitation and secondary prevention in patients with CHD. Geneva: WHO regional office for Europe

6. American College of Sports Medicine (1991) Guidelines for exercise testing and prescription, 4th edn. Lea and Febinger, Philadelphia


DRAFT For peer review only



For peer review only - http://bmjopen.bmj.com/site/about/guidelines.xhtml

# Appendix 2

## *Ealing Cardiac Rehabilitation Health Education Talks*

*Drugs for heart disease and how they work*

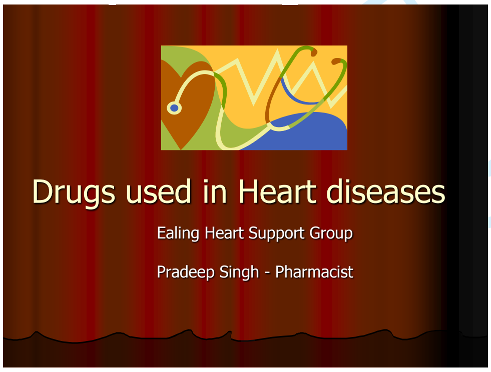


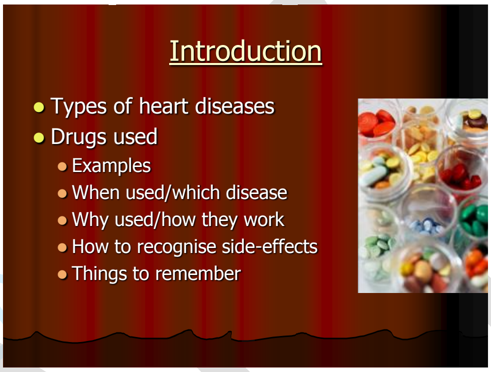


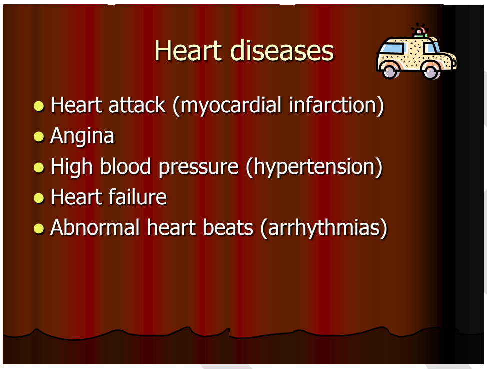


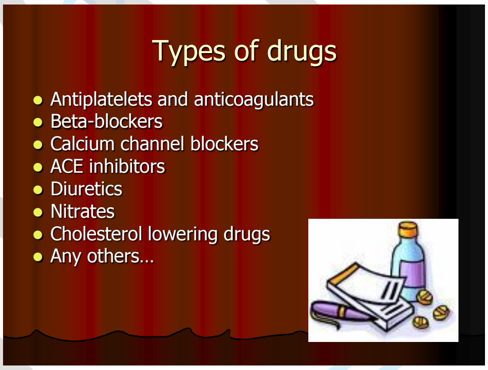


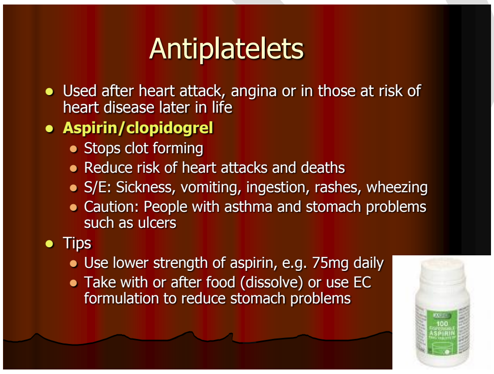


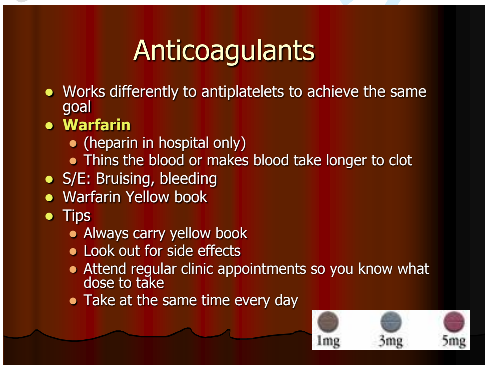

Imperial College London

## Beta-blockers

- Atenolol, bisoprolol
- Used after heart attack, angina, high BP, abnormal heart beats, heart failure
  - Works in many ways
  - Reduce related deaths
  - S/E: fatigue, wheezing, cold hands/feet, sleep problems, heart failure
  - Caution: Asthma and diabetes
- Tips
  - Do not stop taking them suddenly

## Calcium channel blockers

- Amlodipine, diltiazem
- Angina, high BP
  - Widen blood vessels
- S/E: Headache, upset stomach, ankle swelling, flushing
- Tips
  - Use once daily preparations to reduce s/e. For example, Dilzem XL.

## ACE inhibitors

- Lisinopril, Perindopril
- High BP, after heart attack, heart failure
  - Widen blood vessels
  - Reduce related deaths
- S/E: dry cough, low BP (first dose), taste disturbances, sore mouth, rashes, allergy- type reactions
- Comments: can interfere with kidneys, cause salt disturbances

## Diuretics (water tablets)

- Furosemide, Bumetanide
  - Used in heart failure
- Bendroflumethiazide
  - High BP
  - Reduce fluid by increase volume of urine
- S/E: Gout, worsen diabetes, affect salts
- Comments:
  - Often combined with other drugs
  - Salt disturbances reduced with co-amilofruse
  - Take morning or early afternoon

## Nitrates

- Glyceryl trinitrate, Isosorbide mononitrate
  - Used in Angina treatment and prophylaxis
  - Works by widen the blood vessels in the heart muscle which may be partly blocked
- S/E: Headache (temporary) flushing
- Comments:
  - Tablets/spray for under tongue, patches
  - Tablets to swallow
  - Paracetamol usually helps the headache

## Cholesterol lowering drugs

- Simvastatin, Atorvastatin, Pravastatin
  - After heart attack, angina
  - Reduce cholesterol production
  - Reduce heart disease events
- S/E: Muscle weakness, liver effects, headache
- Comments:
  - Take at night
  - Use with dietary advise
  - Care in liver disease
  - Any muscle problems – contact doctor immediately

## Other medicines

| | How it works | Side effects | Interactions |
|---|---|---|---|
| Digoxin | Increases force that heart pumps blood and reduces heart rate | Nausea, vomiting, slow pulse | Levels in the blood are increased by amiodarone, diltiazem, verapamil, dihydropyridines |
| Amiodarone | Antiarrhythmic – used to correct irregular heart rhythms | Sun sensitivity. Changes in thyroid function. Deposits in the cornea | Increases digoxin and warfarin. If given with other antiarrhythmics get and additive effect |
| Warfarin | Thins the blood | Bruising, bleeding (nose, urine, etc) | Effect increased by alcohol, antibiotics, amiodarone, cimetidine, simvastatin |

## What are medicines for the heart used for?

| | Nitrate | Beta blockers | Calcium channel blockers | ACE inhibitor | Diuretic | Digoxin |
|---|---|---|---|---|---|---|
| Angina | Y | Y | Y | | | |
| Raised blood pressure | | Y | Y | Y | Y | |
| Heart failure | Y | | | Y | Y | Y |
| Arrhythmias | | Y | Y | | | Y |


For peer review only - http://bmjopen.bmj.com/site/about/guidelines.xhtml

Imperial College London

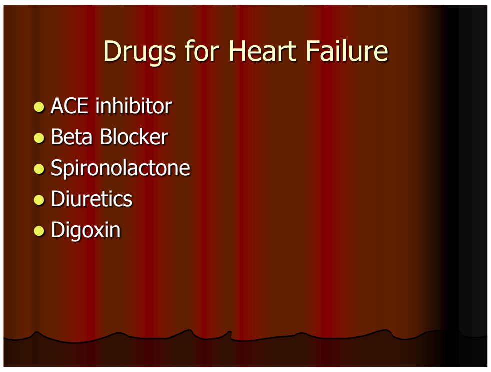


*Managing Stress*

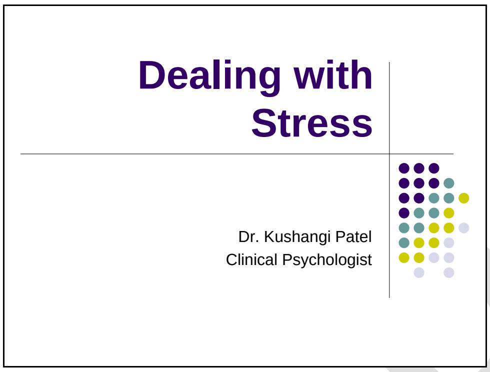


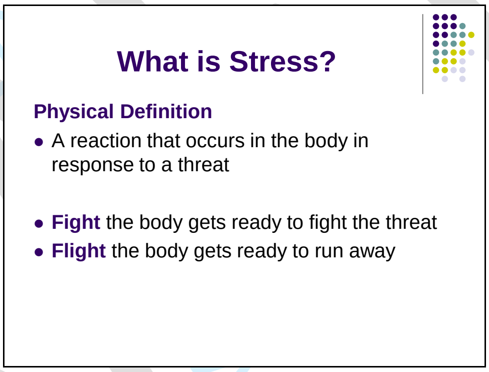


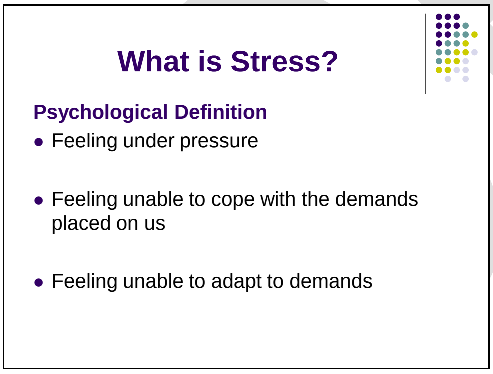


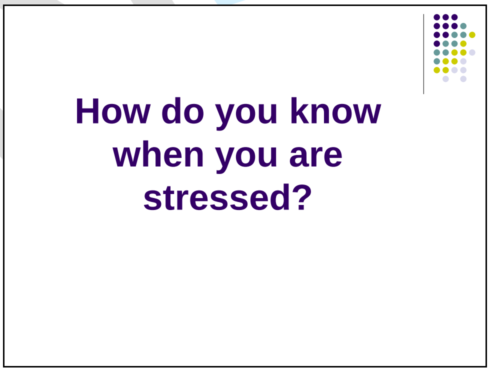


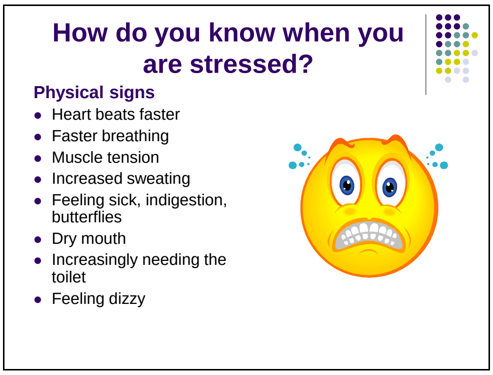


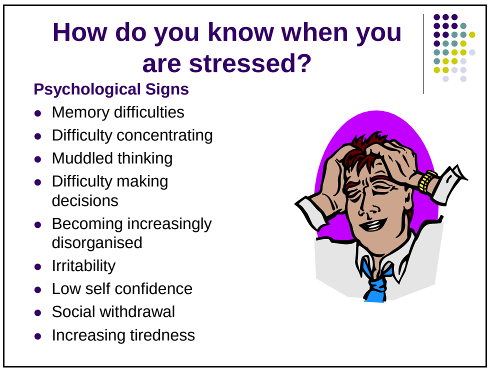




For peer review only - http://bmjopen.bmj.com/site/about/guidelines.xhtml

Imperial College London

## Purpose of stress

- Stress does have a purpose it's not all bad
- To protect ourselves from threat
- A small amount of stress helps increase our performance at things
- Stress can drive us to do things in life

## What makes you stressed?

**Environment**

- Work, family, too many social demands, driving, money, not enough opportunity for enjoyment

**Thoughts**

- I can't cope! I haven't got time! If I don't get this done….. will happen!
- These are e.g's of negative thoughts and predicting worse case scenarios.

## What causes stress in your life?

## What makes you stressed

**Emergency**

- Threat to life/ safety
- Events that pose an immediate threat to us cause stress levels to increase sharply and then fall quickly

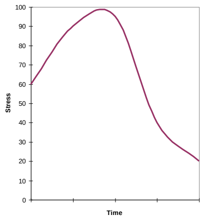


## What makes you stressed

**Daily Hassles**

- Such as shopping, organising holidays/social events, keeping on top of daily tasks at home and/or work.
- These frequently affect our stress levels, increasing it and decreasing it as hassles are encountered and then dealt with.
- Watch out for daily hassles – these lead to a gradual but **regular** build up of stress.

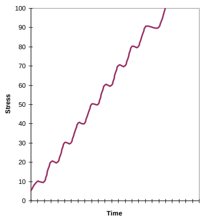


## How stress causes health problems

- Stress causes heart rate and blood pressure to increase and hormones are released to enable us to fight or flight.
- If high and/or frequent stress is experienced and these hormones are not used up by physical activity, the increase in heart rate and blood pressure damages arteries.
- The body heals this damage but as a result the artery walls become thicker and scarred.
- This can affect the supply of blood and oxygen to the heart.

## How health problems cause stress

- Health problems can affect all areas of life.
- Physical – feeling more tired, less able to do the things accomplished before the health problems started.
- Financial - time off work or a need to leave work altogether may have financial implications.
- Interests – a persons ability to engage in things they enjoy may be reduced temporarily or certain hobbies may have to be adapted.

## How health problems cause stress

- Identity – we all have views of ourselves. E.g. independent, responsible, fun, healthy. A health problem may affect this view if we feel we are no longer able to "be" these things.
- All these aspects can lead to feelings of frustration, anger and sadness.
- This can all increase stress levels
- Talk to other people about these feelings and frustrations or seek help from professionals, GP, Cardiac Rehab Nurse.



For peer review only - http://bmjopen.bmj.com/site/about/guidelines.xhtml

Imperial College London

## Health problems and stress

- Heart problems can knock self confidence so it is important to do things at a comfortable pace and build up gradually.
- Work to balance doing things at YOUR pace. Doing nothing and doing too much are both unhelpful.
- Take advice from GP's, cardiac team on how much you can do and when.
- Watch out for negative thoughts as they can lead you into a vicious cycle of inactivity and low mood which all can increase your stress levels.

## Vicious cycle of negative thoughts

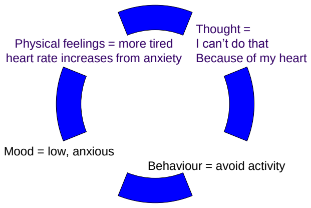


## What are the consequences of stress?

## Consequences of stress

- Health problems
- Difficulty in relationships
- Increase in sick leave
- Being unable to do things you need to do or enjoy
- Feeling upset, angry, frustrated, out of control

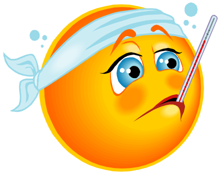

## Exercise

- In pairs talk about how you have coped with stress.
- What sort of things have you done in response to stress?
- What has worked?
- What has not worked so well?

## Coping with stress

**Helpful ways**

- Exercise
- Talk to someone
- Look at my thinking
- Prioritise tasks
- Share the load
- Relaxation
- Get guidance from other people or professionals involved in your care

**Unhelpful Ways**

- Increase alcohol/other drugs
- Work longer hours/ harder to finish tasks
- Shout
- Be on my own
- Ignore it

## Coping with stress

**Notice your early warning signs** – these may be physical signs and/or psychological signs

**Take action**

- **Calm your body by** –
- Relaxation
- Regular physical activity helps keep overall stress levels low
- Eat healthily
- Do something you enjoy
- Rest if you're tired
- Avoid increasing alcohol, smoking or caffeine to cope

## Coping with stress

- **Calm your mind by** –
- Relaxation
- Looking at your thoughts:
  Are you focusing on the negative?
  Are you predicting the worse case scenario without any evidence for it?
- Plan and prioritise
- Think about the long-term – is saying no now going to help you later on

**It may be difficult to do all the techniques so find out what works for you!**


For peer review only - http://bmjopen.bmj.com/site/about/guidelines.xhtml

Imperial College London

*Eating for a healthy heart*

This is an interactive session delivered by a cardiac specialist dietician – no presentation available.

DRAFT

For peer review only



For peer review only - http://bmjopen.bmj.com/site/about/guidelines.xhtml

Imperial College London

*Exercise and the benefits for your heart*

## Exercise & Physical Activity

Amir Zamani

## Introduction

- Physical activity, benefits and preventative effects
- Exercise intensity and RPE
- FITT
- Contraindications
- Angina and exercise
- GTN and general advice on chest pain
- Walking programme
- Important points to remember
- Specific activities and tasks

## TERMINOLOGY

Physical activity

- movement involving skeletal muscles and resulting in energy expenditure

EXERCISE

- planned, structured physical activity aimed at physical fitness

## Some good news

- Help lower your blood pressure
- Improve your blood cholesterol levels
- Reduce your risk of diabetes
- Help you to lose weight
- Reduce your angina
- Reduce your risk of having stroke
- Help you to return to work
- Reduce risk of dying

## Exercise intensity

- 1 - Talk Test
  - can you have a conversation?
- 2 - Listen to your body
  - Muscles
  - Sweating heavily
  - Dizzy, nauseous, very short of breath.
  - Do you feel completely exhausted
- 3 - Effort Scale

## Rate of perceived exertion

| | | |
|---|---|---|
| 0 - | NOTHING AT ALL | (No Intensity) |
| 0.5 | EXTREMELY LIGHT | ( Just Noticeable) |
| 1 - | VERY LIGHT | |
| 2 - | LIGHT | (Light) |
| **3 -** | **MODERATE** | |
| **4 -** | **SOMEWHAT STRONG** | |
| 5 - | STRONG | (Heavy) |
| 6 - | | |
| 7 - | VERY HARD | |
| 8 | | |
| 9 - | EXTREMELY STRONG | ( Almost max) |
| 10- | MAXIMUM | have to stop now! |



For peer review only - http://bmjopen.bmj.com/site/about/guidelines.xhtml

Imperial College London

## FITT

- Frequency Most days
- Intensity Moderate
- Time 30-40 minutes
- Type Aerobic

## STOP if you experiencing any:

- Undue shortness of breath
- Chest pain/discomfort (or pain in your neck/jaw/arm)
- Nausea/headaches/dizziness
- Inappropriate tiredness
- Persistent palpitations
- Feeling unwell

## Angina and exercise

- Angina is often described as a tightness, heaviness or dull sensation in the chest
- It is usually brought on by exertion
- This is the way your heart saying that it is not getting enough oxygen
- It is particularly important to let your GP know if you are getting angina for the first time

## What should I do if I get angina?

- The first thing that you need to do is STOP what you are doing and rest
- If you are given GTN spray or tablets it is important to use this medications

## Remember the following

Angina pain/discomfort

Rest
1-2 GTN Spray/tablets under the tongue

If the pain is not relieved after 5 minutes
1-2 GTN Spray/tablets under the tongue

If the pain is not relieved after 5 minutes
1-2 GTN Spray/tablets under the tongue

If the pain is nor relieved after 5 minutes
Call 999 for an ambulance

## General advice on chest pain

- If you have GTN spray carry it with you at all time
- If you have access to mobile phone, it may be a good idea to carry with you
- If you know of a activity that you know bring on angina, you can take your GTN before the commencing that activity
- Seated while you take your GTN
- Do not stop taking your GTN because of your headache
- Do not be afraid of using your GTN spray

## How do I do Warm-Up?

Should be low level/
Nice and easy

10-15 minutes

Pulse raising activity and stretching

## Warming Up and Cooling Down

WHY WARM UP?

Prepare muscles for
activity - ↓ injury

Prepare heart for activity
- ↓ angina
- ↓ disturbances in heart rhythm

For peer review only
DRAFT

Imperial College London

## What sort of activity

- Aerobic, most beneficial activity for your heart
- Resistance or strength training

## Walking programme

| Stage of recovery | Length of walk (in minute) |
|---|---|
| (Approx. week 1) | 5 Minutes: several times per day Strolling/leisurely pace |
| (approx. week 2) | 10 minutes: twice a day, Leisurely pace |
| (approx. week 3) | 15 minutes: daily, Leisurely/moderate pace |
| (approx. week 4) | 20 minutes: daily, moderate pace |
| (approx. week 5) | 25 minutes: daily, brisk pace |
| (approx. week 6) | 30 minutes: daily brisk pace |
| Target | 30-45 minutes: daily brisk walk |

## Cool Down

WHY COOL DOWN?

↓ Fainting and dizziness

↓ Disturbances to your heart

↓ Muscle soreness

## How do I cool Down?

Goal is to return body to its resting state

Gradually slow down the activity you are doing and stretch

10 minutes

## Sensible Precautions

- Do not exercise if you feel unwell
- Do not exercise on a full or empty stomach
  - Light meal/snack 1½ - 2 hours before
- Do not exercise in extreme temperatures
- Wear suitable clothing
- Take your medications
- Good days and Bad days
- Enjoy!

*Have an active, healthy, happy life!*

Imperial College London

*Risk factors and making lifestyle changes*

## Risk Factors & Making Changes

Olivia Molloy
Cardiac Rehabilitation Sister

## Modifiable and Non Modifiable Risk Factors

| • Modifiable | • Non Modifiable |
|---|---|
| 1. Smoking<br>2. High Blood Pressure<br>3. High Cholesterol<br>4. Physical Inactivity<br>5. Being Overweight<br>6. Diabetes<br>7. Alcohol consumption<br>8. Impaired glucose regulation | 1. Family History of Heart Disease<br>2. Age<br>3. Ethnic Background |

## Smoking

FACT

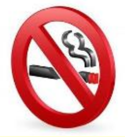

- Smoking is one of the major causes of cardiovascular disease. People who smoke are twice as likely to have a heart attack as to people who have never smoked

## Smoking

- Smoking damages the lining of the arteries leading to build up of Atheroma.
- Carbon Monoxide in cigarette smoke reduces the amount of oxygen that the blood can carry to the heart and around the body
- Nicotine stimulates adrenaline which increases heart rate and raises blood pressure = harder work load for your heart
- Second Hand Smoking…..

## Blood Pressure

- Blood Pressure represents the pressure of the blood in your arteries. We need a certain amount of pressure in our body in order to keep our blood ( circulation) flowing
  - High Blood Pressure is also known as

**“Hypertension”**

This is when your blood pressure is higher than the recommended level.

## Blood Pressure

- What does Systolic and Diastolic Pressure Mean?
- Readings:
  - 140/80mmhg = Non Diabetics
  - Diabetics = 130/80mmhg

**Maximum**

Imperial College London

## Blood Pressure

- Why is High Blood Pressure dangerous

Remember

- High Blood Pressure does not make you feel Sick.

## Cholesterol

- Cholesterol is a fatty substance which is found in the blood. Too much cholesterol is a contributing factor for Heart Disease.
- **LDL**
- **HDL**
- **Total Cholesterol**
- **Triglycerides**

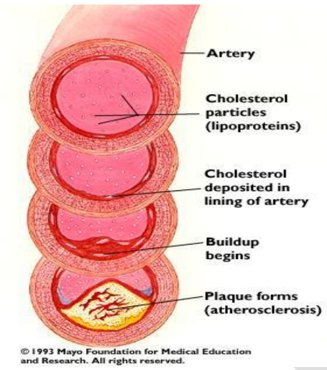


## Excercise

- In the UK people who are not physically active are twice as likely to have a heart attack
- In the UK; 7 out of 10 people do not do physical activity to benefit their health
- What can you do?

## Keeping a Healthy Weight & Shape

- Being overweight can increase your risk of developing Cardiovascular disease.
- Keeping close to a healthy weight will help you control your blood pressure and reduce the workload that you hear has to do.

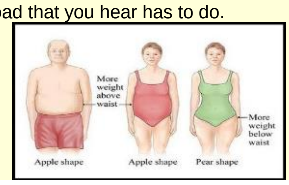


## Family History

- If you have a family history of CVD your own risk of developing the condition is increased.
- A family history means if your

father or brother >55

or

mother or sister >65

- Non Modifiable risk factor

## Eating Healthy For Your Heart

- Eat plenty of fruit and veg
- Choose healthier fats
- Eat oily fish regularly
- Reduce the amount of salt you eat

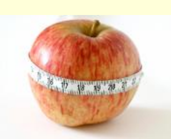

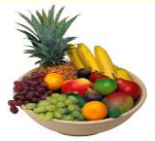

## Alcohol

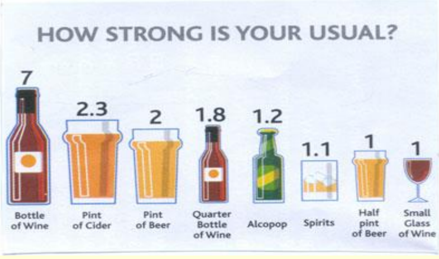




For peer review only - http://bmjopen.bmj.com/site/about/guidelines.xhtml

Imperial College London

## Stress

- Stress is not a direct risk factor for CVD but it is possible that stress may contribute to it, or perhaps bring on some symptons.
- The way you deal with stress can encourage unhealthy behaviours e.g. smoking unhealthy eating , alcohol etc

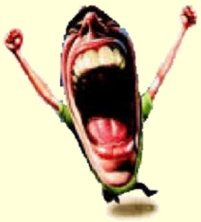

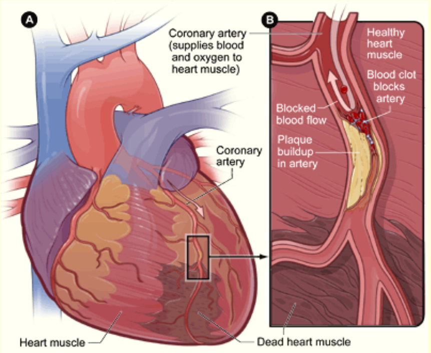


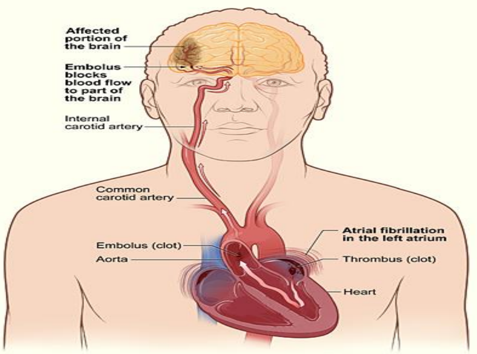


For peer review only
DRAFT

Imperial College London

*The heart and how it works*

## The Heart & How it works

Olivia Molloy
Cardiac Rehabilitation Nurse Specialist

## The Human Heart

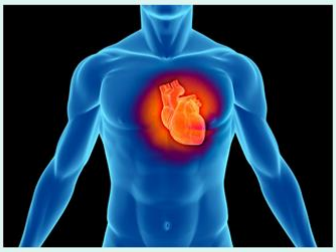

## The Heart

- The heart is a fist sized organ which lies within the chest behind the sternum (breast bone). The heart sits on the diaphragm, the main muscle of breathing, which is found beneath the lungs. The heart is considered to have two 'sides' – the right side and the left side.

  The heart has four chambers – an atria and ventricle on each side.

- The atria are both supplied by large blood vessels that bring blood to the heart (see below for more details).

- Atria have special valves that open into the ventricles. The ventricles also have valves but in this case they open into blood vessels. The walls of the heart chambers are made mainly of special heart muscle. The different sections of the heart have to contract (squeeze) in the correct order for the heart to pump blood efficiently with each heartbeat

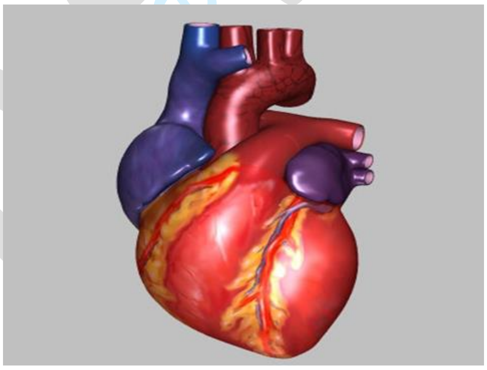

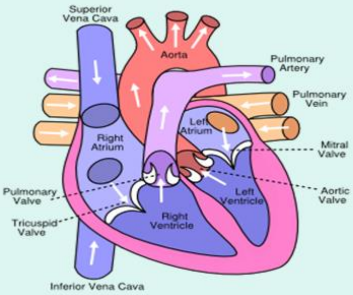


## The Function of the Heart

- The heart is a muscular pump that pushes blood through blood vessels around the body.

- Essential to life, the heart beats continuously, pumping the equivalent of more than 14,000 litres of blood every day.

  Blood vessels form the living system of tubes that carry blood both to and from the heart.

- All cells in the body need oxygen and the vital nutrients found in blood. Without oxygen and these nutrients, the cells will die.


For peer review only - http://bmjopen.bmj.com/site/about/guidelines.xhtml

Imperial College London

## The Heart Cont…

- The heart helps to provide oxygen and nutrients to the body's tissues and organs by ensuring a rich supply of blood.
- Not only do blood vessels carry oxygen and nutrients, but they also transport carbon dioxide and waste products away from our cells.
- Carbon dioxide is passed out of the body by the lungs, and most of the other waste products are disposed of by the kidneys.

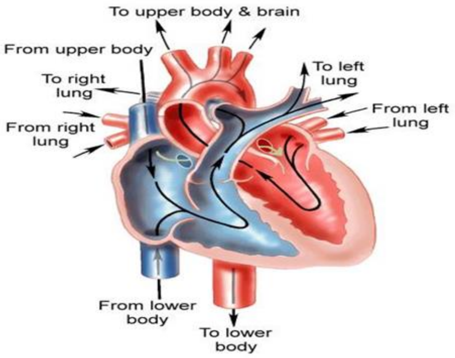


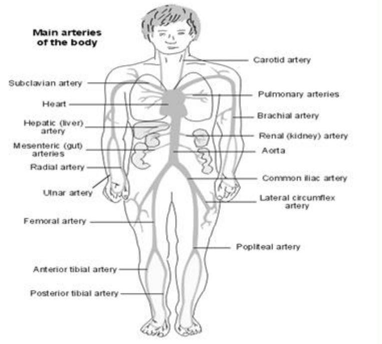


## The Blood Supply to the Heart

- Like any other muscle, the heart muscle needs a good blood supply. The coronary arteries take blood to the heart muscle. These are the first arteries to branch off the aorta - the large artery that takes blood to the body from the left ventricle.

  The right coronary artery mainly supplies the muscle of the right ventricle.

  The left coronary artery quickly splits into two and supplies the rest of the heart muscle.

  The main coronary arteries divide into many smaller branches to supply all the heart

## The coronary arteries of the heart

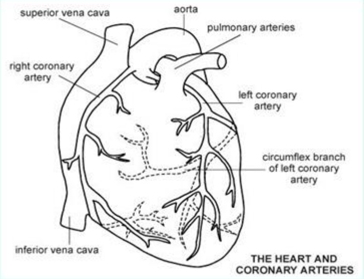


## Angioplasty

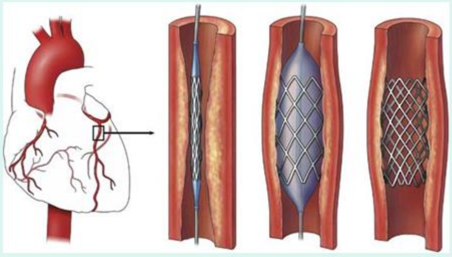

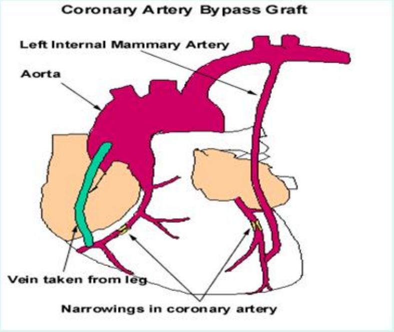


## The Heart Valves

The heart also contains four valves.

- Their role is to ensure the blood flows in a forward direction and prevents a backward flow during any part of the pump action (or cardiac cycle).
- An atrioventricular valve sits on both the left and right sides of the heart between each atrium and ventricle.
- These are the tricuspid valve (right side) and the mitral valve (left side).
- The two remaining valves sit on the outflow tract of the left and right ventricles.



For peer review only - http://bmjopen.bmj.com/site/about/guidelines.xhtml

Imperial College London

## Valves Cont….

- The pulmonary valve is between the right ventricle and the pulmonary artery, which takes deoxygenated blood to the lungs.
- The aortic valve sits between the left ventricle and the aorta, which takes oxygenated blood to the body's tissues.
- These latter two valves are semilunar; they contain three cusps which close to prevent the backward flow of blood from the outflow vessels during the diastolic (filling) phase of the cardiac cycle. The left side of the heart is inevitably under a much higher pressure than the right side, which delivers blood to the lungs only.

## Heart Valves

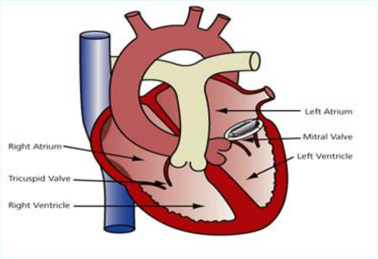


## Chest Pain

- Stop what your doing
- Sit Down and rest
- If you have GTN spray or tablets, use the spray and take your tablets as instructed by your doctor or cardiac rehab nurse

## Chest Pain Continued

- If you don't have GTN CALL 999 if pain does not go away.
- Aspirin , if you are not allergic to aspirin chew 300mgs until the ambulance arrives
- If the pain, discomfort or chest tightness continues especially if its gone on within 15 minutes
- DONT WAIT CALL 999 RIGHT AWAY

## CALL THE AMBULANCE AND STAY RESTING

## Basic Life Support

- Show DVD Recording here

## Blood Pressure / Pulse

- Non Diabetic Patients — 140/80 mmhg
- Diabetic Patients — 130/80 mmhg
- 
- Pulse for all — 60 – 100 bpm

## Diabetics

- All Diabetics Blood Sugar should be less than

6

For peer review only

Imperial College London

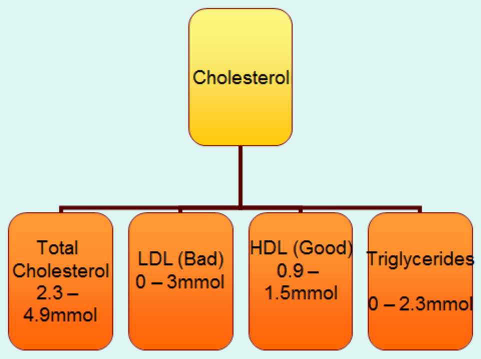


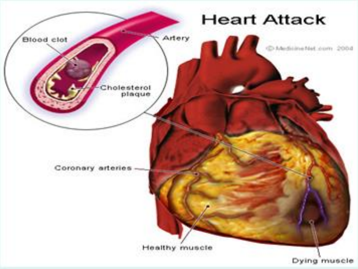


## Cont….

- Arterioles are the smallest arteries in the body. They deliver blood to capillaries. Arterioles are also capable of constricting or dilating and by doing this they control how much blood enters the capillaries.

  Capillaries are tiny vessels that connect arterioles to venules. They have very thin walls which allow nutrients from the blood to pass into the body tissues. Waste products from body tissues can also pass into the capillaries. For this reason capillaries are known as exchange vessels.

  Groups of capillaries within a tissue reunite to form small veins called venules. Venules collect blood from capillaries and drain into veins.

  Veins are the blood vessels that carry blood back to the heart. They may contain valves which stop blood flowing away from the heart.

## Cont….

- The right side of the heart receives deoxygenated blood (lacking oxygen) from the body. After passing through the right atrium and right ventricle this blood is pumped to the lungs. Here blood picks up oxygen and loses another gas called carbon dioxide. Once through the lungs, the blood flows back to the left atrium. It then passes into the left ventricle and gets pumped into the aorta, the main artery supplying the body. Oxygenated blood is then carried though blood vessels to all the body's tissues. Here oxygen and other nutrients pass into the cells where they are used to perform the body's essential functions.

  A blood vessels main function is to transport blood around the body. Blood vessels also play a role in controlling your blood pressure.

  Blood vessels are found throughout the body. There are five main types of blood vessels: arteries, arterioles, capillaries, venules and veins.

  Arteries carry blood away from the heart to other organs. They can vary in size. The largest arteries have special elastic fibres in their walls. This helps to complement the work of the heart, by squeezing blood along when heart muscle relaxes. Arteries also respond to signals from our nervous system, either constricting (tightening) or dilating (relaxing).

## Blood Flow Through the Body

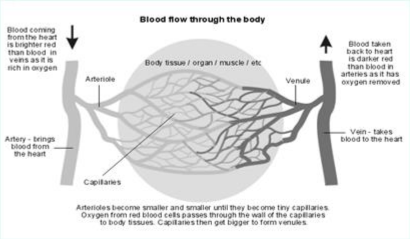




For peer review only - http://bmjopen.bmj.com/site/about/guidelines.xhtml

Imperial College London

# References

1. Sivananda: Yoga Teachers' Training Manual

2. Sivananda Yoga Vedanta Centre: Yoga-Your Home Practice Companion

3. Sivananda Yoga Vedanta centre: The Yoga Cookbook:Vegetarian Food for Body and Mind

4. H.David Coulter: Anatomy of Hatha Yoga

5. Swami Vishnu-Devananda: The Complete Illustrated Book of Yoga

6. www.yoga.about.com

7. www.innerhealthstudio.com

For peer review only

# CONSORT 2010 checklist of information to include when reporting a randomised trial*

| Section/Topic | Item No | Checklist item | Reported on page No |
|---|---|---|---|
| **Title and abstract** | | | |
| | 1a | Identification as a randomised trial in the title | 1 |
| | 1b | Structured summary of trial design, methods, results, and conclusions (for specific guidance see CONSORT for abstracts) | 2 |
| **Introduction** | | | |
| Background and objectives | 2a | Scientific background and explanation of rationale | 3 |
| | 2b | Specific objectives or hypotheses | 3 |
| **Methods** | | | |
| Trial design | 3a | Description of trial design (such as parallel, factorial) including allocation ratio | 4 |
| | 3b | Important changes to methods after trial commencement (such as eligibility criteria), with reasons | 4 |
| Participants | 4a | Eligibility criteria for participants | 4 |
| | 4b | Settings and locations where the data were collected | 8 |
| Interventions | 5 | The interventions for each group with sufficient details to allow replication, including how and when they were actually administered | 4, and supplementary files, 'Yacht Study package_v1_2, which includes 'Policy for Rehabilitation in Ealing' |
| Outcomes | 6a | Completely defined pre-specified primary and secondary outcome measures, including how and when they were assessed | 5-7 |
| | 6b | Any changes to trial outcomes after the trial commenced, with reasons | N/A |
| Sample size | 7a | How sample size was determined | 8 |
| | 7b | When applicable, explanation of any interim analyses and stopping guidelines | N/A |


For peer review only - http://bmjopen.bmj.com/site/about/guidelines.xhtml

| | | | |
|---|---|---|---|
| Randomisation: | | | |
| Sequence generation | 8a | Method used to generate the random allocation sequence | 4 |
| | 8b | Type of randomisation; details of any restriction (such as blocking and block size) | 4 |
| Allocation concealment mechanism | 9 | Mechanism used to implement the random allocation sequence (such as sequentially numbered containers), describing any steps taken to conceal the sequence until interventions were assigned | 4 |
| Implementation | 10 | Who generated the random allocation sequence, who enrolled participants, and who assigned participants to interventions | 4 |
| Blinding | 11a | If done, who was blinded after assignment to interventions (for example, participants, care providers, those assessing outcomes) and how | 7-8 |
| | 11b | If relevant, description of the similarity of interventions | N/A |
| Statistical methods | 12a | Statistical methods used to compare groups for primary and secondary outcomes | 8-9 |
| | 12b | Methods for additional analyses, such as subgroup analyses and adjusted analyses | 8-9 |
| **Results** | | | |
| Participant flow (a diagram is strongly recommended) | 13a | For each group, the numbers of participants who were randomly assigned, received intended treatment, and were analysed for the primary outcome | Figure 1_consort flow diagram_YACHT |
| | 13b | For each group, losses and exclusions after randomisation, together with reasons | Figure 1_consort flow diagram_YACHT |
| Recruitment | 14a | Dates defining the periods of recruitment and follow-up | 4 |
| | 14b | Why the trial ended or was stopped | 4 |
| Baseline data | 15 | A table showing baseline demographic and clinical characteristics for each group | Table 1, Supplemental tables |
| Numbers analysed | 16 | For each group, number of participants (denominator) included in each analysis and whether the analysis was by original assigned groups | Figure 1_consort flow |


For peer review only - http://bmjopen.bmj.com/site/about/guidelines.xhtml

| | | | |
|---|---|---|---|
| | | | diagram_YACHT. Tables 2-5 |
| Outcomes and estimation | 17a | For each primary and secondary outcome, results for each group, and the estimated effect size and its precision (such as 95% confidence interval) | Tables 2-5 |
| | 17b | For binary outcomes, presentation of both absolute and relative effect sizes is recommended | N/A |
| Ancillary analyses | 18 | Results of any other analyses performed, including subgroup analyses and adjusted analyses, distinguishing pre-specified from exploratory | N/A |
| Harms | 19 | All important harms or unintended effects in each group (for specific guidance see CONSORT for harms) | 9 |
| **Discussion** | | | |
| Limitations | 20 | Trial limitations, addressing sources of potential bias, imprecision, and, if relevant, multiplicity of analyses | 13 |
| Generalisability | 21 | Generalisability (external validity, applicability) of the trial findings | 13 |
| Interpretation | 22 | Interpretation consistent with results, balancing benefits and harms, and considering other relevant evidence | 13 |
| **Other information** | | | |
| Registration | 23 | Registration number and name of trial registry | 1 |
| Protocol | 24 | Where the full trial protocol can be accessed, if available | Available on request/file uploaded |
| Funding | 25 | Sources of funding and other support (such as supply of drugs), role of funders | 13 |

*We strongly recommend reading this statement in conjunction with the CONSORT 2010 Explanation and Elaboration for important clarifications on all the items. If relevant, we also recommend reading CONSORT extensions for cluster randomised trials, non-inferiority and equivalence trials, non-pharmacological treatments, herbal interventions, and pragmatic trials. Additional extensions are forthcoming: for those and for up to date references relevant to this checklist, see www.consort-statement.org.

For peer review only

BMJ Open

# Yoga and Cardiovascular Health Trial (YACHT): a UK-based randomised mechanistic study of a yoga intervention plus usual care vs usual care alone following an acute coronary event



| | |
|---|---|
| Journal: | *BMJ Open* |
| Manuscript ID | bmjopen-2019-030119.R2 |
| Article Type: | Original research |
| Date Submitted by the Author: | 08-Oct-2019 |



| | |
|---|---|
| Complete List of Authors: | Tillin, Therese; University College London, Institute of Cardiovascular Science<br>Tuson, Claire; York Hospitals NHS Trust<br>Sowa, Barbara; West London Mental Health NHS Trust<br>Chattopadhyay, Kaushik; University of Nottingham School of Health Sciences; London School of Hygiene and Tropical Medicine<br>Sattar, Naveed; University of Glasgow<br>Welsh, Paul; University of Glasgow<br>Roberts, Ian; London School of Hygiene and Tropical Medicine<br>Ebrahim, Shah; London School of Hygiene and Tropical Medicine<br>Kinra, Sanjay; London School of Hygiene and Tropical Medicine<br>Hughes, A; University College London, Institute of Cardiovascular Science<br>Chaturvedi, Nishi; University College London, Institute of Cardiovascular Science |



| | |
|---|---|
| <b>Primary Subject Heading</b>: | Cardiovascular medicine |
| Secondary Subject Heading: | Rehabilitation medicine |
| Keywords: | Yoga, Cardiac rehabilitation, diastolic function, exercise, blood pressure, heart rate |
| | |


SCHOLARONE™
Manuscripts

# Yoga and Cardiovascular Health Trial (YACHT): a UK-based randomised mechanistic study of a yoga intervention plus usual care vs usual care alone following an acute coronary event

Authors

Therese Tillin, Institute of Cardiovascular Science, UCL, corresponding author: email: t.tillin@ucl.ac.uk

Claire Tuson, York Teaching Hospital NHS Foundation Trust

Barbara Sowa, West London NHS Trust

Kaushik Chattopadhyay, Division of Epidemiology and Public Health, University of Nottingham

Naveed Sattar, Institute of Cardiovascular & Medical Sciences, University of Glasgow

Paul Welsh, Institute of Cardiovascular & Medical Sciences, University of Glasgow

Ian Roberts, London School of Hygiene and Tropical Medicine

Shah Ebrahim, London School of Hygiene and Tropical Medicine

Sanjay Kinra, London School of Hygiene and Tropical Medicine

Alun Hughes, Institute of Cardiovascular Science, UCL

Nishi Chaturvedi, Institute of Cardiovascular Science, UCL

Word count: abstract: 294 words, main text: 5717 words



For peer review only - http://bmjopen.bmj.com/site/about/guidelines.xhtml

## Abstract

**Objective:** To determine effects of yoga practice on subclinical cardiovascular measures, risk factors and neuro-endocrine pathways in patients undergoing cardiac rehabilitation following acute coronary events.

**Design:** 3-month, two arm (yoga+usual care vs usual care alone) parallel randomised mechanistic study.

**Setting:** One general hospital and two primary care cardiac rehabilitation centres in London. Assessments were conducted at Imperial College London.

**Participants:** 80 participants, aged 35-80 years (68% male, 60% South Asian) referred to cardiac rehabilitation programmes 2012- 2014.

**Intervention:** A certified yoga teacher conducted yoga classes which included exercises in stretching, breathing, healing imagery and deep relaxation. It was pre-specified that at least 18 yoga classes were attended for inclusion in analysis. Participants and partners in both groups were invited to attend weekly a 6-12 week local standard NHS cardiac rehabilitation programme.

**Main outcome measures**: i) estimated left ventricular filling pressure (E/e'), ii) distance walked, fatigue and breathlessness in a 6-minute walk test (6MWT), iii) BP, heart rate and estimated peak $VO_2$ following a three-minute step-test. Effects on the hypothalamus-pituitary-adrenal axis, autonomic function, body fat, blood lipids and glucose, stress and general health were also explored.

**Results** 25 participants in the yoga+usual care group and 35 participants in the usual care group completed the study. Following the 3-month intervention period, E/e' was not improved by yoga (E/e': between group difference: yoga minus usual care:-0.40(-1.40, 0.61) Exercise testing and secondary outcomes also showed no benefits of yoga.

**Conclusions** In this small UK-based randomised mechanistic study, with 60 completing participants (of whom 25 were in the yoga+usual care group), we found no discernible improvement associated with the addition of a structured 3 month yoga intervention to usual cardiac rehabilitation care in key cardiovascular and neuroendocrine measures shown to be responsive to yoga in previous mechanistic studies


Keywords: Yoga, cardiac rehabilitation, exercise, blood pressure, heart rate

Trial registration https://clinicaltrials.gov/ct2/show/record/NCT01597960

For peer review only

## Article Summary

### Strengths and Limitations of this study

- Comprehensive clinical and subclinical cardiovascular measures before and after a yoga intervention (plus usual cardiac rehabilitation) vs usual cardiac rehabilitation
- Real world setting – older people following an acute coronary event
- High level of dropout, particularly in the yoga plus usual cardiac rehabilitation arm
- We can only assess the potential of yoga in addition to usual cardiac rehabilitation following an acute coronary event.

## Introduction

The practice of yoga originated in ancient India as a form of exercise which includes breath control, the adoption of bodily postures and meditation which aim to increase strength and flexibility and to aid physical and mental wellbeing.(1) Yoga has been reported to reduce stress and depression and is thought to improve biological cardiovascular risk factors.(2-4) However, despite claims of benefits, the effects of yoga on cardiovascular outcomes remain unclear. Previous systematic reviews (5-12) confirm that investigations of the health benefits of yoga and underlying mechanisms have often been hampered by poor study design, including small sample sizes, inadequate adjustment for confounders, lack of randomisation, unsatisfactory masking of outcomes to assessors, and publication bias. Also, many studies have been conducted in healthy young participants and it is not certain that these findings are generalisable to older adults with established disease.

Cardiac rehabilitation (CR) has been shown to improve cardiovascular mortality and hospital re-admissions in patients with coronary heart disease.(13) However, for myocardial infarction (MI), coronary bypass grafts (CABG) and percutaneous coronary intervention (PCI) patients uptake to CR across in UK was only ~45% in 2012-3 with low representation of ethnic minority people.(14) Yoga could therefore be a useful adjunct to CR.

In this UK-based randomised study (Yoga and Cardiovascular Health Trial (YACHT)), we hypothesised that yoga would be associated primarily with improvements in cardiovascular function and exercise capacity both chronically and acutely in people eligible for CR. The chronic study compared cardiovascular measures at 3 months between two groups randomised either to usual care (including CR) or to usual care plus a programme of yoga classes. For the chronic study, where the emphasis was on rehabilitation following a coronary event, we focussed on the ratio between early mitral inflow and mitral annular early diastolic velocity (E/e') as the primary cardiac measure. E/e' provides an estimate of left ventricular (LV) filling pressure, (15) an aspect of LV diastolic function that predicts survival after myocardial infarction.(16) We also performed a 6-minute walk test (6MWT) as a measure of exercise tolerance and a three-minute step-test as a measure of cardiopulmonary fitness. These measures were chosen as they are reproducible and safe tests which are improved by CR,(17-20) and predict outcomes in people with coronary heart disease.(16, 21-23) Measures chosen for the acute study (before and after the first session of yoga) included blood pressure (BP) and heart rate before and after exercise as indicators of cardiovascular and autonomic function which are associated with cardiovascular outcomes. (21, 23)

In addition to these primary outcome measures, we studied a range of other cardiovascular risk factors and measures which might be expected to improve following CR and provide mechanistic insight into any beneficial effect of yoga; these included markers of the hypothalamic–pituitary axis, measures of autonomic function, measures of cardiac structure and function, brachial and central resting and 24 hour ambulatory



For peer review only - http://bmjopen.bmj.com/site/about/guidelines.xhtml

blood pressure, markers of atherosclerosis, blood glucose and lipids and self-reported health, lifestyle factors and perceived stress levels.

For peer review only



For peer review only - http://bmjopen.bmj.com/site/about/guidelines.xhtml

**Methods**

Study population

Inclusion criteria included referral to CR programmes in north-west London following an acute coronary syndrome (myocardial infarction, percutaneous coronary intervention, coronary artery bypass grafting). Pre-specified inclusion criteria were age between 35 and 80 years, male or female, without co-morbid disease or mobility limitations that would preclude participation in CR and our investigations, and, given the north-west London area of recruitment, able to understand English or Punjabi. Ethnicity was self-defined, and verified by country of birth of all 4 grandparents. 80 participants were recruited following discharge from hospital and randomised in equal numbers to the yoga intervention plus their standard CR programme, or to standard CR programme (usual care) alone. Randomisation was performed by an independent researcher using a standard computerized algorithm (customised Java web application (srub)) and stratified by ethnicity (South Asian and non-South Asian), gender, 5 year age group and rehabilitation centre. The generated sequence was displayed only to the user at the time of assignment to the yoga intervention or usual care. 75% of participants were recruited from referrals to CR programmes at Ealing Hospital in west London, with the remainder recruited from two primary care CR programmes in north-west London (Harrow and Brent (Flexi-Heart Plan)). Recruitment of the planned 80 participants took place between October 2012 and April 2014, with the final participant seen for 3-month follow-up measures in July 2014.

Eligibility criteria were broadened in January 2013 and April 2013 respectively, with ethical approval, to include patients who had undergone coronary artery bypass grafting or who had received medical management only for their acute coronary event. The initial study plans were to recruit only patients referred to a CR programme post-angioplasty as treatment for an acute coronary syndrome. With cardiologist advice, it was felt that the earlier decision to exclude these patients based on safety grounds was unnecessary given the gentle and tailored nature of the exercises.

Patient and Public Involvement

Patients and public were not involved in the study design, conduct, results, evaluation or dissemination.

Ethical approval for the study was granted by Camberwell St Giles Research Ethics Committee (Ref: 12/LO/0597). Informed written consent was obtained from all participants.

Yoga intervention

The yoga intervention was delivered on a twice-weekly group session basis for 12 weeks alongside the usual care, 6-12 week CR programme. There were 24 yoga classes in total. Participants' partners were invited to take part in each session as a method of improving adherence. The yoga session was designed and conducted by a teacher certified in yoga and CR, and included gentle exercises in deep relaxation, stretching, breathing, healing imagery and a healthy diet. A prescription of exercises with an accompanying DVD was provided to be performed regularly at home. Each session lasted approximately 75 minutes, divided into three equal parts: breathing exercises, yogic poses and meditations, education and discussion (details in supplemental material: YACHT study package_v1.2.pdf ). Individuals randomised to the yoga arm had their standard CR care delivered at a separate time to those randomised to usual CR, (although delivered by the same teams), to reduce risks of contamination. Because the study was also



For peer review only - http://bmjopen.bmj.com/site/about/guidelines.xhtml

designed to examine mechanisms underlying any beneficial effects of yoga (24), there was a pre-specified requirement for participants in the yoga + usual care group to complete at least 18 yoga classes.

Usual care

Usual care is described in the supplemental material (YACHT study package_v1.2.pdf) and was similar in all centres in accordance with the UK's National Institute for Health and Care Excellence (NICE) guidelines (https://www.nice.org.uk/guidance/cg48, accessed 25/8/2017) and British Association for Cardiac Prevention and Rehabilitation (BACR) standards (25) with core components of lifestyle (physical activity, exercise, diet and weight management, smoking cessation), education, risk factor management, psychosocial, cardio-protective drug therapy and long-term management strategies. Patients and their partners were invited to attend once-weekly for a 6-12 week programme tailored to individual needs and including 1) on-going risk factor monitoring/advice/support, 2) exercise sessions in a gym, led by cardiac physiologist or a home-based exercise programme, 3) health education lectures (led by CR sister, pharmacist, dietician, clinical psychologist, cardiac physiologist), 4) relaxation sessions, 5) guidance and supervised use of the "Edinburgh Heart Manual" (http://www.theheartmanual.com/Pages/default.aspx, accessed 26/9/2017 ).

Outcome measures

Chronic study: All measures performed pre-intervention and 3 months post-intervention

Primary outcome measures

Echocardiography

Transthoracic two-dimensional (2D) and Doppler echocardiography was performed as previously described (26). Transmitral flow velocity during the early filling phase (E) was acquired by pulsed Doppler and averaged from three consecutive cycles. Tissue Doppler Imaging was performed on the lateral and septal LV wall. Peak velocities during early diastole (e') were averaged from three consecutive representative cycles. The e' wave velocities measured from the lateral and septal walls were averaged. The primary cardiac outcome was the ratio of early filling and early myocardial velocity (E/e'), a non-invasive estimate of LV filling pressure.(15)

Exercise capacity and physical fitness

Exercise capacity was measured by a 6-minute walk test conducted along a 30 m straight path in an outdoor covered area marked clearly with the beginning and end of each lap. Participants wore appropriate shoes and loose-fitting clothing and rested in a chair for 10 minutes before the start of the test. Participants were asked to walk briskly as far as possible for a timed 6 minutes. Fatigue and dyspnoea before and after the walk test were assessed using the Borg scale (27).

Physical fitness was measured using a Tecumseh step-test (28) Participants were asked to step repeatedly on and off a step measuring 60x30x17.5 cm (length, width, height) for three minutes in time with a metronome set to 92 beats per minute (bpm). This corresponds to a rate of energy expenditure approximately 5 times the basal metabolic rate. (29) Heart rate was measured on the right arm immediately afterwards and then again in the seated position after three minutes recovery using an Omron 705CP device. Estimated peak oxygen consumption (peak $VO_2$) was calculated based on achieved heart rate in the immediate post-exercise period as described previously.(28)



For peer review only - http://bmjopen.bmj.com/site/about/guidelines.xhtml

Chronic study: Secondary outcome measures

Measures of cardiac structure and function were obtained as described under primary outcomes above and included left ventricular mass index, relative wall thickness, left atrial diameter, ejection fraction, mitral E/A ratio, s'(peak velocity during systole) and e'(peak velocity during early diastole).

Resting seated brachial and central blood pressure was measured after 5 minutes seated rest using a Pulsecor BP+ device (Uscom Ltd, Sydney, Australia) (30) starting with the left arm and then repeated on the right arm. The average of the final 2 of 3 blood pressure readings for the right arm were used, unless the average SBP was more than 10 mm Hg greater than the average in the left arm, in which case the left arm average readings were used as the measure of clinic BP. BP (standing) before, immediately after and after 3 minutes recovery following the step-test was measured using an Omron 705CP device on the right arm.

A Vicorder oscillometric device (SMT Medical Germany/Skidmore Medical UK) (31) was used to measure carotid-femoral pulse wave velocity (PWV).

Ambulatory blood pressure monitoring was conducted using the oscillometric Mobilograph device (NuMed Healthcare, UK) (32) with an appropriately sized cuff worn on the non-dominant arm to record central blood pressure and heart rate for a 24 hour period; measurements were taken half-hourly between 0700 and 2100 hours and hourly during the night. Ambulatory blood pressure and heart rate analyses included the daytime period from 0900-2100 hours and the night-time period from 0100-0600 excluding the waking and bedtime periods of the day as these periods represent times during which bed rest is inconsistent and, therefore, cannot be categorised reliably.(33)

Heart rate variability (HRV) and baroreceptor sensitivity (BRS) were measured according to a published protocol (34). Briefly, these were measured in the recumbent position for a 10-minute period. Beat to beat arterial BP was recorded non-invasively using a Finometer (FMS Amsterdam, Netherlands), and the ECG was monitored using a 3 lead ECG. Signals were post processed as described in detail previously (34). For HRV we calculated the mean R-R interval, and mean spectral powers in the low frequency (LF: 0.04-0.15 Hz) and high frequency (HF: 0.15-0.4 Hz) bands for the R-R intervals. Frequency domain BRS was calculated as the alpha index given by the square root of the ratio between averaged powers of R-R and systolic BP for each frequency.

Fasting bloods were analysed for glucose and lipids at baseline and 3-month follow-up. The HPA axis was assessed by salivary cortisol sampled at 5 points during the day pre-intervention and at 3 months follow-up as described for the acute study below. Salivary amylase, as a marker of sympathetic activity, was measured at 5 time points during the day, as described for cortisol.

The full extra-cranial carotid artery was examined for the presence of plaque using an iE33 ultrasound machine (Philips) equipped with a linear-array transducer (L11_3) with concurrent recording of 3-lead ECG over 3-5 cardiac cycles. Carotid intima-media thickness (IMT) was measured in the distal 1 cm of the left common carotid artery from three longitudinal planes (anterior, lateral and posterior) in a region free of plaque with a clearly identified double-line pattern. Plaque was defined according to the Mannheim consensus as a focal structure encroaching into the arterial lumen by at least 0.5 mm or 50% of the surrounding IMT value, or a region of IMT having a thickness >1.5 mm. Analyses were performed using a validated semi-automated programme (AMS-II).



For peer review only - http://bmjopen.bmj.com/site/about/guidelines.xhtml

The GeneActiv wrist-worn waterproof accelerometry device was fitted at the end of the pre-intervention and 3-month follow-up visits and worn for 3 days after each visit. Analysis of the data was performed using a validated algorithm at the University of Newcastle (35) to provide average body acceleration (metric milli g where g is gravity) on days with more than 16 hours of valid readings.

Self-completion questionnaires were administered pre-interventions and at the 3-month follow-up as follows:

The international physical activity questionnaire (IPAQ) long version was administered and analysed according to the IPAQ guidelines (http://www.ipaq.ki.se/scoring.pdf, accessed Aug 25th 2017)

A self-completion questionnaire included items regarding frequency of alcohol consumption, number of units consumed and changes in drinking habits. Similar questions were included regarding smoking habits. A food frequency questionnaire, previously used in the SABRE tri-ethnic cohort study(36) covered the previous 7 days.

EQ-5D™ (https://euroqol.org/) is a standardised instrument for use as a measure of health outcome. It provides a simple descriptive profile a visual analogue scale to indicate self-rated health and a health status score based on UK population norms (there is no set of scores based on Indian Asian populations).

The perceived stress 10 item self-completion scale (37) was completed together with questions regarding sleep quality, snoring and breathlessness at night.

Acute study: Yoga+usual care group on day of first yoga session

Primary outcome measures - blood pressure and heart rate at rest and following a 3-minute step-test and estimated peak oxygen consumption (peak $VO_2$) measured immediately post-exercise

Seated brachial blood pressure was measured after 5 minutes rest using an Omron 705CP device on the right arm. Blood pressure, heart rate at rest and following the 3-minute step-test were performed immediately before and after the first yoga session as described above for the chronic study; estimated peak $V0_2$ was also calculated. (28, 29)

Secondary outcome measures

Saliva samples for amylase and cortisol were collected by the participants at home using a Salivette (www.salimetrics.com) collection kit at 5 time points during the day pre-intervention (waking, waking plus 30 minutes, waking plus 90 minutes, waking plus 12 hours, bedtime). For the acute study, waking, waking plus 12 hours and bedtime samples were taken on the day of the first yoga session. The latter two sampling points therefore occurred after the first yoga session. Samples were analysed using using indirect enzyme-linked immunosorbent assay kits (Salimetrics Europe Ltd., Suffolk, UK).

Blinding of observers

Post-processing of echocardiograms, carotid ultrasound scans, accelerometry, ambulatory blood pressure, heart rate variability and baroreceptor sensitivity, blood and saliva analyses were all conducted by observers blinded to participants' identity and study group. Clinic BP, vascular measurements and



For peer review only - http://bmjopen.bmj.com/site/about/guidelines.xhtml

anthropometric measurements were conducted by clinic staff, who may have been aware of study group allocation, given the nature of the interventions.

Location where data were collected

Data were collected at the International Centre for Circulatory Health on the St Mary's campus of Imperial College London (UK).

Statistical analyses

Sample size and power

The sample size estimate was based on primary outcome measures for the chronic effects of yoga, i.e. E/e' echocardiography, 6-minute walk test and Tecumseh 3 minute step test. Previous studies have reported at least half a standard deviation (SD) benefit associated with yoga on both diastolic function and exercise testing (38, 39) corresponding to a 1.1 improvement in E/e'(38, 39), and a study in people with preserved ejection fraction heart failure reported more than double this effect (-3.2) following a 3 month exercise programme.(20) For exercise testing (the 6-minute walk test), a distance of 40m (equivalent to 0.5 SD benefit) was considered a clinically significant improvement in distance walked (40). This improvement was exceeded in a study of CR, where the distance walked increased by 62m. (41) In both cases these minimum important differences corresponded to approximately 0.5 SD . The sample size was estimated to detect effects of this magnitude for the three primary outcomes. Statistical analyses were planned to use regression modelling to adjust final measures for baseline differences, thus improving the precision of estimates of treatment effect, and shrinking the sample size requirement.(42) The intraclass correlation coefficient (ICC) of the primary outcomes was ≥0.85 based on our own data (n = 10) and other observers'.(19) Using a conservative estimate of ICC = 0.70, and allowing for multivariable analysis, 33 completers were required in each arm of the study to detect a 0.5 standard deviation difference between groups (80% power and 5% significance). Thus, 40 people were recruited to each arm to allow for dropouts.

Statistical methods

Chronic study: summary descriptions of continuous pre-intervention characteristics are shown as means (95% CI) for Normally distributed data or as medians (95%CI of the median(CIM)) for non-Normally distributed variables or as number (%) for categorical variables. Pre-intervention characteristics are shown for the whole study group (Table 1) and for those who did and did not complete the study. (Table S1). Outcome analysis is restricted to those who attended the 3-month visit, and for the yoga group, additionally restricted to those who attended 18 out of the 24 yoga sessions, per protocol. A sensitivity analysis added 4 participants who did not complete the requisite number of yoga classes but who attended the 3-month study follow-up visit.

For the 3-minute step-test which was conducted in three stages pre- and post-intervention, repeated measures ANOVA models were used to determine differences by intervention arm and timing (pre-intervention and 3 months follow-up for the chronic study) and for the acute study (pre- and post-first yoga session). Repeated measures ANOVA models were also used for salivary amylase (log transformed) and cortisol measured 5 times on 3 days (yoga + usual care group) or 2 days (usual care group).

The remaining measures were analysed using robust regression models, (43) which are relatively efficient in the presence of outlier-prone error distributions. 3-month follow-up values were adjusted for the pre-



For peer review only - http://bmjopen.bmj.com/site/about/guidelines.xhtml

intervention value of each Normally distributed measure, to provide adjusted mean (95%CI) values to allow comparison with pre-intervention observations. Where data were not Normally distributed pre-intervention, median regression provided comparable 3-month (median (95% CI of the median)) follow-up values adjusted for the pre-intervention value. We show between group differences (95% CI) and p values for all outcome measures. Sensitivity analyses for primary outcomes included adjustment for informative baseline covariates (age, sex, diabetes, body mass index plus height for the 6 minute walk test). Between- and within-group differences in categorical secondary outcome measures were tabulated and tested using the chi square test.

For heart rate variability and baroreceptor sensitivity, we conducted sensitivity analyses that excluded the few participants who were not receiving beta-blocker medication.

Statistical analyses were performed using STATA version 15 software.

**Results**

80 participants were recruited and randomly assigned in equal numbers to the yoga plus usual care and usual care groups. Pre-intervention, average age was 57.1 (95% CI: 54.9, 59.4), 68% were male and 64% were of South Asian origin. Diabetes was present in 36%. The majority were receiving statins (90%) and/or anti-hypertensive medication (95%). (Table 1) Consistent with current practice in the UK, 91% had received percutaneous coronary interventions.

Thirty-five participants in the usual care arm (63% South Asian) and 25 participants in the yoga arm (59% South Asian) completed the study. Greater loss to follow-up occurred in the yoga group, mostly due to unwillingness to continue with yoga classes - participants frequently citing ill health as a reason, although one participant withdrew from the study because of return to work. (Figure S1) Characteristics of those who completed the study and those who dropped out were similar pre-intervention. (Table S1) In addition to overall study dropout, several participants declined or were unable to undergo exercise testing either pre-or post-intervention, mostly due to mobility problems or elevated blood pressure (reasons are listed under Table 2).

No adverse events were reported. There was minimal change in the number and type of medications prescribed over the 3-month course of the study. (Tables 1 and S1)

<u>Chronic study</u>

<u>Primary outcomes</u>

<u>Left ventricular diastolic function</u>

At the three month follow-up, E/e' improved in both groups, but there was no evidence of yoga-related additional benefit in diastolic function (E/e': between group difference: yoga minus usual care:-0.40(-1.40, 0.61) (adjusted for pre-intervention values) p=0.4 (Table 2)

<u>6-minute walk test</u>

The total distance walked increased in both groups at 3-months follow-up, but there was no evidence of yoga-related additional benefit (between group difference yoga minus usual care: -7 (-40, 25) m, p = 0.7; Table 2). Distance walked per minute also increased post-intervention to a similar level in both groups and



For peer review only - http://bmjopen.bmj.com/site/about/guidelines.xhtml

For peer review only

there was no additional advantage related to yoga in the total number of minutes walked or in levels of fatigue and breathlessness. (Table 2)

3-minute step-test

The results of the 3 minute step-test at 3 month follow-up suggested some moderate improvements in immediate post-exercise BP, heart rate and peak $VO_2$ in both groups at follow-up, but there was no evidence of additional benefit associated with yoga. (Table 2)

Secondary outcomes

Other vascular measures

There was no evidence of yoga-related additional benefits for measures of clinic and ambulatory measures of brachial and central SBP at follow-up. Both groups showed improvements in resting brachial DBP and in resting central SBP. Pulse wave velocity was similar in the two groups at follow-up. (Table 3)

Carotid intima-media thickness

There was no evidence of additional yoga-related benefit on carotid IMT levels at 3 months. (Table 3)

Hypothalamic-Pituitary-Adrenal axis (HPA)

Salivary cortisol, as a marker of the HPA, decreased throughout the day in both groups pre-intervention and at 3 months follow-up. There was no evidence of additional yoga-related benefit compared with usual care alone. (Table 3)

Autonomic function

There was no evidence of additional yoga-related benefit compared with usual care alone on markers of heart rate variability baroreceptor sensitivity at 3 month follow-up and salivary amylase. (Table 4)

Metabolic measures

There was no evidence of additional yoga-related benefit compared with usual care alone at 3 months follow-up in glucose, total cholesterol, LDL cholesterol. (Table 3)

Anthropometrics

Both groups had slightly lower waist to hip ratios at follow-up than at baseline, but with no evidence of yoga-related additional benefit compared with usual care alone. (Table 3)

Other measures

Accelerometry over 3 days showed that the usual care group modestly increased levels and the yoga group maintained levels of physical activity during the follow-up period. Self-reported physical activity (IPAQ) increased in both groups, with no evidence of additional benefit from yoga compared with usual care alone. (Table 3)

Similarly the EQ5D measures of health status or self-rated health at follow-up did not show any convincing evidence of a treatment effect at follow-up, although there was a small increase in EQ5D health status based on UK population norms in people randomized to yoga compared with those receiving usual care.



For peer review only - http://bmjopen.bmj.com/site/about/guidelines.xhtml

For peer review only

The EQ5D self-rated health thermometer improved to equal extents in both yoga and usual care groups over the 3 month period. The yoga group had lower stress scores than the usual care group both pre-intervention and at follow-up and there was no convincing evidence of change in stress score in either treatment group. (Table 3)

There were very few current smokers at baseline or follow-up and there were no between- group differences or within group changes. There were no between-group differences or significant within-group changes at follow-up in self-reported hours and quality of sleep, in alcohol consumption or in consumption of fresh fruit and vegetables (not shown).

Sensitivity analyses

Sensitivity analyses of the primary outcomes which added those 4 participants who did not complete 18 yoga classes, but who did attend the 3 month follow-up clinic, did not alter findings. Likewise, exclusion of the few people who were not receiving beta blocker medication did not alter the findings for heart rate variability, baroreceptor sensitivity and salivary amylase. Additional adjustment of primary outcome measures for selected informative baseline covariates (age, sex, diabetes, body mass index (and height for the 6 minute walk test) did not alter conclusions, e.g. adjusted between group difference in E/e' was -0.18 (-1.28, 0.92) compared with -0.38(-1.38, 0.58) when adjusted only for baseline E/e'. The three minute step test and six minute walk test findings were little changed on adjustment for these baseline covariates

Acute study (yoga arm only)

A 3-minute step-test was performed before and after the first yoga session and blood pressure was measured pre-exercise, immediately post exercise and 3 minutes post-exercise. 27 participants undertook this test, 3 refused, 8 were unable to undertake exercise testing due to mobility problems and/or shortness of breath, one had unstable angina and in one case equipment failure resulted in loss of data. There was no convincing evidence of an acute effect of yoga on BP, heart rate or estimated peak $VO_2$. (Table 5a) Salivary cortisol and amylase were similar at the waking+12 hours and bedtime periods after the first yoga session compared with pre-intervention levels. (Table 5b)

## Discussion

We show no additional cardiovascular benefit of a 3-month yoga intervention over and above usual care including CR in a randomised trial in people who had experienced an acute coronary event. Specifically, there was no additional impact on our co-primary outcomes of E/e' or exercise capacity, nor on a wide range of other secondary outcomes including measures of cardiac structure and function, brachial, central and ambulatory blood pressure, blood pressure and heart rate responses to exercise, estimated peak $VO_2$, carotid intima media thickness, blood lipids and glucose, obesity measures including fat mass and body mass index, self-reported physical activity levels, distance walked in the 6 minute walk test alcohol, smoking and dietary intake.

Of the cardiovascular risk factors studied to date, blood pressure appears the most consistently beneficially affected by yoga,(8, 10, 44) with reports that reductions in blood pressure are similar to those obtained by anti-hypertensive medication.(45) However, a community-based crossover study in India of non-pharmacological interventions showed that physical exercise (brisk walking for 50-60 minutes, 3-4 days a week for eight weeks) was a more effective method of reducing blood pressure, compared with yoga training or salt reduction, which both had similar smaller effects.(46) More recently, a community



For peer review only - http://bmjopen.bmj.com/site/about/guidelines.xhtml

based randomised controlled trial in Sweden found no evidence of reductions in resting blood pressure due to a 3-month yoga intervention(47) - similar to our findings.

The acute effects of yoga on cardiovascular responses to exercise have not been well studied, although a study of 33 female college students in the USA reported that reduction in salivary cortisol following one hour sessions of power yoga, stretch yoga or control (watching an educational movie for one hour) were similar between interventions.(48) We saw little change in cortisol levels in the either group at 3-month follow-up or in the yoga group albeit later in the day following the first yoga session. At 3 months of follow-up, improvements in heart rate and estimated peak $VO_2$ were seen in both groups compared with the pre-intervention levels but there was no evidence of benefit from yoga. Likewise, both groups improved in terms of distance walked in the 6-minute walk test at 3 months of follow-up but addition of yoga to usual care had no effect compared with usual care alone. A USA based study in heart failure patients reported a 0.5 SD improvement in exercise tolerance (+17% in 9 patients with heart failure enrolled to an 8 week yoga programme and –7% in 10 patients enrolled to receive standard medical therapy alone(39)). The same study also showed greater improvements in quality of life in the yoga group (scores improved by 26% in the yoga group and by 3% in the standard medical therapy group(39)). Heart failure was relatively infrequent in our study participants, (less than 20% reported previously diagnosed heart failure and the median ejection fraction was 54% which is only slightly below the reported lower limit of the normal reference ranges for male and female Europeans (55.8% in men and 57.3% in women)(49)); but whether this can account for differences between study findings must remain speculative.

Measures of HRV may be more sensitive to subtle changes than traditional tests of autonomic function, and improvements associated with yoga training or tai chi have been observed in systematic reviews for a number of parameters;(50, 51) however, we found little evidence for any yoga-related benefits in these parameters over 3 months.

Yoga has been variously shown to reduce fasting glucose and glycated haemoglobin, insulin, total and LDL cholesterol, triglyceride and weight, even in those without diabetes (52), although not all studies have shown a consistent benefit across these risk factors (7, 53, 54). We found no evidence of yoga-related benefits in blood lipids, glucose or obesity measures. Statin use was high (89%+) in both our study groups which may limit the measurable effect of interventions on lipid levels.

A 3 month multimodality intervention including yoga improved stress management, stress, depression, hostility and quality of life in a large group of CHD patients, although this study lacked a control arm. (55, 56) While we showed little change in perceived stress and did not measure stress management, depression or hostility, there was a small improvement in the EQ5D measure of health status based on UK population norms in the yoga group in our study although the 95% confidence interval of the difference included the null.

Impacts of yoga on subclinical and clinical cardiovascular disease have been inconsistently studied, making it difficult to place in context our findings of no evidence of improvement in E/e' due to yoga, although E/e' appeared lower in both groups at follow-up compared with baseline. Although our participants had pre-intervention levels of E/e' that could be considered 'normal', it is important to note that increased cardiovascular risk increases linearly across the entire range of E/e' and there is evidence that exercise can improve diastolic function even in healthy individuals. (57) Yoga in comparison with walking has been reported to improve cardiac function in older hypertensive individuals in India (58) and, in a high risk subgroup of older individuals in the USA a multimodality intervention including yoga reduced carotid intima media thickness to a greater extent than usual care or dietary and exercise advice; (53) however, no effect



For peer review only - http://bmjopen.bmj.com/site/about/guidelines.xhtml

of the intervention was seen in the whole group, and numbers in the high risk subgroups were small. Although our study was not designed or powered for long-term follow-up of cardiac events, we note that a multi-modality intervention including yoga, in a group of 35 participants, showed reduced numbers of cardiac events and progression of atherosclerosis to a greater extent in the lifestyle intervention arm compared with the control arm over 5 years of follow-up. (59) Similarly in 123 angiographically documented moderate to severe coronary artery disease patients, the Mount Abu Open Heart Trial of a multimodality intervention including Rajyoga meditation showed a trend towards regression of coronary lesions and a reduction in coronary events in those most adherent to the programme compared with those least adherent. (60)

As noted earlier, there is a general difficulty in comparing studies due to the wide variation in study designs and populations. A recent systematic review of 306 randomised controlled trials of yoga found that 91% reached positive conclusions (61). The authors confirmed difficulty in comparison of results across all trials, due to the common lack of *a priori* defined primary outcomes and appropriate group comparisons.

Strengths and limitations: This is the first study to our knowledge to adopt a comprehensive approach to measuring cardiovascular clinical and subclinical outcomes in response to a yoga intervention. It is also unusual in studying outcomes in a real world setting in an older group of people eligible for CR following an acute coronary event; however the trial was not designed to establish whether yoga may have benefits in terms of cardiovascular events or angina, for this much larger studies would be required. Also for some outcomes, such as IMT, it is likely that 3 months is too short a time to observed substantial regression. Dropout in the yoga arm of the study exceeded that in the usual care arm, (25 and 35 completed the study respectively), possibly reflecting the dual burden of attending both yoga training and usual CR. Consequently, the study will not have achieved the planned statistical power, as we had estimated a requirement for 33 in each group to enable detection of a 0.5SD difference in the primary outcomes, although given the measured effect sizes and confidence intervals, we believe if there are benefits of yoga on the measured outcomes, they are likely to be small. We did not adjust for multiple testing as we had identified *a priori* relevant primary outcomes for the trial, but in practice adjustment for multiple testing would not have altered our interpretation given the null findings. Cardiac rehabilitation is standard care following an acute coronary event in the UK, thus ethical reasons prevented comparison of yoga-based CR directly with usual CR alone - hence this study cannot tell us about the potential of yoga as an alternative to traditional CR. Our findings also cannot be generalized to other conditions that may benefit from cardiac rehabilitation, such as heart failure, post-valve replacement, stable angina pectoris, or symptomatic peripheral artery disease.(25) It should also be noted that our study was designed as a mechanistic study to complement a larger (around 4000 patients) Indian Council for Medical Research and Medical Research Council, UK funded study of yoga as a primary method of CR in India, which may shed light on some of the issues discussed above. (24)

Conclusion:

In this small UK-based randomised mechanistic study with 60 completing participants (of whom 25 were in the yoga+usual care group), we found no discernible improvement associated with the addition of a structured 3 month yoga intervention to usual cardiac rehabilitation care in any key cardiovascular or neuroendocrine outcomes shown to be responsive to yoga in previous mechanistic studies.

We suggest that usual care CR programmes in the UK, which include exercise, and optimisation of medical therapy may leave little additional scope for added benefits from a further intervention such as yoga.



For peer review only - http://bmjopen.bmj.com/site/about/guidelines.xhtml

For peer review only

Acknowledgements: We thank the cardiac rehabilitation teams at Ealing Hospital and the Flexi-Heart cardiac rehabilitation teams in Harrow and Brent for their help in identifying suitable participants; Daniel Key for building the study databases and managing the randomisation processes; Paula Carvelli, April McGowan for their help in recruitment and clinical measurements; Nadia de Couto Franscisco and Karikahan Manoharan for echocardiography measurements and analysis; Vincent van Hees (www.esciencecenter.nl) for performing analyses of GeneActiv physical activity data; and the participants for their valuable support.

Author contributions: SK, NC, AH, SE and IR were involved in the conception and design of the study. CT was responsible for data acquisition. BS, KC, SK and CT designed the yoga intervention. NS and PW contributed to data acquisition by performing salivary analyses. TT carried out the statistical analyses and wrote the first draft, with support from SK, AH, NC. All authors critically reviewed and revised all drafts and approved and are accountable for the accuracy and integrity of the final version. The corresponding author attests that all listed authors meet authorship criteria and that no others meeting the criteria have been omitted. TT is the guarantor.

Transparency statement: The lead author (TT) affirms that the manuscript is an honest, accurate, and transparent account of the study being reported; that no important aspects of the study have been omitted; and that any discrepancies from the study as originally planned and registered have been explained.

Funding and role of the funding source: The study was funded by the UK Medical Research Council (MR/J000175/1). The funders and study sponsors have played no role in the study design; in the collection, analysis, and interpretation of data; in the writing of the report; nor in the decision to submit the article for publication. The researchers were independent from the funders and sponsors and all authors had full access to all of the data (including statistical reports and tables) in the study and can take responsibility for the integrity of the data and the accuracy of the data analysis.

Competing Interest statement: All authors have completed the ICMJE uniform disclosure form at http://www.icmje.org/coi_disclosure.pdf and declare no support from any organisation for the submitted work, no financial relationships with any organisations that might have an interest in the submitted work in the previous three years and no other relationships or activities that could appear to have influenced the submitted work.

Ethical approval was granted by Camberwell St Giles Research Ethics Committee (Ref: 12/LO/0597)

Data sharing statement: De-identified participant data are available upon reasonable request to Professor Nish Chaturvedi (n.chaturvedi@ucl.ac.uk)

References

1. Feuerstein G. The yoga tradition. Prescott: Hohm Press; 1998.



For peer review only - http://bmjopen.bmj.com/site/about/guidelines.xhtml

2. Choices N. A Guide to Yoga: Department of Health and Social Care, UK Government; [Available from: https://www.nhs.uk/live-well/exercise/guide-to-yoga/.
3. Chong CS, Tsunaka M, Tsang HW, Chan EP, Cheung WM. Effects of yoga on stress management in healthy adults: A systematic review. Alternative therapies in health and medicine. 2011;17(1):32-8.
4. Cramer H, Lauche R, Langhorst J, Dobos G. Yoga for depression: a systematic review and meta-analysis. Depression and anxiety. 2013;30(11):1068-83.
5. Cramer H, Langhorst J, Dobos G, Lauche R. Associated Factors and Consequences of Risk of Bias in Randomized Controlled Trials of Yoga: A Systematic Review. PLoS One. 2015;10(12):e0144125.
6. Cramer H, Lauche R, Haller H, Steckhan N, Michalsen A, Dobos G. Effects of yoga on cardiovascular disease risk factors: a systematic review and meta-analysis. Int J Cardiol. 2014;173(2):170-83.
7. Innes KE, Vincent HK. The influence of yoga-based programs on risk profiles in adults with type 2 diabetes mellitus: a systematic review. Evid Based Complement Alternat Med. 2007;4(4):469-86.
8. Jayasinghe SR. Yoga in cardiac health (a review). Eur J Cardiovasc Prev Rehabil. 2004;11(5):369-75.
9. Ross A, Thomas S. The health benefits of yoga and exercise: a review of comparison studies. J Altern Complement Med. 2010;16(1):3-12.
10. Yang K. A review of yoga programs for four leading risk factors of chronic diseases. Evid Based Complement Alternat Med. 2007;4(4):487-91.
11. Kwong JS, Lau HL, Yeung F, Chau PH. Yoga for secondary prevention of coronary heart disease. Cochrane Database Syst Rev. 2015(7):Cd009506.
12. Cramer H, Langhorst J, Dobos G, Lauche R. Yoga for metabolic syndrome: A systematic review and meta-analysis. Eur J Prev Cardiol. 2016;23(18):1982-93.
13. Anderson L, Thompson DR, Oldridge N, Zwisler AD, Rees K, Martin N, et al. Exercise-based cardiac rehabilitation for coronary heart disease. Cochrane Database Syst Rev. 2016(1):Cd001800.
14. NACR. The National Audit of Cardiac Rehabilitation—Annual Statistical Report 2014.; 2015.
15. Nagueh SF, Appleton CP, Gillebert TC, Marino PN, Oh JK, Smiseth OA, et al. Recommendations for the evaluation of left ventricular diastolic function by echocardiography. J Am Soc Echocardiogr. 2009;22(2):107-33.
16. Hillis GS, Moller JE, Pellikka PA, Gersh BJ, Wright RS, Ommen SR, et al. Noninvasive estimation of left ventricular filling pressure by E/e' is a powerful predictor of survival after acute myocardial infarction. Journal of the American College of Cardiology. 2004;43(3):360-7.
17. Sandercock G, Hurtado V, Cardoso F. Changes in cardiorespiratory fitness in cardiac rehabilitation patients: a meta-analysis. Int J Cardiol. 2013;167(3):894-902.
18. Bellet RN, Adams L, Morris NR. The 6-minute walk test in outpatient cardiac rehabilitation: validity, reliability and responsiveness--a systematic review. Physiotherapy. 2012;98(4):277-86.
19. Hanson LC, McBurney H, Taylor NF. The retest reliability of the six-minute walk test in patients referred to a cardiac rehabilitation programme. Physiotherapy research international : the journal for researchers and clinicians in physical therapy. 2012;17(1):55-61.
20. Edelmann F, Gelbrich G, Dungen HD, Frohling S, Wachter R, Stahrenberg R, et al. Exercise training improves exercise capacity and diastolic function in patients with heart failure with preserved ejection fraction: results of the Ex-DHF (Exercise training in Diastolic Heart Failure) pilot study. J Am Coll Cardiol. 2011;58(17):1780-91.
21. Schultz MG, Otahal P, Cleland VJ, Blizzard L, Marwick TH, Sharman JE. Exercise-induced hypertension, cardiovascular events, and mortality in patients undergoing exercise stress testing: a systematic review and meta-analysis. Am J Hypertens. 2013;26(3):357-66.
22. Beatty AL, Schiller NB, Whooley MA. Six-minute walk test as a prognostic tool in stable coronary heart disease: data from the heart and soul study. Arch Intern Med. 2012;172(14):1096-102.
23. Filipovsky J, Ducimetiere P, Safar ME. Prognostic significance of exercise blood pressure and heart rate in middle-aged men. Hypertension. 1992;20(3):333-9.
24. Chandrasekaran AM, Kinra S, Ajay VS, Chattopadhyay K, Singh K, Singh K, et al. Effectiveness and cost-effectiveness of a Yoga-based Cardiac Rehabilitation (Yoga-CaRe) program following acute myocardial infarction: Study rationale and design of a multi-center randomized controlled trial. Int J Cardiol. 2019.
25. BACPR. The BACPR Standards and Core Components for Cardiovascular Disease Prevention and Rehabilitation 2012. 2012.



For peer review only - http://bmjopen.bmj.com/site/about/guidelines.xhtml

26. Park CM, Tillin T, March K, Ghosh AK, Jones S, Wright A, et al. Hyperglycemia has a greater impact on left ventricle function in South Asians than in Europeans. Diabetes Care. 2014;37(4):1124-31.
27. Borg GA. Psychophysical bases of perceived exertion. Med Sci Sports Exerc. 1982;14(5):377-81.
28. Hughes AD, Chaturvedi N. Estimation of Maximal Oxygen Consumption and Heart Rate Recovery Using the Tecumseh Sub-Maximal Step Test and their Relationship to Cardiovascular Risk Factors. Artery Res. 2017;18:29-35.
29. Montoye HJ, Willis PW, 3rd, Cunningham DA, Keller JB. Heart rate response to a modified Harvard step test: males and females, age 10-69. Res Q. 1969;40(1):153-62.
30. Park CM, Korolkova O, Davies JE, Parker KH, Siggers JH, March K, et al. Arterial pressure: agreement between a brachial cuff-based device and radial tonometry. Journal of hypertension. 2014;32(4):865-72.
31. Pucci G, Cheriyan J, Hubsch A, Hickson SS, Gajendragadkar PR, Watson T, et al. Evaluation of the Vicorder, a novel cuff-based device for the noninvasive estimation of central blood pressure. Journal of hypertension. 2013;31(1):77-85.
32. Weiss W, Gohlisch C, Harsch-Gladisch C, Tolle M, Zidek W, van der Giet M. Oscillometric estimation of central blood pressure: validation of the Mobil-O-Graph in comparison with the SphygmoCor device. Blood pressure monitoring. 2012;17(3):128-31.
33. Staessen J, Bulpitt CJ, Fagard R, Mancia G, O'Brien ET, Thijs L, et al. Reference values for the ambulatory blood pressure and the blood pressure measured at home: a population study. Journal of human hypertension. 1991;5(5):355-61.
34. Bathula R, Hughes AD, Panerai R, Potter J, Thom SA, Francis DP, et al. Indian Asians have poorer cardiovascular autonomic function than Europeans: this is due to greater hyperglycaemia and may contribute to their greater risk of heart disease. Diabetologia. 2010;53(10):2120-8.
35. van Hees VT, Fang Z, Langford J, Assah F, Mohammad A, da Silva IC, et al. Autocalibration of accelerometer data for free-living physical activity assessment using local gravity and temperature: an evaluation on four continents. J Appl Physiol (1985 ). 2014;117(7):738-44.
36. Tillin T, Forouhi NG, McKeigue PM, Chaturvedi N. Southall And Brent REvisited: Cohort profile of SABRE, a UK population-based comparison of cardiovascular disease and diabetes in people of European, Indian Asian and African Caribbean origins. Int J Epidemiol. 2012;41(1):33-42.
37. Cohen S, Kamarck T, Mermelstein R. A global measure of perceived stress. J Health Soc Behav. 1983;24(4):385-96.
38. Konar D, Latha R, Bhuvaneswaran JS. Cardiovascular responses to head-down-body-up postural exercise (Sarvangasana). Indian J Physiol Pharmacol. 2000;44(4):392-400.
39. Pullen PR, Nagamia SH, Mehta PK, Thompson WR, Benardot D, Hammoud R, et al. Effects of yoga on inflammation and exercise capacity in patients with chronic heart failure. J Card Fail. 2008;14(5):407-13.
40. Schrover R, Evans K, Giugliani R, Noble I, Bhattacharya K. Minimal clinically important difference for the 6-min walk test: literature review and application to Morquio A syndrome. Orphanet journal of rare diseases. 2017;12(1):78.
41. Wright DJ, Khan KM, Gossage EM, Saltissi S. Assessment of a low-intensity cardiac rehabilitation programme using the six-minute walk test. Clinical rehabilitation. 2001;15(2):119-24.
42. Borm GF, Fransen J, Lemmens WA. A simple sample size formula for analysis of covariance in randomized clinical trials. Journal of clinical epidemiology. 2007;60(12):1234-8.
43. Huber PJ, Ronchetti E. Robust statistics. 2nd ed. ed. Oxford: Wiley-Blackwell; 2009.
44. Hagins M, States R, Selfe T, Innes K. Effectiveness of yoga for hypertension: systematic review and meta-analysis. Evidence-based complementary and alternative medicine : eCAM. 2013;2013:649836.
45. Murugesan R, Govindarajulu N, Bera TK. Effect of selected yogic practices on the management of hypertension. Indian J Physiol Pharmacol. 2000;44(2):207-10.
46. Subramanian H, Soudarssanane MB, Jayalakshmy R, Thiruselvakumar D, Navasakthi D, Sahai A, et al. Non-pharmacological Interventions in Hypertension: A Community-based Cross-over Randomized Controlled Trial. Indian J Community Med. 2011;36(3):191-6.
47. Wolff M, Rogers K, Erdal B, Chalmers JP, Sundquist K, Midlov P. Impact of a short home-based yoga programme on blood pressure in patients with hypertension: a randomized controlled trial in primary care. Journal of human hypertension. 2016;30(10):599-605.



For peer review only - http://bmjopen.bmj.com/site/about/guidelines.xhtml

48. Sullivan M, Carberry A, Evans ES, Hall EE, Nepocatych S. The effects of power and stretch yoga on affect and salivary cortisol in women. Journal of health psychology. 2017:1359105317694487.
49. Kou S, Caballero L, Dulgheru R, Voilliot D, De Sousa C, Kacharava G, et al. Echocardiographic reference ranges for normal cardiac chamber size: results from the NORRE study. Eur Heart J Cardiovasc Imaging. 2014;15(6):680-90.
50. Zou L, Sasaki JE, Wei GX, Huang T, Yeung AS, Neto OB, et al. Effects of Mind(-)Body Exercises (Tai Chi/Yoga) on Heart Rate Variability Parameters and Perceived Stress: A Systematic Review with Meta-Analysis of Randomized Controlled Trials. Journal of clinical medicine. 2018;7(11).
51. Posadzki P, Kuzdzal A, Lee MS, Ernst E. Yoga for Heart Rate Variability: A Systematic Review and Meta-analysis of Randomized Clinical Trials. Applied psychophysiology and biofeedback. 2015;40(3):239-49.
52. Bijlani RL, Vempati RP, Yadav RK, Ray RB, Gupta V, Sharma R, et al. A brief but comprehensive lifestyle education program based on yoga reduces risk factors for cardiovascular disease and diabetes mellitus. J Altern Complement Med. 2005;11(2):267-74.
53. Fields JZ, Walton KG, Schneider RH, Nidich S, Pomerantz R, Suchdev P, et al. Effect of a multimodality natural medicine program on carotid atherosclerosis in older subjects: a pilot trial of Maharishi Vedic Medicine. Am J Cardiol. 2002;89(8):952-8.
54. Mahajan AS, Reddy KS, Sachdeva U. Lipid profile of coronary risk subjects following yogic lifestyle intervention. Indian Heart J. 1999;51(1):37-40.
55. Govil SR, Weidner G, Merritt-Worden T, Ornish D. Socioeconomic status and improvements in lifestyle, coronary risk factors, and quality of life: the Multisite Cardiac Lifestyle Intervention Program. American journal of public health. 2009;99(7):1263-70.
56. Daubenmier JJ, Weidner G, Sumner MD, Mendell N, Merritt-Worden T, Studley J, et al. The contribution of changes in diet, exercise, and stress management to changes in coronary risk in women and men in the multisite cardiac lifestyle intervention program. Annals of behavioral medicine : a publication of the Society of Behavioral Medicine. 2007;33(1):57-68.
57. Levy WC, Cerqueira MD, Harp GD, Johannessen KA, Abrass IB, Schwartz RS, et al. Effect of endurance exercise training on heart rate variability at rest in healthy young and older men. The American journal of cardiology. 1998;82(10):1236-41.
58. Patil SG, Patil SS, Aithala MR, Das KK. Comparison of yoga and walking-exercise on cardiac time intervals as a measure of cardiac function in elderly with increased pulse pressure. Indian Heart J. 2017;69(4):485-90.
59. Ornish D, Scherwitz LW, Billings JH, Brown SE, Gould KL, Merritt TA, et al. Intensive lifestyle changes for reversal of coronary heart disease. Jama. 1998;280(23):2001-7.
60. Gupta SK, Sawhney RC, Rai L, Chavan VD, Dani S, Arora RC, et al. Regression of coronary atherosclerosis through healthy lifestyle in coronary artery disease patients--Mount Abu Open Heart Trial. Indian Heart J. 2011;63(5):461-9.
61. Cramer H, Lauche R, Langhorst J, Dobos G. Are Indian yoga trials more likely to be positive than those from other countries? A systematic review of randomized controlled trials. Contemp Clin Trials. 2015;41:269-72.

For peer review only

Table 1: Pre-intervention characteristics by randomisation group (unadjusted)

| N(%) or means(95%CI) unless otherwise stated | Yoga + usual care | Usual Care | P value for between group difference |
|---|---|---|---|
| Pre-intervention | N=40 | N=40 | |
| Ethnicity: South Asian | 25(63%) | 26(65%) | 0.8 |
| Sex: Male | 28(70%) | 26(67%) | 0.8 |
| Age: years | 57.4(54.1, 60.7), range(35, 77) | 56.9(53.8, 60.0), range(35, 78) | 0.8 |
| Days since coronary event | 50(43, 57) | 59(53, 65) | 0.09 |
| Diabetes* (self report of physician diagnosis/anti-diabetic medication) | 15(38%) | 14 (35%) | 0.8 |
| Hypertension*( self report of physician diagnosis) | 29/37 (78%) | 25/37(68%) | 0.3 |
| Heart Failure* (self report of physician diagnosis) | 7/29(19%) | 7/29(19%) | 1.0 |
| Antihypertensive medications* | 39(98%) | 36(90%) | 0.17 |
| Number of antihypertensive medications, median(95%CI)* | 3(3,3) | 3(3,3) | 0.8 |
| Beta blockers* | 33(83%) | 32(80%) | 0.8 |
| Statins* | 36(90%) | 36(90%) | 1.0 |
| Current smoker/ex/never smoker, number* | 4/14/19 | 1/14/24 | 0.3 |
| Alcohol: never/ever drinkers, number* | N=36<br>Never drinkers: 13<br>Ever drinkers: 23 | N=35<br>Never drinkers: 10<br>Ever drinkers: 25 | 0.5 |
| Units/week (ever drinkers)*, median(95% CI) | 2.5(0, 10) | 4(1, 7) | 0.9 |
| Currently employed* | N=35<br>15(43%) | N=32<br>15(47%) | 0.7 |

*self-reported, N=number of responses to questionnaire item if incomplete

For peer review only



For peer review only - http://bmjopen.bmj.com/site/about/guidelines.xhtml

Table 2: <u>Chronic study: **Primary outcomes**</u>: Recruitment and three month follow-up (includes only those who attended study clinics at both time points and attended at least 18 classes if in the yoga group: N=35 in usual care group and N=25 in yoga group, unless otherwise stated)

| | Pre-intervention Means(95%CI) | | 3 months follow-up Means(95%CI), adjusted for pre-intervention levels | | Between group difference adjusted for pre-intervention levels (95%CI) | P value for between group difference, adjusted for pre-intervention levels |
|---|---|---|---|---|---|---|
| | Yoga + usual care | Usual care | Yoga + usual care | Usual care | Yoga minus usual care | |
| **Diastolic function** | N=25 | N=33* | N=25 | N=33* | | |
| E/e'. Median(95%CI) | 9.74(8.37, 11.12) | 8.72(7.76, 9.68) | 8.81(8.33, 9.29) | 8.26(7.79, 8.74) | -0.40 (-1.38, 0.58) | 0.4 |
| **6-minute walk test** | N=19*** | N=30** | N=19*** | N=30** | | |
| Total Distance, m | 462 (449, 517) | 442 (402,482) | 488 (463, 513) | 491 (471, 512) | -7 (-39, 26) | 0.7 |
| Total minutes walked | 6.0(6.0, 6.0) | 5.8(5.4, 6.0) | 5.9(5.7, 6.1) | 5.8(5.6, 6.0) | 0.1(-0.3, 0.5) | 0.5 |
| Distance, m/minute | 77(72, 82) | 77(71, 82) | 81(78, 83) | 81(78, 83) | 0.8(-4.6, 6.2) | 0.8 |
| Fatigue (Borg scale: 0-10) | | | | | | |
| Pre test | 0.2 | 0.2 | 0.02 | 0 | 0.07 | 0.17 |
| | (0, 0.7) | (0, 0.5) | (-0.03, 0.07) | (0, 0.06) | (-0.03, 0.2) | 0.9 |
| Post-test | 0.6 (0, 1.4) | 0.7 (0.03, 1.4) | 0.08 (-0.06, 0.20) | 0.08 (-0.04, 0.19) | -0.01 (-0.3,0.2) | |
| Dyspnoea (Borg Scale: 0-10) | | | | | | |
| Pre-test | 0 (0, 0) | 0.07(0, 0.23) | 0(0, 0.09) | 0.04(0, 0.10) | -0.04(-0.1, 0.07) | 0.5 |
| Post-test | 0.6(0, 1.2) | 1.0(0.3, 1.7) | 0.2(-0.3, 0.8) | 0.6(0.2, 1.0) | -0.2(-0.9, 0.5) | 0.5 |
| **Response to exercise:3-minute step test** | N=18*** | N=30** | N=18*** | N=30** | | |
| <u>Pre-step test</u> | | | | | | |
| Brachial SBP, mm Hg | 140 (134, 146) | 135 (130, 140) | 138 (133, 140) | 131 (126, 136) | 6 (-5, 18) | 0.3 |
| Brachial DBP, mm Hg | 83 (80, 85) | 80 (78, 83) | 82 (79, 84) | 79 (77, 82) | 2 (-4, 9) | 0.4 |
| Heart rate, bpm | 60(56, 64) | 62(59, 66) | 58(54, 61) | 60(57, 63) | -2(-8,5) ) | 0.6 |
| <u>Immediately post-step test</u> | | | | | | |
| Brachial SBP, mm Hg | 161 (155, 167) | 152 (147, 157) | 156 (151, 162) | 149 (145, 155) | 2 (-10, 14) | 0.7 |
| Brachial DBP, mm Hg | 85(82, 87) | 80(78, 83) | 77(74, 80) | 77(74, 79) | -1(-8, 5) | 0.7 |
| Heart rate, bpm | 89 (85, 93) | 89(86,93) | 81(77, 85) | 85(82, 88) | -4(-11, 3) | 0.2 |
| Peak $VO_2$ | N=14 | N=27 | N=14 | N=27 | | |
| ml/min/kg | 35.7 | 38.2 | 41.3 | 42.6 | 0.4 | 0.9 |



For peer review only - http://bmjopen.bmj.com/site/about/guidelines.xhtml

| | Pre-intervention Means(95%CI) | | 3 months follow-up Means(95%CI), adjusted for pre-intervention levels | | Between group difference adjusted for pre-intervention levels (95%CI) | P value for between group difference, adjusted for pre-intervention levels |
|---|---|---|---|---|---|---|
| | Yoga + usual care | Usual care | Yoga + usual care | Usual care | Yoga minus usual care | |
| | (31.5, 40.0) | (34.3, 42.1) | (38.0, 44.6) | (39.3, 45.8) | (-5.9, 6.6) | |
| <u>3 minutes post-step test</u> | | | | | | |
| Brachial SBP, mm Hg | 143 (137, 149) | 134 (129, 139) | 144 (138, 150) | 136 (131, 140) | 4(-8, 15) | 0.6 |
| Brachial DBP, mm Hg | 83(80, 86) | 80(78, 83) | 80(77, 83) | 79(77, 82) | -1(-3, 9) | 0.8 |
| Heart rate, bpm | 66(62, 70) | 65(61, 68) | 61 (57, 65) | 60(58, 64) | 0(-7, 7) | 1.0 |

*2 missing cases due to error readings
**5 missing cases: test not performed due to poor mobility(3), recent myocardial infarction(1), refused(1)
***7 missing cases: test not performed due to poor mobility(2), elevated blood pressure(>180/100mm Hg)(4), refused step-test(1)

For peer review only

Table 3: Chronic study: **Secondary outcomes**: Pre-intervention and 3-month follow-up (includes only those who attended study clinics at both time points and attended at least 18 classes if in the yoga group).
N=35 in usual care group and N=25 in yoga group, unless otherwise stated

| | Pre-intervention Means (95%CI) unless stated otherwise | | 3 months follow-up, adjusted for pre-intervention levels. Means (95%CI) unless stated otherwise | | Between group difference adjusted for pre-intervention levels (95% CI) | P value for between group difference, adjusted for pre-intervention levels |
|---|---|---|---|---|---|---|
| | Yoga + usual care | Usual care | Yoga + usual care | Usual care | Yoga minus usual care | |
| **Cardiac structure** | | | | | | |
| Left ventricular mass indexed to height $^{2.7}$, g/m $^{2.7}$, median(95%CI) | 40.1 (36.6, 51.2) | 38.7 (35.6, 43.5) | 39.4 (36.2, 42.5) | 39.3 (36.1, 42.5) | 3.4 (-3.3, 10.1) | 0.3 |
| Relative wall thickness, median(95%CI) | 0.45 (0.38, 0.48) | 0.42 (0.40, 0.47) | 0.41 (0.39, 0.44) | 0.42 (0.39, 0.44) | -0.01 (-0.07, 0.04) | 0.6 |
| Left atrial diameter indexed to height, cm/m | 2.2 (2.2, 2.3) | 2.3 (2.2, 2.4) | 2.2 (2.2, 2.3) | 2.2 (2.2, 2.3) | -0.08 (-0.21, 0.05) | 0.2 |
| **Cardiac function** | | | | | | |
| Ejection fraction, %, median(95%CI) | 54(44, 68) | 54(44, 64) | 54(44, 63) | 54(45, 63) | -0.5(-19, 18) | 1.0 |
| Mitral E:A ratio(of peak velocity in early diastole to peak velocity in late diastole, median(95%CI) | 1.02 (0.97, 1.12) | 1.16 (0.95, 1.29) | 1.09 (1.01, 1.18) | 1.14 (1.07, 1.23) | -0.01 (-0.20, 0.19) | 1.0 |
| s', peak velocity during systole, cm/sec, median(95%CI) | 7.11 (6.49, 8.25) | 7.31 (6.35, 7.64) | 7.25 (6.90, 7.59) | 7.16 (6. 82, 7.51) | 0.10 (-0.56, 0.75) | 0.8 |
| e', peak velocity during early diastole, cm/sec, median(95%CI) | 7.56 (6.77, 9.35) | 8.57 (7.58, 9.44) | 8.20 (7.73, 8.67) | 8.45 (7.98, 8.92) | 0.14 (-0.72, 0.99) | 0.8 |
| **Resting blood pressure and heart rate** | | | | | | |
| Heart rate, beats per minute(bpm) | 60(55, 64) | 64(60, 68) | 59(57, 61) | 61(60, 63) | -0.4(-4, 3) | 0.8 |
| Brachial SBP, mm Hg | 134 (125, 143) | 126(120, 133) | 127(124, 130) | 122(119, 125) | -2(-8, 4) | 0.5 |
| Brachial DBP, mm Hg | 77(74, 80) | 74(72, 76) | 75(73, 76) | 73(71, 74) | -0.8(-3, 2) | 0.6 |
| Central SBP, mm Hg | 128(119, 137) | 120(114, 125) | 122(119, 125) | 117(114, 120) | -1(-8, 6) | 0.8 |



For peer review only - http://bmjopen.bmj.com/site/about/guidelines.xhtml

| | Pre-intervention Means (95%CI) unless stated otherwise | | 3 months follow-up, adjusted for pre-intervention levels. Means (95%CI) unless stated otherwise | | Between group difference adjusted for pre-intervention levels (95% CI) | P value for between group difference, adjusted for pre-intervention levels |
|---|---|---|---|---|---|---|
| | Yoga + usual care | Usual care | Yoga + usual care | Usual care | Yoga minus usual care | |
| **24 hour ambulatory blood pressure** | N=20 | N=30 | N=20 | N=30 | | |
| Average day central SBP, mm Hg | 115 (108, 122) | 113 (109, 116) | 113 (110, 116) | 112 (109, 114) | 2(-4, 8) | 0.5 |
| Average night central SBP, mm Hg | 104(96, 112) | 104(98, 110) | 104(100,108) | 104(100, 108) | 1(-7, 10) | 0.8 |
| Average day heart rate, bpm | 65(60, 69) | 68(65, 71) | 64(63, 66) | 67(65, 69) | 2(-2, 5) | 0.3 |
| Average night heart rate, bpm | 59(54, 63) | 64(60, 67) | 59(57, 61) | 63(61, 65) | 4(-0.4, 7) | 0.08 |
| **Pulse wave velocity**, m/sec median(95%CI) | N=25 9.03(8.14, 9.67) | N=32 8.63(8.27, 9.10) | N=25 9.02(8.47, 9.59) | N=32 8.75(8.28, 9.22) | 0.5(-0.7, 1.7) | 0.3 |
| **Carotid intima media thickness (CIMT)**. Far wall, mm, maximum of means (without plaque &CIMT<1.5mm) | N=18 0.754 (0.691, 0.816) | N=28 0.746 (0.681, 0.810) | N=18 0.764 (0.699, 0.830) | N=28 0.761 (0.696, 0.827) | -0.1 (-0.2, 0.1) | 0.4 |
| **Bloods, fasting, medians(95%CIM)** | N=25 | N=31 | N=25 | N=31 | | |
| Triglycerides, mmol/l | 1.10 (0.94, 1.40) | 1.03 (0.96, 12.4) | 1.11 (1.01, 1.20) | 1.10 (1.00, 1.19) | -0.04 (-0.24, 0.17) | 0.7 |
| HDL cholesterol, mmol/l | 0.91 (0.84, 1.07) | 0.91 (0.79, 1.01) | 1.02 (0.97, 1.06) | 0.97 (0.92, 1.02) | 0.05 (-0.04, 0.14) | 0.3 |
| Total cholesterol, mmol/l | 3.00 (2.80, 3.50) | 3.20 (2.90, 3.60) | 3.30 (3.18, 3.41) | 3.36 (3.25, 3.47) | 0.07 (-0.29, 0.15) | 0.6 |
| Cholesterol:HDL ratio | 3.33 (2.82, 3.67) | 3.40 (3.23, 3.78) | 3.31 (3.13, 3.48) | 3.52 (3.34, 3.69) | -0.06 (-0.38, 0.26) | 0.7 |
| LDL cholesterol, mmol/l | 1.59 (1.35, 1.77) | 1.60 (1.54, 1.73) | 1.76 (1.66, 1.87) | 1.81 (1.71, 1.92) | 0.01 (-0.19, 0.20) | 0.9 |
| Glucose, mmol/l | 5.40 (4.90, 6.0) | 5.50 (4.90, 6.20) | 5.78 (5.62, 5.94) | 5.68 (5.52, 5.84) | 0.05 (-0.23, 0.33) | 0.7 |
| **Anthropometrics** | N=25 | N=35 | N=25 | N=35 | | |
| Body mass indexl, kg/m$^2$, median(95%CI) | 27.6 (25.1, 29.5) | 27.2 (25.3, 29.6) | 27.6 (27.4, 27.9) | 27.5 (27.3, 27.7) | 0(-0.5, 0.5) | 1.0 |
| Waist:hip ratio | 0.99 (0.96, 1.03) | 0.99 (0.96, 1.02) | 0.98 (0.97, 0.99) | 0.98 (0.97, 0.98) | 0.01 (-0.01, 0.02) | 0.3 |
| Fat mass percent | 28(25, 32) | 30(27, 33) | 28(27, 28) | 30(29, 30) | 0.2(-1, 2) | 0.8 |
| **HPA axis** | | | | | | |



For peer review only - http://bmjopen.bmj.com/site/about/guidelines.xhtml

For peer review only

| | Pre-intervention Means (95%CI) unless stated otherwise | | 3 months follow-up, adjusted for pre-intervention levels. Means (95%CI) unless stated otherwise | | Between group difference adjusted for pre-intervention levels (95% CI) | P value for between group difference, adjusted for pre-intervention levels |
|---|---|---|---|---|---|---|
| | Yoga + usual care | Usual care | Yoga + usual care | Usual care | Yoga minus usual care | |
| **Salivary cortisol nmol/L** | N=23 | N=29 | N=23 | N=29 | | |
| Waking | 11.2 (8.5,13.8) | 12.6 (10.3,14.9) | 11.2 (8.7, 13.7) | 12.5 (10.4, 14.7) | -1.3 (-4.2, 1.6) | 0.5 |
| Waking+30 minutes | 10.6 (8.0, 13.3) | 16.0(13.6, 18.3) | 10.4 (8.0, 12.9) | 12.9 (10.7, 15.0) | -2.5 (-5.4, 10.5) | 0.17 |
| Waking +1hrs 30 minutes | 5.2(2.5, 7.8) | 8.0(5.6, 10.4) | 8.0(5.6, 10.4) | 7.5(5.3, 9.7) | 0.5(-2.4, 3.4) | 0.8 |
| Waking +12 hours | 3.9(1.2, 6.7) | 3.2(0.8, 5.7) | 4.1(1.7, 6.6) | 2.5(0.3, 4.7) | 1.6(-1.4, 4.5) | 0.4 |
| Bedtime | 2.3(0, 5.0) | 3.1(0.7, 5.5) | 3.7(1.3, 6.2) | 2.2(0.04, 4.3) | 1.6(-1.3, 4.5) | 0.4 |
| **Exercise/physical activity** | | | | | | |
| **Average body acceleration over 3 days**, milli g (GeneActiv) | N=20 25.1 (20.4, 29.8) | N=28 22.5 (19.5, 25.5) | N=20 24.9 (22.7, 27.1) | N=28 23.5 (21.3, 25.7) | -4.0 (-8.3, 0.23) | 0.064 |
| **International Physical Activity Questionnaire(IPAQ)** self-report. Total metabolic equivalent(met) minutes/week. Median(95%CI) | N=25 693 (60, 1386) | N=34 1409 (495, 2310) | N=25 2273 (1434, 3112) | N=34 2899 (2065, 3734) | 179 (-1542, 1900) | 0.8 |
| **EQ5D** health status based on UK population norms(1= full health) median (95%CI) | N=14 0.77 (0.69,1.0) | N=21 0.80 (0.73,.81) | N=14 0.83 (0.70,0.97) | N=21 0.80 (0.66,0.93) | 0 (-0.24, 0.24) | 1.0 |
| **EQ5D** self-rated health thermometer (100=best possible), median(95%CI) | N=21 70(60,75) | N=27 70(50, 80) | N=25 73(68, 78) | N=27 73(68, 78) | 2.5(-5.8, 10.9) | 0.6 |
| **Perceived stress score** (possible range: 0-40. 13 is considered average, high stress groups: 20+) | N=25 14.9 (11.8, 18.0) | N=34 18.2 (15.2, 21.2) | N=25 14.7 (13.1, 16.4) | N=34 17.2 (15.5, 18.8) | -2.7(-6.1,5.1) | 0.11 |



For peer review only - http://bmjopen.bmj.com/site/about/guidelines.xhtml

Table 4. Chronic Study: Autonomic function: heart rate variability, baroreceptor sensitivity and salivary amylase

| | Pre-intervention | | 3 months follow-up adjusted for Pre-intervention level | | Between group difference adjusted for pre-intervention levels (95% CI) | P value for between-group difference adjusted for pre-intervention levels |
|---|---|---|---|---|---|---|
| | Yoga + usual care | Usual care | Yoga + usual care | Usual care | Yoga minus usual care | |
| **Heart rate variability and baroreceptor sensitivity 10 minute recording** Medians (95%CIM) | N=24 | N=33 | N=24 | N=33 | | |
| Number of beats | 677(605, 877) | 709(632, 798) | 644 (589, 698) | 638 (584, 693) | -1(-110, 108) | 1.0 |
| Number of ectopics | 19 (8, 71) | 22 (8, 48) | 29 (6, 53) | 26 (2, 49) | -1(-46, 44) | 1.0 |
| Mean RR interval , millisecond(ms) | 1016(888, 1128) | 977(925, 1073) | 1050(1012, 1089) | 1020(982, 1059) | 13(-62, 87) | 0.7 |
| SDNN, ms | 55.5 (39.7, 78.6) | 53.4 (37.4, 69.2) | 48.2 (36.7, 59.6) | 47.2 (35.9, 58.6) | 3.6 (-20.4, 27.5) | 0.8 |
| RMSSD, ms | 38.5 (30.0, 60.0) | 38.8 (30.2, 57.8) | 44.4 (35.2, 53.5) | 39.9 (30.8, 48.9) | 4.4 (-13.8, 22.8) | 0.6 |
| NN50 | 40(18,57) | 49(24,67) | 49 (31, 66) | 51(34, 68) | 17(-20, 54) | 0.4 |
| pNN50 | 0.08 (0.03, 0.11)) | 0.08 (0.04, 0.14) | 0.14 (0.10, 0.17) | 0.11 (0.07,0.15) | 0.01 (-0.06, -0.08) | 0.7 |
| Triangular index | 179(149, 231) | 177(139, 235) | 189 (163, 216) | 187(160, 214) | 20(-37, 77) | 0.5 |
| Total RR interval power, ms$^2$ | 1514 (633, 2339) | 995 (680, 2097) | 1157 (653, 1662) | 1171 (668, 1673) | 401 (-591, 1393) | 0.4 |
| LF RR interval power, ms$^2$ | 340(119, 613) | 284 (177, 491) | 340(143, 537) | 340(143, 538) | 20(-391, 430) | 0.9 |
| HF RR interval power, ms$^2$ | 251(77, 759) | 235(126, 317) | 265(113, 417) | 265(113, 16) | 101(-256, 457) | 0.6 |
| LF/HF power ratio | 1.5(0.8, 2.1) | 1.4(1.1, 1.7) | 1.2(0.9, 1.4) | 1.2 (0.9, 1.4) | 0.04(-0.5, 0.6) | 0.9 |
| LF RR interval power, normalised units(nu) | 0.23 (0.16, 0.32) | 0.27 (0.25, 0.30) | 0.28 (0.24, 0.31) | 0.29 (0.26, 0.32) | 0.03 (-0.03, 0.09) | 0.3 |
| HF RR interval power, nu | 0.17 (0.12, 0.33) | 0.19 (0.14, 0.28) | 0.23 (0.17, 0.29 | 0.23 (0.17, 0.29) | -0.02 (-0.13, 0.10) | 0.8 |
| LF alpha index, ms/mm Hg | 6.9 (4.6, 12.9) | 9.1(6.6, 13.6) | 10.1 (7.5, 12.7) | 10.4(7.8, 13.0) | 0.8(-4.4, 6.1) | 0.8 |
| HF alpha index, ms/mm Hg | 9.2(4.6, 25.6) | 13.9(9.2, 22.6) | 15.8 (7.7, 23.9) | 16.4(8.3, 24.5) | 3.7(-9.4, 16.8) | 0.6 |
| Baroreceptor sensitivity on | 9.9 (6.3, 16.1) | 10.0(7.0, 13.0) | 10.6 (8.2, 13.1) | 10.6(8.2, 13.0) | 1.5(-2.6, 5.6) | 0.5 |



For peer review only - http://bmjopen.bmj.com/site/about/guidelines.xhtml

| | Pre-intervention | | 3 months follow-up adjusted for Pre-intervention level | | Between group difference adjusted for pre-intervention levels (95% CI) | P value for between-group difference adjusted for pre-intervention levels |
|---|---|---|---|---|---|---|
| | Yoga + usual care | Usual care | Yoga + usual care | Usual care | Yoga minus usual care | |
| sequence analysis, ms/mmHg | | | | | | |
| | | | | | | |
| **Salivary amylase, log microunits/L ,** (means(95%CI)) | N=23 | N=32 | N=23 | N=32 | | |
| Waking | 4.19 (3.83, 4.41) | 4.48 (4.23, 4.73) | 4.39 (4.06, 4.71) | 4.45 (4.17, 4.74) | -0.05 (-0.68, 0.58) | 0.9 |
| Waking+30 minutes | 4.03 (3.74, 4.33) | 4.08 (3.83, 4.33) | 4.03 (3.71, 4.34) | 4.26 (3.98, 4.54) | -0.23 (-0.84, 0.39) | 0.5 |
| Waking +1hrs 30 minutes | 4.45 (4.16, 4.74) | 4.88 (4.62, 5.13) | 4.48 (4.16, 4.79) | 4.47 (4.19, 4.76) | -0.01 (-0.63, .61) | 1.0 |
| Waking +12 hours | 4.61 (4.30, 4.92) | 4.74 (4.48, 4.99) | 4.29 (3.97, 4.61) | 4.70 (4.42, 4.98) | -0.42 (-1.0, 0.21) | 0.19 |
| Bedtime | 4.42 (4.12, 4.72) | 4.66 (4.41, 4.92) | 4.56 (4.23, 4.88) | 4.44 (4.16, 4.73) | 0.11 (-0.52, 0.73) | 0.7 |

Table abbreviations:
Mean RR interval: time in milliseconds (ms) between successive R waves on ECG
SDNN: standard deviation of N-N intervals, ms
RMSSD: root mean square of successive R-R interval differences, ms
NN50: number of successive N-N intervals that differ by more than 50 ms
pNN50: Proportion of successive RR intervals that differ by more than 50 ms
LF RR interval power: absolute power of the low-frequency band (0.04–0.15 Hz), $ms^2$
HF RR interval power: absolute power of the high-frequency band (0.15–0.4 Hz, $ms^2$
LF/HF power ratio: Ratio of LF-to-HF power
LF RR interval power: Relative power of the low-frequency band (0.04–0.15 Hz) , normalised units(nu)
HF RR interval power: Relative power of the high-frequency band (0.15–0.4 Hz), nu
LF alpha index: Low frequency alpha index, ms/mm Hg
HF alpha index: High frequency alpha index, ms/mm Hg



For peer review only - http://bmjopen.bmj.com/site/about/guidelines.xhtml

For peer review only

Table 5a: Acute study, **Primary outcomes**: immediate post-exercise results following a 3-minute step-test, before and after (same day) the first yoga class

| **N=27** | **Before first yoga session** Mean (95%CI) | **After first yoga session:** Mean (95% CI) | **Post –pre first yoga session difference** Mean (95% CI) | **P value for comparison between before and after first yoga session** |
|---|---|---|---|---|
| **Resting seated** | | | | |
| Systolic blood pressure, mm Hg | 132(128, 136) | 133(129, 136) | 1(-9, 11) | 0.9 |
| Diastolic blood pressure, mm Hg | 78(77, 81) | 79(77, 81) | 1(-5, 7) | 0.7 |
| Heart rate, bpm | 69(67, 71) | 68(66, 70) | -1(-8, 6) | 0.9 |
| **Immediately post-step-test, standing** | | | | |
| Systolic blood pressure, mm Hg | 161(157, 164) | 156(148, 163) | -5(-15, 6) | 0.4 |
| Diastolic blood pressure, mm Hg | 80(78, 82) | 80(78, 82) | -1(-7, 5) | 0.8 |
| Heart rate, bpm | 92(90, 94) | 88(86, 90) | -3(-10, 4) | 0.4 |
| Peak $VO_2$, ml/min/kg (n=24) | 36.9(33.4, 40.4) | 38.9(35.4, 42.4) | 2.0(-0.2, 4.1) | 0.07 |
| **3 minutes post-step-test, seated** | | | | |
| Systolic blood pressure, mm Hg | 133(129, 136) | 135(131, 139) | 2(-8, 13) | 0.7 |
| Diastolic blood pressure, mm Hg | 78(76, 80) | 79(77, 82) | 1(-5, 8) | 0.6 |
| Heart rate, bpm | 72(70, 74) | 69(67, 71) | -3(-10, 4) | 0.4 |

*12 participants did not participate in the step-test both before and after the first yoga session due to: mobility problems+/- shortness of breath (n=3), refused (n=3), frailty (n=2), unstable angina (n=1), other (high blood pressure/dizziness/weakness on left side/restricted movement; n=3). Blood pressure readings were unavailable for 1 participant due to equipment failure.

Table 5b: Acute study: Secondary outcomes: salivary amylase and cortisol: pre-intervention and day of first yoga class

| | **Pre-intervention** Means(95%CI) | **Day of first yoga session** Means(95%CI) | **Post –pre first yoga session difference Means(95% CI)** |
|---|---|---|---|
| | N=32* | N=32* | |
| **Cortisol, nmol/l** | | | |
| Waking | 12.7(11.0, 14.4) | 15.4(12.8, 17.9)(pre yoga) | N/A |
| 12 hours after waking | 3.6(1.8, 5.4) | 4.4(1.7, 7.0) (post yoga) | 0.5(-3.5, 4.5) |
| Bedtime | 3.4(1.6, 5.2) | 3.0(0.3, 5.7) (post yoga) | -0.3(-4.3, 3.7) |
| **Amylase, log microunits/L** | | | |
| Waking | 4.40 (4.23, 4.57) | 4.28(4.02, 4.54) (pre yoga) | N/A |
| 12 hours after waking | 4.66(4.47, 4.84) | 4.58(4.32, 4.85) (post yoga) | -0.08(-0.54, 0.38) |
| Bedtime | 4.60(4.42, 4.78) | 4.74(4.47, 5.01) (post yoga) | 0.13(-0.32, 0.59) |

*8 participants were unable to provide adequate saliva samples pre-recruitment and on the day of the first yoga session. N/A, not available.



For peer review only - http://bmjopen.bmj.com/site/about/guidelines.xhtml

For peer review only

# Figure S1

## YACHT Study Recruitment

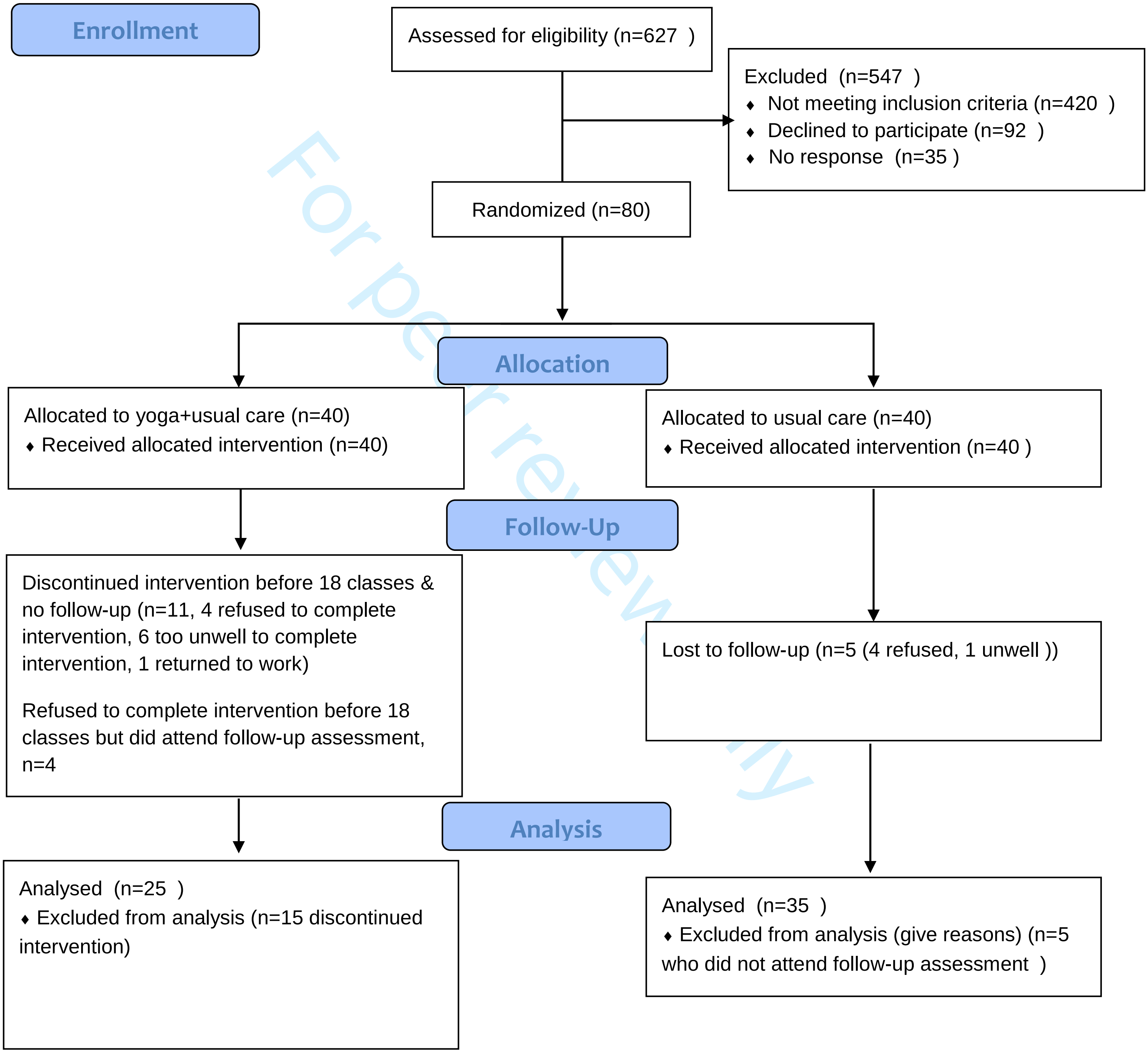


For peer review only

SUPPLEMENTAL Table S1a. Characteristics at start of study (pre intervention) of those who completed/did not complete 3month follow-up: Usual care group

| Means95%CI), number(%) unless otherwise stated | Usual Care, completed | Usual Care, dropped out |
|---|---|---|
| | N=35 | N=5 |
| Ethnicity: South Asian | 22(63%) | 4(80%) |
| Sex: Male | 22(63%) | 5(100%) |
| Age: years | 57.2(54.0, 59.8) | 54.5(38.3,73.6) |
| Days since coronary event | 58(52, 65) | 61(41,84) |
| Previous heart attack | 5/33(15%) | 0/5 |
| Diabetes, self report of physician diagnosis | 13(37%) | 1(20%) |
| Heart failure, self report of physician diagnosis | 6/31(19%) | 1(20%) |
| Hypertension, self report of physician diagnosis | 23/33 (70%) | 1/4(25%) |
| Blood pressure lowering medication* | 35(100%) | 5(100%) |
| Number of blood pressure lowering medications*, median(IQR) | 3(2,3) | 3(2,3) |
| Beta blocker use* | 27(77%) | 5(100%) |
| Statin use * | 31(89%) | 5(100%) |
| Current smoker/ex smoker/never smoker, number | N=31<br>1/13/17 | N=5<br>0/2/3 |
| Alcohol, never/ever drinkers, number<br>Units/week (ever drinkers), median(IQR) | N=30<br>7/23<br>2(1,6) | <br>3/2<br>7(7,7) |
| Reason given for dropout | | Refused follow-up (4)<br>Unwell (1) |

*self-reported either pre-intervention or at follow-up

For peer review only

Table S1b. Characteristics at start of study (pre intervention) of those who completed/did not complete 3 month follow-up. Yoga+usual care group

| Means(95%CI), number(%) unless otherwise stated | Yoga+usual care Completed 3 month follow-up +/- 18 sessions | Yoga+usual care Attended 3 month follow-up and at least 18 yoga sessions | Yoga+usual care Dropped out of yoga and follow-up | Yoga+usual care Attended 3 month follow-up but attended <18 yoga sessions |
|---|---|---|---|---|
| | N=29 (4 did not complete 18 sessions) | N=25 | N=11 | N=4 |
| Ethnicity: South Asian | 17(59%) | 15(60%) | 8(73%) | 2(50%) |
| Sex: Male | 21(72%) | 18(72%) | 7(64%) | 3(75%) |
| Age: years | 58.5(54.5, 62.5) | 57.9(53.6, 62.2) | 54.5(47.9, 61.2) | 62.2(42.8, 81.7) |
| Days since coronary event | 51(42,59) | 50±24 | 49±22 | 54(32,75) |
| Previous heart attack | 4/28 (14%) | 4/24(17%) | 3/7(30%) | 0/4 |
| Diabetes, self report of physician diagnosis | 12(41%) | 12(41%) | 3 (27%) | 3 (75%) |
| Heart failure, self report of physician diagnosis | 4/27(15%) | 2/23(9%) | 3/9(33%) | 2(50%) |
| Hypertension, self report of physician diagnosis | 23/28(82%) | 19/24(79%) | 5/8(63%) | 4(100%) |
| Blood pressure lowering medication* | 29(100%) | 25(100%) | 10/10(100%) | 4(100%) |
| Number of blood pressure lowering medications*, median(IQR) | 3(3,3) | 3(3, 3) | 3(2,3) | 3(2,4) |
| Beta blocker use* | 25(86%) | 22(88%) | 8(73%) | 3(75%) |
| Statin use * | 28(97%) | 24(96%) | 8(73%) | 4(100%) |
| Current smoker/ex smoker, number | N=27<br>2/10 | N=23<br>2/8 | 2/4 | 0/2 |
| Alcohol, never/ever drinkers, number<br>Units/week (ever drinkers), median(IQR) | N=26<br>8/18<br>2(0,5) | N=23<br>8/15<br>2(0,4) | N=10<br>5/5<br>11(1,12) | <br>0/4<br>9(1, 20) |
| Reason given for dropout | | | Refused follow-up (4)<br>Unwell(6)<br>Returned to work, unable to attend further classes/follow-up (1) | Refused further yoga(4) |

*self-reported either pre-intervention or at follow-up



For peer review only - http://bmjopen.bmj.com/site/about/guidelines.xhtml

For peer review only



For peer review only - http://bmjopen.bmj.com/site/about/guidelines.xhtml

# YACHT

Yoga And Cardiovascular Health Trial

# Manual of Operations

Version 1.2

July 2012

**Compiled by Barbara Sowa and Claire Tuson**

For peer review only

Imperial College London

# Table of Contents


**STUDY SUMMARY** ........ 3

**OUTLINE STUDY DESIGN** ........ 4

**YOGA SUMMARY** ........ 5

**YOGA FULL DESCRIPTION** ........ 5

PART 1: INITIAL RELAXATION AND WARM-UP – 10 MINUTES ........ 5

*Initial relaxation* ........ *6*

*Warm-up* ........ *7*

PART II: ASANAS - 20 MINUTES ........ 8

*Standing poses (High positions)* ........ *8*

*Kneeling & Sitting poses (Medium positions)* ........ *10*

*Lying down poses (Low positions)* ........ *13*

PART III: COOL DOWN: BREATHING EXERCISES AND FINAL RELAXATION - 20 MINUTES ........ 14

*Deep Abdominal Breathing* ........ *14*

*Full Yogic Breath* ........ *15*

*Final Relaxation* ........ *15*

*Alternative relaxation* ........ *16*

PART IV: SUPERVISION OF PARTICIPANTS POST-EXERCISE AND EDUCATION - 20 MINUTES ........ 18

*Proper Exercise* ........ *18*

*Proper Breathing* ........ *18*

*Proper Relaxation-Savasana* ........ *19*

*Proper Diet-Vegetarian – Part I* ........ *19*

*Proper Diet-Vegetarian – Part II* ........ *20*

*Positive Thinking and Meditation – Part I* ........ *20*

*Positive Thinking and Meditation – Part II* ........ *21*

*Making Positive Changes in Your Life* ........ *22*

**APPENDIX 1** ........ 23

POLICY FOR CARDIAC REHABILITATION IN EALING ........ 23

**APPENDIX 2** ........ 32

EALING CARDIAC REHABILITATION HEALTH EDUCATION TALKS ........ 32

*Drugs for heart disease and how they work* ........ *32*

*Managing Stress* ........ *34*

*Eating for a healthy heart* ........ *37*

*Exercise and the benefits for your heart* ........ *38*

*Risk factors and making lifestyle changes* ........ *41*

*The heart and how it works* ........ *44*

**REFERENCES** ........ 48


DRAFT
For peer review only

Imperial College London

## Study Summary

| | |
|---|---|
| **TITLE** | Yoga And Cardiovascular Health Trial (YACHT) |
| **DESIGN** | Epidemiological |
| **AIMS** | To perform a mechanistic study to determine the acute and chronic effects of yoga on neuro-endocrine pathways, and downstream effects on CVD risk factors and subclinical outcomes. This will provide complimentary information to a larger clinical trial in India designed to determine effects of yoga on cardiovascular morbidity and mortality in acute coronary syndromes. |
| **POPULATION** | Indian Asians and Europeans, 40 in each ethnic group, (self-defined, verified by country of birth of all 4 grandparents). Aged between 35 to 80 years, male or female, without co-morbid disease and mobility limitations that would preclude participation in cardiac rehabilitation and our investigations. |
| **ELIGIBILITY** | Referred to cardiac rehabilitation programmes in West London post-angioplasty as treatment for an acute coronary syndrome. Able to understand English or Punjabi, Hindi or Gujarati, but in order to be able to follow the yoga class instructions the participant will need to have a basic command of the English language. |
| **DURATION** | Recruitment is planned for 1 year. |

DRAFT



For peer review only - http://bmjopen.bmj.com/site/about/guidelines.xhtml

Imperial College London

# Outline Study Design

Indian Asians and Europeans, 40 in each ethnic group, (self-defined, verified by country of birth of all 4 grandparents), referred to cardiac rehabilitation programmes in West London post-angioplasty as treatment for an acute coronary syndrome, will be invited to participate when being discharged from hospital.

Those who agree will be randomized to the yoga intervention plus their standard cardiac rehabilitation programme (usual care), or usual care alone.

In order to evaluate the chronic effects of yoga, baseline and 3 month measurements will be performed on all participants as described below:

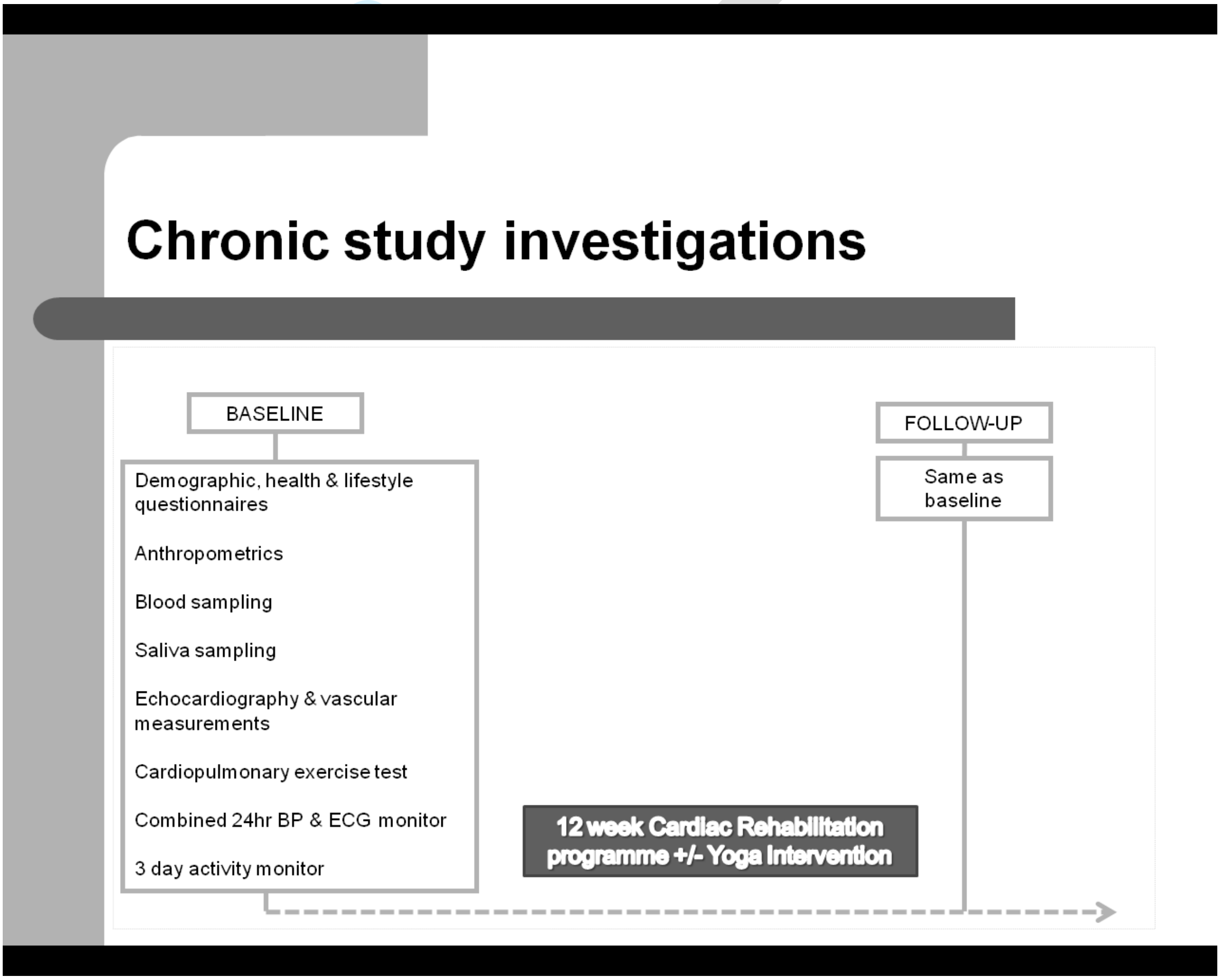

Imperial College London

## Yoga Summary

The yoga intervention will be delivered on a bi-weekly group session basis for 12 weeks alongside the cardiac rehabilitation programme.  There will be 24 yoga classes in total, of which, each participant will be required to attend a minimum of 18.  The yoga session will be designed and conducted by a teacher certified in yoga and cardiac rehabilitation, and will encompass physical fitness (yoga poses), stress reduction (breath control and meditation) and positive lifestyle changes (diet, smoking and alcohol).

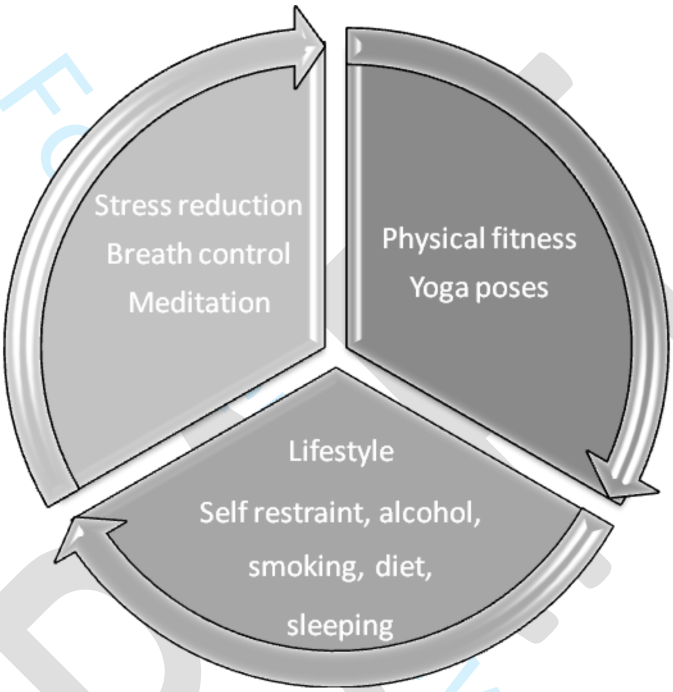


Each class will be approximately 1 hour and 15 minutes and in order to address the balance shown in the above diagram it will consist of the following parts:

- Yogic poses – approx 25 mins
- Breathing exercises and meditation – approx 25 mins
- Education and discussion – approx 25 mins

## Yoga Full Description

### *Part 1: Initial relaxation and warm-up – 10 minutes*

**Rationale:**


For peer review only - http://bmjopen.bmj.com/site/about/guidelines.xhtml

The initial relaxation and warm-up should be 10 - 15 minutes in duration. Gradual increase in intensity triggers three mechanisms which increase coronary blood flow to match the increased myocardial demand. As a result the ischaemic threshold is extended and the risk of angina and the risk of arrhythmias is reduced.

Due to older average age of this group (compared with mainstream) a gradual progression of range of motion exercises is prescribed.

The short preparatory stretches are included to prepare the muscles for the range of movement involved in Asanas to reduce risk of injury and encourage good balance and alignments. Since incorporating a static stretching will result in fall in heart rate, stretches will be intersperse with some dynamic movement (walking on the spot) designed to maintain the elevated heart rate.

## Initial relaxation

1. Emphasise mood, breathing, and relaxation

2. Set the mood for the class. Explain what yoga is, how can help, how should be practiced. Concentration, breathing and relaxation in all yoga practice should be explained and repeated in each class. Put participants in a relaxed state emphasising body position, breathing and relaxation. Use voice to relax them.

3. Begin by finding a comfortable position standing position with your feet hip or shoulder-width apart. You can change positions any time during the relaxation exercises to make yourself more comfortable as needed. Start from breathing. Breathe in slowly and deeply through your nose. Continue to breathe slowly and gently. Allow your breathing to relax you.

The next relaxation exercise focuses on relaxing the muscles of your body.

1. Start with the large muscles of your legs. Tighten all the muscles of your legs. Hold it for a few moments and now relax. Let all the tension go.

2. Now focus on the muscles in your arms. Tighten your shoulders, upper arms, lower arms, and hands. Squeeze your hands into tight fists. Tense the muscles in your arms and hands as tightly as you can. Hold it for a few moments and release. Allow the muscles in your arms to relax completely.

3. Focus again on your breathing. Slow, even, regular breaths. Continue to breathe slowly and rhythmically.

4. Now focus on the muscles of your buttocks. Tighten these muscles as much as you can. Hold this tension and release. Relax your muscles.

5. Tighten the muscles of your back now. Feel your back tightening, pulling your shoulders back and tensing the muscles along your spine. Arch your back slightly as you tighten these muscles. Hold and relax. Let all the tension go. Feel your back comfortably relaxing into a good and healthy posture.

6. Turn your attention now to the muscles of your chest and stomach. Tighten and tense these muscles and release. Relax the muscles of your trunk.

7. Finally, tighten the muscles of your face. Scrunch your eyes shut tightly, wrinkle your nose, and tighten your cheeks and chin. Hold this tension in your face and relax. Release all the tension. Feel how relaxed your face is.

Notice all of the muscles in your body, notice how relaxed your muscles fee l. Allow any last bits of tension to drain away. Enjoy the relaxation you are experiencing. Notice your calm breathing and your relaxed muscles. Enjoy the relaxation for a few moments.



For peer review only - http://bmjopen.bmj.com/site/about/guidelines.xhtml

When you are ready to return to your usual level of alertness and awareness, slowly begin to re-awaken your body. Wiggle your toes and fingers. Swing your arms gently. Shrug your shoulders.

## Warm-up

1. Now take a few steps on the spot and stand with your feet shoulder-width apart (set position), inhale and bend your knees and do mini-squat, exhale and come back to your set position. Repeat 4 times.

2. Take another few steps on the spot and come back to your set position. Inhale, roll your shoulders up, exhale bring them back and down. Repeat 4 times. During the last repetition hold your shoulders up for 4 to 8 seconds.

3. Take another few steps on the spot and come back to your set position. Inhale, roll your shoulders up, exhale bring them forward and down. Repeat 4 times. During the last repetition hold your shoulders up for 4 to 8 seconds.

4. Take another few steps on the spot and come back to your set position. Inhale, raise your arms to the side (shoulder height), exhale bring them down. Repeat 4 times. During the last repetition hold your arms up for 4 to 8 seconds.

5. Take another few steps on the spot and come back to your set position. Inhale, raise your arms to the front (shoulder height), exhale bring them down. Repeat 2 times. As above add heel raises at the same time if comfortable. Hold the last repetition for 4 to 8 seconds.

6. Take another few steps on the spot and come back to your set position. Inhale, raise your arms to the side (shoulder height), exhale and twist your trunk to the right. Inhale and come back to the centre, exhale and twist your trunk to the left. Repeat 2 times on each side. Hold the last repetition for 4 to 8 seconds.

7. Relax your arms and take another few steps on the spot and come back to set position. Inhale, raise your arms to the side (shoulder height) and look up. Exhale, bring your right arm crossover the chest to the left arm. Inhale, bring your right arm to the side. Exhale, bring your left arm crossover the chest to the right arm. Inhale, bring your left arm to the side. Repeat 2 times on each side. Hold the last repetition for 4 to 8 seconds.

8. Take another few steps on the spot and come back to your set position. Inhale and laterally bend your trunk to the right, exhale and come back to the centre. . Inhale and laterally bend your trunk to the left, exhale and come back to the centre. Repeat 2 times on each side. Hold the last repetition for 4 to 8 seconds.



For peer review only - http://bmjopen.bmj.com/site/about/guidelines.xhtml

Imperial College London

# *Part II: Asanas - 20 minutes*

**Rationale:**
Gentle progression from high to low positions is prescribed to avoid rapid changes of body positions in order to decrease the risk of arrhythmias or orthostatic hypotension in some individuals. Various 'chest opener' Asanas are prescribed to correct the round shouldered posture adopted by the population in response to discomfort in the area of the sterna incision.

## Standing poses (High positions)

### Mountain pose –Tad asana

1. Come to stand with the big toes touching.

2. Lift up all your toes and let them fan out, then drop them down creating a wide solid base. You can separate your heels slightly if your ankles are knocking together uncomfortably.

3. Bring your weight evenly onto all four corners of both feet.

4. Let the feet and the calves root down into the floor.

5. Engage the quadriceps and draw them upward, causing your knee caps to rise.

6. Rotate both thighs inward, creating a widening of the sit bones, and tuck your tailbone in between the sit bones.

7. Tone the belly, drawing it in slightly.

8. Widen the collar bones and make sure the shoulders are parallel to the pelvis.

9. The neck is long, the crown of the head rises toward the ceiling, and the shoulder blades slide down the back.

10. Hold for 5-10 breaths.

### Raised Hands Pose - Urdhva Hastasana

1. From Tadasana, bring your arms out to the side and up.

2. Press the palms together, keep the arms straight and take the gaze up toward your thumbs.

3. Slide the shoulder blades down the back.

4. Maintain your alignment.

5. Hold for 5-10 breaths.

### Awkward Chair Pose - Utkatasana

1. From  Tadasana bend the knees until the thighs are almost parallel to the floor.

2. Keep the butt low.



For peer review only - http://bmjopen.bmj.com/site/about/guidelines.xhtml

Imperial College London

3. Bring the arms up towards the ceiling.

4. Bring a slight back bend into the upper back.

5. Hold for 5-10 breaths.

**Beginners**: Work on bring the thighs closer and closer to parallel to the floor.

**Advanced**: Try this variation: Bring the hands into a prayer position at the heart. Twist to the right side, bringing the left elbow outside the right knee. Stay low in the pose and keep the knees pressing together. Come back to centre and then do the left side.

**Triangle Pose – Trikonasana**

1. From Tadasana take a big step backwards with your left leg.

2. Pivot on the ball of the left foot and drop the left heel onto the floor with the toes turned out about 45 degrees from the heel.

3. Bring the arms out to the side.

4. Slide the shoulder blades down the back.

5. Begin to reach the right arm forward, drawing the right thigh upwards and tucking the hip as you come forward.

6. Drop the right hand down to your shin or ankle, or if you are able, onto the floor inside or outside the right foot. Do whichever one feels most comfortable,

7. The left shoulder stacks on top of the right one as you open the chest reaching the left fingertips upwards while keeping the left shoulder rooted in the socket.

8. Take your gaze up towards the left fingertips.

9. Draw the right thigh muscle upwards, deepening the right hip crease.

10. Slightly bend the right knee.

12. Hold for 5-10 breaths

13. Repeat on the left side.

**Beginners**: Bring the right hand higher up on your leg or use a block on the floor to rest your hand on. It is more important to keep the right leg straight than to bring the right hand to the floor. Do not rest the hand directly on the knee, though, as this creates too much pressure on the knee.

**Advanced**: Line up the right heel with the arch of the left foot. For a variation, try dropping the left arm over the left ear so it comes parallel to the floor, while keeping the shoulder rooting into the socket.

**Tree Pose - Vrksasana**

1. Come to stand in Tadasana.

2. Feel your weight equally on all four corners of both feet.


For peer review only - http://bmjopen.bmj.com/site/about/guidelines.xhtml
DRAFT For peer review only

3. Begin to shift the weight over to the right foot, lifting the left foot off the floor.

4. Bend the left knee, bringing the sole of the left foot high onto the inner right thigh.

5. Press the foot into the thigh and the thigh back into the foot.

6. Try not to let the right hip jut out. Keep both hips squared towards the front.

7. Focus on something that doesn't move to help you keep your balance.

8. Hold for 5-10 breaths.

9. Repeat the move while standing on the left foot.

**Beginners**: If you cannot bring the left foot high inside the right thigh, bring it lower on the right leg - but be careful to avoid placing the left foot directly on the right knee.

Use the wall for balance if necessary.

**Advanced**: Bring the arms up towards the ceiling with the palms touching. Open the arms out to side.

Try closing the eyes and see if you can stay balanced.

## Kneeling & Sitting poses (Medium positions)

### Cat - Cow Stretch - Chakravakasana

1. Start on all fours, bringing the wrists underneath the shoulders and the knees underneath the hips.

2. Think of the spine as a straight line connecting the shoulders to the hips. Try visualizing the line extending forward through the crown of the head and backwards through the tail bone.

3. Keep the neck the natural extension of the spine.

On an inhale:

1. Curl the toes under.

2. Drop the belly.

3. Take the gaze up toward the ceiling.

4. Let the movement in the spine start from the tailbone, so that that neck is the last part to move.

On the next exhale:

1. Release the tops of the feet to the floor.

2. Round the spine.

3. Drop the head.



For peer review only - http://bmjopen.bmj.com/site/about/guidelines.xhtml

Imperial College London

4. Take the gaze to the navel.

5. Repeat the Cat - Cow Stretch on each inhale and exhale, matching the movement to your own breath.

6. Continue for 5-10 breaths, moving the whole spine. After your final exhale, come back to a neutral spine.

## Hands and Knees Balance

1. Come on to all fours with the wrists underneath the shoulders and the knees underneath the hips.

2. Extend the right leg to the back of your mat and flex the foot.

3. Lift the right leg up to hip-level, keeping the hips squared towards the floor and the foot flexed.

4. Lift the left arm up to shoulder level.

5. Balance on the left knee and right hand, keeping the spine neutral and the neck long.

5. Stay 5-10 breaths before lowering the lifted hand and knee and doing the other side.

**Beginners**: Take care not to let the spine collapse while you are balancing.

**Advanced**: Bend the knee of the lifted leg. Reach around with the lifted arm and hold on to the inside of the lifted foot.

## Staff Pose - Dandasana

1. Sit with the legs outstretched straight in front.

2. Engage the thigh muscles and flex the feet. The heels may come up off the floor.

3. Make your spine long.

4. Stack the shoulders directly on top of the hips.

5. Hold for 5-10 breaths.

**Beginners**: Put padding under your sit bones, if necessary.

**Advanced**: This pose looks easy, but if you are really working the thighs, you can break a sweat.

## Seated Forward Bend - Paschimottanasana

1. From Dandasana bring the arms straight out to the sides and up over your head.

2. Inhale and draw the spine up long.

3. As you exhale, begin to come forward, hinging at the hips.

4. On each inhale, extend the spine, and on each exhale, come a bit farther into the forward bend.

5. Keep the neck at the natural extension of the spine.

6. Do not round the back.


For peer review only - http://bmjopen.bmj.com/site/about/guidelines.xhtml

Imperial College London

7. Take hold of the ankles or shins, whichever you can reach.

8. Hold for 5-10 breaths.

**Beginners**: Put padding under the sit bones if necessary. Imagine the belly coming to rest on your thighs, rather than the nose coming to the knees - this will help you keep the spine long instead of curving over.

**Advanced**: If you can easily grab the soles of your feet, try taking a block in front of the feet and holding that instead.

**Crab pose- Catuspadapitham**

**1.** From Dandasana, bend the knees bringing the feet flat on the floor hip width apart. Keep the arms behind your hips with the fingers pointed away from your body.

**2.** Lean back into the arms and slowly inhale and lift the hips up towards the ceiling. Make sure the toes and knees are pointing straight ahead. Look straight ahead, up at the ceiling or carefully drop the head back.

**3.** Press into the feet, squeezing the thighs and buttocks and engaging Mula Bandha to lift the hips high. Press into the hands and draw the shoulder blades towards each other to lift up high through the sternum.

**4.** Breathe and hold for 2-6 breaths.

**5.** To release: slowly exhale the hips back down to the floor.

**Beginners**: If there is pain or discomfort in the wrists, point the fingers in the opposite direction or make fists with the hands.

**Advanced**: Inhale one leg up towards the ceiling at a time, pressing out through the heel.

**Half Lord of the Fishes Pose (Half Spinal Twist) - Ardha Matsyendrasana**

1. From Dandasana, bend your left knee and bring the sole of your left foot to the floor on the outside of the right thigh.

2. Bend the right knee, and tuck the right foot in near the left buttock.

3. Inhale and bring the right arm up near your right ear.

4. Exhale and twist the to the left, bringing the right elbow to the outside the of left knee and the left palm to the floor, just behind your sit bones.

5. Look out over the left shoulder, but don’t overturn the neck -- the twist originates in the belly, not the neck.

6. On each inhale, draw the spine long, and on each exhale, twist a little deeper.

7. Be sure to keep the sole of your left foot flat on the floor.

8. Hold for 5-10 breaths.

9. When you release the pose, take a slight counter twist to the opposite direction.

10. Release the legs and switch their position as you prepare to twist to the other side.

DRAFT For peer review only

Imperial College London

**Beginners**: You may want to sit on some padding if you are uncomfortable. If you cannot bend it into the ideal position, you may also keep the right leg extended.

**Advanced**: Come into a bind with the arms. Thread the right arm back underneath the left knee. Reach the left arm behind your back, and clasp the left wrist with your right hand.

## Easy Pose - Sukhasana

1. Arrange padding under your sit bones so that your hips come above your knees.

2. Come to sit in a comfortable, cross-legged position.

3. Bring one heel in towards your groin. The other foot may rest on the floor in front of you or you may bring it into your lap.

4. Root your seat down as your spine grows long. Stack the shoulders over the hips and slide the shoulder blades down your back. The crown of your head rises towards the ceiling.

## Neck Exercises

1. In Sukhasana, with your back straight and your chest erect. Slowly bring your head forwards towards the chest to give the back of your neck a good stretch.

2. After a few breaths slowly lift your head and extend your neck back and bring your head to neutral position.

3. Lower your right ear close to your right shoulder, then repeat on the other side. Keep both shoulders level throughout. Repeat the exercise 5 times.

4. Turn your head to the right side. Contract the muscles on the right side of your neck and feel the stretch on the left side. Repeat on the opposite side. Repeat the exercise 5 times.

# Lying down poses (Low positions)

## Single Leg Lift

1. Lie flat on your back with your legs together, arm next to your body, and palms face down.

2. Inhale and raise your left leg, keeping your knee straight, toes towards your head.

3. Exhale and lower your leg to the starting position.

4. Repeat up to 5 times on the each side.

## Head to Knee Raise

1. Start from Single Leg Left Step 2.

2. With an exhalation, bend your left leg and clasp your hands around your knee.

3. With an inhalation, lift your head and try to bring your forehead against your left knee.

4. With an exhalation, lower your head, arms, and leg.

5. Repeat on the opposite side.


For peer review only - http://bmjopen.bmj.com/site/about/guidelines.xhtml
DRAFT
For peer review only

Imperial College London

**Beginners**: Keep your head on the floor.

**Advanced**: Progress to Deep Stretch Single Leg Lift.

1. Start from Single Leg Lift Step 2.

2. With an exhalation, take hold of your leg with both hands, lift your back off the mat and try to bring your chest and head close to the raised leg.

## Happy Baby Pose - Ananda Balasana

1. Come to lie on the back.

2. Bend the knees into the chest.

3. Open the knees, bringing them towards the armpits.

4. Stack each ankle directly over the knee, so that the shins are perpendicular to the floor.

5. Flex the feet.

6. Hold the outer edges of the feet at you draw the knees towards the floor.

This pose is appropriate for both beginners and advanced students.

## Corpse Pose - Savasana

1. Come to lie down on the back with your arms and feet apart and your eyes closed.

2. Let the feet fall out to either side.

3. Turn the palms to face upwards.

4. Relax the whole body, including the face. Let the body feel heavy.

## *Part III: Cool Down: Breathing exercises and final relaxation - 20 minutes*

**Rationale:**
Twenty minutes Cool Down/Breathing/Relaxation period is prescribed to reduce risk of hypotension or arrhythmias and to allow the hart rate to return to pre-exercise rates.

**Deep Abdominal Breathing and Full Yogic Breath Practice-5 minutes**

### Deep Abdominal Breathing

1. In Corpse Pose place both hands on your abdomen with your fingers apart.

2. As you inhale, feel your abdomen and hands rising.

3. As you exhale, feel your abdomen and hands sinking.

4. Try to breathe rhythmically, with an inhalation lasting 3-5 seconds and exhalation of the same length.



For peer review only - http://bmjopen.bmj.com/site/about/guidelines.xhtml

Imperial College London

## Full Yogic Breath

1. In Corpse Pose place one hand on your chest and the other on your abdomen.

2. As you inhale, gradually expand the abdomen, then rise and open the rib cage, and finally lift the collar bones.

3. Begin the exhalation by relaxing the abdomen, then lower the rib cage, and finally slightly contract the abdomen to actively empty the lungs.

Alternate Nostril Breathing (Anuloma Viloma)- 5 minutes

Single Nostril Breathing

In Easy Pose, place your right hand in front of your face in Vishnu Mudra*. Close your right nostril with your thumb. Inhale for three seconds and exhale for six seconds through your left nostril. This is one round. Practice ten rounds. Repeat on the other nostril: close your left nostril with your ring finger, and inhale and exhale through your right nostril.

**Beginners**: Gradually increase the ratio of the inhalation to the exhalation lengthening to 4:8, 5:10 and 6:12.
**Advanced:** Progress to Simple Alternate Nostril Breathing

Simple Alternate Nostril Breathing

In Easy Pose close your right nostril with your thumb, inhale through the left nostril for three seconds, close your left nostril with your ring finger, open your right nostril and exhale through it for six seconds. Inhale through your right nostril for three seconds, then exhale through your left nostril for six seconds. Practice for ten rounds. Gradually increase the inhalation: exhalation ratio to 4:8, 5:10 and 6:12.

*Vishnu Mudra: Hold your right hand with the palm facing you and fold the first and second fingers into the palm. Try to keep your thumb and ring fingers straight.

## Final Relaxation

1. Inhale and lift your right leg a few inches off the mat. Tense your leg, then exhale and allow your leg to drop. Repeat with the left leg.

2. Inhale and lift both arms a few inches off the mat. Clench your fists, tense your arms, then exhale and allow your arms to drop to the mat.

3. Inhale and lift your hips and buttocks off the mat. Tense your buttocks and then exhale and release.

4. Inhale and lift your chest off the mat. Tense your shoulder blades, then exhale and release.

5. Inhale and pull your shoulders towards your ears. Exhale and release your shoulders.

6. Inhale and squeeze the muscles of your face tightly together. Exhale and release.

7. Inhale, open your mouth, stick your tongue out and look to your forehead. Exhale and release.

8. With an inhalation, slowly roll your head to one side; with an exhalation, roll it to the other side. End by bringing your head back to centre.



DRAFT
For peer review only
For peer review only - http://bmjopen.bmj.com/site/about/guidelines.xhtml

Imperial College London

Take a few slow rhythmic breaths using your abdomen, then follow this exercise in auto suggestion.

I'm relaxing my feet.....My feet are relaxed.....

I'm relaxing my ankles.....My ankles are relaxed.....

I'm relaxing my calves.....My calves are relaxed.....

I'm relaxing my knees.....My knees are relaxed.....

I'm relaxing my thighs.....My thighs are relaxed.....

I'm relaxing my hips and buttocks.....My hips and buttocks are relaxed.....

I'm relaxing my abdomen and chest.....My abdomen and chest are relaxed.....

I'm relaxing my lower and middle back.....My lower and middle back are relaxed.....

I'm relaxing my shoulders and neck.....My shoulders and neck are relaxed.....

I'm relaxing my hands and fingers.....My hands and fingers are relaxed.....

I'm relaxing my arms.....My arms are relaxed.....

I'm relaxing my mouth and eyes.....My mouth and eyes are relaxed.....

I'm relaxing my facial muscles and scalp.....My facial muscles and scalp are relaxed.....

I'm relaxing my internal organs: my kidneys, my livers, my intestines, my bladder, my pancreas, my stomach, my heart, my lungs and my brain.....My internal organs: my kidneys, my livers, my intestines, my bladder, my pancreas, my stomach, my heart, my lungs and my brain are relaxed.....

Continue abdominal breathing and relaxation. Visualise a calm lake, unruffled by waves. Picture the still water resting on your inner self, which is timeless and unchanging. Continue for a few more minutes.

Then take a few deep breaths, slowly move your legs and arms, and give your whole body a good stretch. Finally bring yourself slowly to sitting cross-legged position. Deeply inhale and exhale. Inhale and bring your hands into a prayer position and as you exhale bow your head thanking everyone for the practice.

## Alternative relaxation

In Savasana pose. For the next few moments, focus on calming your mind by focusing on your breathing. Allow you breathing to centre and relax you. Breathe in.... and out.

In..... out.....

In.... Out.....

Continue to breathe slowly and peacefully as you allow the tension to start to leave your body.

Release the areas of tension, feeling your muscles relax and become more comfortable with each breath.

Continue to let your breathing relax you....

DRAFT
For peer review only


For peer review only - http://bmjopen.bmj.com/site/about/guidelines.xhtml

Imperial College London

Breathe in....2...3...4.... hold....2.....3...... out...2...3....4...... 5

again....2.....3....4....hold....2....3.... out...2...3....4.... 5

Continue to breathe slowly, gently, comfortably.....

Let the rate of your breathing become gradually slower as your body relaxes.

Now begin to create a picture in your mind of a place where you can completely relax. Imagine what this place needs to be like in order for you to feel calm and relaxed.

Start with the physical layout of the place you are imagining..... where is this peaceful place? You might envision somewhere outdoors.... or indoors..... it may be a small place or large one..... create an image of this place.

(pause)

Now picture some more details about your peaceful place. Who is in this place? Are you alone? Or perhaps you are with someone else? Are there other people present? Animals? Birds? Imagine who is at your place, whether it is you only, or if you have company.

(pause)

Imagine even more detail about your surroundings. Focus now on the relaxing sounds around you in your peaceful place.

Now imagine any tastes and smells your place has to offer.

Imagine the sensations of touch... including the temperature, any breeze that may be present, the surface you are on.... imagine the details of this calming place in your mind.

Focus now on the sights of your place - colours, shapes.... objects.... plants..... water..... all of the beautiful things that make your place enjoyable.

To add further detail to this relaxing scene, imagine yourself there. What would you be doing in this calming place? Perhaps you are just sitting, enjoying this place, relaxing. Maybe you imagine walking around.... or doing any other variety of activities.

Picture yourself in this peaceful place. Imagine a feeling of calm..... of peace..... a place where you have no worries, cares, or concerns.... a place where you can simply rejuvenate, relax, and enjoy just being.

(pause)

Enjoy your peaceful place for a few moments more. Memorize the sights, sounds, and sensations around you. Know that you can return to this place in your mind whenever you need a break. You can take a mental vacation to allow yourself to relax and regroup before returning to your regular roles.

In these last few moments of relaxation, create a picture in your mind that you will return to the next time you need a quick relaxation break. Picture yourself in your peaceful place. This moment you are imagining now, you can picture again the next time you need to relax.

When you are ready to return to your day, file away the imaginary place in your mind, waiting for you the next time you need it.

Turn your attention back to the present. Notice your surroundings as your body and mind return to their usual level of alertness and wakefulness.


For peer review only - http://bmjopen.bmj.com/site/about/guidelines.xhtml
For peer review only
DRAFT

Imperial College London

Keep with you the feeling of calm from your peaceful place as you return to your everyday life.

## *Part IV: Supervision of participants post-exercise and education - 20 minutes*

**Rationale:**
Because of an increased risk of arrhythmia and hypotension following exercise, a period of 15-20 minutes supervision is adopted before participants go home. This time will be used for education sessions.

### *Proper Exercise*

*Yoga versus physical culture*

Aim:

- For the group to understand the importance of taking up physical activity in cardiac rehabilitation
- For the participants to understand that yoga improves not only flexibility but strength, balance and cardiovascular function

Brainstorm question: What are the differences between yoga and physical culture?

Explore the answers and highlight the following:

- Yoga regards the body as a vehicle for the soul in its journey towards perfection
- Yoga promotes gentle movement whereas physical culture emphasises violent muscle movements
- Muscle development does not necessarily mean a healthy body
- Health is a state wherein all organs function perfectly under intelligent control of mind
- Asanas are designed to develop not only the body but also broaden the mental faculties and spiritual capacities
- The body is as young as it is flexible, so yoga postures primary focus on the health of the spine, its strength and flexibility
- Asanas work on the internal machinery of the body, the glands and organs as well as the muscles
- Hand in hand with the practice of yoga postures we practise deep breathing and concentration of the mind

### *Proper Breathing*

*Yogic breathing*

Observe your breathing for a while. How would you describe your breath?

Ask participants to share their observation and then highlight the following:

- Yoga philosophy claims we are allotted a certain number of breaths per lifetime. How we choose to illustrate that then becomes our practice of longevity. Breathing is the first thing we do when we are born and the last thing we do when we die. Practice observing your breath as often as possible.
- Most people use only a fraction of their potential lung capacity when breathing


For peer review only - http://bmjopen.bmj.com/site/about/guidelines.xhtml

Imperial College London

- There are three types of breathing: clavicular, intercostal and deep abdominal.
- A full yogic breath combines all three types of breathing
- Yogic breathing exercises are called pranayama which means to control the prana-subtle energy. Pranayama begins by controlling the motion of the lungs, by which the prana is control.
- Yogic breathing exercises might be very useful in process of quitting smoking

## *Proper Relaxation-Savasana*

Brainstorm question: How do we relax?

Explore the answers and highlight the following:

- When the body and mind and the mind are constantly overworked, their natural efficiency diminishes
- Modern social life and entertainment make it difficult for people to relax by over stimulating the nervous system
- By learning to relax we learn to economise the energy produced by our body as well as regulate and balance the work of the body and mind
- In ordered to achieve perfect relaxation, three methods are used for yogis: physical, mental and spiritual relaxation
- The relaxation position is known as Savasana, the 'Corpse pose'

## *Proper Diet-Vegetarian – Part I*

*'You are what you eat'*

Discuss and highlight the following:

- Proper yogic diet is lactovegetarian one based on simple, natural and wholesome food
- According to yogic philosophy all of Nature, including our diet, is categorised into three qualities (Gunas): sattvic (pure), rajasic (overstimulating) and tamasic (putrified)
- Sattvic food increases vitality, energy, vigor, health and joy
- Food should be as fresh and natural as possible, preferably organically grown
- Sattvic food include:

  **Grains:** corn, barley, wheat, unpolished rice, oat, millet and quinoa.
  Grains supply necessary carbohydrates, the main source of energy for the body, and they also contain about half the amino acids that are needed to form protein.
  **Protein foods:** legumes, nuts and seeds

  **Fruits:** both fresh and dried, as well as pure fruit juices



For peer review only - http://bmjopen.bmj.com/site/about/guidelines.xhtml

For peer review only
DRAFT

**Vegetables:** they contain minerals, vitamins and fibre. There are best eaten raw or cooked as lightly as possible

**Herbs:** for seasoning and herbal tea

**Natural sweeteners:** honey, molasses, maple syrup, and apple juice concentrate. White sugar is best avoided in a healthy diet.

**Dairy products:** milk, butter, cheese and yogurt

## *Proper Diet-Vegetarian – Part II*

*Guidelines for healthy eating*

Recap from the previous 'Proper diet' session and then highlight the following:

- Always respect your food and maintain a peaceful attitude during meals
- Do not eat when you are angry
- Do not eat food that is too hot or too cold, as this will upset your stomach
- Do not force yourself to eat anything you do not like, but also do not only eat foods that you like the most
- Abandon too many mixtures or combination of foods as they are difficult to digest
- Try to refrain from drinking during meals as this will dilute the gastric juice
- Eat slowly and savour your food
- Eat moderately, do not overload your stomach
- Try to eat at fixed times and try to refrain from eating between meals
- Try not to eat large meals at night
- Take some lemon and honey in the morning for health and energy and to purify the blood
- Do not practise asanas immediately after eating, nor when you are hungry
- Try sitting in Vajra Asana (sitting on the heels with knees and feet together) for 10 minutes after a meal to assist digestion

## *Positive Thinking and Meditation – Part I*

*Practical approach to meditation*

Discuss and highlight the following:

- Before we can learn to meditate we have to be able to concentrate
- Concentration means attending fully to one thought or object for a substantial length of time



For peer review only - http://bmjopen.bmj.com/site/about/guidelines.xhtml

Imperial College London

- Concentration exercises energise the mind, boosting efficiency at work and in the other tasks, while building will-power and the ability to influence other people positively

**Exercise: Listen to a sound**
Now listen carefully to the ticking of a watch. When your mind wanders, bring it back to the sound. How long can you concentrate on that sound?
*Exercises to practice at home-leaflet to be given*

**Lose yourself in a book**
Read two or three pages of a book, giving them your full attention. Then test your concentration by stopping at the end of a page. How much do you remember of the story? Can you classify, group or compare the facts you have been reading about?

**Contemplate nature**
During the day, concentrate on the sky. Feel your mind expand as you reflect on its vast expanse. At night, concentrate on the moon or stars. By the sea, focus on waves. Or shift your gaze between objects near and far, such as a nearby tree and a distant mountain.

**Focus on a flower**
Sit comfortably with your eyes closed.  Imagine a garden with many flowers. Gradually, bring your attention to a single flower. Visualise its colour and explore its other qualities, such as texture, shape, and scent. Concentrate on the flower's qualities for as long as possible.

## *Positive Thinking and Meditation – Part II*

*Practical approach to meditation*

Ask participants if they had chance to practice any of the concentration exercises then discuss and highlight:

- Meditation is a state of relaxed awareness

- The more care and attention you give to your preparation for meditation, the more positive the results will be

- Get the atmosphere right for meditation:

  **Place**: It is best to separate one portion of a room to use for your practice. Keep it clean and tidy, and place a candle or spiritually uplifting picture there. Burning incense can also help to create a meditative mood.

  **Time**: The best times for meditation are at dawn and dusk. Alternatively, find a time when you are free from daily activities and your mind can be calm.

  **Habit**: Practise every day at the same time. As your subconscious mind gets accustomed to the regularity, you will find it easier to settle and focus.

  **Sitting position**: Sit on the floor to meditate, in position that you can maintain comfortably, keeping your spine and neck straight but not tense. A simple, crossed-legged pose makes a firm base. Sitting on the cushion helps the thighs to relax and bring brings knees closer to the ground.

  If you cannot sit on the floor easily, sit on a comfortable chair with your ankles crossed.

DRAFT
For peer review only

**Breathing**: Once you are sitting comfortably, relax your body as much as possible. Broaden your chest and lift your rib cage to encourage abdominal breathing. Then inhale and exhale rhythmically for about 3 seconds each, gradually slowing your breath down.

## *Making Positive Changes in Your Life*

Topic to reflect: Think about one change you would like to make (if any) to make your lifestyle healthier.

Ask if anyone would like to share their idea then suggest the following changes to make within first two months of practising yoga.

- **Proper exercise:** Try to practise asanas regularly
- **Proper breathing:** Practice deep abdominal breathing
- **Proper relaxation:** Learn Corpse pose and try to relax for 15 minutes daily
- **Reduce negative dietary habits:** Cut down or eliminate meat and cut down on fried food
- **Reinforcing positive dietary habits:** Drink 4 to 5 glasses of water and eat one raw salad daily
- **Eradicating negative habits:** If you smoke replace it with abdominal breathing
- **Concentration exercises:** Practice listening and hearing what others are saying
- **Positive thinking:** Refrain from using abusive language and try to spend time with people who have a positive outlook on life
- **Meditation:** Sit silently for at least 20 minutes daily with the mind focused on breath
- **Study:** Read something of inspiration daily

DRAFT


For peer review only - http://bmjopen.bmj.com/site/about/guidelines.xhtml

Imperial College London

# Appendix 1

## *Policy for cardiac rehabilitation in Ealing*

### INTRODUCTION

Cardiac disease is the leading cause of death in United Kingdom and is the leading cause of hospitalisation for both men and women. Cardiac rehabilitation programmes are recognised as a way to enhance recovery following acute cardiac events and encourage behaviour aimed at the secondary prevention of coronary artery disease. The key elements of cardiac rehabilitation are contained in the definition produced by the Scottish Intercollegiate Guidelines Network (SIGN): Cardiac rehabilitation is the process by which patients with cardiac disease, in partnership with a multidisciplinary team of health professionals, are encouraged and supported to achieve and maintain optimal physical and psychological health.

Cardiac rehabilitation is defined by the World Health Organisation as:
*".. the sum of activities required to influence favourably the underlying cause of the disease, as well as the best possible, physical, mental and social conditions, so that they (people) may, by their own efforts preserve or resume when lost, as normal place as possible in the community. Rehabilitation cannot be regarded as an isolated form or stage of therapy but must be integrated within secondary prevention services of which it forms only one facet".*

The provision of skilled help, support and supervision that is tailored to individual patients can: a) help people understand their illness and its treatment; b) provide psychological and emotional support; c) improve people's success in making beneficial lifestyle changes; and d) help people make the transition back to a full and as normal life as possible. (NSF: cardiac rehabilitation, 2007)

The 2007 NICE guidelines on "secondary prevention for patients following a myocardial infarction" state that cardiac rehabilitation should be equally accessible and relevant to all patients after an MI, particularly people from groups that are less likely to access this service. These include people from black and minority ethnic groups, older people, people from lower socioeconomic groups, women, people from rural communities and people with mental and physical health co morbidities.

The British Association of Cardiac Rehabilitation (BACR) standards 2007 defined the core components of cardiac rehabilitation as lifestyle (physical activity and exercise, diet and weight management, smoking cessation), education, risk factor management, psychosocial, cardio protective drug therapy and implantable devices, and long-term management strategies (4).

Four phases of cardiac rehabilitation were defined by the BACR and endorsed by the National Service Framework (2007) for CHD in England and Wales and SIGN for Scotland (2002). Each phase represents a different component of the journey of care. Phase 1 is generally concerned with the in-patient episode with Phases 2-4 following the patient from early discharge to long-term maintenance.

According to the NSF goal, every hospital should ensure that:
more than 85% of people discharged from hospital with a primary diagnosis of acute myocardial infarction or after coronary revascularisation are offered cardiac rehabilitation and one year after discharge at least 50% of people are non-smokers, exercise regularly and have a BMI <30 kg/m$^2$; these should be demonstrated by clinical audit data no more than 12 months old. Trusts should agree, implement and audit a detailed plan and protocol for identifying, treating and following up their patients who may benefit from cardiac rehabilitation.

### THE CARDIAC REHABILIATION PROGRAMME IN EALING HOSPITAL NHS TRUST

The aim of the comprehensive cardiac rehabilitation programme is to reduce the risk of subsequent cardiac problems and to promote the return to a full and normal life. The provision of a cardiac rehabilitation service for all eligible patients is clearly desirable for health and economic reasons.


For peer review only - http://bmjopen.bmj.com/site/about/guidelines.xhtml

Imperial College London

Comprehensive help with lifestyle modification involving education and psychological input as well as exercise training can reduce mortality by 20-25% over 3 years. ( Oldridge et al 1988; O'Connor et al 1989)

**1. TARGET CLIENT GROUPS**

Patients admitted with or who have undergone the following will be eligible for the programme.

- NSTEMI
- STEMI
- Acute Coronary Syndrome
- Revascularisation
- CABG
- Valve surgery
- Heart Failure

All the above patients admitted to Ealing Hospitals will be offered a choice as to where their cardiac rehabilitation will take place.

**2. IDENTIFYING PATIENTS**

- Patients are identified through CCU/ITU, cardiology and general medical wards, cardiology out patients, and from waiting lists for revascularisation procedures.
- Referrals are accepted from other acute trusts (using North West London Cardiac Rehabilitation referral form); Ealing Hospital cardiac catheter laboratory, as well as the community referrals from GP's, Practice Nurses, Community Specialist Clinics.

**3. PHASE 1** (Before discharge from hospital)

Where possible the Cardiac Rehabilitation Specialist Nurse will visit the patient and his / her family during the hospital stay. The following will be carried out during this phase:
• assessment of physical, psychological and social needs for cardiac rehabilitation
• negotiation of a written individual plan for meeting these identified needs
• initial advice on lifestyle e.g. smoking cessation, physical activity (including sexual activity), diet, alcohol consumption, driving and employment
• review of prescription of effective medication and education about its use, benefits and harms
• involvement of family members and/or relevant informal carer(s)
• provision of information about cardiac support groups
• provision of locally relevant written information about cardiac rehabilitation

It is important to establish rapport and therapeutic relationship with every patient and involve family or/and carers from this early stage. This will increase the likelihood or patient's participation in consecutive phases of cardiac rehabilitation programme and reduce a risk of DNA incidences.

The “Edinburgh Heart Manual” for education, exercise and stress management components can be given to eligible patients at this stage. Social needs and preferences of patients will be identified and taken into account for a purpose of structuring of individually tailored cardiac rehabilitation programmes.

Guidelines for Phase 1 Cardiac Rehabilitation Service will be followed ( see Appendix 1\)

BACR Guidelines for Secondary Prevention will be followed. They are:

- Risk factors of each patient should be identified and managed accordingly.
- All patients who smoke should be offered structured anti-smoking advice and, if necessary, specific


For peer review only - http://bmjopen.bmj.com/site/about/guidelines.xhtml

treatment.

- All patients after acute myocardial infarction or coronary revascularisation should be treated, if necessary, with lipid lowering therapy (diet control, statin therapy, lifestyle changes to include regular exercises), antiplatelet therapy (with aspirin, dipyridamole or clopidogrel), beta blockers, ACE inhibitors, other secondary prevention measures (better control of diabetes, hypertension, body mass index (BMI)).
- All patients with heart failure should be given advice on fluid balance (daily weights, fluid intake, diuretic dosages), salt restriction, avoidance of ethanol consumption and smoking, influenza vaccination, and considered for therapy with ACE inhibitors, beta blockers, spironolactone, angiotensin receptor blockers for prognostic benefits.
- Patients with other conditions should receive appropriate advice and treatment as secondary prevention of their specific cardiac conditions (e.g. avoidance of caffeine and treatment with beta blockers in patients with cardiac arrhythmia).

**4. PHASE 2** (Early post-discharge period)

During early post discharge period, support to patients can be provided by home visiting where appropriate, telephone contact and by supervised use of the Heart Manual.
Patient will be sent an invite letter for the first outpatient appointment in cardiac rehabilitation clinic of Ealing Hospital within 2 -3 weeks after discharge from hospital. The time tables with dates of currently run educational sessions will be included with an invite letter.( see Appendix 2). Where possible, patients will be asked to have their blood tests done in GP practices prior to appointment with cardiac rehabilitation specialist nurse. Patients will also be asked to bring their medication list and any outpatient appointments' letters with them.
During the consultation in OPD clinic, the individual needs, expectations of cardiac rehabilitation programme and wishes will be explored. The suitability for exercise programme ( a component of comprehensive cardiac rehabilitation programme) can also be assessed at this point.

Following will be carried out:

- Provision of general advice about the cardiac condition(s) and complications that the patient has, including risk factor management, medication (what they are for, adjustment of doses and potential adverse reactions), presentation of further events and what actions to take; symptom control advise
- Patients’ misconceptions and undue fears or anxieties will be identified and addressed
- Patients will be advised on the stages towards resuming normal life (e.g. physical activity levels, sexual function, driving, flight, return to work, weight control, fluid balance, alcohol consumption).
- Advice will be provided to patients to address vocational, social, cultural, educational needs, and referral for occupational therapy assessment and management.
- Measurement of patient's body weight, calculation of body mass index (BMI) and central obesity (girth measurements)
- Review of fasting lipid profile +- advice/ appropriate referrals to GP/lipid clinic.
- Review of fasting blood glucose +- advice/ referral to diabetic specialist nurse/GP
- Dietary habit assessment and advice on healthy eating
- Blood Pressure will be measured and heart rate record
- If known to have diabetes a urine sample for microalbuminurea will be collected.
- Assessment of wounds and advice as necessary (post surgery patients)
- HADS +-quality of life (QoL) assessments will be carried out to estimate patients' health perceptions and to help detect patients with inappropriate levels of anxiety or depression, a small proportion of whom may need referral for specialist evaluation and treatment
- Advice how to stop smoking- for those who smoke+- referral to specialist services
- Review involvement with cardiac support groups
- Offer resuscitation training for family members
- Encouraging patients' immediate family members to engage in health improvement and lifestyle modification

“Client Feedback and Goal Planning Form” (see Appendix 3), as well as “Agreed Action Plan” (see Appendix 4) forms will be given to those who were not seen at Ealing Hospital during Phase 1. Patients will be encouraged to


For peer review only - http://bmjopen.bmj.com/site/about/guidelines.xhtml

participate in joint (with nurse specialist) care planning, goal setting, time-allocation for improvement and exploration of barriers to achievement of desirable results. This process is very important for achievement of lifestyle modification and behavioural change.
Patients will be encouraged to bring those forms to meetings with cardiac rehabilitation nurse, or/and to educational, or/and exercise programmes, so appropriate to the area of concern specialist will be able to answer the questions and give patient-tailored advice and recommendations.
Patients will be also given comprehensive information regarding diagnosis, procedures, practical advice and risk factor modification in written form. Patients will be issued with a wallet sized card that allows each patient to keep a record of his or her risk factors, including blood pressure, cholesterol and glucose, lifestyle modifications, dates of procedures and current medication.

Patients and their partners will be invited to enter an 6-12 week health promotion programme where patients will receive 1) on-going risk factor monitoring/ advice/support, 2) exercise sessions in a gym, led by cardiac physiologist or home-based exercise programme, 3) health education lectures (led by cardiac rehabilitation sister; pharmacist; dietician; clinical psychologist; cardiac physiologist), 4) relaxation sessions, 5) guidance and supervised use of “Edinburgh Heart Manual”.

Patients will be asked about their preferences on exercise programme, whether they would like to join exercise programme run in local Gym(s) or to have home based exercise programme ( e.g. using “Edinburgh Heart Manual” as guideline and/or using exercise plan prescribed by cardiac physiologist and/or using pedometers, etc).
Patients will be given information on forthcoming dates/ topics/ venues of educational programme. Patients themselves and their family members or carers will be encouraged to come to educational sessions together.
For patients whose English is limited, the interpreter services will be provided where possible. Patients will be encouraged to bring their English speaking relatives for a consultation. The information about each individual condition/treatment/recommendations can be ordered for patient from British Heart Foundation in a form of audio tape/video-tape/pocket size leaflets, etc
Those patients who will not respond to invitation letter to attend an OPD clinic will be contacted via a telephone. Rehabilitation staff will try to address the issues which might impede patient's decision about participation in a programme using individual approach. However, If a patient will state clearly that he/ she does not wish to participate, then patient's GP will be informed and patient will be discharged from CR service.

**4.1.Referrals:**

- Weight management and diet advice: All patients regardless of their cardiac condition will be referred to cardiac specialist dietician for cardio-protective diet advice and weight management programme if appropriate.
- Diabetes: Newly diagnosed and those patients with diabetes who are not well controlled will be referred to the community diabetes team.
- Erectile Dysfunction: patients experiencing sexual problems can be referred to the ED clinic at GSTT.
- Psychological Problems: Rehabilitation staff will do their best to identify and address cardiac misconceptions in patients with CHD in order to reduce possibility of anxiety or/and depression. Hospital anxiety and Depression Scale (HADS) will be used. Screening will take place at discharge (where possible), 6-12 weeks post MI or following a decision on surgical intervention; can be repeated every 3 months if necessary. Psychological interventions of cardiac rehabilitation programme, such as stress management, relaxation, goal setting, taking part in group exercise and education can relieve anxiety and mild depression. Patients who score persistently above 11 on the HAD scale can be considered for referral to a clinical psychologist for assessment/ interventions.

- **Health Education Talks** (please see appendix 2 for the current health education presentations)

Discussions will be given by a health care professional with specialist knowledge of the subject. This is an information giving session to increase patients knowledge.

The group discussion allows patients to explore the information given and how best to apply it to themselves and their families. These sessions are intentionally informal and encourage patients to recognise their own risk factors and develop strategies for change. The following topics are to be covered by the workshops:

1. Drugs for heart disease and how they work (presented by pharmacist)


For peer review only - http://bmjopen.bmj.com/site/about/guidelines.xhtml

Imperial College London

2. Managing Stress (by clinical psychologist)
3. Eating for a healthy heart (by cardiac specialist dietician)
4. Exercise and the benefits for your heart (cardiac exercise physiologist)
5. Risk factors and making lifestyle changes (by cardiac rehabilitation sister)
6. The heart and how it works (by cardiac rehabilitation sister)

**5. PHASE 3 (**Four weeks after an acute cardiac event / 4-6 weeks post surgery)

Structured exercise as a therapeutic intervention is central to cardiac rehabilitation. Exercise training should form a core element of cardiac rehabilitation programmes (SIGN,2002).
At this stage, patients and their family members/carers should be aware of all the benefits of the physical exercise programme and should be committed to participate. Most patients will benefit from and will be encouraged to undertake at least low to moderate intensity exercise. However, patients with clinically unstable cardiac disease or limiting co-morbid illness will be excluded from exercise training. People whose potential to exercise is limited may have much to gain from the non-exercise components of cardiac rehabilitation.

**5.1. Contraindications to Exercise**

- Unstable Angina
- Unstable Ischemia
- Active pericarditis or myocarditis
- Hypertrophic obstructive cardiomyopathy
- SBP >180 mmHg or DBP >100 mmHg
- BP drop 20 mmHg during incremental exercise
- Resting/uncontrolled tachycardia >100
- Severe and symptomatic aortic stenosis
- Uncontrolled atrial or ventricular arrhythmias
- Severe pulmonary hypertension
- Heart failure that is not compensated
- Recent embolism
- Thrombophlebitis
- Unstable diabetes
- 30 AV block (without pacemaker)
- Febrile illness

**5.2. Exercise sessions.**

- The exercise sessions are held twice a week for 1 hour in St.Bernard's and Southall sport centres each week and patients are encouraged to attend between 8-12 sessions.
- Those patients who wish to participate in the exercise programme need to sign a consent form
- All patients need to attend an initial screening appointment and perform a sub maximal functional capacity test prior to attending the classes.
- Patients will be risk stratified into low, medium and high risk categories as defined by the American Association of Cardiovascular & Pulmonary Rehabilitation (AACVPR) as recommended by American College of Sports Medicine**.** (Appendix 5);"low risk" patients will be enrolled to attend a community sport centre, "moderate/ high risk" patients will be invited to participate in an exercise programme, based in a gym which in located in close proximity to Ealing Hospital.
- Patients, who will not want to attend formal taught sessions will be offered home-based exercise plan.

**Assessment before Exercise Classes:**

- Prior to participation in exercise training patients will undertake a submaximal functional capacity test (e.g. the 6 minutes walk test or shuttle walk test). This will usually be carried out by cardiac physiologist during a separate appointment.
- Prior to submaximal testing of functional capacity a pre-screening checklist will be completed to ensure suitability, an end point of 80% HR max will be determined (adjusted as appropriate for high risk patients) and a rating of perceived exertion of 15, using the Borg Scale category ratio 6-20 scale (Appendix 4). Prior to participation in the exercise test the patient will be familiarized to the Borg scale as below

DRAFT
For peer review only

- Target heart rates for the exercise classes will be set to between 60-75% of the maximal heart rate minus 30 if on Beta Blockers. This range can be adjusted based on risk stratification. The range will be written in patient's exercise plan.

**Before the Class Begins**

- Brief discussion with patients about their progress, home exercises, changes, concerns
- BP and pre-exercise heart rate will be recorded
- Blood glucose levels checked for diabetic patients
- Equipment set up in advance
- Those patients who will complain of feeling generally unwell or become symptomatic or clinically unstable can be excluded from a session for a day. Depending on a condition, the symptoms will be treated with existing medications; patient will be either accompanied to A&E ( if severely unwell) or referred to GP.

**The Exercise Components.**
All sessions should include:

**Warm-Up (15 minutes minimum):** The warm-up period will include graduated low intensity aerobic exercise and short dynamic stretches to increase myocardial blood supply, soft tissue flexibility and mobilize joints.
**Circuit ( 20-30 minutes):** All patients will participate in a progressive exercise training programme, which is modified to meet individual need.
**Cool Down (10 minutes):** This will include graduated low intensity exercise and muscle stretching. Once complete HR will be rechecked and recorded (aim to be within 10 beats of pre-exercise rate)
**Relaxation (15 minutes minimum):** Following the exercise class, patients should be supervised for a 15 minute period.

**Health and Safety Requirements.**

- Each patient will be risk stratified as described above.
- Exercise will be delivered by experienced staff with training in exercise physiology and prescription and an understanding of the specific needs of cardiac patients in relation to exercise.
- 3 members of staff where possible - minimum ratio 1:5. This includes visitors. However priority for exercise is given to patients and if the numbers exceed the safety requirements, visitors will not be able to join in on that occasion.
- Cardiac physiologist to be trained BLS skills (minimum), cardiac rehabilitation nurse-to be present at each session and to be trained at ILS level (minimum)
- Resuscitation equipment available in the gym for the duration of the class.
- All visitors who wish to join in the exercise class need to complete a ParQ
- Venue must be suitable i.e. adequate space, temperature (65-72F,18-23C),
- Drinking water should be available.
- Immediate access to a telephone.
- Annual environmental risk assessment

**Monitoring patients**

- Heart rate monitors to be worn during a patient's first session.
- Heart rates recorded at the beginning of each class, during the class and after the cool down.
- Borg scale of perceived exertion will be recorded during exercise.

**Patients with diabetes**

- Record blood glucose level before the start of the exercise
- Avoid exercise if glucose is over 16mmol/L.
- Avoid exercise if glucose is under 6 and no snack is available prior to exercise
- If glucose >13 and <16 then do warm up and retest – level should fall. If remains >13 and rising should not continue exercise until their status has been stabilised.
- Those taking insulin should avoid injecting into subcutaneous tissue of thigh i.e. avoid sites near to exercising muscle groups.
- Avoid exercise during peak insulin times.

DRAFT
For peer review only

Imperial College London

**Medical Emergencies**

- Nurse to stay with patient
- Exercise specialist to ensure safety of other patients
- Third person to call for help (999 and/or cardiologist)

**DNA Policy**

Patients will be informed about current DNA policy and their obligation to notify a cardiac rehabilitation nurse or cardiac physiologist if they have to miss a session. If a patient does not attend two consecutive classes, contact will be initiated by a member of the Cardiac Rehabilitation team and if no response is received then the patient is discharged from the programme and a letter sent to patient's GP as well as patient.

**End of Programme.**

On completion of the programme patient is given a Certificate stating patients achievements ( see Appendix 6) and a re-screening appointment is made in 2-4 weeks. Patient's GP will receive a letter from Cardiac Rehabilitation Team with all relevant information.

**5.3. Home-Based Programmes**

Those patients who would prefer a home programme or are unable to attend the group sessions will be assessed by the cardiac physiologist and given a suitable physical activity programme. Progress will be monitored regularly and risk factor management will continue as required. This may involve the patient attending regular appointments with the cardiac nurse for blood pressure/heart rate/ blood results monitoring/relevant support and advice. The patient is offered the opportunity to attend the health education talks where possible.

**6. RE-SCREENING OF PATIENTS**

On completion of the health promotion sessions all patients and their families are invited back to the Cardiac Rehabilitation OPD clinic where they will be reassessed as follows:

- Cardiac Risks will be assessed again and progress recorded
- Blood pressure, heart rate, lipids and glucose levels are repeated and recorded
- Those with diabetes will have their HbA1c checked
- Current medications therapy is reviewed
- HAD and QOL is repeated
- 6 MWT or Shuttle walk test is repeated Diet is reassessed and long term recommendations made
- BMI and girth measurement is checked again and recorded

Patients with stable coronary disease will be encouraged to continue regular moderate intensity aerobic exercises. The relevant information about the exercises, stretching techniques, relaxation exercises, and all available sport/leisure centres in the area will be given to patients on discharge. Information about local yoga/dancing/swimming/golf classes, etc. will be available on request. Individual approach will apply, hence if someone will prefer to carry on home based exercises he/she will be supported in their decision. Others, who prefer formal class based cardiac exercise programmes can be referred to the Phase 4 exercise sessions held in St.Bernard and Southall sport centres. The exercise sessions are lead by BACR trained exercise physiologists.

**7. PHASE 4** (Long-term maintenance of changed behaviour)

Long term follow-up in primary care will be arranged.
Involvement with local cardiac support groups or groups of interest (e.g. gardening, cooking, walking, cycling, etc.) will be offered.
Referral to specialist cardiac, behavioural (e.g. exercise, smoking cessation) or psychological services will be made, if clinically indicated.

**8. ANNUAL REVIEW**



For peer review only - http://bmjopen.bmj.com/site/about/guidelines.xhtml

Imperial College London

All patients are invited to attend a follow-up appointment one year after completion of the programme. At this appointment fasting lipids, glucose, and, if appropriate, and HbA1C are measured. Blood pressure is checked twice and anthropometry is recorded. A physical activity and brief dietary assessment are carried out. A summary is sent to the GP and patient with further recommendations if appropriate.

**9. INTERGRATING CARE BETWEEN SECONDARY & PRIMARY CARE.**

A seamless transition between hospital provision of cardiac rehabilitation and the continuing support provided by primary care practitioners requires good communication between all involved in the care of patients with CHD. The primary care team, with detailed knowledge of an individual's social and medical background, includes professionals who are likely to be aware of the implications of CHD for both the individual and their family. Accurate information shared between the various members of multidisciplinary teams across both primary and secondary care will enable the best possible care to be given to the patient.

**10. AUDIT & EVALUATION ( TO AGREE ON EVALUATION OF WORK)**

The Cardiac Rehabilitation service will carry out clinical audit using routinely collected data. Long term goals can be monitored by observing changes over time in incidence and mortality from CHD.

Data will be collected onto the CR database and will also be exported to the national database annually as required for a purpose of NACR. On completion of the programme, patients will be asked to fill in a satisfaction questionnaire.

Standards that we need to follow:

The service should be referred to in the HImP and reflected in long term service agreements.

A clear description of the district cardiac rehabilitation programme should be available to the public, to service providers and to commissioners and should be cited in the HImP.
This description should include details of:

• the patients to be offered cardiac rehabilitation
• staffing (including details of the skills and training required)
• the location and timetable of service provision
• audit criteria
• investment and resources.

Whatever the detail of local rehabilitation services, records should be kept so that the service can be audited against nationally recommended guidelines. This should include information about ethnicity so that it is possible to monitor equity of access. Audit will be easier to undertake if data is stored electronically in a way that allows ready analysis.
National Service Framework – Coronary Heart Disease

**Clinical audit**

Clinical audit – the systematic assessment of the quality of care – is an essential component of modern, high quality health care. It will also be an essential component of effective clinical governance embracing all health professionals.
Trusts should work with their local PCTs and their constituent practices to undertake clinical audit that allows them to review annually the items listed in **bold** below. They may also wish to review the other items when it becomes possible to collect these data.

1) number and % of patients discharged from hospital after coronary revascularisation OR with a primary diagnosis of AMI with documentation of arrangements for cardiac rehabilitation in discharge communication to GP (by Trust and PCG/PCT and by sex,

DRAFT
For peer review only

Imperial College London

age 35-74iii years, and ethnic group)

2) number and % of patients discharged from hospital with a primary diagnosis of CHD recruited to a cardiac rehabilitation programme by Trust and PCG/PCT and by sex, age 35-74iii years, and ethnic group

3) total number and % of those recruited to cardiac rehabilitation who have an individualised plan for rehabilitation and secondary prevention before discharge from hospital
4) total number and % of those recruited to cardiac rehabilitation who, one year after discharge, report:
• regular physical activity of at least 30 minutes duration on average 5 times a week
• not smoking
• BMI < 30 kg/m2.

(NB. PCTs and rehabilitation services may wish to collaborate in the collection, analysis and interpretation of their audit data to avoid duplication of effort and to gain a more complete picture of the quality of rehabilitation and secondary prevention services.)

This Policy will be reviewed and updated if necessary on annual bases.

**REFERENCES**


1. Scottish Intercollegiate Guidelines Network (2002) Cardiac Rehabilitation. A national clinical guideline. SIGN guideline 57. http://tinyurl.com/27g33c

2. National Institute for Health and Clinical Excellence (2007) MI: secondary prevention. Clinical Guideline 48. May. http://tinyurl.com/38tom3

3. British Association of Cardiac Rehabilitation (2007) Standards and Core Components for Cardiac Rehabilitation. http://tinyurl.com/3ydagw

4. Department of Health (2000) Coronary Heart Disease: National Service Frameworks. HMSO: London

5. World Health Organisation (1993) Needs and action priorities in cardiac rehabilitation and secondary prevention in patients with CHD. Geneva: WHO regional office for Europe

6. American College of Sports Medicine (1991) Guidelines for exercise testing and prescription, 4th edn. Lea and Febinger, Philadelphia




For peer review only - http://bmjopen.bmj.com/site/about/guidelines.xhtml

Imperial College London

# Appendix 2

## *Ealing Cardiac Rehabilitation Health Education Talks*

### *Drugs for heart disease and how they work*

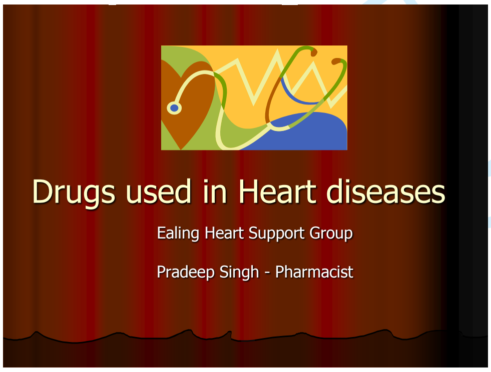


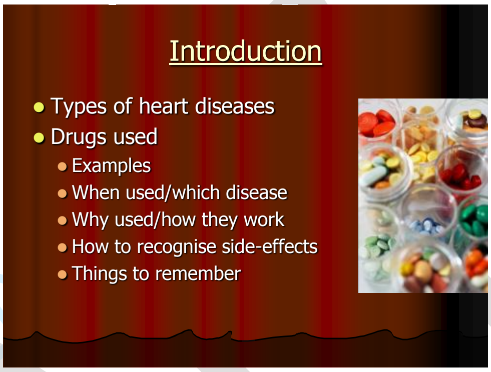


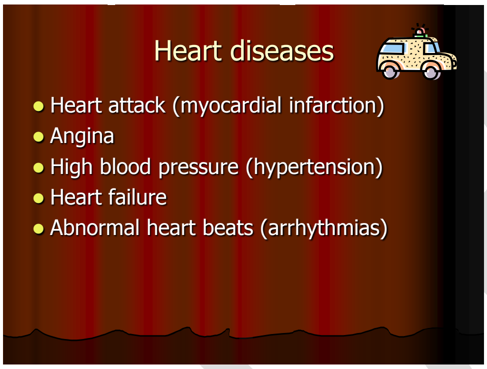


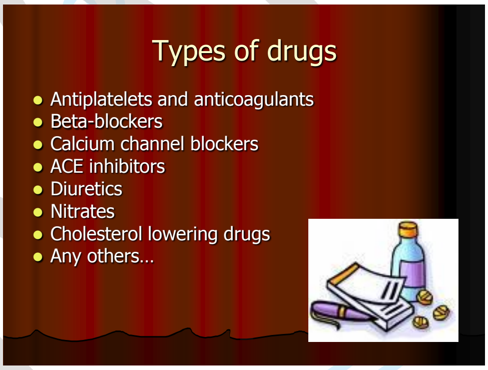


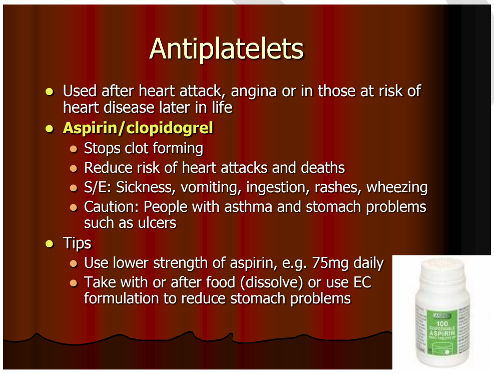


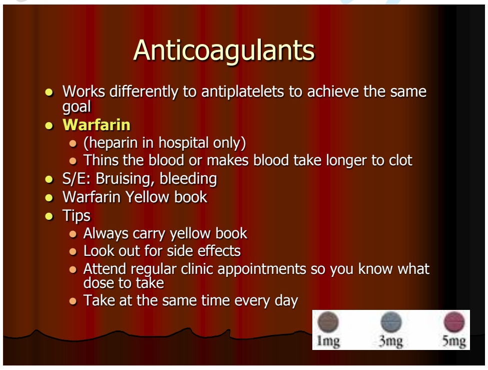




For peer review only - http://bmjopen.bmj.com/site/about/guidelines.xhtml

Imperial College London

## Beta-blockers

- Atenolol, bisoprolol
- Used after heart attack, angina, high BP, abnormal heart beats, heart failure
  - Works in many ways
  - Reduce related deaths
  - S/E: fatigue, wheezing, cold hands/feet, sleep problems, heart failure
  - Caution: Asthma and diabetes
- Tips
  - Do not stop taking them suddenly

## Calcium channel blockers

- Amlodipine, diltiazem
- Angina, high BP
  - Widen blood vessels
- S/E: Headache, upset stomach, ankle swelling, flushing
- Tips
  - Use once daily preparations to reduce s/e. For example, Dilzem XL.

## ACE inhibitors

- Lisinopril, Perindopril
- High BP, after heart attack, heart failure
  - Widen blood vessels
  - Reduce related deaths
- S/E: dry cough, low BP (first dose), taste disturbances, sore mouth, rashes, allergy- type reactions
- Comments: can interfere with kidneys, cause salt disturbances

## Diuretics (water tablets)

- Furosemide, Bumetanide
  - Used in heart failure
- Bendroflumethiazide
  - High BP
  - Reduce fluid by increase volume of urine
- S/E: Gout, worsen diabetes, affect salts
- Comments:
  - Often combined with other drugs
  - Salt disturbances reduced with co-amilofruse
  - Take morning or early afternoon

## Nitrates

- Glyceryl trinitrate, Isosorbide mononitrate
  - Used in Angina treatment and prophylaxis
  - Works by widen the blood vessels in the heart muscle which may be partly blocked
- S/E: Headache (temporary) flushing
- Comments:
  - Tablets/spray for under tongue, patches
  - Tablets to swallow
  - Paracetamol usually helps the headache

## Cholesterol lowering drugs

- Simvastatin, Atorvastatin, Pravastatin
  - After heart attack, angina
  - Reduce cholesterol production
  - Reduce heart disease events
- S/E: Muscle weakness, liver effects, headache
- Comments:
  - Take at night
  - Use with dietary advise
  - Care in liver disease
  - Any muscle problems – contact doctor immediately

## Other medicines

| | How it works | Side effects | Interactions |
|---|---|---|---|
| Digoxin | Increases force that heart pumps blood and reduces heart rate | Nausea, vomiting, slow pulse | Levels in the blood are increased by amiodarone, diltiazem, verapamil, dihydropyridines |
| Amiodarone | Antiarrhythmic – used to correct irregular heart rhythms | Sun sensitivity. Changes in thyroid function. Deposits in the cornea | Increases digoxin and warfarin. If given with other antiarrhythmics get and additive effect |
| Warfarin | Thins the blood | Bruising, bleeding (nose, urine, etc) | Effect increased by alcohol, antibiotics, amiodarone, cimetidine, simvastatin |

## What are medicines for the heart used for?

| | Nitrate | Beta blockers | Calcium channel blockers | ACE inhibitor | Diuretic | Digoxin |
|---|---|---|---|---|---|---|
| Angina | Y | Y | Y | | | |
| Raised blood pressure | | Y | Y | Y | Y | |
| Heart failure | Y | | | Y | Y | Y |
| Arrhythmias | | Y | Y | | | Y |


For peer review only - http://bmjopen.bmj.com/site/about/guidelines.xhtml

Imperial College
London

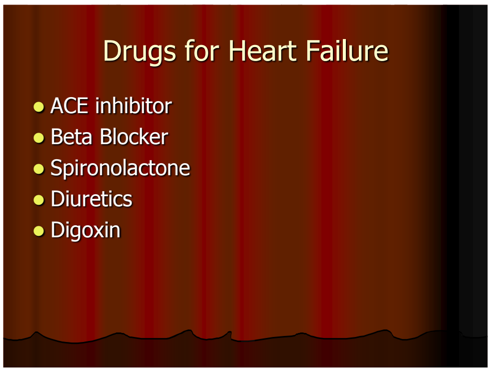


*Managing Stress*

## Dealing with Stress

Dr. Kushangi Patel
Clinical Psychologist

## What is Stress?

**Physical Definition**

- A reaction that occurs in the body in response to a threat
- **Fight** the body gets ready to fight the threat
- **Flight** the body gets ready to run away

## What is Stress?

**Psychological Definition**

- Feeling under pressure
- Feeling unable to cope with the demands placed on us
- Feeling unable to adapt to demands

## How do you know when you are stressed?

## How do you know when you are stressed?

**Physical signs**

- Heart beats faster
- Faster breathing
- Muscle tension
- Increased sweating
- Feeling sick, indigestion, butterflies
- Dry mouth
- Increasingly needing the toilet
- Feeling dizzy

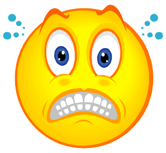

## How do you know when you are stressed?

**Psychological Signs**

- Memory difficulties
- Difficulty concentrating
- Muddled thinking
- Difficulty making decisions
- Becoming increasingly disorganised
- Irritability
- Low self confidence
- Social withdrawal
- Increasing tiredness

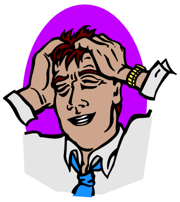

Imperial College London

## Purpose of stress

- Stress does have a purpose it's not all bad
- To protect ourselves from threat
- A small amount of stress helps increase our performance at things
- Stress can drive us to do things in life

## What makes you stressed?

**Environment**

- Work, family, too many social demands, driving, money, not enough opportunity for enjoyment

**Thoughts**

- I can't cope! I haven't got time! If I don't get this done….. will happen!
- These are e.g's of negative thoughts and predicting worse case scenarios.

## What causes stress in your life?

## What makes you stressed

**Emergency**

- Threat to life/ safety
- Events that pose an immediate threat to us cause stress levels to increase sharply and then fall quickly

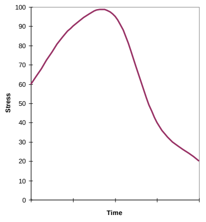


## What makes you stressed

**Daily Hassles**

- Such as shopping, organising holidays/social events, keeping on top of daily tasks at home and/or work.
- These frequently affect our stress levels, increasing it and decreasing it as hassles are encountered and then dealt with.
- Watch out for daily hassles – these lead to a gradual but **regular** build up of stress.

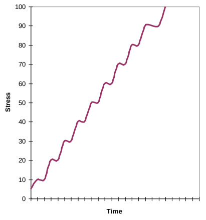


## How stress causes health problems

- Stress causes heart rate and blood pressure to increase and hormones are released to enable us to fight or flight.
- If high and/or frequent stress is experienced and these hormones are not used up by physical activity, the increase in heart rate and blood pressure damages arteries.
- The body heals this damage but as a result the artery walls become thicker and scarred.
- This can affect the supply of blood and oxygen to the heart.

## How health problems cause stress

- Health problems can affect all areas of life.
- Physical – feeling more tired, less able to do the things accomplished before the health problems started.
- Financial - time off work or a need to leave work altogether may have financial implications.
- Interests – a persons ability to engage in things they enjoy may be reduced temporarily or certain hobbies may have to be adapted.

## How health problems cause stress

- Identity – we all have views of ourselves. E.g. independent, responsible, fun, healthy. A health problem may affect this view if we feel we are no longer able to "be" these things.
- All these aspects can lead to feelings of frustration, anger and sadness.
- This can all increase stress levels
- Talk to other people about these feelings and frustrations or seek help from professionals, GP, Cardiac Rehab Nurse.


For peer review only - http://bmjopen.bmj.com/site/about/guidelines.xhtml

Imperial College London

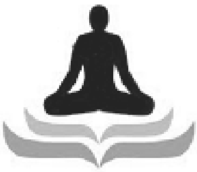

## Health problems and stress

- Heart problems can knock self confidence so it is important to do things at a comfortable pace and build up gradually.
- Work to balance doing things at YOUR pace. Doing nothing and doing too much are both unhelpful.
- Take advice from GP's, cardiac team on how much you can do and when.
- Watch out for negative thoughts as they can lead you into a vicious cycle of inactivity and low mood which all can increase your stress levels.

## Vicious cycle of negative thoughts

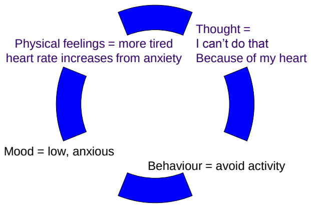


## What are the consequences of stress?

## Consequences of stress

- Health problems
- Difficulty in relationships
- Increase in sick leave
- Being unable to do things you need to do or enjoy
- Feeling upset, angry, frustrated, out of control

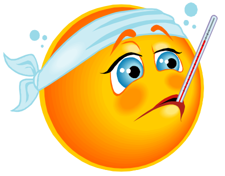

## Exercise

- In pairs talk about how you have coped with stress.
- What sort of things have you done in response to stress?
- What has worked?
- What has not worked so well?

## Coping with stress

**Helpful ways**

- Exercise
- Talk to someone
- Look at my thinking
- Prioritise tasks
- Share the load
- Relaxation
- Get guidance from other people or professionals involved in your care

**Unhelpful Ways**

- Increase alcohol/other drugs
- Work longer hours/ harder to finish tasks
- Shout
- Be on my own
- Ignore it

## Coping with stress

**Notice your early warning signs** – these may be physical signs and/or psychological signs

**Take action**

- **Calm your body by** –
- Relaxation
- Regular physical activity helps keep overall stress levels low
- Eat healthily
- Do something you enjoy
- Rest if you're tired
- Avoid increasing alcohol, smoking or caffeine to cope

## Coping with stress

- **Calm your mind by** –
- Relaxation
- Looking at your thoughts:
  Are you focusing on the negative?
  Are you predicting the worse case scenario without any evidence for it?
- Plan and prioritise
- Think about the long-term – is saying no now going to help you later on

**It may be difficult to do all the techniques so find out what works for you!**

For peer review only

*Eating for a healthy heart*

This is an interactive session delivered by a cardiac specialist dietician – no presentation available.

DRAFT
For peer review only


For peer review only - http://bmjopen.bmj.com/site/about/guidelines.xhtml

Imperial College
London

*Exercise and the benefits for your heart*

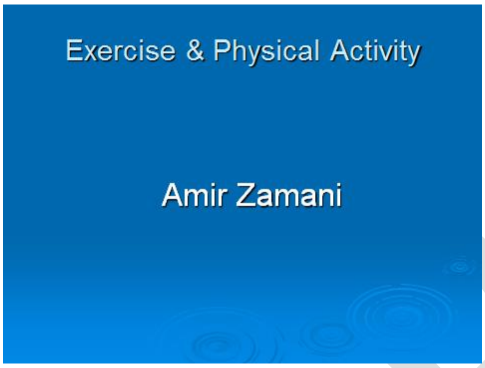


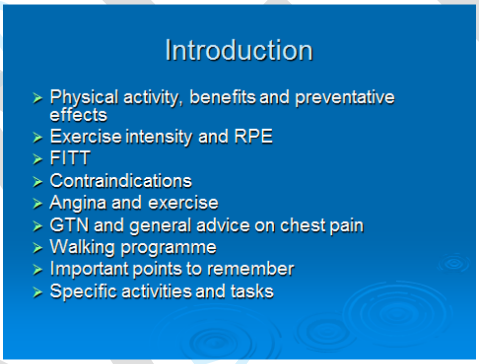


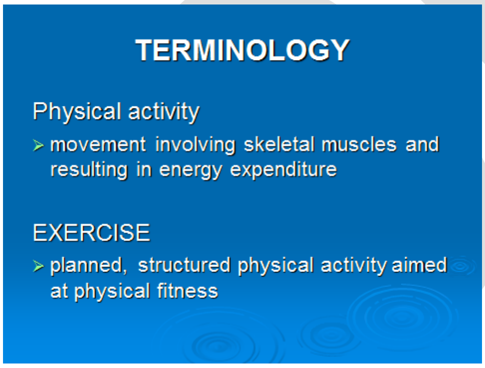


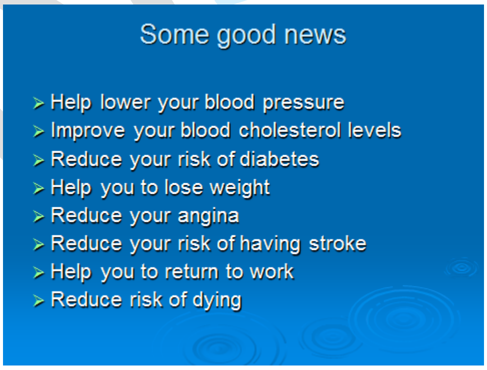


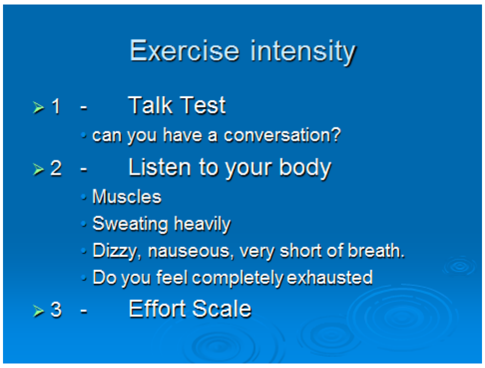


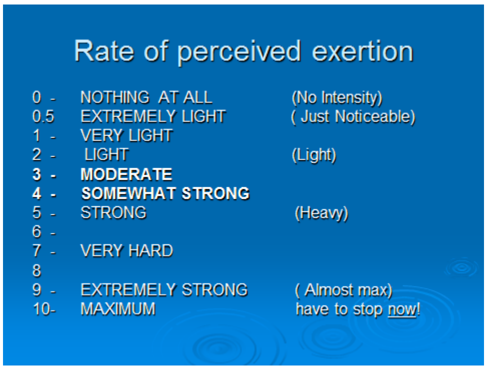

Imperial College London

## FITT

- Frequency — Most days
- Intensity — Moderate
- Time — 30-40 minutes
- Type — Aerobic

## STOP if you experiencing any:

- Undue shortness of breath
- Chest pain/discomfort (or pain in your neck/jaw/arm)
- Nausea/headaches/dizziness
- Inappropriate tiredness
- Persistent palpitations
- Feeling unwell

## Angina and exercise

- Angina is often described as a tightness, heaviness or dull sensation in the chest
- It is usually brought on by exertion
- This is the way your heart saying that it is not getting enough oxygen
- It is particularly important to let your GP know if you are getting angina for the first time

## What should I do if I get angina?

- The first thing that you need to do is STOP what you are doing and rest
- If you are given GTN spray or tablets it is important to use this medications

## Remember the following

Angina pain/discomfort

Rest
1-2 GTN Spray/tablets under the tongue

If the pain is not relieved after 5 minutes
1-2 GTN Spray/tablets under the tongue

If the pain is not relieved after 5 minutes
1-2 GTN Spray/tablets under the tongue

If the pain is nor relieved after 5 minutes
Call 999 for an ambulance

## General advice on chest pain

- If you have GTN spray carry it with you at all time
- If you have access to mobile phone, it may be a good idea to carry with you
- If you know of a activity that you know bring on angina, you can take your GTN before the commencing that activity
- Seated while you take your GTN
- Do not stop taking your GTN because of your headache
- Do not be afraid of using your GTN spray

## How do I do Warm-Up?

Should be low level/
Nice and easy

10-15 minutes

Pulse raising activity and stretching

## Warming Up and Cooling Down

WHY WARM UP?

Prepare muscles for
activity - ↓ injury

Prepare heart for activity
- ↓ angina
- ↓ disturbances in heart rhythm

For peer review only

Imperial College London

## What sort of activity

- Aerobic, most beneficial activity for your heart
- Resistance or strength training

## Walking programme

| Stage of recovery | Length of walk (in minute) |
|---|---|
| (Approx. week 1) | 5 Minutes: several times per day Strolling/leisurely pace |
| (approx. week 2) | 10 minutes: twice a day, Leisurely pace |
| (approx. week 3) | 15 minutes: daily, Leisurely/moderate pace |
| (approx. week 4) | 20 minutes: daily, moderate pace |
| (approx. week 5) | 25 minutes: daily, brisk pace |
| (approx. week 6) | 30 minutes: daily brisk pace |
| Target | 30-45 minutes: daily brisk walk |

## Cool Down

WHY COOL DOWN?

↓ Fainting and dizziness

↓ Disturbances to your heart

↓ Muscle soreness

## How do I cool Down?

Goal is to return body to its resting state

Gradually slow down the activity you are doing and stretch

10 minutes

## Sensible Precautions

- Do not exercise if you feel unwell
- Do not exercise on a full or empty stomach
  - Light meal/snack 1½ - 2 hours before
- Do not exercise in extreme temperatures
- Wear suitable clothing
- Take your medications
- Good days and Bad days
- Enjoy!

*Have an active, healthy, happy life!*

For peer review DRAFT

Imperial College
London

*Risk factors and making lifestyle changes*

## Risk Factors & Making Changes

Olivia Molloy
Cardiac Rehabilitation Sister

## Modifiable and Non Modifiable Risk Factors

| • Modifiable | • Non Modifiable |
|---|---|
| 1. Smoking<br>2. High Blood Pressure<br>3. High Cholesterol<br>4. Physical Inactivity<br>5. Being Overweight<br>6. Diabetes<br>7. Alcohol consumption<br>8. Impaired glucose regulation | 1. Family History of Heart Disease<br>2. Age<br>3. Ethnic Background |

## Smoking

FACT

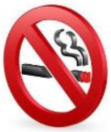

- Smoking is one of the major causes of cardiovascular disease. People who smoke are twice as likely to have a heart attack as to people who have never smoked

## Smoking

- Smoking damages the lining of the arteries leading to build up of Atheroma.
- Carbon Monoxide in cigarette smoke reduces the amount of oxygen that the blood can carry to the heart and around the body
- Nicotine stimulates adrenaline which increases heart rate and raises blood pressure = harder work load for your heart
- Second Hand Smoking…..

## Blood Pressure

- Blood Pressure represents the pressure of the blood in your arteries. We need a certain amount of pressure in our body in order to keep our blood ( circulation) flowing

- High Blood Pressure is also known as

**"Hypertension"**

This is when your blood pressure is higher than the recommended level.

## Blood Pressure

- What does Systolic and Diastolic Pressure Mean?
- Readings:
  - 140/80mmhg = Non Diabetics
  - Diabetics = 130/80mmhg

**Maximum**



For peer review only - http://bmjopen.bmj.com/site/about/guidelines.xhtml

Imperial College London

## Blood Pressure

- Why is High Blood Pressure dangerous

Remember

- High Blood Pressure does not make you feel Sick.

## Cholesterol

- Cholesterol is a fatty substance which is found in the blood. Too much cholesterol is a contributing factor for Heart Disease.
- **LDL**
- **HDL**
- **Total Cholesterol**
- **Triglycerides**

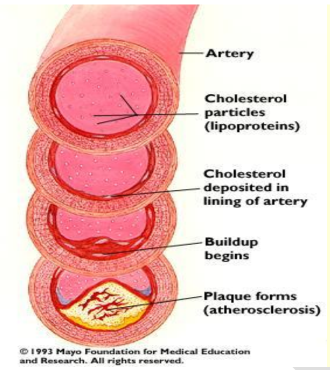


## Excercise

- In the UK people who are not physically active are twice as likely to have a heart attack
- In the UK; 7 out of 10 people do not do physical activity to benefit their health
- What can you do?

## Keeping a Healthy Weight & Shape

- Being overweight can increase your risk of developing Cardiovascular disease.
- Keeping close to a healthy weight will help you control your blood pressure and reduce the workload that you hear has to do.

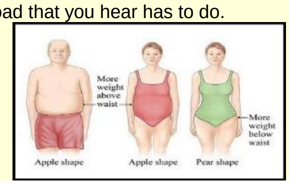


## Family History

- If you have a family history of CVD your own risk of developing the condition is increased.
- A family history means if your

father or brother >55

or

mother or sister >65

- Non Modifiable risk factor

## Eating Healthy For Your Heart

- Eat plenty of fruit and veg
- Choose healthier fats
- Eat oily fish regularly
- Reduce the amount of salt you eat

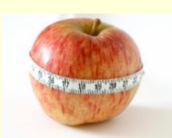

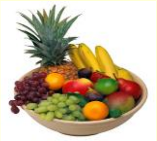

## Alcohol

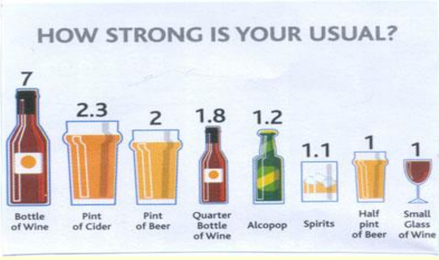

Imperial College London

## Stress

- Stress is not a direct risk factor for CVD but it is possible that stress may contribute to it, or perhaps bring on some symptons.
- The way you deal with stress can encourage unhealthy behaviours e.g. smoking unhealthy eating , alcohol etc

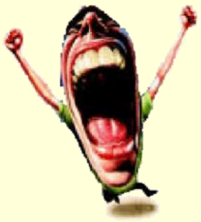

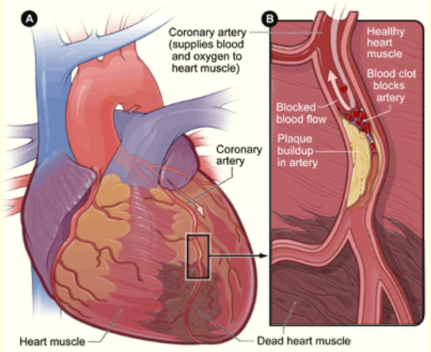


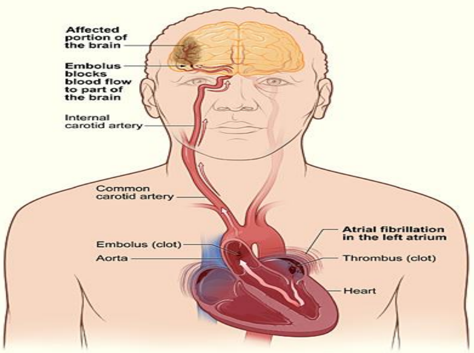


DRAFT
For peer review only

Imperial College London

*The heart and how it works*

## The Heart & How it works

Olivia Molloy
Cardiac Rehabilitation Nurse Specialist

## The Human Heart

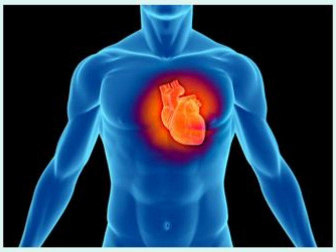

## The Heart

- The heart is a fist sized organ which lies within the chest behind the sternum (breast bone). The heart sits on the diaphragm, the main muscle of breathing, which is found beneath the lungs. The heart is considered to have two 'sides' – the right side and the left side.

  The heart has four chambers – an atria and ventricle on each side.

- The atria are both supplied by large blood vessels that bring blood to the heart (see below for more details).

- Atria have special valves that open into the ventricles. The ventricles also have valves but in this case they open into blood vessels. The walls of the heart chambers are made mainly of special heart muscle. The different sections of the heart have to contract (squeeze) in the correct order for the heart to pump blood efficiently with each heartbeat

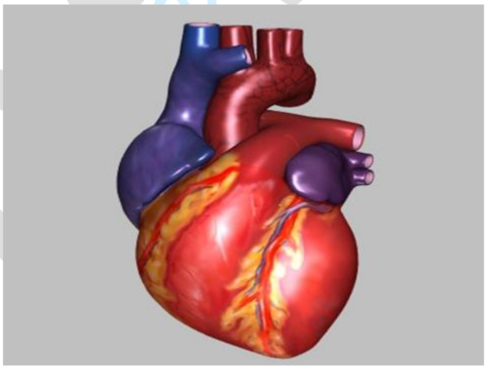

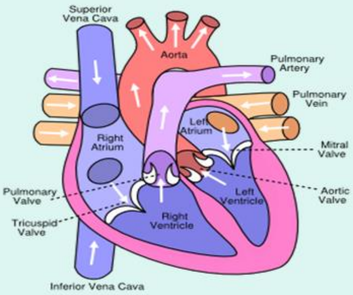


## The Function of the Heart

- The heart is a muscular pump that pushes blood through blood vessels around the body.

- Essential to life, the heart beats continuously, pumping the equivalent of more than 14,000 litres of blood every day.

  Blood vessels form the living system of tubes that carry blood both to and from the heart.

- All cells in the body need oxygen and the vital nutrients found in blood. Without oxygen and these nutrients, the cells will die.


For peer review only - http://bmjopen.bmj.com/site/about/guidelines.xhtml

Imperial College London

## The Heart Cont…

- The heart helps to provide oxygen and nutrients to the body's tissues and organs by ensuring a rich supply of blood.
- Not only do blood vessels carry oxygen and nutrients, but they also transport carbon dioxide and waste products away from our cells.
- Carbon dioxide is passed out of the body by the lungs, and most of the other waste products are disposed of by the kidneys.

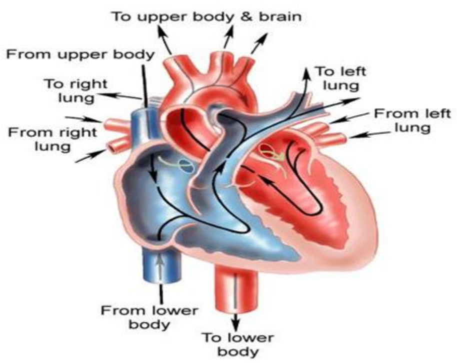


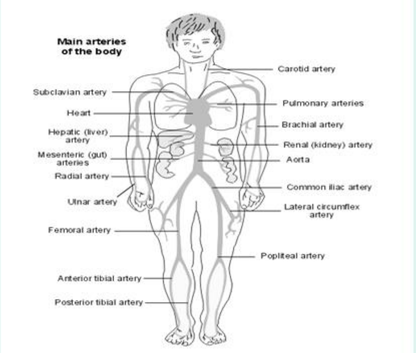


## The Blood Supply to the Heart

- Like any other muscle, the heart muscle needs a good blood supply. The coronary arteries take blood to the heart muscle. These are the first arteries to branch off the aorta - the large artery that takes blood to the body from the left ventricle.

  The right coronary artery mainly supplies the muscle of the right ventricle.

  The left coronary artery quickly splits into two and supplies the rest of the heart muscle.

  The main coronary arteries divide into many smaller branches to supply all the heart

## The coronary arteries of the heart

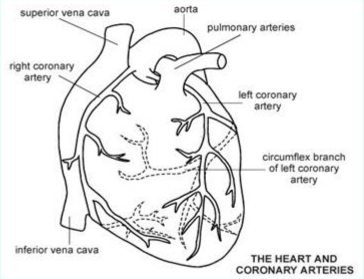


## Angioplasty

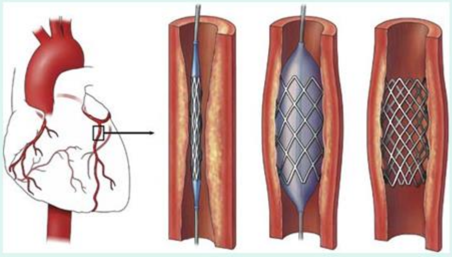

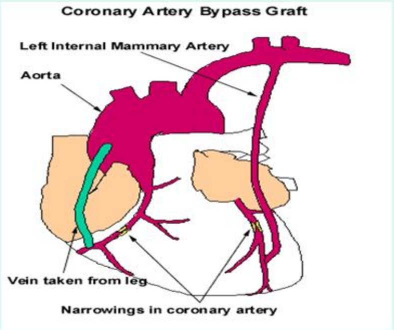


## The Heart Valves

The heart also contains four valves.

- Their role is to ensure the blood flows in a forward direction and prevents a backward flow during any part of the pump action (or cardiac cycle).
- An atrioventricular valve sits on both the left and right sides of the heart between each atrium and ventricle.
- These are the tricuspid valve (right side) and the mitral valve (left side).
- The two remaining valves sit on the outflow tract of the left and right ventricles.


For peer review only - http://bmjopen.bmj.com/site/about/guidelines.xhtml

Imperial College London

## Valves Cont….

- The pulmonary valve is between the right ventricle and the pulmonary artery, which takes deoxygenated blood to the lungs.
- The aortic valve sits between the left ventricle and the aorta, which takes oxygenated blood to the body's tissues.
- These latter two valves are semilunar; they contain three cusps which close to prevent the backward flow of blood from the outflow vessels during the diastolic (filling) phase of the cardiac cycle. The left side of the heart is inevitably under a much higher pressure than the right side, which delivers blood to the lungs only.

## Heart Valves

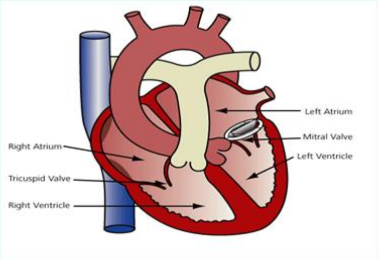


## Chest Pain

- Stop what your doing
- Sit Down and rest
- If you have GTN spray or tablets, use the spray and take your tablets as instructed by your doctor or cardiac rehab nurse

## Chest Pain Continued

- If you don't have GTN CALL 999 if pain does not go away.
- Aspirin , if you are not allergic to aspirin chew 300mgs until the ambulance arrives
- If the pain, discomfort or chest tightness continues especially if its gone on within 15 minutes
- DONT WAIT CALL 999 RIGHT AWAY

## CALL THE AMBULANCE AND STAY RESTING

## Basic Life Support

- Show DVD Recording here

## Blood Pressure / Pulse

- Non Diabetic Patients — 140/80 mmhg
- Diabetic Patients — 130/80 mmhg
- 
- Pulse for all — •60 – 100 bpm

## Diabetics

- All Diabetics Blood Sugar should be less than

6

Imperial College London

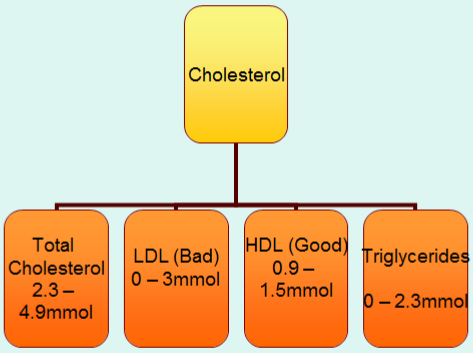


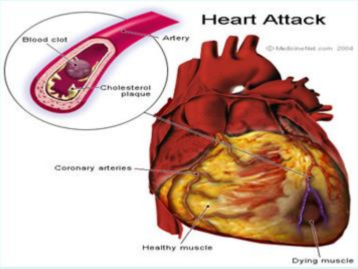


## Cont.…

- Arterioles are the smallest arteries in the body. They deliver blood to capillaries. Arterioles are also capable of constricting or dilating and by doing this they control how much blood enters the capillaries.

  Capillaries are tiny vessels that connect arterioles to venules. They have very thin walls which allow nutrients from the blood to pass into the body tissues. Waste products from body tissues can also pass into the capillaries. For this reason capillaries are known as exchange vessels.

  Groups of capillaries within a tissue reunite to form small veins called venules. Venules collect blood from capillaries and drain into veins.

  Veins are the blood vessels that carry blood back to the heart. They may contain valves which stop blood flowing away from the heart.

## Cont.…

- The right side of the heart receives deoxygenated blood (lacking oxygen) from the body. After passing through the right atrium and right ventricle this blood is pumped to the lungs. Here blood picks up oxygen and loses another gas called carbon dioxide. Once through the lungs, the blood flows back to the left atrium. It then passes into the left ventricle and gets pumped into the aorta, the main artery supplying the body. Oxygenated blood is then carried though blood vessels to all the body's tissues. Here oxygen and other nutrients pass into the cells where they are used to perform the body's essential functions.

  A blood vessels main function is to transport blood around the body. Blood vessels also play a role in controlling your blood pressure.

  Blood vessels are found throughout the body. There are five main types of blood vessels: arteries, arterioles, capillaries, venules and veins.

  Arteries carry blood away from the heart to other organs. They can vary in size. The largest arteries have special elastic fibres in their walls. This helps to complement the work of the heart, by squeezing blood along when heart muscle relaxes. Arteries also respond to signals from our nervous system, either constricting (tightening) or dilating (relaxing).

## Blood Flow Through the Body

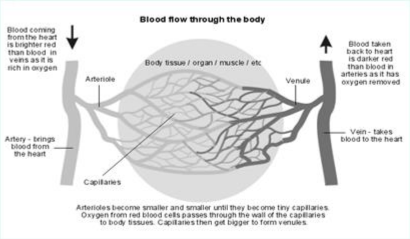

Imperial College London

## References

1. Sivananda: Yoga Teachers' Training Manual

2. Sivananda Yoga Vedanta Centre: Yoga-Your Home Practice Companion

3. Sivananda Yoga Vedanta centre: The Yoga Cookbook:Vegetarian Food for Body and Mind

4. H.David Coulter: Anatomy of Hatha Yoga

5. Swami Vishnu-Devananda: The Complete Illustrated Book of Yoga

6. www.yoga.about.com

7. www.innerhealthstudio.com

DRAFT
For peer review only

# CONSORT 2010 checklist of information to include when reporting a randomised trial*

| Section/Topic | Item No | Checklist item | Reported on page No |
|---|---|---|---|
| **Title and abstract** | | | |
| | 1a | Identification as a randomised trial in the title | 1 |
| | 1b | Structured summary of trial design, methods, results, and conclusions (for specific guidance see CONSORT for abstracts) | 2 |
| **Introduction** | | | |
| Background and objectives | 2a | Scientific background and explanation of rationale | 3 |
| | 2b | Specific objectives or hypotheses | 3-4 |
| **Methods** | | | |
| Trial design | 3a | Description of trial design (such as parallel, factorial) including allocation ratio | 5 |
| | 3b | Important changes to methods after trial commencement (such as eligibility criteria), with reasons | 5 |
| Participants | 4a | Eligibility criteria for participants | 5 |
| | 4b | Settings and locations where the data were collected | 9 |
| Interventions | 5 | The interventions for each group with sufficient details to allow replication, including how and when they were actually administered | 5-6, and supplementary files, 'Yacht Study package_v1_2, which includes 'Policy for Rehabilitation in Ealing' |
| Outcomes | 6a | Completely defined pre-specified primary and secondary outcome measures, including how and when they were assessed | 6-9 |
| | 6b | Any changes to trial outcomes after the trial commenced, with reasons | N/A |
| Sample size | 7a | How sample size was determined | 9 |
| | 7b | When applicable, explanation of any interim analyses and stopping guidelines | N/A |

For peer review only


For peer review only - http://bmjopen.bmj.com/site/about/guidelines.xhtml

| | | | |
|---|---|---|---|
| Randomisation: | | | |
| Sequence generation | 8a | Method used to generate the random allocation sequence | 5 |
| | 8b | Type of randomisation; details of any restriction (such as blocking and block size) | 5 |
| Allocation concealment mechanism | 9 | Mechanism used to implement the random allocation sequence (such as sequentially numbered containers), describing any steps taken to conceal the sequence until interventions were assigned | 5 |
| Implementation | 10 | Who generated the random allocation sequence, who enrolled participants, and who assigned participants to interventions | 6 |
| Blinding | 11a | If done, who was blinded after assignment to interventions (for example, participants, care providers, those assessing outcomes) and how | 8-9 |
| | 11b | If relevant, description of the similarity of interventions | N/A |
| Statistical methods | 12a | Statistical methods used to compare groups for primary and secondary outcomes | 9-10 |
| | 12b | Methods for additional analyses, such as subgroup analyses and adjusted analyses | 9-10 |
| **Results** | | | |
| Participant flow (a diagram is strongly recommended) | 13a | For each group, the numbers of participants who were randomly assigned, received intended treatment, and were analysed for the primary outcome | Figure 1_consort flow diagram_YACHT |
| | 13b | For each group, losses and exclusions after randomisation, together with reasons | Figure 1_consort flow diagram_YACHT |
| Recruitment | 14a | Dates defining the periods of recruitment and follow-up | 5 |
| | 14b | Why the trial ended or was stopped | 5 |
| Baseline data | 15 | A table showing baseline demographic and clinical characteristics for each group | Table 1, Supplemental tables |
| Numbers analysed | 16 | For each group, number of participants (denominator) included in each analysis and whether the analysis was by original assigned groups | Figure 1_consort flow |

For peer review only



For peer review only - http://bmjopen.bmj.com/site/about/guidelines.xhtml

| | | | |
|---|---|---|---|
| | | | diagram_YACHT. Tables 2-5 |
| Outcomes and estimation | 17a | For each primary and secondary outcome, results for each group, and the estimated effect size and its precision (such as 95% confidence interval) | Tables 2-5 |
| | 17b | For binary outcomes, presentation of both absolute and relative effect sizes is recommended | N/A |
| Ancillary analyses | 18 | Results of any other analyses performed, including subgroup analyses and adjusted analyses, distinguishing pre-specified from exploratory | N/A |
| Harms | 19 | All important harms or unintended effects in each group (for specific guidance see CONSORT for harms) | 10 |
| **Discussion** | | | |
| Limitations | 20 | Trial limitations, addressing sources of potential bias, imprecision, and, if relevant, multiplicity of analyses | 14 |
| Generalisability | 21 | Generalisability (external validity, applicability) of the trial findings | 14 |
| Interpretation | 22 | Interpretation consistent with results, balancing benefits and harms, and considering other relevant evidence | 14 |
| **Other information** | | | |
| Registration | 23 | Registration number and name of trial registry | 2 |
| Protocol | 24 | Where the full trial protocol can be accessed, if available | Available on request/file uploaded |
| Funding | 25 | Sources of funding and other support (such as supply of drugs), role of funders | 15 |

*We strongly recommend reading this statement in conjunction with the CONSORT 2010 Explanation and Elaboration for important clarifications on all the items. If relevant, we also recommend reading CONSORT extensions for cluster randomised trials, non-inferiority and equivalence trials, non-pharmacological treatments, herbal interventions, and pragmatic trials. Additional extensions are forthcoming: for those and for up to date references relevant to this checklist, see www.consort-statement.org.

For peer review only
